# Supplementary material for: T-Racing: a modern tool for supporting epidemiological investigation in animal disease outbreaks in Italy
Source: PLoS One. 2025 Feb 19;20(2):e0317313. doi: 10.1371/journal.pone.0317313 (PMC11838889; doi:10.1371/journal.pone.0317313)
Supplement: Attachment — Submitted filename: PONE-D-24-01354_review_v1.pdf [file pone.0317313.s001.pdf]

PLOS ONE

T-Racing: a modern tool for supporting epidemiological investigation in animal disease outbreaks

--Manuscript Draft--

**Manuscript Number:**	PONE-D-24-01354
**Article Type:**	Research Article
**Full Title:**	T-Racing: a modern tool for supporting epidemiological investigation in animal disease outbreaks
**Short Title:**	T-Racing epidemiological web application
**Corresponding Author:**	Luca Candeloro, M.D.<br>Istituto Zooprofilattico Sperimentale dell'Abruzzo e del Molise G Caporale<br>Teramo, ITALY
**Keywords:**	One health; Epidemiology; Tracing; temporal network analysis; Shiny web application; outbreak investigation; Zoonoses management.
**Abstract:**	Background: The One Health approach unites efforts at the human-animal-environment interface to address shared threats such as zoonotic diseases. In this context, T-Racing, a modern web tool, plays a vital role in epidemiological investigations and the containment of livestock-related disease spread. It aligns with One Health principles to safeguard public health. Aim: This study aims to showcase how T-Racing, in alignment with the One Health approach, utilizes Temporal Network Analysis techniques to comprehensively understand disease transmission patterns, ultimately facilitating swift and effective interventions. Methods: T-Racing utilizes Temporal Network Analysis techniques to account for the dynamic nature of animal trade and perform rapid and efficient back-and-forward tracing procedures. T-Racing leverages web services to retrieve data from multiple sources simultaneously and in near real-time, utilizing the plumber package. The tool is distributed using Shinyproxy and manages diverse datasets, including animal movement data, livestock owner and producer information, disease outbreak data, and genomic data obtained from national databases within a unified environment. Results: This study showcases T-Racing's capabilities through the simulation of epidemiological investigations into Brucellosis and Tuberculosis outbreaks that occurred in non-endemic areas of Italy. Conclusions: T-Racing, by integrating Temporal Network Analysis and web-based data retrieval, emerges as a valuable tool for One Health-driven epidemiological investigations. It makes easier and faster data analysis and enables a comprehensive understanding of disease transmission patterns facilitating prompt interventions.
**Order of Authors:**	Luca Candeloro, M.D.
	Lara Savini
	Annamaria Conte
	Paolo Calistri
	Diana Palma
	Samuel Perticara
	Alessio Di Lorenzo
	Susanna Tora
	Sara Serrani
	Michela Toro
	Fabrizio De Massis
**Additional Information:**	
**Question**	**Response**
**Financial Disclosure**	Yes

Powered by Editorial Manager® and ProduXion Manager® from Aries Systems Corporation

Enter a financial disclosure statement that describes the sources of funding for the work included in this submission. Review the submission guidelines for detailed requirements. View published research articles from *PLOS ONE* for specific examples.<br><br>This statement is required for submission and **will appear in the published article** if the submission is accepted. Please make sure it is accurate.<br><br>**Funded studies**<br>Enter a statement with the following details:<br>• Initials of the authors who received each award<br>• Grant numbers awarded to each author<br>• The full name of each funder<br>• URL of each funder website<br>• Did the sponsors or funders play any role in the study design, data collection and analysis, decision to publish, or preparation of the manuscript?<br><br>Did you receive funding for this work?	
Please add funding details.<br>as follow-up to "**Financial Disclosure**<br><br>Enter a financial disclosure statement that describes the sources of funding for the work included in this submission. Review the submission guidelines for detailed requirements. View published research articles from *PLOS ONE* for specific examples.<br><br>This statement is required for submission and **will appear in the published article** if the submission is accepted. Please make sure it is accurate.	This research was funded by Italian Ministry of Health (MSRCTE0219).

Powered by Editorial Manager® and ProduXion Manager® from Aries Systems Corporation

**Funded studies**<br>Enter a statement with the following details:<br>• Initials of the authors who received each award<br>• Grant numbers awarded to each author<br>• The full name of each funder<br>• URL of each funder website<br>• Did the sponsors or funders play any role in the study design, data collection and analysis, decision to publish, or preparation of the manuscript?<br><br>Did you receive funding for this work?"	
Please select the country of your main research funder (please select carefully as in some cases this is used in fee calculation).<br>as follow-up to "**Financial Disclosure**<br><br>Enter a financial disclosure statement that describes the sources of funding for the work included in this submission. Review the submission guidelines for detailed requirements. View published research articles from *PLOS ONE* for specific examples.<br><br>This statement is required for submission and **will appear in the published article** if the submission is accepted. Please make sure it is accurate.<br><br>**Funded studies**<br>Enter a statement with the following details:<br>• Initials of the authors who received each award<br>• Grant numbers awarded to each author<br>• The full name of each funder<br>• URL of each funder website<br>• Did the sponsors or funders play any role in the study design, data collection and analysis, decision to publish, or preparation of the manuscript?<br><br>Did you receive funding for this work?"	ITALY - IT
**Competing Interests**	The authors have declared that no competing interests exist

Use the instructions below to enter a competing interest statement for this submission. On behalf of all authors, disclose any competing interests that could be perceived to bias this work—acknowledging all financial support and any other relevant financial or non-financial competing interests.<br><br>This statement is required for submission and **will appear in the published article** if the submission is accepted. Please make sure it is accurate and that any funding sources listed in your Funding Information later in the submission form are also declared in your Financial Disclosure statement.<br><br>View published research articles from *PLOS ONE* for specific examples.<br><br>**NO authors have competing interests**<br><br>Enter: *The authors have declared that no competing interests exist.*<br><br>**Authors with competing interests**<br><br>Enter competing interest details beginning with this statement:<br><br>*I have read the journal's policy and the authors of this manuscript have the following competing interests: [insert competing interests here]*<br><br>* typeset	
**Ethics Statement**<br><br>Enter an ethics statement for this submission. This statement is required if the study involved:<br><br>• Human participants<br>• Human specimens or tissue<br>• Vertebrate animals or cephalopods<br>• Vertebrate embryos or tissues	N/A

Powered by Editorial Manager® and ProduXion Manager® from Aries Systems Corporation

• Field research<br><br>Write "N/A" if the submission does not require an ethics statement.<br><br>General guidance is provided below. Consult the submission guidelines for detailed instructions. **Make sure that all information entered here is included in the Methods section of the manuscript.**	

Powered by Editorial Manager® and ProduXion Manager® from Aries Systems Corporation

**Format for specific study types**<br><br>**Human Subject Research (involving human participants and/or tissue)**<br>• Give the name of the institutional review board or ethics committee that approved the study<br>• Include the approval number and/or a statement indicating approval of this research<br>• Indicate the form of consent obtained (written/oral) or the reason that consent was not obtained (e.g. the data were analyzed anonymously)<br><br>**Animal Research (involving vertebrate animals, embryos or tissues)**<br>• Provide the name of the Institutional Animal Care and Use Committee (IACUC) or other relevant ethics board that reviewed the study protocol, and indicate whether they approved this research or granted a formal waiver of ethical approval<br>• Include an approval number if one was obtained<br>• If the study involved *non-human primates*, add *additional details* about animal welfare and steps taken to ameliorate suffering<br>• If anesthesia, euthanasia, or any kind of animal sacrifice is part of the study, include briefly which substances and/or methods were applied<br><br>**Field Research**<br><br>Include the following details if this study involves the collection of plant, animal, or other materials from a natural setting:<br>• Field permit number<br>• Name of the institution or relevant body that granted permission	
**Data Availability**<br><br>Authors are required to make all data underlying the findings described fully available, without restriction, and from the time of publication. PLOS allows rare exceptions to address legal and ethical concerns. See the PLOS Data Policy and FAQ for detailed information.	No - some restrictions will apply

A Data Availability Statement describing where the data can be found is required at submission. Your answers to this question constitute the Data Availability Statement and **will be published in the article**, if accepted.<br><br>**Important:** Stating 'data available on request from the author' is not sufficient. If your data are only available upon request, select 'No' for the first question and explain your exceptional situation in the text box.<br><br>Do the authors confirm that all data underlying the findings described in their manuscript are fully available without restriction?	
**Describe where the data may be found in full sentences. If you are copying our sample text, replace any instances of XXX with the appropriate details.**<br><br>• If the data are **held or will be held in a public repository**, include URLs, accession numbers or DOIs. If this information will only be available after acceptance, indicate this by ticking the box below. For example: *All XXX files are available from the XXX database (accession number(s) XXX, XXX.*).<br>• If the data are all contained **within the manuscript and/or Supporting Information files**, enter the following: *All relevant data are within the manuscript and its Supporting Information files.*<br>• If neither of these applies but you are able to provide **details of access elsewhere**, with or without limitations, please do so. For example:<br><br>*Data cannot be shared publicly because of [XXX]. Data are available from the XXX Institutional Data Access / Ethics Committee (contact via XXX) for researchers who meet the criteria for access to confidential data.*<br><br>*The data underlying the results presented in the study are available from (include the name of the third party*	Data used in this article, for showing the developed tool, are property of the Italian Ministry of Health and contain sensitive information that can not be shared for privacy constraints. Thus the article does not report data, and the data availability policy is not applicable to this article.

and contact information or URL).<br>• This text is appropriate if the data are owned by a third party and authors do not have permission to share the data.<br><br>* typeset	
Additional data availability information:	

Whole manuscript including figures

Click here to access/download;Manuscript;TRacing_CANDELORO_ET_AL.

Article

T-Racing: a modern tool for supporting epidemiological investigation in animal disease outbreaks

Luca Candeloro [1], Lara Savini [1*], Annamaria Conte [1], Paolo Calistri [1], Diana Palma [1], Samuel Perticara [1], Alessio di Lorenzo [1], Susanna Tora [1], Sara Serrani [1], Michela Toro [1] and Fabrizio De Massis [1]

[1] Istituto Zooprofilattico Sperimentale dell'Abruzzo e del Molise "G.Caporale", Italy
* Correspondence: l.savini@izs.it

Abstract: The One Health approach unites efforts across human-animal-environment interfaces against shared threats like zoonotic diseases. T-Racing, a modern web tool, helps epidemiological investigations and lets contain livestock-related disease spread, aligning with One Health principles to safeguard public health. The application uses Temporal Network Analysis techniques to account for the dynamic nature of animal trade and to perform quick and efficient back and forward tracing procedures. T-Racing leverages web services to retrieve data from multiple simultaneously and near real-time sources through the plumber package and has been distributed using Shinyproxy. Indeed, T-Racing manages and analyses extensive and diverse datasets, including animal movement data, livestock owners' and producers' information, disease outbreak data, and genomic data obtained from National databases within the same environment. In this work, we show T-Racing's capabilities by simulating epidemiological investigations of some Brucellosis and Tuberculosis outbreaks that occurred in non–endemic areas, in Italy. This tool greatly supports epidemiological investigations, making easier and faster data analysis and enabling a comprehensive understanding of disease transmission patterns facilitating prompt interventions.

Keywords: One Health; Epidemiology; Tracing; Temporal Network Analysis; Shiny web application; Outbreak investigation; Zoonoses management.

1. Introduction

Preventing and controlling communicable diseases in livestock, through outbreak early detection, increases the effectiveness of the emergency response and reduce the social, economic, and environmental costs associated with the outbreak [1, 2]. Moreover, animals serve as an excellent channel for monitoring both novel and known pathogens with outbreak potential, as more than 60% of emerging infectious diseases in humans originate from zoonotic sources [3-5]. This emphasizes the critical role of animal health in discovering, monitoring, and predicting environmental and human health hazards [6], in a One-health approach.

In this context, the movement of animals is often considered a major risk for disease spreading between herds [7], and to humans when zoonosis are involved. Consequently, livestock transport data is registered in national and international databases to enable contact tracing in the event of an outbreak [8-12]. Another official movements database is TRACES (TRAde Control and Expert System), an information platform of the European Union for the reporting, certification and control of imports of animals, products of animal origin, food and feed of plant origin and other products of veterinary interest into the EU, as well as for intra-EU trade and exports from the EU of animals and certain products of animal origin. Currently, TRACES is the integrated computerised veterinary system with a single architecture provided for in Decisions 2003/24/EC and 2004/292/EC (EU, 2019) [13,14].

Tracing the spread of diseases within animal populations, and tracing potential routes of exposure and spread is critical for effective diseases control [15], and is crucial in the final steps of an eradication programme.

Contact tracing plays a pivotal role in identifying the action to be taken to break the transmission chain, and to reduce the spread and duration of infectious diseases, thus preventing new cases [16]. When zoonoses are considered, this means also protecting public health.

Additionally, contact tracing has the subsequent effect of providing assessments to stakeholders useful for quickly identifying and implementing containment, surveillance, and control systems.

Animal health officials require to rapidly identify which animals are involved, where and when infected animals are located, and what other susceptible animals might have been exposed to the disease.

- Having tools in place to facilitate disease control and surveillance during critical outbreak stages can save time, help in preventing further spread, thereby minimizing the costs and consequences of the outbreak [17, 2].

Technology plays a pivotal role in enhancing the outcome of epidemiological investigations. Advanced analytics and link analysis may help in uncovering hidden patterns of disease spreading [18, 2].

Yet, contact tracing, although sounding straightforward, can be overwhelmingly complex and resource-intensive. However, efforts in contact-tracing can be significantly improved by using data visualization and analytics allowing public health officials to uncover insights from contact-tracing efforts and all available health data to understand:

- Missing or unexpected links in contact details.
- Identifying animals who should undergo testing.
- Tracking the geographical spread of the virus.
- Determining which farms are at the highest risk.

This could be an invaluable tool that allows veterinary services, epidemiologists, and competent authorities to respond quickly in identifying the "hot spots" and in implementing effective control and eradication measures.

In this article we present T-Racing, a user-friendly web application developed in an R environment to support epidemiological investigations through interacting maps, graphs, and tables.

2. Materials and Methods

T-Racing has been designed with the aim of providing an efficient and simple tool for analysing spatiotemporal pathways, accounting for the epidemiological useful datasets.

Along with animal movement data including livestock owners' and producers' information, disease outbreaks data, and also Genomic data, like Multi Loci Sequence Type (MLST) have been included in the application.

Animal movements and livestock owners and producers' data, outbreak disease data, and Genomic data are provided by the Italian:

1. National Database for Animal Registration (BDN, www.vetinfo.sanita.it),
2. National Animal Disease Notification System (SIMAN, www.vetinfo.sanita.it), and
3. National Reference Centre for Whole Genome Sequencing of microbial pathogens database and bioinformatics analysis' (GENPAT) [19, 20].

These systems are managed by Istituto Zooprofilattico Sperimentale dell'Abruzzo e del Molise "G. Caporale" (IZS-Teramo) on behalf of the Italian Ministry of Health.

To ensure data accuracy and consistency, rigorous data collection, processing, and control rules have been implemented for each of these systems. These rules are continuously updated and adapted to comply with national and European laws and regulations, reflecting a commitment to maintaining the accuracy, relevance, and reliability of the data.

The application relies on Temporal Network Analysis techniques and accounts for the dynamic nature of animal trade to facilitate rapid and efficient back and forward tracing processes.

T-Racing has been developed using the Shiny library by the R statistical environment, and leaflet and visnetwork libraries were used for map and graph visualization respectively (in Table 1 are listed all of the packages used). The application lets manage large amounts of data from different information systems and share them efficiently and securely in real-time.

Indeed, computationally heavy services are executed on a remote server, differently from the serverless web apps, and the Plumber package let the application query data sources in real-time, through web API.

The system architecture (Figure 1) makes sure the application scalability, thanks to shiny proxy (a free and open-source Docker-based application).

The application makes use of an interactive graph, map, and tables to improve the user experience. The control bar, displayed in the center of the dashboard, provides different tools to:

- Filter data based on multi-species selection, period interval, tracing direction (back or forward), and include or exclude animal trade data from and to foreign countries or slaughterhouses;
- Implement multi-seed selection, including those within a spatial buffer around a farm or outbreak. Moreover, T-Racing offers two methods for selecting the seeds for tracing: either by choosing one or more farm codes from the list of farms or by selecting an outbreak code from the list of officially notified outbreaks;
- Customize the graph visualization by choosing a layout option: hierarchical, geographical, or nicely mode. Additionally, the operator can thematise edge/nodes by selecting attributes of data such as facility type or production orientation for nodes, and time, species, or depth level for edges;
- Remove unnecessary nodes or edges from performed tracing;
- Simplify the resulting network by keeping only temporally valid paths among outbreaks and/or those involving individually identified animals;
- Add sequenced pathogens for the animals or outbreaks data engaged in a specific disease epidemiological investigation;
- View spatial-temporal paths of individual animals by querying their identity code;
- Store the results in a dynamic HTML report for offline use.

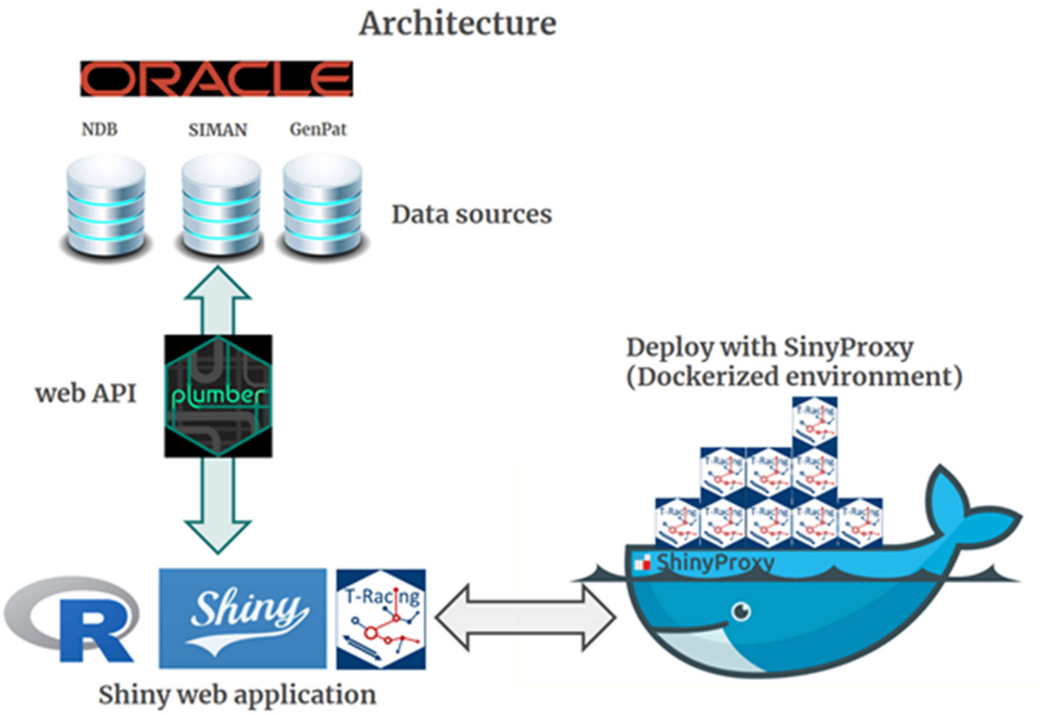

Figure 1. T-Racing architecture: T-Racing makes use of web services to retrieve data from databases, through the plumber package, and has been distributed using Shinyproxy.

Table 1. List of the R-packages used in T-Racing developing.

R-package	Use	[illegible]rence
shiny	UI/SERVER	Winston Chang, Joe Cheng, JJ Allaire, Carson Sievert, Barret Schloerke, Yihui Xie, Jeff Allen, Jonathan McPherson, Alan Dipert and Barbara Borges (2021)
shinyjs	UI/SERVER	Dean Attali (2020). shinyjs: Easily Improve the User Experience of Your Shiny Apps in Seconds
shinyWidgets	UI	Victor Perrier, Fanny Meyer and David Granjon (2021). shinyWidgets: Custom Inputs Widgets for Shiny
shinydashboard	UI	Winston Chang and Barbara Borges Ribeiro (2018). shinydashboard: Create Dashboards with 'Shiny'
shinyBS	UI	Eric Bailey (2015). shinyBS: Twitter Bootstrap Components for Shiny
shinybusy	UI/SERVER	Fanny Meyer and Victor Perrier (2020). shinybusy: Busy Indicator for 'Shiny' Applications
DT	UI	Yihui Xie, Joe Cheng and Xianying Tan (2021). DT: A Wrapper of the JavaScript Library 'DataTables'
rintrojs	UI/SERVER	Carl Ganz (2016). rintrojs: A Wrapper for the Intro.js Library. Journal of Open Source Software, 1(6), October 2016
shinycssloaders	UI/SERVER	Andras Sali and Dean Attali (2020). shinycssloaders: Add Loading Animations to a 'shiny' Output While It's Recalculating
htmltools	UI	Joe Cheng, Carson Sievert, Barret Schloerke, Winston Chang, Yihui Xie and Jeff Allen (2021). htmltools: Tools for HTML
leaflet	UI	Joe Cheng, Bhaskar Karambelkar and Yihui Xie (2022). leaflet: Create Interactive Web Maps with the JavaScript 'Leaflet' Library
rgdal	SERVER	Roger Bivand, Tim Keitt and Barry Rowlingson (2021). rgdal: Bindings for the 'Geospatial' Data Abstraction Library
sp	SERVER	Pebesma, E.J., R.S. Bivand, 2005. Classes and methods for spatial data in R. R News 5 (2)
bezier	SERVER	Aaron Olsen (2018). bezier: Toolkit for Bezier Curves and Splines
geosphere	SERVER	Robert J. Hijmans (2021). geosphere: Spherical Trigonometry
visNetwork	UI	Almende B.V., Benoit Thieurmel and Titouan Robert (2019). visNetwork: Network Visualization using 'vis.js' Library
igraph	SERVER	Csardi G, Nepusz T: The igraph software package for complex network research, InterJournal, Complex Systems 1695. 2006
ggplot2	SERVER	H. Wickham. ggplot2: Elegant Graphics for Data Analysis. Springer-Verlag New York, 2016
RColorBrewer	SERVER	Erich Neuwirth (2014). RColorBrewer: ColorBrewer Palettes
xlsx	SERVER	Adrian Dragulescu and Cole Arendt (2020). xlsx: Read, Write, Format Excel 2007 and Excel 97/2000/XP/2003 Files
dplyr	SERVER	Hadley Wickham, Romain François, Lionel Henry and Kirill Müller (2021). dplyr: A Grammar of Data Manipulation
jsonlite	SERVER	Jeroen Ooms (2014). The jsonlite Package: A Practical and Consistent Mapping Between JSON Data and R Objects. arXiv:1403.2805

3. Results

T-Racing is currently available as a demo within IZS-Teramo. Once the testing phase is completed, it will be made available to healthcare professionals, epidemiologists, and relevant authorities. In this way, it will become an operational tool supporting field epidemiological investigations in Italy.

Traceability in terms of trace-back and trace-forward can be used to detect pathways of introduction and spread of pathogens transmitted by animal-to-animal contacts. Figure 2 shows the T-Racing interface by displaying the network related to bovine movements on a map and graph resulting from investigating temporally valid paths starting from a Brucellosis outbreak in Sicily (an Italian southern island). The simultaneous visualization of the graph and map provides a comprehensive and user-friendly exploratory experi-

ence. Node's shape represents the type of facilities (pastures being squares, holdings circles, and assembly or rest points ellipses), arrows represent movements (the width is proportional to the number of moved animals, and the red colour highlights paths followed by animals, singularly identified, had gone out of the seed). The fire icons on the map represent the Brucellosis outbreaks reached by the seed farm (red contoured circle).

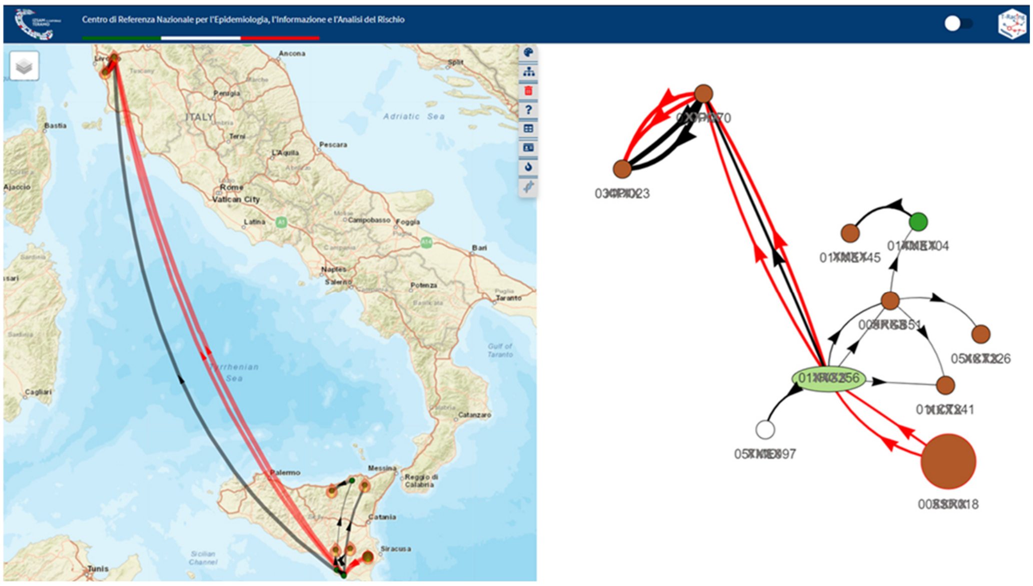

Figure 2. The T-Racing interface shows the network (map and graph) resulting from investigating temporally valid paths starting from a Brucellosis outbreak in Sicily Region, Italy. Node's shape represents the type of facilities (pastures being squares, holdings circles, and assembly or rest points ellipses), arrows represent movements (the width is proportional to the number of animals moved, and the red colour highlights paths followed by animals, singularly identified, had gone out of the seed). The fire icons on the map represent the Brucellosis outbreaks reached by the seed farm (red contoured circle).

3.1. T-Racing: Simulation Exercise

We demonstrated the use of T-Racing for a Brucellosis and Tuberculosis outbreaks that occurred in non–endemic areas, in Italy.

3.1.1. Case study: Tracing back from a notified outbreak of Brucellosis in the Marche region, central Italy, in 2019, to identify its potential sources of infection.

Brucellosis caused by *Brucella abortus* and tuberculosis caused by *Mycobacterium bovis* are important zoonosis that constitute a serious hazard to public health. Prevention of these zoonoses depends on the control of the disease in animals. Livestock movement data represent a valuable source of information to understand the pattern of contacts between holdings, which may determine the spread of such diseases. The first national plans for the control of bovine tuberculosis and brucellosis were approved with the Ministerial Decree of 11 and 12 March 1965, respectively. Through the DM 27 August 1994 n. 651 entered into force in Italy the national eradication plan for bovine brucellosis, which establishes the measures to be applied to cattle farms in order to obtain the eradication of the disease for the protection of public health and protection of officially free herds. The regulation provides for control official serological test twice a year at an interval of not less than three and not more than six months, in all cattle farms, even if in the wild, of all animals over the age of twelve months. Similar procedures have been established with Ministerial Decree 15 December 1995, n. 592 – Regulation concerning the national plan for the eradication of tuberculosis in cattle and buffalo herds – by testing with the tuberculin intradermal test.

At the end of 2019 (2019-12-09), a brucellosis outbreak was confirmed in central Italy, specifically in the Marche region, which was free from the disease. The T-Racing application was utilized to identify the possible source of infection, focusing on animal trade. For tracing purposes, only bovine species were selected, excluding slaughterhouses and foreign countries as sources. A backward search was conducted, considering a broad time frame encompassing all incoming movements to the identified outbreak over the past two years. In this case study, the seed was selected using one of the methods provided by the application, namely by selecting the known outbreak code from a list of proposed brucellosis outbreaks for the study period. The search for the subnetwork resulted in 1.826 movements involving 386 nodes. The subnetwork covers a substantial area of central Italy, including establishments that reach the seed even from the islands of Sicily and Sardinia. The theming of nodes based on type reveals the involvement of different facility types. In addition to breeding farms, there are pastures (structures that play a significant role in the spread of brucellosis) and a hub node, a genetic collection center located in Perugia province, Umbria region, in central Italy and connecting 132 nodes (Figure 3). To make the network more manageable and relevant for the conducted tracing, a subset was created by setting 3 levels and reducing the depth to 399 days, resulting in 199 movements and 38 nodes. To identify plausible paths of disease spread, the application allows the utilization of notified outbreaks within a customizable time period for the specific disease. These outbreaks are visualized on the map, enabling the tracing to be narrowed down to the connections between outbreaks included in the tracing. This helps identify a focused subset of connections to investigate. The network includes multiple Brucellosis outbreaks, primarily located in the southern and central regions. The reduced network, specifically focusing on Brucellosis outbreaks within the network, consists of 103 movements involving 14 nodes (Figure. 4).

At this point, we can assess the presence of genetic information as further confirmation of the tracing's plausibility. To do so, we utilize the tool by adding the available sequence type for Brucella abortus. The seed is an outbreak for which sequence type "2" is available, and the identified origin zones are also affected by clusters of the same sequence type.

An interesting aspect is to determine how many of these links involve the same animals that reached the seed. The dedicated tool allows for theming the links based on this characteristic by conducting a search for individually identified animals. The visualization highlights that only a small part of the subnetwork involves the same animals that reach the seed. The final result consists of only 21 movements involving 5 nodes (including the seed and a pasture, which is the presumed source of infection). Further investigation of individually identified and traced animals revealed that an infected animal, identified as sequence type "2" in the seed, originated from a pasture, which was subsequently identified as the plausible source of the sought-after infection (Figure. 5).

The presented tracing example has highlighted a plausible path of disease diffusion and the presumed origin. Starting from a relatively large and heterogeneous network (1.826x386) in terms of both spatial and temporal dimensions, it was possible to focus attention on a result of 21 movements and 5 nodes in just a few steps, utilizing interactive and iterative use of complementary information, such as disease outbreaks, genetic information, and movements of individually identified animals.

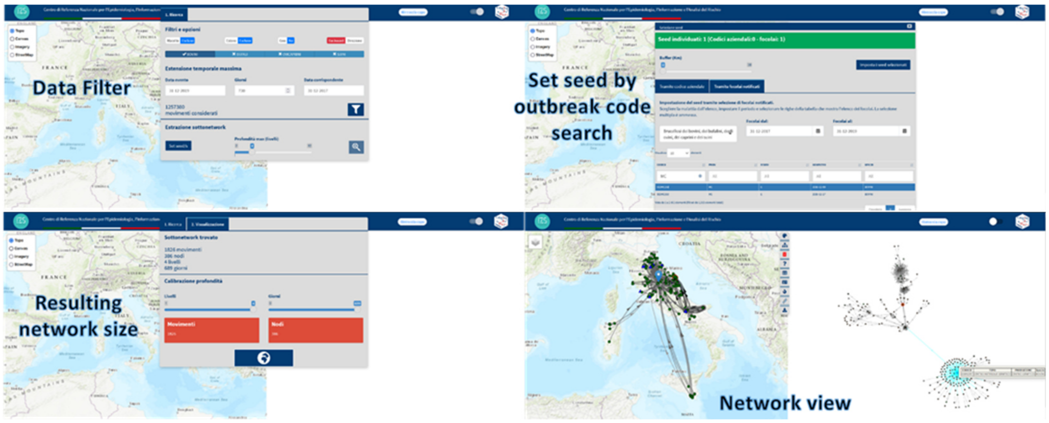

Figure 3. Trace-back simulation. Sequential depiction of four steps: 1) Data Filter, 2) Set seed by outbreak code search, 3) Resulting network size, and 4) Network view. The trace-back focusing on bovine species, excluding slaughterhouses and foreign countries, was conducted to trace the outbreak occurring in December in the Marche region, in Italy. 1,826 movements involving 386 nodes were identified within a broad time spanning the past two years. The seed is contoured in red. The network covers a substantial area of central Italy, including farms extending to the islands. Node theming based on structure type highlights diverse farm types, including pastures and a genetic collection center located in Perugia and highlighted in light blue in the figure.

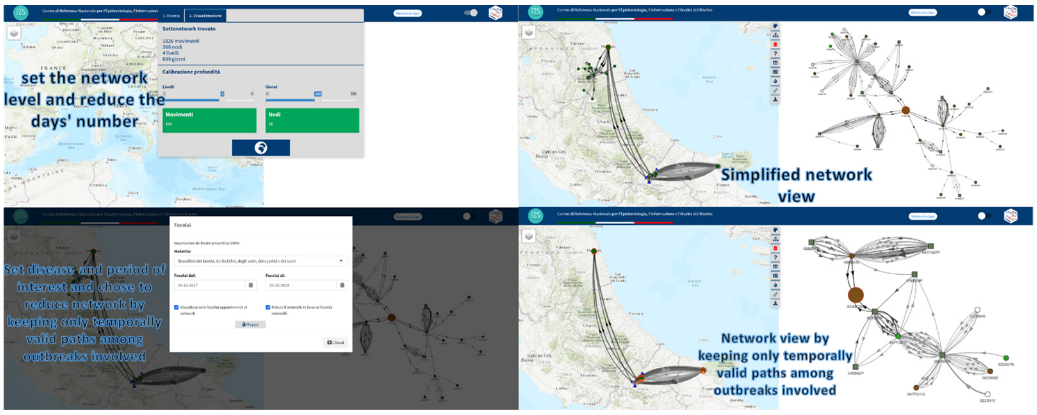

Figure 4. A large network is often impractical and less informative for the tracing process. T-Racing allows for reducing the network using diverse tools. The figure shows a possible sequential of four steps: 1) Network reduction by setting the network level and reducing days' number (the new network size is 199 links and 38 nodes), 2) Reduced network view, 3) Reducing network by setting outbreaks for a specific disease and period of interest 4) Resulting network by keeping only temporally valid paths among outbreaks involved (the network size is 103 links and 14 nodes and the three Brucellosis' outbreaks involved are shown on the map with the red fire icons).

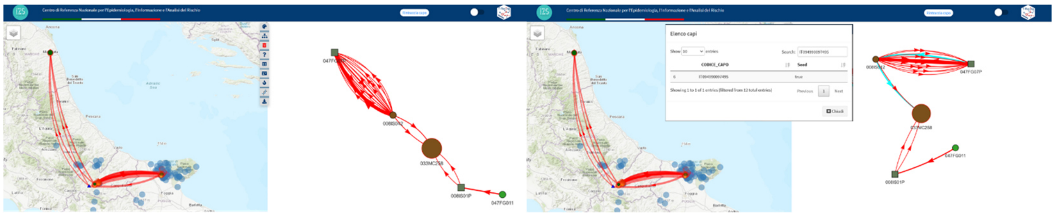

Figure 5. In the map and graph on the left, all the links involving the same animals that reached the seed are highlighted in red. Furthermore, the distribution of the sequences data available for Brucella abortus has been added to the map (type 2 in blue). A specific tool (in the menu bar) allows you to extrapolate the subnet of the highlighted links only and the final result consists of only 21 movements involving 5 nodes (map and graph on the right). Further investigation on the animals

individually identified and found by querying the links, highlighted in light blue on the graph, revealed that an infected animal originating from the pasture (at the top right of the graph) has reached the seed.

3.1.2. Case study: In Italy, Bovine Tuberculosis (BTB) is endemic in the southern region. However, sporadic outbreaks occur in non-endemic area. Two interesting example of how this application can help in controlling the spread of the disease or in tracing the source of the infection, are presented hereafter: The Elba island outbreak and the Cuneo province in Piedmont region (northern Italy) outbreak.

Four BTB outbreaks were notified during June 2023 in a small, non-endemic area, in Italy, affecting Elba island and the near Tuscany coast. Using T-Racing allowed the detection of the real flow of the disease spread. Indeed, a forward tracing starting from the outbreak detected in Elba island highlighted an animal movement (of nine cattle) during April 2023 reaching the farms on the nearby coast and let to establish the real source of the infection and the most likely flow of the spread (Figure 6.). Interestingly, the movements reached four farms in the Tuscany coast (three of which, were outbreaks). Using a specific tool to query the individually identified animals, we discovered the animals reaching the outbreaks were the same animals leaving Elba island and some of them (at the time of writing this article) had not yet been slaughtered.

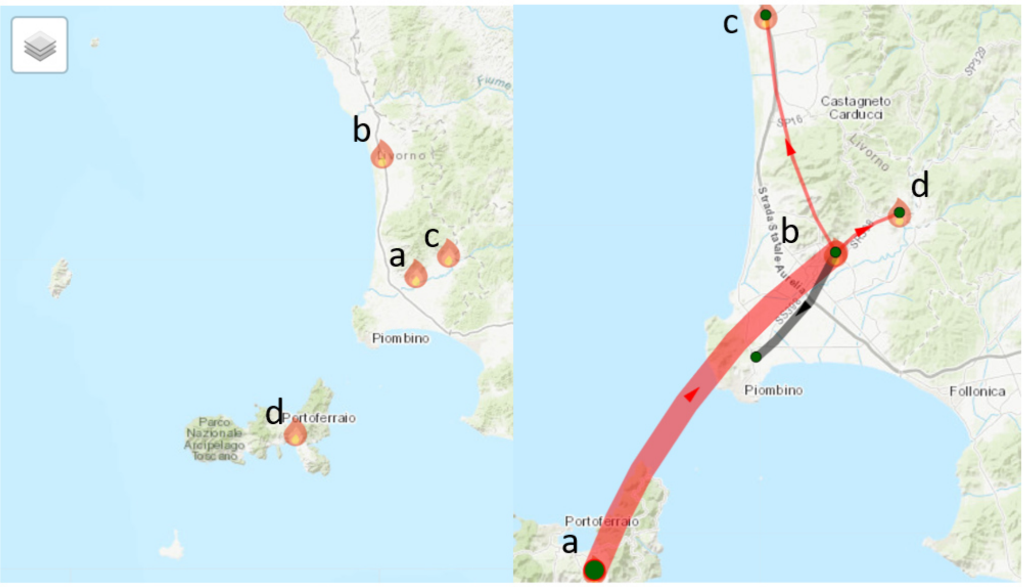

Figure 6. Trace –forward simulation. Left: Outbreaks that occurred in the study area, arranged in the order of their notification timing (with 'a' representing the earliest and 'd' the most recent). Right: Network of animal movements involving the outbreaks (red links indicate the presence of at least one animal originating from the seeding site).

BTB outbreaks in northern Italy are considered uncommon. In December 2022, an outbreak of BTB occurred in the Cuneo province of the Piedmont region. To determine the most likely source of infection, we employed T-Racing, utilizing a wide temporal window of one year to track incoming movements. The resulting network encompassed 353 farms, with approximately six hundred connections, predominantly located in the Sicily region. To facilitate a more focused investigation, we employed a tool that retained only paths connecting outbreaks, resulting in a simplified network of 72 connections involving nine farms in Sicily region, three of which were BTB outbreaks. The investigation thus suggested three outbreaks occurred during the previous months as the most likely source of infection. Furthermore, the animals reaching the Cuneo province came from a fattening farm where they were probably infected by mixing with animals from the outbreaks. (Figure 7). In the context of the National BTB eradication programme, the primary screening

test for BTB in cattle in Italy is the single intradermal comparative tuberculin test. The specificity of this test is very high, approximately 99.98% at standard interpretation. This means that you would expect approximately one false positive result for every 5,000 uninfected cattle tested using standard interpretation (De la Rua-Domenech et al., 2006). However, the main limitation of the tuberculin skin test is its sensitivity. On average 20-25% of BTB-infected cattle can be missed by one round of skin testing using standard interpretation, thus reducing the efficacy of the surveillance programme, especially in areas where the disease is still circulating. To increase the level of sensitivity of the surveillance system, the Ministry of health has recently introduce the use of γ-interferon test for animals from free herds located in non-free areas and moved onto free areas.

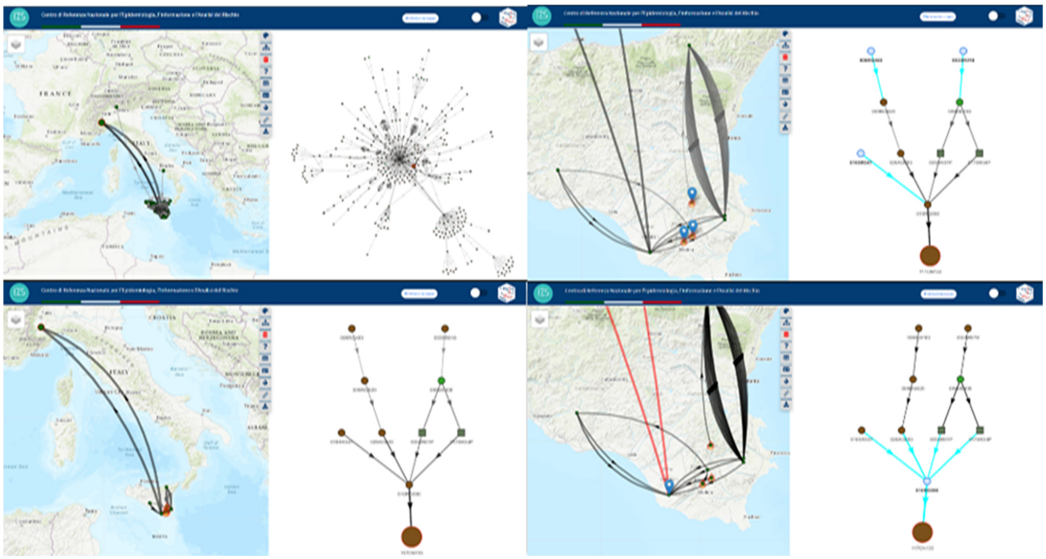

Figure 7. Trace-back simulation. The network resulting from the trace-back from the Cuneo BTB outbreak involved 353 farms (with about six hundred links), mostly located in the Sicily region (left-up). To simplify this network and make it possible to investigate deeply, we used the tool letting to retain only paths among outbreaks. The simplified network (left-down) resulted in 72 links involving 10 farms (3 being BTB outbreaks). Details on the network involving 3 outbreaks in the Sicily region and their outgoing movements are light blue highlighted on the right-up side. The links involving the farm where the mixing of infected animals may have occurred are shown in the right-down panel, along with the links involving animals reaching the Cuneo outbreak (red arcs).

4. Discussion

Preventing and controlling communicable diseases in livestock, through outbreak early detection, increases the effectiveness of the emergency response and reduce the social, economic, and environmental costs associated with the outbreak [1, 2]. Timely identification of outbreaks and systematic contact tracing can be effective in stopping the transmission chain of highly contagious diseases, provided there is strong collaboration between epidemiological teams, laboratory surveillance, and outbreak management, in a One-Health context [21].

One Health is the collaborative efforts of multiple disciplines to attain optimal health for people, animals and the environment, a concept that historically owes much to the study of brucellosis, including recently political and ethical considerations. Brucellosis One Health actors include Public Health and Veterinary Services, microbiologists, medical and veterinary practitioners and breeders. Brucellosis awareness, and correct use of diagnostic, epidemiological and prophylactic tools is essential [22]. Thus, racing the spread of diseases within animal populations, and tracing potential routes of exposure and spread is critical for effective diseases control [15] and a further important actor of the One-Health concept. Contact tracing plays a pivotal role in breaking the transmission

chain, reducing the spread and duration of infectious diseases and preventing new cases [16].

Animal movements (from an origin to a destination farm) represent the major source of infection between animal populations, especially in the case of direct-contact diseases, so should be always explored as a path for disease spreading. Indeed, the actions following outbreak detections, although well-timed, can avoid only a part of the potential disease diffusion pathways that tracing on animal movements can detect (Figure 8).

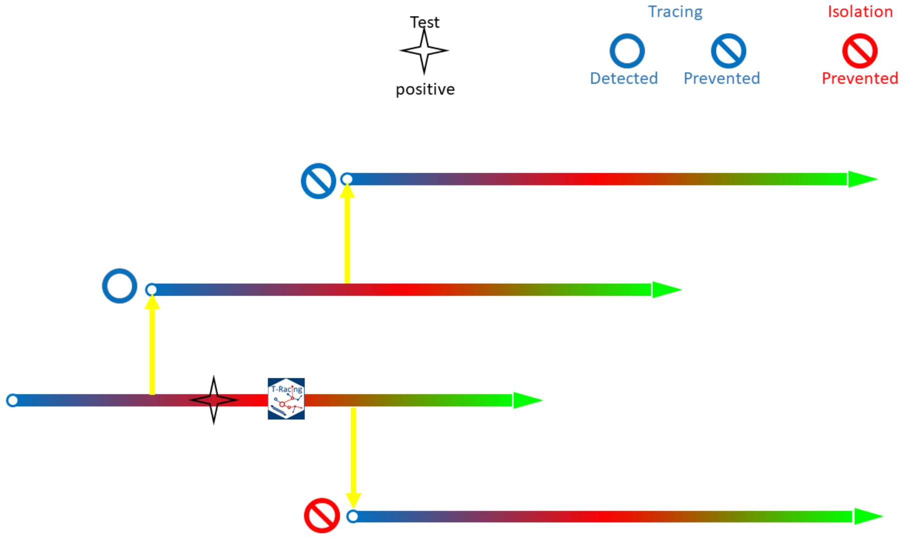

Figure 8. Illustration of the importance of tracing in preventing and detecting diffusion path-ways versus the simple farm isolation when only positivity recorded in the context of the surveillance programme is considered.

So having data about traded animals may seem enough for an effective approach. However, a simple and raw query on traded animal data may result in a huge network, sometimes confusing or completely useless. Then, a tool able to manage huge datasets and to add useful information is needed to understand where the disease most likely came from, or could have gone.

A good scalability of the system would enable the easy incorporation of additional functionalities and information, such as test and inspection histories, as well as disease status of the establishments, in the light of an enhanced disease control programs.

An important feature of the modern response to epidemics is the increasing focus on exploiting all available data to inform the response in nearly real-time and allow the data evidence-based decision-making [23].

This tool has the potential to significantly enhance animal disease control measures, as it can rapidly organize crucial contact information from extensive datasets and identify connections in multi-area outbreak investigations, thus efficiently supporting epidemiological investigations.

Lastly, in an era of rapid technological advancements, it is essential to foster innovation and develop tools that revolutionize epidemiological investigations. By combining cutting-edge technology with comprehensive data analysis, T-Racing opens new possibilities for effective and efficient disease control in animal disease outbreaks.

The presented simulation exercises highlighted the most plausible path of disease diffusion and the presumed origin/source of the infection in just a few steps, utilizing interactive and iterative use of complementary information, such as disease outbreaks, genetic information, and movements of individually identified animals.

Building trust should be one critical goal of One Health programs; thus, it should exist at all levels, including between the Veterinary and Human Health systems [22].

With its user-friendly interface and interactive features, T-Racing empowers clinicians, veterinarians, ecosystem health professionals, researchers, and policymakers to leverage the power of animal surveillance within the One Health perspective [3, 24]. By embracing this innovative tool, stakeholders can gain valuable insights, make informed decisions, and take proactive measures to mitigate the impact of infectious diseases on animal and human health.

This dynamic approach enables the T-Racing application to provide accurate and timely data for epidemiological investigations, emphasizing its data-driven nature. It recognizes the crucial role of accurate data in conducting effective investigations and highlights the need for robust data management practices. Without reliable data, the utility and reliability of the T-Racing application would be compromised.

5. Conclusions

T-Racing represents a modern and forward-thinking approach to supporting epidemiological investigations in animal disease outbreaks. By integrating advanced technology, comprehensive data management, and cutting-edge analytics, it provides a unified environment to manage and analyse the expanding pool of data sources. With its capability to offer meaningful insights and facilitate evidence-based decision-making, T-Racing stands at the forefront of disease control, strengthening our ability to address emerging challenges and protect global health, in a One-Health perspective.

Author Contributions: Conceptualization, L.C., L.S., A.C., P.C. and F.D.M.; methodology, L.C. and L.S.; software, L.C. and S.P.; validation, F.D.M. and P.C.; data curation, D.P.; writing—original draft preparation, L.S., L.C. and F.D.M.; writing—review and editing, L.C., L.S., A.C., F.D.M., P.C., S.P., A.D.L., D.P., S.T., S.S., and M.T.; project administration, P.C.; funding acquisition, P.C.. All authors have read and agreed to the published version of the manuscript.

Funding: This research was funded by Italian Ministry of Health (RC MSRCTE0219).

Conflicts of Interest: The authors declare no conflict of interest.

References

1. Schirdewahn, F., K. Lentz, H. H., Colizza, V., Koher, A., Hövel, P., & Vidondo, B. Early warning of infectious disease outbreaks on cattle-transport networks. PLOS ONE, 16(1), e0244999. https://doi.org/10.1371/journal.pone.0244999
2. Maria Nöremark and Stefan Widgren. EpiContactTrace: an R-package for contact tracing during livestock disease outbreaks and for risk-based surveillance. BMC Veterinary Research 2014, 10:71 Page 2 of 9 http://www.biomedcentral.com/1746-6148/10/71
3. Prata, J. C., Ribeiro, A. I., & Rocha-Santos, T. (2022). An introduction to the concept of One Health. One Health, 1-31. https://doi.org/10.1016/B978-0-12-822794-7.00004-6
4. Wondwossen A. Gebreyes, Daral Jackwood, Celso Jose Bruno de Oliveira, Chang-Won Lee, Armando E. Hoet, Siddhartha Thakur. Molecular Epidemiology of Infectious Zoonotic and Livestock Diseases. 2020. Microbiology Spectrum, 10.1128/microbiolspec.ame-0011-2019, 8, 2, https://journals.asm.org/doi/abs/10.1128/microbiolspec.ame-0011-2019
5. Neo, J. P. S., & Tan, B. H. (2017). The use of animals as a surveillance tool for monitoring environmental health hazards, human health hazards and bioterrorism. Veterinary Microbiology, 203, 40-48. https://doi.org/10.1016/j.vetmic.2017.02.007
6. D Kriebel, J Tickner, P Epstein, J Lemons, R Levins, E L Loechler, M Quinn, R Rudel, T Schettler, A M Stoto. The precautionary principle in environmental science. 2001, Environmental Health Perspectives, 871-876, 109, 9, https://ehp.niehs.nih.gov/doi/abs/10.1289/ehp.01109871
7. Ansari, S., Heitzig, J., Brzoska, L., Lentz, H. H., Mihatsch, J., Fritzemeier, J., & Moosavi, M. R. (2021). A Temporal Network Model for Livestock Trade Systems. Frontiers in Veterinary Science, 8. https://doi.org/10.3389/fvets.2021.766547
8. Loria GR, Migliore S, Bongiorno C, Ciaccio G and Laddomada A (2022) New European rules introduced by regulation (EU) 2016/429 to facilitate animal trade: With great risk comes great responsibility. Front. Vet. Sci. 9:1003732. https://doi.org/10.3389/fvets.2022.1003732
9. Porphyre, T., Boden, L. A., Correia-Gomes, C., Auty, H. K., Gunn, G. J., & J. Woolhouse, M. E. (2016). Using national movement databases to help inform responses to swine disease outbreaks in Scotland: The impact of uncertainty around incursion time. Scientific Reports, 6. https://doi.org/10.1038/srep20258
10. Regulation (EU) 2016/429 of the European Parliament and of the Council of 9 March 2016 on transmissible animal diseases and amending and repealing certain acts in the area of animal health ('Animal Health Law') (Text with EEA relevance). Off J Eur Communities. (2016) L84:1–208
11. ORDINANZA 9 agosto 2012 Misure straordinarie di polizia veterinaria in materia di tubercolosi, brucellosi bovina e bufalina, brucellosi ovi-caprina, leucosi, nelle regioni Calabria, Campania, Puglia e Sicilia. (12A09729) (GU Serie Generale n.212 del 11-09-2012) https://www.gazzettaufficiale.it/eli/id/2012/09/11/12A09729/sg (visitato il 25/08/2023)
12. ORDINANZA 28 maggio 2015, Misure straordinarie di polizia veterinaria in materia di tubercolosi, brucellosi bovina e bufalina, brucellosi ovi-caprina, leucosi bovina enzootica. (15A04879) (GU n.144 del 24-6-2015) and following amendments and additions
13. 2003/24/EC: Commission Decision of 30 December 2002 concerning the development of an integrated computerised veterinary system (notified under document number C(2002) 5496)
14. EU, 2019. Commission Implementing Regulation (EU) 2019/1715 of 30 September 2019 laying down rules for the functioning of the information management system for official controls and its system components (the IMSOC Regulation) OJ L 261, 14.10.2019
15. Mardones, F., zu Donha, H., Thunes, C., Velez, V., & Carpenter, T. (2013). The value of animal movement tracing: A case study simulating the spread and control of foot-and-mouth disease in California. Preventive Veterinary Medicine, 110(2), 133-138. https://doi.org/10.1016/j.prevetmed.2012.11.024
16. Judite Monteiro Braga et al. Investigation and contact tracing of the first cases of COVID-19 in Mozambique, 2020. Pan African Medical Journal. 2022;41:302. https://doi.org/10.11604/pamj.2022.41.302.26979
17. Bingham, P., Wada, M., van Andel, M., McFadden, A., Sanson, R., & Stevenson, M. (2020). Real-Time Standard Analysis of Disease Investigation (SADI)—A Toolbox Approach to Inform Disease Outbreak Response. Frontiers in Veterinary Science, 7, 563140. https://doi.org/10.3389/fvets.2020.563140
18. Kölzsch, A., Davidson, S.C., Gauggel, D. et al. MoveApps: a serverless no-code analysis platform for animal tracking data. Mov Ecol 10, 30 (2022). https://doi.org/10.1186/s40462-022-00327-4
19. Decreto 30 maggio 2017. Istituzione di un Centro di referenza nazionale nel settore veterinario. G.U. n 196 del 26 Agosto, 2017
20. Di Lorenzo A, Mangone I, Colangeli P, Cioci D, Curini V, Vincifori G, Mercante MT, Di Pasquale A, Iannetti S. One health system supporting surveillance during COVID-19 epidemic in Abruzzo region, southern Italy. One Health. 2023 Jun;16:100471. https://doi.org/10.1016/j.onehlt.2022.100471. Epub 2022 Dec 7. PMID: 36507072; PMCID: PMC9726647.
21. Tegegne, H. A., Bogaardt, C., Collineau, L., Cazeau, G., Lailler, R., Reinhardt, J., Freeth, F. T., Taylor, E., Prada, J. M., & Hénaux, V. (2023). OH-EpiCap: A semi-quantitative tool for the evaluation of One Health epidemiological surveillance capacities and capabilities. Frontiers in Public Health, 11, 1053986. https://doi.org/10.3389/fpubh.2023.1053986
22. Moriyón, I., Blasco, J. M., Letesson, J. J., De Massis, F., & Moreno, E. Brucellosis and One Health: Inherited and Future Challenges. Microorganisms, 11(8), 2070. https://doi.org/10.3390/microorganisms11082070

23. Jonathan A. Polonsky, Amrish Baidjoe, Zhian N. Kamvar, Anne Cori, Kara Durski, W. John Edmunds, Rosalind M. Eggo, Sebastian Funk, Laurent Kaiser, Patrick Keating, Olivier le Polain de Waroux, Michael Marks, Paula Moraga, Oliver Morgan, Pierre Nouvellet, Ruwan Ratnayake, Chrissy H. Roberts, Jimmy Whitworth and Thibaut Jombart. Outbreak analytics: a developing data science for informing the response to emerging pathogens. Philosophical Transactions of the Royal Society B: Biological Sciences, Volume 374, Issue 1776. https://doi.org/10.1098/rstb.2018.0276
24. Charlier, J., Barkema, H. W., Becher, P., De Benedictis, P., Hansson, I., Hennig-Pauka, I., La Ragione, R., Larsen, L. E., Madoroba, E., Maes, D., Marín, C. M., Mutinelli, F., Nisbet, A. J., Podgórska, K., Vercruysse, J., Vitale, F., Williams, D. J. L., & Zadoks, R. N. (2022). Disease control tools to secure animal and public health in a densely populated world. The Lancet Planetary Health, 6(10), e812-e824. https://doi.org/10.1016/S2542-5196(22)00147-4
