# Supplementary material for: T-Racing: a modern tool for supporting epidemiological investigation in animal disease outbreaks in Italy
Source: PLoS One. 2025 Feb 19;20(2):e0317313. doi: 10.1371/journal.pone.0317313 (PMC11838889; doi:10.1371/journal.pone.0317313)
Supplement: Attachment — Submitted filename: PONE-D-24-01354_R1_reviewer_ea_2024_07_25.pdf [file pone.0317313.s003.pdf]

PLOS ONE

T-Racing: a modern tool for supporting epidemiological investigation in animal disease outbreaks

--Manuscript Draft--

**Manuscript Number:**	PONE-D-24-01354R1
**Article Type:**	Research Article
**Full Title:**	T-Racing: a modern tool for supporting epidemiological investigation in animal disease outbreaks
**Short Title:**	T-Racing epidemiological web application
**Corresponding Author:**	Luca Candeloro, M.D.<br>Istituto Zooprofilattico Sperimentale dell'Abruzzo e del Molise G Caporale<br>Teramo, ITALY
**Keywords:**	One Health; Epidemiology; Tracing; temporal network analysis; Shiny web application; Outbreak investigation; Zoonoses management.
**Abstract:**	The One Health approach unites efforts across human-animal-environment interfaces against shared threats like zoonotic diseases. T-Racing is a Shiny web application, that supports epidemiological investigations and helps contain livestock-related disease spread, aligning with One Health principles of safeguarding public health. The application uses Temporal Network Analysis techniques to address the dynamic nature of animal trade, facilitating backward and forward tracing strategies. Therefore, T-Racing leverages web services to retrieve data from multiple sources simultaneously and almost in real-time through the plumber package and is distributed using Shinyproxy. Indeed, T-Racing manages and analyze extensive and diverse datasets within the same environment, including animal movement data, disease outbreak data, and genomic data, all obtained from Italian National databases. In this work, we show T-Racing's capabilities by simulating epidemiological investigations of brucellosis and tuberculosis outbreaks that occurred in non–endemic areas of Italy. This tool supports epidemiological investigations by adopting a data-driven approach, guiding users through the analysis via an iterative process while leveraging their expertise. Therefore, it enables faster data analysis, improves understanding of disease transmission patterns, and facilitates prompt and targeted interventions.
**Order of Authors:**	Luca Candeloro, M.D.
	Lara Savini
	Annamaria Conte
	Paolo Calistri
	Diana Palma
	Samuel Perticara
	Anna Cecilia Trolesi Reis Borges Costa, PhD
	Alessio Di Lorenzo
	Susanna Tora
	Sara Serrani
	Michela Toro
	Karina Diniz Baumgarten, PhD
	Fabrizio De Massis
**Response to Reviewers:**	We attached the doc file with answers to reviewers
**Additional Information:**	
**Question**	**Response**

Powered by Editorial Manager® and ProduXion Manager® from Aries Systems Corporation

**Financial Disclosure**<br><br>Enter a financial disclosure statement that describes the sources of funding for the work included in this submission. Review the submission guidelines for detailed requirements. View published research articles from *PLOS ONE* for specific examples.<br><br>This statement is required for submission and **will appear in the published article** if the submission is accepted. Please make sure it is accurate.<br><br>**Funded studies**<br>Enter a statement with the following details:<br>• Initials of the authors who received each award<br>• Grant numbers awarded to each author<br>• The full name of each funder<br>• URL of each funder website<br>• Did the sponsors or funders play any role in the study design, data collection and analysis, decision to publish, or preparation of the manuscript?<br><br>Did you receive funding for this work?	Yes
Please add funding details.<br>as follow-up to "**Financial Disclosure**<br><br>Enter a financial disclosure statement that describes the sources of funding for the work included in this submission. Review the submission guidelines for detailed requirements. View published research articles from *PLOS ONE* for specific examples.<br><br>This statement is required for submission and **will appear in the published article** if the submission is accepted. Please make sure it is accurate.	This research was funded by Italian Ministry of Health (MSRCTE0219).

**Funded studies**<br>Enter a statement with the following details:<br>• Initials of the authors who received each award<br>• Grant numbers awarded to each author<br>• The full name of each funder<br>• URL of each funder website<br>• Did the sponsors or funders play any role in the study design, data collection and analysis, decision to publish, or preparation of the manuscript?<br><br>Did you receive funding for this work?"	
Please select the country of your main research funder (please select carefully as in some cases this is used in fee calculation).<br>as follow-up to "**Financial Disclosure**<br><br>Enter a financial disclosure statement that describes the sources of funding for the work included in this submission. Review the submission guidelines for detailed requirements. View published research articles from *PLOS ONE* for specific examples.<br><br>This statement is required for submission and **will appear in the published article** if the submission is accepted. Please make sure it is accurate.<br><br>**Funded studies**<br>Enter a statement with the following details:<br>• Initials of the authors who received each award<br>• Grant numbers awarded to each author<br>• The full name of each funder<br>• URL of each funder website<br>• Did the sponsors or funders play any role in the study design, data collection and analysis, decision to publish, or preparation of the manuscript?<br><br>Did you receive funding for this work?"	ITALY - IT
**Competing Interests**	The authors have declared that no competing interests exist

Powered by Editorial Manager® and ProduXion Manager® from Aries Systems Corporation

Use the instructions below to enter a competing interest statement for this submission. On behalf of all authors, disclose any competing interests that could be perceived to bias this work—acknowledging all financial support and any other relevant financial or non-financial competing interests.<br><br>This statement is required for submission and **will appear in the published article** if the submission is accepted. Please make sure it is accurate and that any funding sources listed in your Funding Information later in the submission form are also declared in your Financial Disclosure statement.<br><br>View published research articles from *PLOS ONE* for specific examples.<br><br>**NO authors have competing interests**<br><br>Enter: *The authors have declared that no competing interests exist.*<br><br>**Authors with competing interests**<br><br>Enter competing interest details beginning with this statement:<br><br>*I have read the journal's policy and the authors of this manuscript have the following competing interests: [insert competing interests here]*<br><br>* typeset	
**Ethics Statement**<br><br>Enter an ethics statement for this submission. This statement is required if the study involved:<br><br>• Human participants<br>• Human specimens or tissue<br>• Vertebrate animals or cephalopods<br>• Vertebrate embryos or tissues	N/A

• Field research<br><br>Write "N/A" if the submission does not require an ethics statement.<br><br>General guidance is provided below. Consult the submission guidelines for detailed instructions. **Make sure that all information entered here is included in the Methods section of the manuscript.**	

Powered by Editorial Manager® and ProduXion Manager® from Aries Systems Corporation

**Format for specific study types**<br><br>**Human Subject Research (involving human participants and/or tissue)**<br>• Give the name of the institutional review board or ethics committee that approved the study<br>• Include the approval number and/or a statement indicating approval of this research<br>• Indicate the form of consent obtained (written/oral) or the reason that consent was not obtained (e.g. the data were analyzed anonymously)<br><br>**Animal Research (involving vertebrate animals, embryos or tissues)**<br>• Provide the name of the Institutional Animal Care and Use Committee (IACUC) or other relevant ethics board that reviewed the study protocol, and indicate whether they approved this research or granted a formal waiver of ethical approval<br>• Include an approval number if one was obtained<br>• If the study involved *non-human primates*, add *additional details* about animal welfare and steps taken to ameliorate suffering<br>• If anesthesia, euthanasia, or any kind of animal sacrifice is part of the study, include briefly which substances and/or methods were applied<br><br>**Field Research**<br><br>Include the following details if this study involves the collection of plant, animal, or other materials from a natural setting:<br>• Field permit number<br>• Name of the institution or relevant body that granted permission	
**Data Availability**<br><br>Authors are required to make all data underlying the findings described fully available, without restriction, and from the time of publication. PLOS allows rare exceptions to address legal and ethical concerns. See the PLOS Data Policy and FAQ for detailed information.	Yes - all data are fully available without restriction

A Data Availability Statement describing where the data can be found is required at submission. Your answers to this question constitute the Data Availability Statement and **will be published in the article**, if accepted.<br><br>**Important:** Stating 'data available on request from the author' is not sufficient. If your data are only available upon request, select 'No' for the first question and explain your exceptional situation in the text box.<br><br>Do the authors confirm that all data underlying the findings described in their manuscript are fully available without restriction?	
**Describe where the data may be found in full sentences. If you are copying our sample text, replace any instances of XXX with the appropriate details.**<br><br>• If the data are **held or will be held in a public repository**, include URLs, accession numbers or DOIs. If this information will only be available after acceptance, indicate this by ticking the box below. For example: *All XXX files are available from the XXX database (accession number(s) XXX, XXX.)*.<br>• If the data are all contained **within the manuscript and/or Supporting Information files**, enter the following: *All relevant data are within the manuscript and its Supporting Information files.*<br>• If neither of these applies but you are able to provide **details of access elsewhere**, with or without limitations, please do so. For example:<br><br>*Data cannot be shared publicly because of [XXX]. Data are available from the XXX Institutional Data Access / Ethics Committee (contact via XXX) for researchers who meet the criteria for access to confidential data.*<br><br>*The data underlying the results presented in the study are available from (include the name of the third party*	A representative dataset has been specially generated and made available through the dedicated demo application accessible at the link: https://demo.izs.it/app_direct/tracing/.

and contact information or URL).<br>• This text is appropriate if the data are owned by a third party and authors do not have permission to share the data.<br><br>* typeset	
Additional data availability information:	

Manuscript Click here to access/download;Manuscript;Manuscript def.docx

Article

T-Racing: a modern tool for supporting epidemiological investigation in animal disease outbreaks

Luca Candeloro [1], Lara Savini [1], Annamaria Conte [1], Paolo Calistri [1], Diana Palma [1], Samuel Perticara [1], Anna Cecília Trolesi Reis Borges Costa [2*], Alessio di Lorenzo [1], Susanna Tora [1], Sara Serrani [1], Michela Toro [1], Karina Diniz Baumgarten [2, 3] and Fabrizio De Massis [1]

[1] Istituto Zooprofilattico Sperimentale dell'Abruzzo e del Molise "G.Caporale", Italy
[2] Departamento de Medicina Veterinária, Escola de Zootecnia e Medicina Veterinária, Universidade Federal de Lavras, Lavras Minas Gerais, Brazil.
[3] State Department of Animal Health. Companhia Integrada de Desenvolvimento Agrícola de Santa Catarina – CIDASC, Florianópolis, SC, Brazil

[*] Correspondence: anninha_veterinaria@hotmail.com

Abstract: The One Health approach unites efforts across human-animal-environment interfaces against shared threats like zoonotic diseases. T-Racing is a Shiny web application, that supports epidemiological investigations and helps contain livestock-related disease spread, aligning with One Health principles of safeguarding public health. The application uses Temporal Network Analysis techniques to address the dynamic nature of animal trade, facilitating backward and forward tracing strategies. Therefore, T-Racing leverages web services to retrieve data from multiple sources simultaneously and almost in real-time through the plumber package and is distributed using Shinyproxy. Indeed, T-Racing manages and analyze extensive and diverse datasets within the same environment, including animal movement data, disease outbreak data, and genomic data, all obtained from Italian National databases. In this work, we show T-Racing's capabilities by simulating epidemiological investigations of brucellosis and tuberculosis outbreaks that occurred in non–endemic areas of Italy. This tool supports epidemiological investigations by adopting a data-driven approach, guiding users through the analysis via an iterative process while leveraging their expertise. Therefore, it enables faster data analysis, improves understanding of disease transmission patterns, and facilitates prompt and targeted interventions.

Keywords: One Health; Epidemiology; Tracing; Temporal Network Analysis; Shiny web application; Outbreak investigation; Zoonoses management.

1. Introduction

Preventing and controlling communicable diseases in livestock, through early detection of outbreaks, increases the effectiveness of the emergency response and reduces the social, economic, and environmental costs associated with these events [1, 2]. Animals serve as an excellent proxy for monitoring novel and known zoonotic pathogens given that more than 60% of emerging infectious diseases in humans originate from animal sources [3-5]. This underscores the critical role of animal health in the implementation of the One Health approach, by detecting, monitoring, and predicting health hazards that could also affect environmental and human health since some pathogens can survive long periods in the environment [6]. Among the diseases that could affect human health are tuberculosis and brucellosis, both chronic infectious diseases caused by bacteria. Bovine

tuberculosis, caused by *Mycobacterium bovis* [7], is a zoonotic disease that was estimated to have a human prevalence varying from 0.4% to 76.7% worldwide [8]. On the other hand, animal brucellosis caused by *Brucella* spp., is a zoonosis of utmost significance, since it was projected to globally affect 2.1 million people by year [9]. The control of both diseases is anchored on the surveillance and control in animals, with the traceability of animal cases being of paramount importance to prevent occurrences in humans, especially for human brucellosis that does not have vaccination. In this context, the movement of animals is often considered a major risk for disease spreading between herds [10], and to humans when zoonoses are involved. Hence, livestock transportation data is registered in national and international databases to facilitate contact tracing in the event of an outbreak [11-15]. An example of an international official movements database is TRACES-NT (TRAde Control and Expert System New Technology). TRACES-NT is an information platform of the European Commission for the reporting, certification, and control of imports of animals, origin of animal products, food and feed of plants, and other products of veterinary interest. The system also registers trade and exports of animals and certain products of animal origin into the EU and intra-EU. Currently, TRACES-NT is the integrated computerized veterinary system with a single architecture provided in Decisions 2003/24/EC and 2004/292/EC (EU, 2019) [16, 17]. Consequently, the usage of animal movement systems facilitates tracing the spread of diseases within animal populations and the potential routes of exposure being critical for effective disease control and crucial in the final steps of eradication programs [18]. Contact tracing plays a pivotal role in identifying the necessary actions to break the transmission chain, to reduce the spread and duration of infectious diseases, thus preventing new cases [19]. When zoonoses are considered, this also means protecting public health, once contact tracing has the subsequent effect of providing assessments to stakeholders which is useful for quickly identifying and implementing containment, surveillance, and control systems. In this sense, technology is fundamental to building online tracing applications, that can improve the results of epidemiological investigations. Advanced analytics and link analysis can be used to uncover hidden patterns in the spread of diseases [20, 2]. Therewith, tracing applications have been developed to assist animal health officials in quickly identifying the animals involved in an outbreak, where and when the infected animals are located, and whether other susceptible animals might have been exposed to the disease. These actions would be taken by uncovering the missing or unexpected links from the contact details, identifying the animals that should undergo testing, tracking the geographical spread of the disease, and determining which farms are at the highest risk. Thus, tracing applications can help in disease control and surveillance during critical outbreak stages, saving time, preventing further spreading, minimizing costs and reducing the consequences of the disease outbreaks [21, 2]. Tracing applications enables veterinary services, epidemiologists, and competent authorities to respond quickly in identifying the "hot spots" by implementing effective targeted control and eradication measures. Although it may sound simple, the tracing application can become overwhelmingly complex and resource-intensive, however, using data visualization and analytics can significantly improve the performance and effectiveness, making the application an invaluable tool. Currently, due to the COVID-19 global pandemic, contact tracing strategies have been widely used and many contact-tracing web apps have been developed for this purpose in human health [22, 23]. In veterinary epidemiology, there is limited literature on specific contact tracing web applications, such as EpiContactTrace [2], which is an open-source tool implemented in R and EpiExploreR, a Shiny web application that integrates disease mapping and spatiotemporal analysis tools [24]. Even though there are tracing applications already developed in the veterinary field, there is still a need for a web tool that can manage various and heterogeneous datasets and

incorporate analytical features to quickly perform forward and backward tracing within a spatial-temporal environment. Therefore, the aim of this paper was to introduce T-Racing, a web-based operational tool that has been created to assist veterinary epidemiological investigations in Italy. Based on our knowledge, T-Racing has several distinctive features, including its ability to integrate diverse data sources, its user-friendly interface, and its scalability. This tool is designed specifically to facilitate veterinary public health institutes, services, epidemiologists, veterinary professionals, and competent authorities to explore and analyze the complex spatiotemporal pathways that shape disease spread. It enables users to draw meaningful conclusions based on a data-driven, user-guided approach.

2. Materials and Methods

T-Racing is an application providing users with flexibility and facilitating the usage of methods and tools through an interacting interface in an environment composed by epidemiologically useful data.

2.1 Data sources and collection

T-Racing gathers near-real-time data from Italian National Databases which include disease notified outbreaks obtained from the National Information System for the Notification and Management of Animal Diseases (SIMAN) [25], animal movements records extracted from the Italian National Database for Animal Identification and Registration (NDB) [26], and genomic data, such as Multi Loci Sequence Type (MLST), provided by National Reference Centre for Whole Genome Sequencing of microbial pathogens database and bioinformatics analysis (GenPat) [27, 28]. These systems can be accessed through the VETINFO portal (https://www.vetinfo.sanita.it/), which is a secure platform to collect and present data for managing Italy's Animal Health and Food Safety system. VETINFO provides unified access to various stakeholders, including public health institutes, veterinary services, farmers, and competent authorities. To ensure user trust and regulatory compliance, the portal strictly adheres to GDPR compliance techniques, requiring users to access it with personal credentials and mandating specific functionalities for transparent and secure data reporting. The management of these databases is the responsibility of the Istituto Zooprofilattico Sperimentale dell'Abruzzo e del Molise "G. Caporale" (IZS-Teramo) on behalf of the Italian Ministry of Health.

T-Racing collects detailed data for each reported outbreak, including the disease name, outbreak code, geographical coordinates, region, province, administrative unit, suspected date, outbreak state (i.e., suspect, extinct, and confirmed), and species involved (including animal, vector species such as mosquitoes and flies, and birds, etc.). Animal movement data includes the origin and destination holding codes, animal single identification codes or batches of traded animals per species (including horses, cattle, buffalo, pigs, sheep and goats), and the date of the movement. The system also considers various holding attributes (livestock properties), such as the holding type (e.g., farm, slaughterhouse, staging point, transit station, market, assembly centre, foreign country, etc.), production type (e.g., milk, meat, wool, reproduction, etc.), address, and geographical coordinates. Additionally, in terms of genomic data, T-Racing records the holding code, animal single identification code, Sequence Type number, and date of sampling. Furthermore, T-Racing relies on data collected over the past 7 years extracted from the databases through daily scheduled procedures.

2.2 Temporal Network Analysis Methods in T-Racing

The application uses Temporal Network Analysis techniques [29] to address the dynamic nature of animal trade, enabling quick and efficient backward (BCT) and forward (FCT) tracing strategies. In the T-Racing environment, the animal trading network is represented as a time-ordered directed and weighted network, where holdings are the nodes, animal movements between holdings on a given day are the edges, and the number of moved animals is the weight (see Fig 1 on the left).

Animal movements are recorded in the NDB at the administrative farm owner (farmer) level. Consequently, multiple farmers from the same holding may be represented as different nodes (see Fig 1, top right), causing the network to be artificially fragmented. This fragmentation significantly impacts the analysis of diseases affecting multiple animal species. To address this issue, the application allows for network aggregation, ensuring that each node represents the physical holding, i.e. the geographical location to where the animals transited. Thus, a node can represent multiple owners at a fiscal level (see Fig 1, bottom right).

The framework of time-ordered networks, essentially directed acyclic graphs [29], is useful for understanding flow dynamics [30], particularly in the context of diseases spread through direct contact. We assume diseases spread along paths driven by animal movements, so accounting for the direction and timing of these movements allows us to detect temporally valid paths, providing a likely estimation of disease spread, similar to the Susceptible-Infection network-based compartmental model (where the contact rate is one). Direction and the timing of animal movements are important for detecting disease spread because they highlight impossible paths, and or too-long time elapsed for short paths [30, 31]. For example, in Fig 1, if farm A is infected at time zero, it can infect holding B, which can then infect holding C through subsequent animal movements. Since the probability of disease transmission decreases with increasing degrees of separation, we typically limit the search for plausible paths to a maximum degree of separation, or "level depth." Depending on the disease, the time elapsed between events might be too long to assume that nodes remain infectious and undetected, requiring a restriction of the time horizon to a specific number of days, known as "temporal depth." The choice of depth, in terms of both days and levels, affects the resulting subnetwork and depends on factors such as the disease's infectiousness, incubation period, and the timing of surveillance system controls. Furthermore, the removal of node(s) and/or edge(s) from a time-ordered directed network involves the change of the resulting paths. Therefore, each network modification requires a recalculation of the paths.

Contact tracing following a positive detection has been widely used in various fields, with different interpretations and applications depending on the context [30, 32]. In T-Racing, FCT, BCT, and a combination of both (FCT-BCT) strategies have been implemented to support track and control the spread of diseases. These methods provide different perspectives and insights depending on the context and time of disease outbreaks. **FCT method** focuses on identifying the pathways of pathogen spread starting from an index node, known as the seed, and an event date. By analyzing outgoing movements within a specified depth (in terms of days and levels) FCT highlights the nodes that the disease could have potentially reached. This approach supports targeted disease control more effectively than a general surveillance system, which, despite being timely conducted, can only prevent a portion of the potential disease spread (as illustrated in Fig 2). The control shown in Fig 2 can be considered a real-time monitoring and proactive control in response to a new outbreak detection. It is performed as soon as the new outbreak is notified to detect its outgoing movements in the recent past. The subnetwork found in a forward direction will help identify positive farms and prevent the spread of infection to other farms in the future. **BCT method**, on the other hand, aims to trace the primary source of infection from a newly detected outbreak. This retrospective

strategy consists of finding temporal valid pathways through incoming movements within specific time constraints and degrees of separation. Typically, BCT uses a broader time window compared to FCT, allowing for a comprehensive analysis of the infection's origins. Both methods rely on (require) updated data. Nowadays, current Italian legislation requires the registration of animal movements in NDB within 7 days, but this does not always happen. More often, and especially for species regulated more recently, registration times are longer. This means that some of the real connections may be missing in NDB when performing FCT or BCT strategies, leading to undetected possible infectious nodes. **Combination of strategies (FCT-BCT)** involves using both methods to enhance disease control efforts. For instance, after performing a BCT starting from multiple recent outbreaks within a large time window, the resulting subnetwork may include numerous nodes. If among these nodes there are outbreaks notified in the past, an FCT can be conducted on this newly identified BCT subnetwork. This intersection (BCT ∩ FCT) reveals the subnetwork connecting the outbreaks through temporally valid paths. Then, by working in the past, when data is more likely to be updated in NDB, this strategy identifies nodes that have been exposed to infected animals but have not yet tested positive. A comparison of animal movement dates with the last inspections on the farms can help promptly identify nodes for further inspection, aiding in containing the disease's spread. In the end, such kind of strategies could be performed using batches of animals (and thus letting the paths highlight the spread accounting for transmission between animals sharing the same farms) or considering the individual animals moving forward or backward from a seed farm. These strategies can be applied either to batches of animals (highlighting the spread through shared farm contacts) or to individual animals moving between farms. The T-Racing application facilitates these analyses, enabling users to leverage epidemiological information and expert knowledge to transform vast amounts of data into clear, actionable guidelines for disease control.

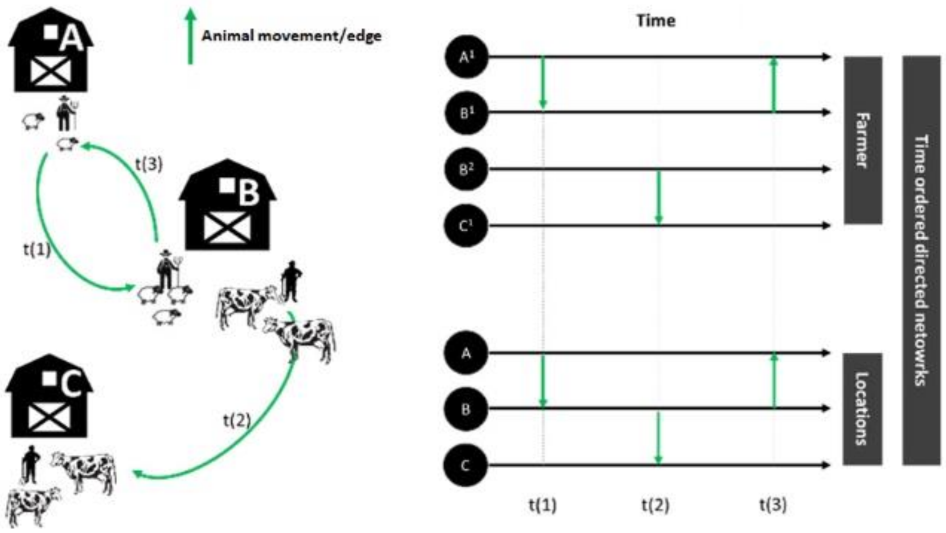

Fig 1 Network fragmentation and aggregation in animal movement records. The top right panel shows how multiple farmers of the same holding are represented as different nodes, leading to artificial fragmentation. The bottom right panel demonstrates the aggregation process, where each node represents the physical holding, encompassing multiple owners at a fiscal level.

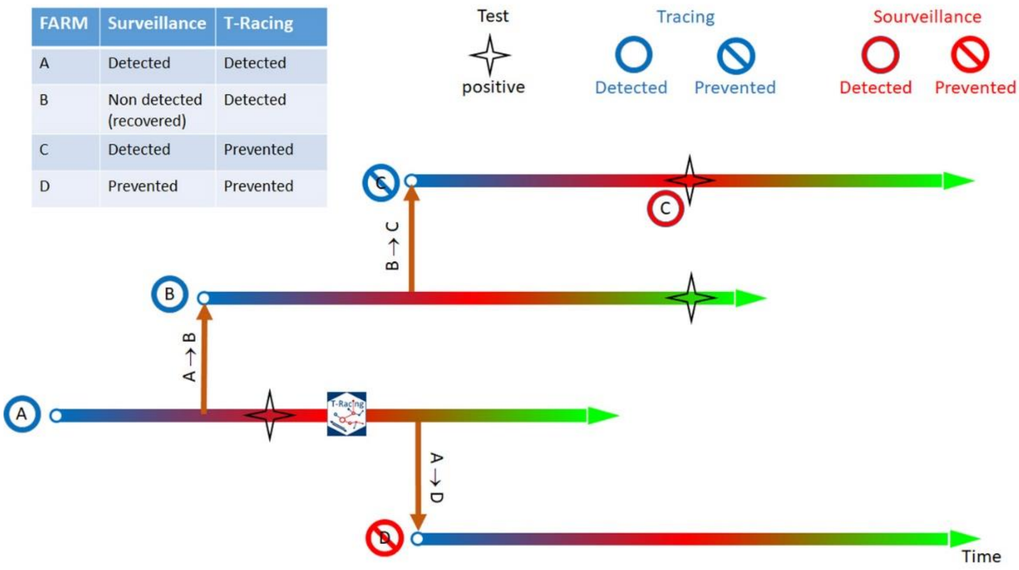

Fig 2 Schematic illustration of the importance of tracing in preventing and detecting diffusion pathways versus the simple farm isolation when only positivity recorded in the context of the surveillance program is considered.

2.3 T-Racing Design

T-Racing interface allows users to quickly query, filter, analyze, and visualize data through interactive graph, map, and tables for a seamless and intuitive experience. Fig 3 provides a conceptual map of the application, highlighting its primary dashboard sections and features.

Tools for extracting and viewing animal trade data, including:

- Filtering data based on multi-species selections, time frame, tracing direction mode (backward or forward methods), network geo-aggregation mode, and including/excluding animal trade data from/to foreign countries or slaughterhouses;
- Multi-seed selection for tracing, allowing users to either type or select one or more farm codes from the list of farms or select outbreak codes from the list of officially notified outbreaks within a specified time frame. Alternatively, the user can select all farms and/or outbreaks falling within a spatial buffer (neighbours distant by kilometres) around selected farms and outbreaks;
- Tracking a single animal by typing its identification code and viewing all resulting spatial-temporal paths within a specified time frame.

The system retrieves movement data based on these filters from the NDB and builds a time-ordered directed weighted network originated by the set seeds. These tools are useful for analyzing diseases that are transmitted through direct contact (i.e. brucellosis or tuberculosis) and diseases that are transmitted through spatial proximity (i.e. African swine fever or vector-borne diseases such as west Nile or Bluetongue, etc.), using spatial buffering and contact tracing methods.

Tools for customizing network visualization and optimization:

- Customizing graph visualization by setting from a range of layout options such as hierarchical, geographical, or nicely mode (default mode). Users can also make the shape and colour of nodes or the colour of edges reflect selected data attributes such as

holding type or production type for nodes, and level, time, or species for edges. All possible attribute colours and shapes are predefined in a configuration file;
- Removing unnecessary nodes or edges from performed tracing;
- Highlighting within the network all edges involving animals moved from/to the seed, or reducing the network to only those edges involving animals moved from/to the seed, according to the tracing direction mode set by the user;
- Reducing the network size by decreasing network deepness (in days or levels).

For each network reduction method applied, a new time-ordered network will be created to ensure and maintain the temporal coherence in the events.

Tools for epidemiological information integration and results printing:

- Adding a layer of sequenced pathogens for the animals;
- Adding a layer of outbreaks for a specific disease or reducing the resulting network by keeping only temporally valid paths among involved outbreaks by a combination of FCT and BCT methods;
- Download the network's tables (or a selection of them) in Excel format;
- Storing the results in a dynamic HTML report for offline use.

By using these tools, the network graph can be customized and fine-tuned to provide a more meaningful representation of the data.

The T-Racing application was created by a team of experts including epidemiologists, veterinarians, mathematicians, and computer scientists. This collaborative effort spanned all stages of development, from gathering data to creating tools and building a user-friendly interface. The team has extensive experience in epidemiological tracing and dynamic network analysis, as evidenced by similar web applications they have developed since 2011 [24, 33, 34, 35, 36]. T-Racing is an improved version of previous applications, offering faster speeds, additional features, and integration of various epidemiological datasets. Currently, it is available on the internal network of IZS-Teramo and used by our veterinarians and epidemiologists. It is pending technical adjustments and official approval from the Italian Ministry of Health to be integrated into the VETINFO portal. This integration will ensure secure access and the protection of sensitive data, as T-Racing inherits the robust GDPR techniques already applied in the portal.

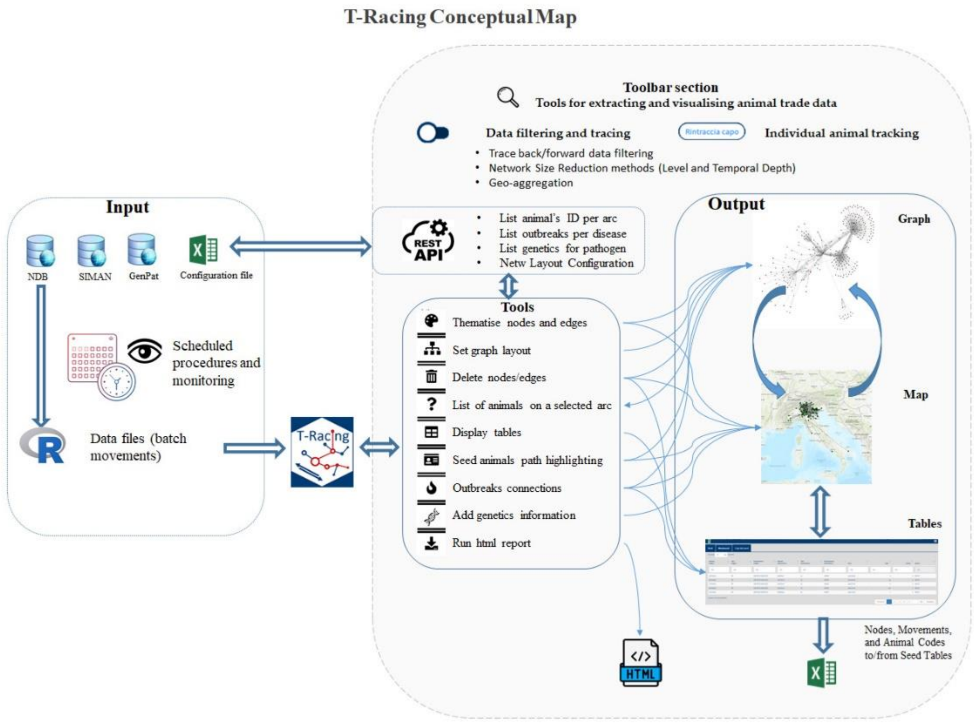

Fig 3 T-Racing conceptual map.

2.4 Data flow and system architecture

T-Racing has been developed using the Shiny library by the R statistical environment, and leaflet and visnetwork libraries were used for map and graph visualization respectively (Table 1 contains a list of all packages used). The application manages large amounts of data from different information systems and shares them efficiently and securely in real-time. Differently from the serverless JavaScript web apps, computationally heavy services are executed on a remote server, and the Plumber package allows the application to query data sources in real-time, through web API.

The system architecture facilitates the application scalability, thanks to shiny proxy (a free and open-source Docker-based application). The described stack and the involved application modules are displayed in Fig 4.

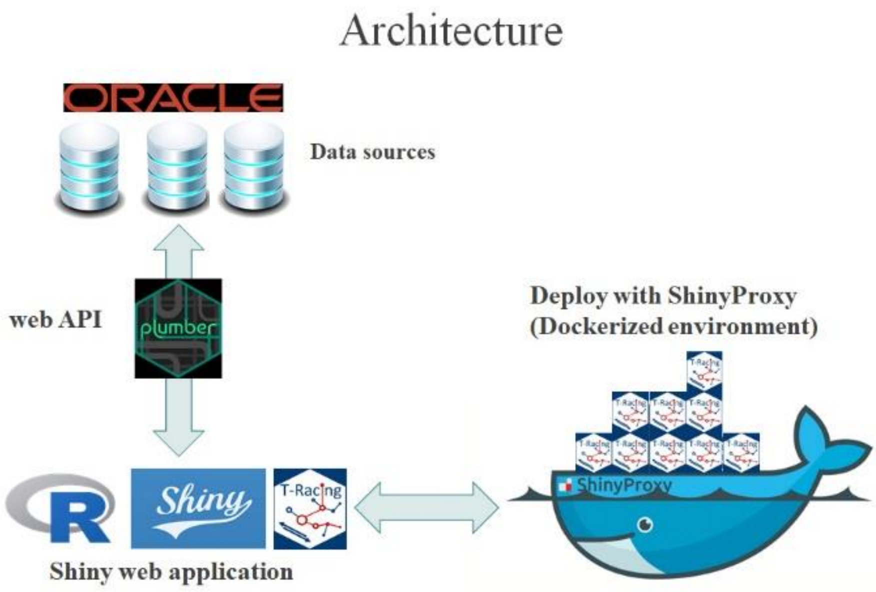

Fig 4 T-Racing architecture.

Table 1. List of the R-packages used in T-Racing development.

R-package	Use	Reference
shiny (v. 1.7.1)	UI/SERVER	Winston Chang, Joe Cheng, JJ Allaire, Carson Sievert, Barret Schloerke, Yihui Xie, Jeff Allen, Jonathan McPherson, Alan Dipert and Barbara Borges (2021)
shinyjs (v. 2.1.0)	UI/SERVER	Dean Attali (2020). shinyjs: Easily Improve the User Experience of Your Shiny Apps in Seconds
shinyWidgets (v. 0.6.4)	UI	Victor Perrier, Fanny Meyer and David Granjon (2021). shinyWidgets: Custom Inputs Widgets for Shiny
Shinydashboard (v. 0.7.2)	UI	Winston Chang and Barbara Borges Ribeiro (2018). shinydashboard: Create Dashboards with 'Shiny'
shinyBS (v. 0.61.1)	UI	Eric Bailey (2015). shinyBS: Twitter Bootstrap Components for Shiny
shinybusy (v. 0.3.0)	UI/SERVER	Fanny Meyer and Victor Perrier (2020). shinybusy: Busy Indicator for 'Shiny' Applications
DT (v. 0.22)	UI	Yihui Xie, Joe Cheng and Xianying Tan (2021). DT: A Wrapper of the JavaScript Library 'DataTables'
rintrojs (v. 0.3.0)	UI/SERVER	Carl Ganz (2016). rintrojs: A Wrapper for the Intro.js Library. Journal of Open Source Software, 1(6), October 2016
shinycssloaders (v. 1.0.0)	UI/SERVER	Andras Sali and Dean Attali (2020). shinycssloaders: Add Loading Animations to a 'shiny' Output While It's Recalculating
htmltools (v. 0.5.2)	UI	Joe Cheng, Carson Sievert, Barret Schloerke, Winston Chang, Yihui Xie and Jeff Allen (2021). htmltools: Tools for HTML
leaflet (v. 2.1.1)	UI	Joe Cheng, Bhaskar Karambelkar and Yihui Xie (2022). leaflet: Create Interactive Web Maps with the JavaScript 'Leaflet' Library
rgdal (v. 1.5.31)	SERVER	Roger Bivand, Tim Keitt and Barry Rowlingson (2021). rgdal: Bindings for the 'Geospatial' Data Abstraction Library
sp (v. 1.4.7)	SERVER	Pebesma, E.J., R.S. Bivand, 2005. Classes and methods for spatial data in R. R News 5 (2)
bezier (v. 1.1.2)	SERVER	Aaron Olsen (2018). bezier: Toolkit for Bezier Curves and Splines
geosphere (v. 1.5.14)	SERVER	Robert J. Hijmans (2021). geosphere: Spherical Trigonometry
visNetwork (v. 2.1.0)	UI	Almende B.V., Benoit Thieurmel and Titouan Robert (2019). visNetwork: Network Visualization using 'vis.js' Library
igraph (v. 1.3.1)	SERVER	Csardi G, Nepusz T: The igraph software package for complex network research, InterJournal, Complex Systems 1695. 2006
ggplot2 (v. 3.3.6)	SERVER	H. Wickham. ggplot2: Elegant Graphics for Data Analysis. Springer-Verlag New York, 2016

RColorBrewer (v. 1.1.3)	SERVER	Erich Neuwirth (2014). RColorBrewer: ColorBrewer Palettes
xlsx (v. 0.6.5)	SERVER	Adrian Dragulescu and Cole Arendt (2020). xlsx: Read, Write, Format Excel 2007 and Excel 97/2000/XP/2003 Files
dplyr (v. 1.0.9)	SERVER	Hadley Wickham, Romain François, Lionel Henry and Kirill Müller (2021). dplyr: A Grammar of Data Manipulation
jsonlite (v. 1.8.0)	SERVER	Jeroen Ooms (2014). The jsonlite Package: A Practical and Consistent Mapping Between JSON Data and R Objects. arXiv:1403.2805
curl (v. 4.3.2)	SERVER	Jeroen Ooms, Hadley Wickham, RStudio
rJava (v. 1.0.6)	UI	

2.6 Demo

The T-Racing application is not publicly available as it handles sensitive data from Italian national information systems. Therefore, a demo version has been developed to demonstrate its potential to the scientific community. This demo uses a limited and anonymized dataset. Adjustments have been made by adding noise and rounding coordinates to the second decimal place in the georeferencing of holdings, which may result in some holdings falling outside the Italian boundaries. Additionally, it has been translated into English, while the original application is in Italian. The original development and publication environment, as well as the web services, have been duplicated and adapted for the demo. The underlying dataset is contained in a single Oracle database. The demo environment provides a comprehensive preview of its features, as listed in Table 2.

Table 2. List of Data available in T-Racing Demo.

**Data**	**From**	**To**	**Type/feature**
**Movements**	01/12/2017	30/06/2023	batch and individual for bovine and buffalo
**Outbreaks**	01/12/2017	30/06/2023	*Brucellosis*, *Tuberculosis*
**ST**	01/01/2015	01/12/2018	*B. abortus* and *B. melitensis* species

2.5 Brucellosis and Tuberculosis: Rationale for Case Studies and Italian Regulations

The following case studies were considered:

Brucellosis in the Marche region (central Italy). On December 9, 2019, a brucellosis outbreak was confirmed in central Italy, specifically in the Marche region. The T-Racing application was utilized to identify the potential source of the infection. Only bovine species were considered, while slaughterhouses and foreign countries were excluded from the analysis. The known outbreak code from a list of proposed brucellosis outbreaks was selected as the seed for a trace backwards analysis encompassing all incoming movements to the identified outbreak over the past two years at 4 depth levels. The resulting network was refined using the "outbreaks connection" tool, and genetic data was added to support the conclusions drawn from the final network.

Tuberculosis outbreaks affected Elba Island and the nearby Tuscany coast in June 2023. A forward tracing from March to December 2023 to identify the likely spread trajectory leading up to the outbreak on Elba Island was used. Only bovine species were considered, slaughterhouses were included, and foreign countries were excluded from the analysis. Additionally, we utilized a "Seed animal's path highlighting" tool to emphasize the flow of the infection about the animals coming from the seed.

A tuberculosis outbreak occurred in the Cuneo province in December 2022. A trace backward analysis over a 13-month temporal window with a level depth of 4 was used to determine the most likely source of infection. Network reduction was applied using the

dedicated tools for highlighting paths among involved outbreaks ("outbreaks connection" tool).

We used T-Racing to investigate brucellosis and tuberculosis outbreaks in non–endemic areas of Italy, where an occurrence of an outbreak should trigger a rigorous investigation by the official veterinary service to prevent the disease from spreading, demonstrating the speed and effectiveness of the investigation carried out with the proposed web app.

Brucellosis caused by *Brucella abortus*, and tuberculosis caused by *Mycobacterium bovis* are important zoonoses that constitute a serious hazard to public health, and were the chosen diseases due to their chronic development, allowing for the use of FCT and BCT methods with a broad time-elapse. Prevention of these zoonoses in humans depends on the control of the disease in animals. Livestock movement data represent a valuable source of information to understand the pattern of contacts between holdings, which may determine the spread of such diseases. The first national plans for the control of bovine tuberculosis and brucellosis were approved with the Ministerial Decree of 11 and 12 March 1965, respectively. Through the Ministerial Decree of 27 August 1994 n. 651 entered into force in Italy the national eradication plan for bovine brucellosis, which establishes the measures to be applied to cattle farms to obtain the eradication of the disease for the protection of public health and protection of officially free herds. The regulation determines for control the use of official serological tests twice a year at an interval of not less than three and not more than six months. All animals over the age of twelve months in all cattle farms have to be tested, even if are located in the wild. Similar procedures have been established (test all animals in all heads at once, with 3 to 6 months intervals) with Ministerial Decree 15 December 1995, n. 592 – Regulation concerning the national plan for the eradication of tuberculosis in cattle and buffalo herds – by testing with the tuberculin intradermal test.

3. Results

T-Racing Demo, which uses a limited and anonymized dataset, is available at this link https://demo.izs.it/app_direct/tracing/. The basemaps throughout all the following figures were created using ArcGIS® software by Esri and integrated into the shiny web application using the leaflet.providers R library, sourced from leaflet-providers.js (https://github.com/leaflet-extras/leaflet-providers). ArcGIS® and ArcMap™ are the intellectual property of Esri and are used herein under license. Copyright © Esri. All rights reserved. For more information about Esri® software, please visit www.esri.com.

Fig 5 shows the T-Racing interface, which displays temporally valid paths originating from a brucellosis outbreak in Sicily, an Italian southern island, using the trace forward method. The investigation revealed this outbreak, detected in July 2020, was likely the source of two subsequent outbreaks in Pisa, Tuscany, northern Italy. Animals moved from the seed in March reaching, via a transit station, two farms in Pisa in April and May, but the outbreaks were only detected by surveillance in December 2020. The simultaneous visualization of the queryable and interactive graph, map, and tables, combined with the iterative use of tools for network calibration and targeted result personalization, offers a more comprehensive and user-friendly result viewing.

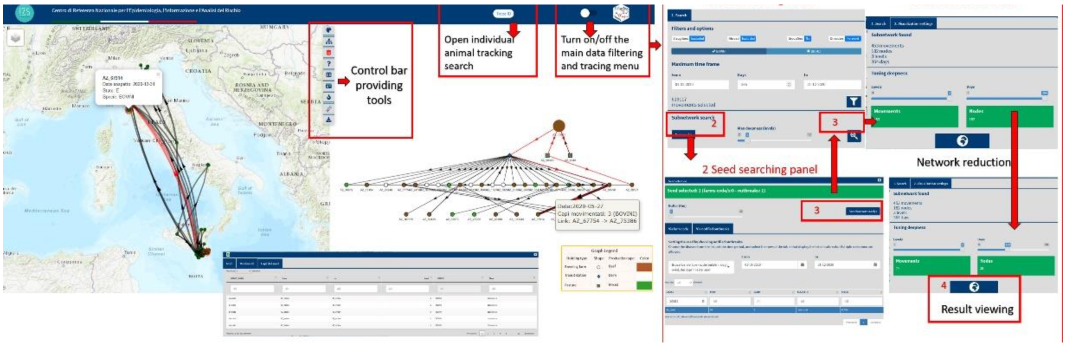

Fig 5 The T-Racing interface (on the left) shows the subnetwork resulting from the trace forward analysis on a map, graph, and tables and the steps followed to get the results in the interface are shown on the right. This analysis was performed using a brucellosis outbreak in July 2020 in the Sicily Region as the seed, extracting all bovine movements in 2020 up to the third level of depth (three degrees of separation). A time frame-based network reduction/optimization method was applied for the result. The "hierarchical" graph layout shows all nodes and edges in the subnetwork, highlighting probable disease pathways and nodes reached during the study period. Node shape and colour represent farm type and production activity (see legend). The larger, red-bordered icon represents the seed, with each node labelled by farm code. Arrows indicate animal movements between two nodes, with the width proportional to the number of animals moved. Red-coloured arrows highlight paths followed by animals moving from the seed (using the "Seed animals path highlighting" tool). Fire icons on the map represent brucellosis outbreaks detected during the selected period and included in the subnetwork (using the "Outbreaks connections" tool). Tooltips and tables provide detailed information, facilitating navigation between the map, graph, and tables.

3.1 T-Racing - Case Studies

3.1.1. Tracing backward from a notified outbreak of brucellosis in the Marche region of central Italy, in December 2019, to identify its potential sources of infection.

The search for the subnetwork resulted in 1.825 movements involving 386 nodes. The subnetwork covers a substantial area of central Italy, including holdings that reach the seed even from the islands of Sicily and Sardinia. The theming of nodes based on type reveals the involvement of different holding types. In addition to breeding farms, there are pastures (structures that play a significant role in the spread of brucellosis) and a hub node, a genetic collection centre located in Perugia province, Umbria region, in central Italy, that connects 132 nodes (Fig 6). To make the network more manageable and relevant for the conducted tracing, a subnetwork was created by setting 3 levels and reducing the depth to 399 days, resulting in 198 movements and 38 nodes. To identify plausible paths of disease spread, the application allows the utilization of notified outbreaks within a customizable time frame for the specific disease. These outbreaks are visualized on the map, enabling the tracing to be narrowed down to the connections between outbreaks included in the tracing. This helps identify a focused subset of connections to investigate. The subnetwork includes multiple brucellosis outbreaks, primarily located in the southern and central regions. The reduced network, created by keeping only temporally valid paths among involved outbreaks within the network, consists of 94 movements involving 11 nodes. At this point, we can assess the presence of genetic information as further confirmation of the tracing's plausibility. To do so, we utilize the tool by adding the available sequence type for *Brucella abortus*. The seed is an outbreak for which sequence type "2" is available, and the identified origin zones are also affected by clusters of the same sequence type. An interesting aspect is to determine how many of these links involve the same animals that reached the seed. The dedicated tool allows for theming the links based on this characteristic by searching for individually identified animals. The visualization highlights on the subnetwork the paths involving the same animals that reach the seed. These highlights paths consist of only 21 movements involving 5 nodes (including the seed and one pasture, which is the presumed source of infection). Further investigation of individually identified and traced animals revealed that an infected

animal, identified as sequence type "2" in the seed, originated from a pasture, which was subsequently identified as the plausible source of the sought-after infection (Fig 7).

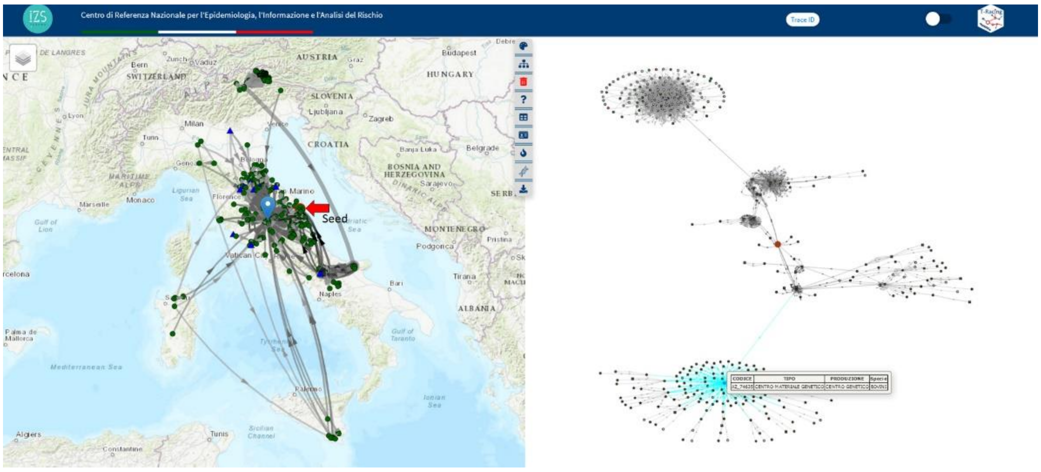

Fig 6 Trace backward method. Sequential investigation steps: 1) Data Filter, 2) Set seed by outbreak code search, and 3) Network view. The trace-back focusing on bovine species, excluding slaughterhouses and foreign countries, was conducted to trace the outbreak occurring in December in the Marche region, in Italy. 1,825 movements involving 386 nodes were identified within a broad time spanning the past two years. The seed is contoured in red. The network covers a substantial area of central Italy, including farms extending to the islands (Sicily and Sardinia). Node theming based on structure type highlights diverse holding types, including pastures and a genetic collection centre in Perugia, highlighted in light blue on the graph (simultaneously, its marker appears on the map).

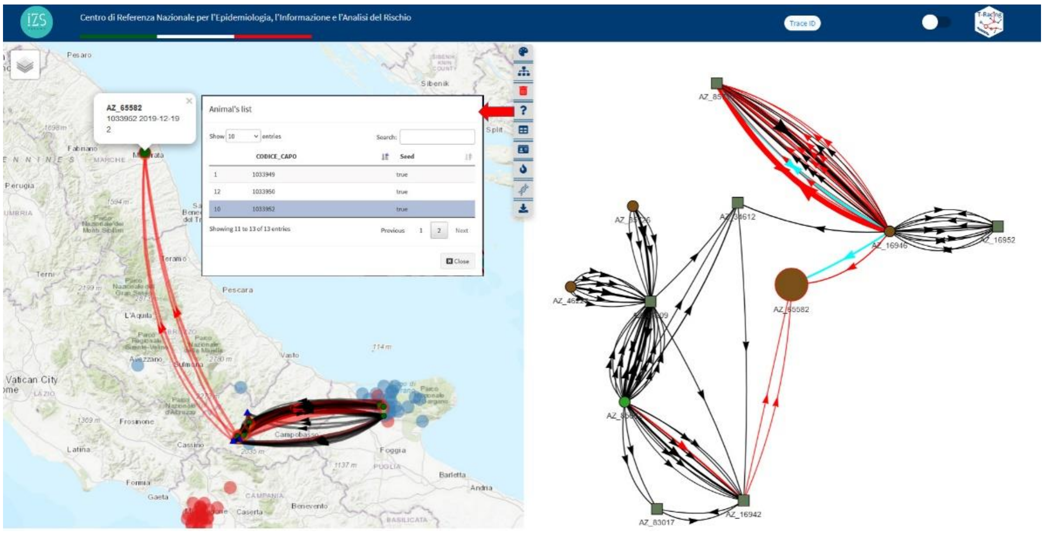

Fig 7 The subnetwork result after the following four sequential steps: 1) Network reduction by setting the network level to 3 and days depth to 399 (the new subnetwork size is 198 links and 38 nodes), 2) Further reducing the subnetwork by keeping only temporally valid paths among involved outbreaks (resulting in a subnetwork size of 94 links and 11 nodes; the four detected brucellosis' outbreaks involved are shown on the map with red fire icons), 3) Adding the sequence type for Brucella abortus layer to the map (type 2 in blue circles) and displaying a popup on the seed showing the identification code of the animal, sampled date, and detected Brucella abortus sequence type, and 4) Highlighting on the graph are all links involving the same animals that reached the seed (red links). Further investigation on "individually identified animals," found by querying the highlighted links (in light blue on the graph), revealed that an infected animal originating from the pasture reached the seed. This animal is listed in the Animal list produced by the application, matching the animal reported in the map popup.

3.1.2. Tracing the flow of tuberculosis outbreaks: Elba Island and Cuneo Province.

-Four BTB outbreaks were notified in June 2023 in a small, non-endemic area, in Italy, affecting Elba Island and the nearby Tuscany coast. Using T-Racing allowed for the detection of the real flow of the disease spread. The resulting network highlighted an animal movement of nine cattle in April 2023, which reached farms on the nearby coast. This established the real source of the infection and the most likely flow of the spread (Fig 8). We discovered that the animals reaching the outbreaks were the same ones that had left Elba Island and one of them (at the time of writing this article) had not yet been slaughtered.

-**In December 2022, an outbreak of BTB occurred in the Cuneo province of the Piedmont region in northern Italy.** The resulting network comprised 306 farms and 664 connections, predominantly located in the Sicily region. For a more focused investigation, we considered only temporally valid paths connecting BTB outbreaks included in the network resulting in a subnetwork of 50 connections involving 9 breeding farms in the Sicily region, four of which were BTB outbreaks, and two pastures. The investigation suggested that four outbreaks occurring in the previous months are the most likely source of infection. Additionally, the 119 animals reaching the Cuneo province in June and July originated from a fattening farm where they were likely infected by mixing with animals coming from the four outbreaks. The results indicate the need for targeted control measures for the other five farms involved in the revealed diffusion paths (Fig 9).

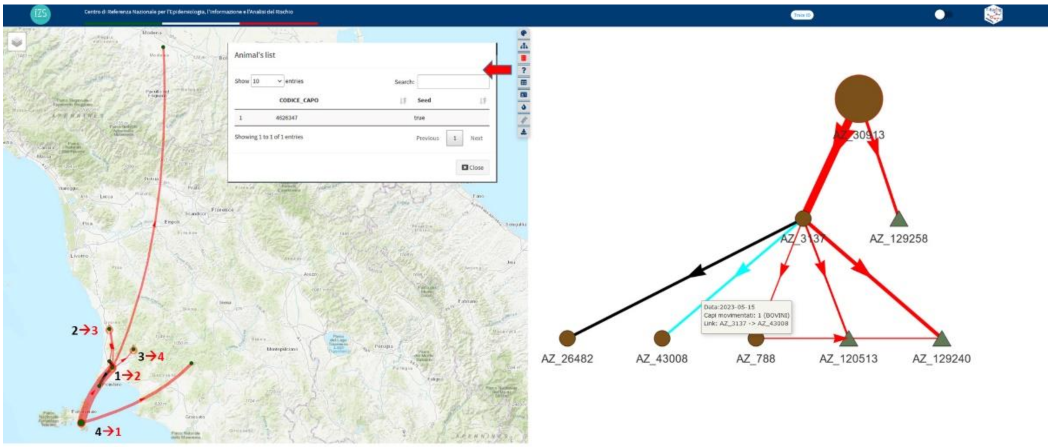

Fig 8 Trace forward method. The map and graph show outbreaks that occurred on Elba island and the nearby Tuscany coast (black numbers '1' to '4', represent the notification timing, with 1 the earliest and '4' the most recent notified one). The network of animal movements involving the outbreaks is displayed with red links indicating the presence of at least one animal originating from the seed, and black link representing the movement of animals not originating from the seed. The notified outbreaks' timing contrasts with the real flow of disease spread revealed by T-Racing analysis (red numbers '1-4'). The triangles on the graph are slaughterhouses. Further investigation on "individually identified animals," found by querying the highlighted link (in light blue on the graph), revealed that one animal originating from the seed had not yet been slaughtered. This animal is listed in the Animal's list produced by the application.

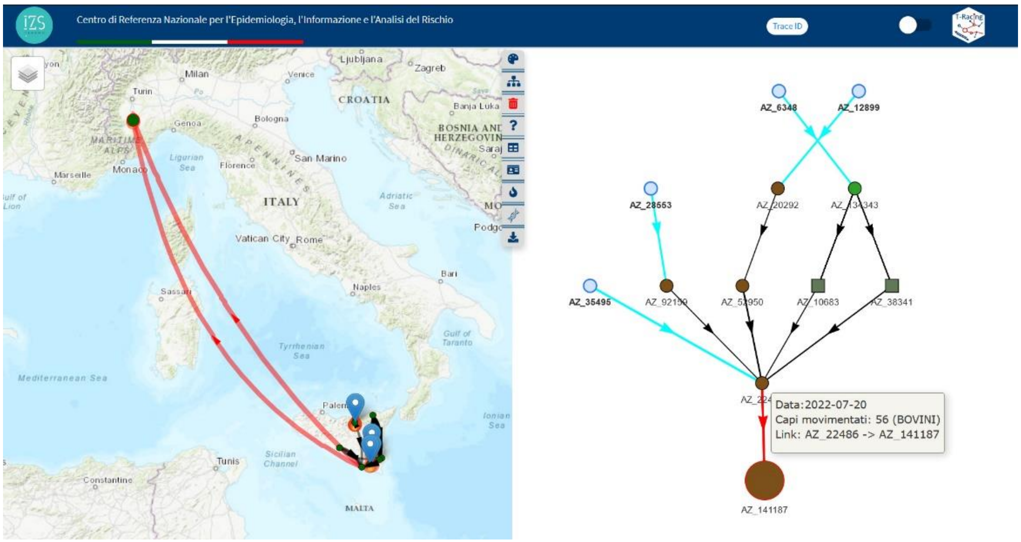

Fig 9 Trace backward method. The network resulting from the BTB outbreak occurred in Cuneo province, in the Tuscany region in December 2022 involving 306 farms and 664 links, mostly located in the Sicily region. The map and graph show the reduced network by considering only temporally valid paths connecting involved outbreaks consisting of 50 connections and 12 holdings which 9 breeding farms in the Sicily region (circles on the graph), four of which are BTB outbreaks (fire icons and marked on the map and highlighted in light blue on the graph), and two pastures (squares on the graph). The links involving animals reaching the Cuneo outbreak are in red colour.

4. Discussion

Timely identification of outbreaks and systematic contact tracing can be effective in stopping the transmission chain of contagious diseases, especially with strong collaborations between epidemiological teams, laboratory surveillance, and decision-making officials, improving outbreak management, in a One Health context [37]. An important feature of the modern response to epidemics is the increasing focus on exploiting all available data to inform the response in nearly real-time and allow for the data evidence-based decision-making [38]. T-Racing has the potential to significantly enhance animal disease control measures, as it can rapidly organize crucial contact information from extensive datasets and identify connections in a multi-outbreak investigation. It introduces new methods and tools currently not available in other systems or tools [2, 24, 33, 34, 35, 36]. These include multi-seed/species selection, seed selection using a spatial buffer around cases or outbreaks, and the combination of strategies (FCT-BCT) configuring itself as an innovative application in its field. Thus, the application efficiently supports epidemiological investigations, as it was shown in the demo examples for brucellosis and tuberculosis. In these examples, it is possible to see how deep into the transmission chain the T-Racing allows us to explore. The initial tracing example has identified a likely path of disease spread and the presumed origin, starting from a relatively large and diverse network (1.825x386) in both spatial and temporal dimensions. By using interactive and iterative tools, along with additional information such as disease outbreaks, genetic data, and movements of individually identified animals, it was possible to narrow down the focus to 21 movements and 5 nodes in just a few steps, saving a significant amount of time. Trying to achieve the same result by simply extracting the movement network through a query of the NDB would have been extremely difficult, like finding a needle in a haystack. The second example clarified the actual timing of the tuberculosis spread. Importantly, the animals from the seed had not been slaughtered at the time of writing this article, and in the last example, results indicate a need for targeted control measures for the other five farms involved in the revealed diffusion paths.

Considering chronic diseases, such as brucellosis and tuberculosis, clarifying the disease's spread patterns allows for focus measures of control, improving targeting financial and human resources, as well as time sensitive decisions. Noteworthy, the almost real-time data used, the possibility of analyzing many datasets at once and the user-friendly environment are also features that could greatly advance responses to emergencies with acute and highly contagious diseases when movement data could be generated in real-time.

Complementarily, the application of molecular epidemiological data into the analysis, validating the identified paths of disease (brucellosis) spread, is an example of a One Health approach by a multidisciplinary team on building a new technology for disease control. In this sense, in an era of rapid technological advancements, it is essential to foster innovation and develop tools that revolutionize epidemiological investigations, by combining cutting-edge technology with comprehensive data analysis, which could benefit trust between the Veterinary and Human Health systems while dealing with zoonosis [38]. T-Racing empowers clinicians, veterinarians, ecosystem health professionals, researchers, and policymakers to leverage the power of animal surveillance within the One Health perspective [3, 39]. In that regard, by embracing this innovative tool, stakeholders can gain valuable insights, make informed decisions, and take proactive measures to mitigate the impact of infectious diseases on animal and human health. Therewith, this dynamic approach enables the T-Racing application to provide accurate and timely data for epidemiological investigations. Complementary, the system emphasizes its data-driven nature, recognizing the crucial role of accurate data in conducting effective investigations and highlighting the need for robust practices on data management. Noteworthy, without reliable data, the utility and reliability of the T-Racing application would be compromised.

Although T-Racing is currently operational, the authors and team of experts will continue to develop future versions to improve the application by adding new datasets and functionalities. At present, the genetic data is restricted to the Brucella genus, but additional pathogen data will be incorporated as soon as it becomes available in GenPat. Data regarding testing from surveillance systems on farms will be included, and functions will be created to factor in the timing of testing when selecting the most probable disease spread paths. Also, detailed information about single animals, like demographics and parturition dates for calves, along with human cases will be considered. There is also an evaluation underway for the development of scheduled algorithms that will operate directly on the database to identify critical networks to be automatically provided to T-Racing's users. This will enable more efficient, rapid, and straightforward validation and initiation of investigations. Concurrently, work is ongoing for a more general application that allows users to upload their own data, and its source code will be freely distributed on GitHub.

5. Conclusions

The T-Racing web app represents a modern and forward-thinking approach to support epidemiological investigations in animal disease outbreaks. It provides a unified environment to manage and analyze the expanding pool of data sources iteratively by integrating advanced technology, comprehensive data management, and cutting-edge analytics. T-Racing has the capability to draw meaningful conclusions based on a data-driven, user-guided approach while leveraging its expertise. It facilitates evidence-based decision-making and serves as a leading tool in disease control, enhancing our capacity to address emerging challenges and safeguard global health from a One Health perspective.

Author Contributions: Conceptualization, L.C., L.S., A.C., P.C. and F.D.M.; methodology, L.C. and L.S.; software, L.C. and S.P.; validation, F.D.M. and P.C.; data curation, D.P.; writing—original draft preparation, L.S., L.C., A.C.T.R.B.C. and F.D.M.; writing—review and editing, L.C., L.S., A.C.,

F.D.M., P.C., S.P., A.D.L., D.P., S.T., S.S., and M.T.; project administration, P.C.; funding acquisition, P.C.. All authors have read and agreed to the published version of the manuscript.

Funding: This research was funded by Italian Ministry of Health (RC MSRCTE0219).

Conflicts of Interest: The authors declare no conflict of interest.

References

1 Schirdewahn, F., K. Lentz, H. H., Colizza, V., Koher, A., Hövel, P., & Vidondo, B. Early warning of infectious disease outbreaks on cattle-transport networks. PLOS ONE, 16(1), e0244999. https://doi.org/10.1371/journal.pone.0244999

2 Maria Nöremark and Stefan Widgren. EpiContactTrace: an R-package for contact tracing during livestock disease outbreaks and for risk-based surveillance. BMC Veterinary Research 2014, 10:71 Page 2 of 9 http://www.biomedcentral.com/1746-6148/10/71

3 Prata, J. C., Ribeiro, A. I., & Rocha-Santos, T. (2022). An introduction to the concept of One Health. One Health, 1-31. https://doi.org/10.1016/B978-0-12-822794-7.00004-6

4 Wondwossen A. Gebreyes, Daral Jackwood, Celso Jose Bruno de Oliveira, Chang-Won Lee, Armando E. Hoet, Siddhartha Thakur. Molecular Epidemiology of Infectious Zoonotic and Livestock Diseases. 2020. Microbiology Spectrum, 10.1128/microbiolspec.ame-0011-2019, 8, 2, https://journals.asm.org/doi/abs/10.1128/microbiolspec.ame-0011-2019

5 Neo, J. P. S., & Tan, B. H. (2017). The use of animals as a surveillance tool for monitoring environmental health hazards, human health hazards and bioterrorism. Veterinary Microbiology, 203, 40-48. https://doi.org/10.1016/j.vetmic.2017.02.007

6 D Kriebel, J Tickner, P Epstein, J Lemons, R Levins, E L Loechler, M Quinn, R Rudel, T Schettler, A M Stoto. The precautionary principle in environmental science. 2001, Environmental Health Perspectives, 871-876, 109, 9, https://ehp.niehs.nih.gov/doi/abs/10.1289/ehp.01109871

7 Rocha VC Figueiredo, Souza-Filho AF, Ikuta CY, et al. High discrimination of Mycobacterium bovis isolates in Brazilian herds by spoligotyping. **Preventive Veterinary Medicine**, v.179, p.104976, 2020. Doi: 10.1016/j.prevetmed.2020.104976.

8 Taye H, Alemu K, Mihret A, Wood JLN, Shkedy Z, Berg S, Aseffa A. Global prevalence of Mycobacterium bovis infections among human tuberculosis cases: Systematic review and meta-analysis. Zoonoses Public Health. 2021 Nov;68(7):704-718. doi: 10.1111/zph.12868. Epub 2021 Jun 24. PMID: 34169644; PMCID: PMC8487997.

9 Laine CG, Johnson VE, Scott H, Arenas-Gamboa AM. Global Estimate of Human Brucellosis Incidence. Emerg Infect Dis. 2023;29(9):1789-1797. https://doi.org/10.3201/eid2909.230052

10 Ansari, S., Heitzig, J., Brzoska, L., Lentz, H. H., Mihatsch, J., Fritzemeier, J., & Moosavi, M. R. (2021). A Temporal Network Model for Livestock Trade Systems. Frontiers in Veterinary Science, 8. https://doi.org/10.3389/fvets.2021.766547

11 Loria GR, Migliore S, Bongiorno C, Ciaccio G and Laddomada A (2022) New European rules introduced by regulation (EU) 2016/429 to facilitate animal trade: With great risk comes great responsibility. Front. Vet. Sci. 9:1003732. https://doi.org/10.3389/fvets.2022.1003732

12 Porphyre, T., Boden, L. A., Correia-Gomes, C., Auty, H. K., Gunn, G. J., & J. Woolhouse, M. E. (2016). Using national movement databases to help inform responses to swine disease outbreaks in Scotland: The impact of uncertainty around incursion time. Scientific Reports, 6. https://doi.org/10.1038/srep20258

13 Regulation (EU) 2016/429 of the European Parliament and of the Council of 9 March 2016 on transmissible animal diseases and amending and repealing certain acts in the area of animal health ('Animal Health Law') (Text with EEA relevance). Off J Eur Communities. (2016) L84:1–208

14 ORDINANZA 9 agosto 2012 Misure straordinarie di polizia veterinaria in materia di tubercolosi, brucellosi bovina e bufalina, brucellosi ovi-caprina, leucosi, nelle regioni Calabria, Campania, Puglia e Sicilia. (12A09729) (GU Serie Generale n.212 del 11-09-2012) https://www.gazzettaufficiale.it/eli/id/2012/09/11/12A09729/sg (visitato il 25/08/2023)

15 ORDINANZA 28 maggio 2015, Misure straordinarie di polizia veterinaria in materia di tubercolosi, brucellosi bovina e bufalina, brucellosi ovi-caprina, leucosi bovina enzootica. (15A04879) (GU n.144 del 24-6-2015) and following amendments and additions

16 2003/24/EC: Commission Decision of 30 December 2002 concerning the development of an integrated computerised veterinary system (notified under document number C(2002) 5496)

17 EU, 2019. Commission Implementing Regulation (EU) 2019/1715 of 30 September 2019 laying down rules for the functioning of the information management system for official controls and its system components (the IMSOC Regulation) OJ L 261, 14.10.2019

18 Mardones, F., zu Donha, H., Thunes, C., Velez, V., & Carpenter, T. (2013). The value of animal movement tracing: A case study simulating the spread and control of foot-and-mouth disease in California. Preventive Veterinary Medicine, 110(2), 133-138. https://doi.org/10.1016/j.prevetmed.2012.11.024

19 Judite Monteiro Braga et al. Investigation and contact tracing of the first cases of COVID-19 in Mozambique, 2020. Pan African Medical Journal. 2022;41:302. https://doi.org/10.11604/pamj.2022.41.302.26979

20 Kölzsch, A., Davidson, S.C., Gauggel, D. et al. MoveApps: a serverless no-code analysis platform for animal tracking data. Mov Ecol 10, 30 (2022). https://doi.org/10.1186/s40462-022-00327-4

21 Bingham, P., Wada, M., van Andel, M., McFadden, A., Sanson, R., & Stevenson, M. (2020). Real-Time Standard Analysis of Disease Investigation (SADI)—A Toolbox Approach to Inform Disease Outbreak Response. Frontiers in Veterinary Science, 7, 563140. https://doi.org/10.3389/fvets.2020.563140

22 Hang, C.-N.; Tsai, Y.-Z.; Yu, P.-D.; Chen, J.; Tan, C.-W. Privacy-Enhancing Digital Contact Tracing with Machine Learning for Pandemic Response: A Comprehensive Review. Big Data Cogn. Comput. 2023, 7, 108. https://doi.org/10.3390/bdcc7020108

23 Anglemyer A, Moore TH, Parker L, Chambers T, Grady A, Chiu K, Parry M, Wilczynska M, Flemyng E, Bero L. Digital contact tracing technologies in epidemics: a rapid review. Cochrane Database Syst Rev. 2020 Aug 18;8(8):CD013699. doi: 10.1002/14651858.CD013699. PMID: 33502000; PMCID: PMC8241885.
24 Savini, L., Candeloro, L., Perticara, S., & Conte, A. (2019). EpiExploreR: A Shiny Web Application for the Analysis of Animal Disease Data. Microorganisms, 7.SIMAN. Available online: https://www.vetinfo.sanita.it/j6_siman/.
25 Sistema Informativo Veterinario. Available online: https://www.vetinfo.sanita.it/sso_portale/accesso.pl .
26 Decreto 30 maggio 2017. Istituzione di un Centro di referenza nazionale nel settore veterinario. G.U. n 196 del 26 Agosto, 2017
27 Di Lorenzo A, Mangone I, Colangeli P, Cioci D, Curini V, Vincifori G, Mercante MT, Di Pasquale A, Iannetti S. One health system supporting surveillance during COVID-19 epidemic in Abruzzo region, southern Italy. One Health. 2023 Jun;16:100471. https://doi.org/10.1016/j.onehlt.2022.100471. Epub 2022 Dec 7. PMID: 36507072; PMCID: PMC9726647.
28 Diestel, R. 2010. Graph theory. Graduate Texts in Mathematics, Vol. 173, Fourth Edition, Springer-Verlag,866 Heidelberg.
29 Blonder, B., Wey, T.W., Dornhaus, A., James, R. and Sih, A. (2012), Temporal dynamics and network analysis. Methods in Ecology and Evolution, 3: 958-972. https://doi.org/10.1111/j.2041-210X.2012.00236.x
30 Chan J, Holmes A, Rabadan R. Network analysis of global influenza spread. PLoS Comput Biol. 2010 Nov 18;6(11):e1001005. doi: 10.1371/journal.pcbi.1001005. PMID: 21124942; PMCID: PMC2987833
31 Chris Rorres, Maria Romano, Jennifer A. Miller, Jana M. Mossey, Tony H. Grubesic, David E. Zellner, Gary Smith, Contact tracing for the control of infectious disease epidemics: Chronic Wasting Disease in deer farms, Epidemics, Volume 23, 2018, Pages 71-75, ISSN 1755-4365. https://doi.org/10.1016/j.epidem.2017.12.006.
32 Natale F, Savini L, Giovannini A, Calistri P, Candeloro L, Fiore G. Evaluation of risk and vulnerability using a Disease Flow Centrality measure in dynamic cattle trade networks. Prev Vet Med. 2011 Feb 1;98(2-3):111-8. doi: 10.1016/j.prevetmed.2010.11.013. Epub 2010 Dec 15. PMID: 21159393.
33 Calistri P, Conte A, Natale F, Possenti L, Savini L, Danzetta ML, Iannetti S, Giovannini A. Systems for prevention and control of epidemic emergencies. Vet Ital. 2013 Jul-Sep;49(3):255-61. doi: 10.12834/VetIt.1206.06. http://www.izs.it/vet_italiana/pdf/VetIt_1206_06.pdf
34 Iannetti S, Savini L, Palma D, Calistri P, Natale F, Di Lorenzo A, Cerella A, Giovannini A. An integrated web system to support veterinary activities in Italy for the management of information in epidemic emergencies. Prev Vet Med. 2014 Mar 1;113(4):407-16. doi: 10.1016/j.prevetmed.2014.01.015. Epub 2014 Jan 22. PMID: 24485707.
35 Di Lorenzo A., Savini L., Candeloro L., Tora S., Cerella A., Di Sabatino D., Conte A. "Web-GIS and livestock trace tools for epidemiological surveillance, control and management" Front. Vet. Sci. doi: 10.3389/conf.fvets.2019.05.0004
36 Tegegne, H. A., Bogaardt, C., Collineau, L., Cazeau, G., Lailler, R., Reinhardt, J., Freeth, F. T., Taylor, E., Prada, J. M., & Hénaux, V. (2023). OH-EpiCap: A semi-quantitative tool for the evaluation of One Health epidemiological surveillance capacities and capabilities. Frontiers in Public Health, 11, 1053986. https://doi.org/10.3389/fpubh.2023.1053986
37 Jonathan A. Polonsky, Amrish Baidjoe, Zhian N. Kamvar, Anne Cori, Kara Durski, W. John Edmunds, Rosalind M. Eggo, Sebastian Funk, Laurent Kaiser, Patrick Keating, Olivier le Polain de Waroux, Michael Marks, Paula Moraga, Oliver Morgan, Pierre Nouvellet, Ruwan Ratnayake, Chrissy H. Roberts, Jimmy Whitworth and Thibaut Jombart. Outbreak analytics: a developing data science for informing the response to emerging pathogens. Philosophical Transactions of the Royal Society B: Biological Sciences, Volume 374, Issue 1776. https://doi.org/10.1098/rstb.2018.0276
38 Moriyón, I., Blasco, J. M., Letesson, J. J., De Massis, F., & Moreno, E. Brucellosis and One Health: Inherited and Future Challenges. Microorganisms, 11(8), 2070. https://doi.org/10.3390/microorganisms11082070
39 Charlier, J., Barkema, H. W., Becher, P., De Benedictis, P., Hansson, I., Hennig-Pauka, I., La Ragione, R., Larsen, L. E., Madoroba, E., Maes, D., Marín, C. M., Mutinelli, F., Nisbet, A. J., Podgórska, K., Vercruysse, J., Vitale, F., Williams, D. J. L., & Zadoks, R. N. (2022). Disease control tools to secure animal and public health in a densely populated world. The Lancet Planetary Health, 6(10), e812-e824. https://doi.org/10.1016/S2542-5196(22)00147-4

Article

T-Racing: a modern tool for supporting epidemiological investigation in animal disease outbreaks

Luca Candeloro [1], Lara Savini [1~~*~~], Annamaria Conte [1], Paolo Calistri [1], Diana Palma [1], Samuel Perticara [1], Anna Cecília Trolesi Reis Borges Costa [2*], Alessio di Lorenzo [1], Susanna Tora [1], Sara Serrani [1], Michela Toro [1], Karina Diniz Baumgarten [2, 3] and Fabrizio De Massis [1]

[1] Istituto Zooprofilattico Sperimentale dell'Abruzzo e del Molise "G.Caporale", Italy
[2] Departamento de Medicina Veterinária, Escola de Zootecnia e Medicina Veterinária, Universidade Federal de Lavras, Lavras Minas Gerais, Brazil.
[3] State Department of Animal Health. Companhia Integrada de Desenvolvimento Agrícola de Santa Catarina – CIDASC, Florianópolis, SC, Brazil

[*] Correspondence: anninha_veterinaria@hotmail.com

Abstract: The One Health approach unites efforts across human-animal-environment interfaces against shared threats like zoonotic diseases. T-Racing~~,~~ is a ~~modern~~Shiny web ~~tool, helps~~application, that supports epidemiological investigations and ~~lets~~helps contain livestock-related disease spread, aligning with One Health principles ~~to safeguard~~ of safeguarding public health. The application uses Temporal Network Analysis techniques to ~~account for~~address the dynamic nature of animal trade~~ and to perform~~, facilitating ~~quick and efficient~~ backward and forward tracing ~~procedures~~strategies. Therefore~~Indeed~~, T-Racing leverages web services to retrieve data from multiple sources simultaneously and ~~near~~ almost in real-time ~~sources~~ through the plumber package and ~~has been~~ is distributed using Shinyproxy. Indeed, T-Racing manages and ~~analyses~~analysizes extensive and diverse datasets within the same environment, including animal movement data~~,~~ ~~livestock owners' and producers' information~~, disease outbreak data, and genomic data, all obtained from Italian National databases ~~within the same environment~~. In this work, we show T-Racing's capabilities by simulating epidemiological investigations of ~~some~~ b~~B~~rucellosis and t~~T~~uberculosis outbreaks that occurred ~~in Italy~~ in non–endemic areas of Italy~~, in Italy~~. This tool ~~greatly~~ supports epidemiological investigations~~, making easier and~~ by adopting a data-driven approach, guiding ~~user~~users through the analysis via an iterative process while leveraging their expertise. Therefore, it ~~It~~ enables faster data analysis ~~and enabling~~, improves ~~a comprehensive~~ understanding of disease transmission patterns, and facilitates~~ing~~ prompt and targeted interventions.

Keywords: One Health; Epidemiology; Tracing; Temporal Network Analysis; Shiny web application; Outbreak investigation; Zoonoses management.

1. Introduction

Preventing and controlling communicable diseases in livestock, through ~~outbreak~~ early detection of outbreaks, increases the effectiveness of the emergency response ~~to them,~~ and ~~reduce~~reduces the social, economic, and environmental costs associated with ~~the outbreak~~these events [1, 2]. ~~Moreover, animals~~Animals serve as an excellent ~~channel~~proxy for monitoring ~~both~~ novel and known zoonotic pathogens ~~with outbreak potential, as~~given that more than 60% of emerging infectious diseases in humans originate ~~from zoonotic~~ from animal sources [3-5]. –This underscores ~~emphasizes~~ the critical role of animal health in ~~discovering~~the implementation of the One Health approach, by detecting, monitoring, and predicting health hazards that could also affect environmental

and human health, since some pathogens can survive long periods in the environment ~~hazards~~ [6]. Among the diseases that could affect human health are~~,~~ tuberculosis and brucellosis, ~~are~~ both chronic infectious diseases caused by bacteria. Bovine tuberculosis, caused by *Mycobacterium bovis* [7], is a zoonotic ~~tuberculosis~~ disease that ~~and~~ was estimated to have a human prevalence varying from 0.4% to 76.7% worldwide [8]. On the other hand, animal brucellosis is caused by *Brucella* spp., being a zoonosis of utmost significance, since it was projected to globally affect ~~by the year~~ 2.1 million people by year [9]~~globally~~. The control of both diseases is anchored on the surveillance and control in ~~a One-health approach.~~animals, with the traceability of animal cases being of paramount importance to prevent occurrences in humans, especially for human brucellosis that does not have vaccination. In this context, ~~the~~ the movement of animals is often considered a major risk for disease spreading between herds [~~7~~10], and to humans when ~~zoonosis~~ zoonoses are involved. ~~Consequently~~Hence, livestock ~~transport~~transportation data ~~is~~is~~are~~ registered in national and international databases to ~~enable~~ facilitate contact tracing in the event of an outbreak [~~8~~11-~~12~~15]. ~~Another~~An example of an international official movements database is TRACES-NT (TRAde Control and Expert System New Technology)~~,~~. TRACES-NT is an information platform of the European ~~Union~~Commission for the reporting, certification, and control of imports of animals, ~~products~~origin of animal products, ~~origin,~~ food and feed of plants, ~~origin~~ and other products of veterinary interest ~~into the EU, as well as for intra-EU~~. The system also registers trade and exports ~~from the EU~~ of animals and certain products of animal origin~~.~~ into the EU and intra-EU. Currently, TRACES-NT is the integrated ~~computerised~~computerized veterinary system with a single architecture provided ~~for~~ in Decisions 2003/24/EC and 2004/292/EC (EU, 2019) [~~13~~16, ~~14~~17].

Consequently, the usage of the animal movement systems facilitates t~~T~~racing the spread of diseases within animal populations and ~~, and~~ the~~racing~~ potential routes of exposure ~~and spread is~~ being critical for effective disease~~s~~ control [~~15~~18]~~,~~ and ~~is~~ crucial in the final steps of ~~an~~ eradication ~~programme~~programs. Contact tracing plays a pivotal role in identifying the necessary actions ~~to be taken~~ to break the transmission chain, ~~and~~ to reduce the spread and duration of infectious diseases, thus preventing new cases [~~16~~19]. When zoonoses are considered, this also means ~~also~~ protecting public health, once ~~.~~ c~~C~~ontact tracing has the subsequent effect of providing assessments to stakeholders ~~that~~which ~~are~~ is useful for quickly identifying and implementing containment, surveillance, and control systems. In this sense, t~~T~~echnology is fundamental to~~in~~ build~~ing~~ online tracing applications, ~~plays a pivotal role in~~ that can improve~~enhancing~~ the ~~outcome~~results of epidemiological investigations. Advanced analytics and link analysis can be used to uncover~~may help in uncovering~~ hidden patterns in the spread of diseases ~~of disease spreading~~ [~~18~~20, 2]. Therewith, tracing applications have been developed to assist animal health officials in quickly identifying the animals involved in an outbreak, where and when the infected animals are located, and whether other susceptible animals might have been exposed to the disease. These actions would be taken by uncovering the missing or unexpected links from the contact details, identifying the animals that should undergo testing, tracking the geographical spread of the disease, and determining which farms are at the highest risk. Thus, tracing application can help in disease control and surveillance during critical outbreak stages~~,~~. ~~This can save~~ saving~~es~~ time, ~~help in preventing~~ prevent~~ings~~ further spreading, ~~thereby~~ minimizing~~inge~~ ~~the~~ costs and reducing~~es~~ the consequences of the disease outbreaks [~~17~~21, 2]. Tracing applications ~~It~~ enables~~.~~ ~~This could be an invaluable tool that allows~~ veterinary services, epidemiologists, and competent authorities to respond quickly in identifying the "hot spots" by~~and in~~ implementing effective~~ and~~ targeted control and eradication measures.

~~Technology plays a pivotal role in enhancing the outcome of epidemiological investigations. Advanced analytics and link analysis may help in uncovering hidden patterns of disease spreading [18, 2].~~

Yet, contact tracing, Aalthough it may sound simple,ing straightforward, the tracing application can be coame overwhelmingly complex and resource-intensive,. hHowever, using data visualization and analytics can significantly improve theits performance and effectiveness, making the applicationit . However, efforts in contact-tracing can be significantly improved by using data visualization and analytics allowing public health officials to uncover insights from contact-tracing efforts and all available health data to understand:an invaluable tool.

Currently, This could be an invaluable tool that allows veterinary services, epidemiologists, and competent authorities to respond quickly in identifying the “hot spots” and in implementing effective control and eradication measures. In responsedue to the COVID-19 global pandemic, contact tracing strategies have been widely used in the last three years and .manyVarious contact-tracing web apps have been developed for this purpose in human health [22, 23](Ching-Nam Hang et al., 2023; Anglemyer et al 2020). In veterinary epidemiology, there areis limited literature on specific contact tracingCT web applications, such as. Some examples in the veterinary field include EpiContactTrace [2], which is an open-source tool implemented in R for contact tracing during disease outbreaks, and EpiExploreR, a Shiny web application that integrates disease mapping and spatiotemporal analysis tools [24](Savini et al. 2019). HoweverEven though there are tracing applications already developed in the veterinary field, there isareis still a need for a web tool that can manage various and heterogeneous datasets and incorporate analytical features to quickly perform forward and backward tracing within a spatial-temporal environment. Therefore, thehis article aim of this paper wass to introduce T-Racing, a web-based operational tool that has been created to assist veterinary epidemiological investigations in Italy. Based on our knowledge, T-Racing has several distinctive features, including its ability to integrate diverse data sources, its user-friendly interface, and its scalability. This tool is designed specifically to facilitate veterinary public health institutes, services, epidemiologists, veterinary professionals, and competent authorities explore and analyze the complex spatiotemporal pathways that shape disease spread. It enables users to draw meaningful conclusions based on a data-driven, user-guided approach.an application providing users with flexibility and facilitating usage of methods and tools through an interacting interface in an environment surroundedcomposed by epidemiologically useful data. this article we present T-Racing, a user-friendly web application developed in an R environment to support epidemiological investigations through interacting maps, graphs, and tables.

2. Materials and Methods

T-Racing is an application providing users with flexibility and facilitating usage of methods and tools through an interacting interface in an environment composed by epidemiologically useful data. a web-based operational tool that has been created to assist designed with the aim of providing an efficient veterinary epidemiological investigations in Italy. and simpleThis tool is specifically designed to allow veterinary public health institutes, services, epidemiologists, veterinary professionals, and competent authorities, to explore and analyseanalyze thefor analysing complex spatiotemporal pathways that shape disease spread and

combine multiple epidemiological datasets within one platform.

2.1 Data sources and collection

Along with animal movementT-Racing gathers near-real-time data from Italian National Databases which includeincluding livestock owners’ and producers’ information, disease notified outbreaks dataobtained from the National Information System for the Notification and Management of Animal Diseases (SIMAN) [1925], animal movements records extracted from the Italian National Database for Animal Identification

and Registration (NDB) [2~~0~~6], and ~~also g~~Genomic data, such as~~like~~ Multi Loci Sequence Type (MLST), ~~have been included in the application.~~

~~Animal movements and livestock owners and producers' data, outbreak disease data, and Genomic data are~~ provided by ~~the Italian:~~

~~1. National Database for Animal Registration (BDN, www.vetinfo.sanita.it),~~

~~2. National Animal Disease Notification System (SIMAN, www.vetinfo.sanita.it), and~~

National Reference Centre for Whole Genome Sequencing of microbial pathogens database and bioinformatics analysis' (~~GENPAT~~GenPat) [~~19~~2~~1~~7, ~~20~~2~~2~~8]. These systems can be accessed through~~are~~ the VETINFO portal (https://www.vetinfo.sanita.it/), which is a secure platform to collect and present data for managing Italy's Animal Health and Food Safety system. VETINFO provides unified access to various stakeholders, including public health institutes, veterinary services, farmers, and competent authorities. To ensure user trust and regulatory compliance, the portal strictly adheres to GDPR compliance techniques, requiring users to access it with personal credentials and mandating specific functionalities for transparent and secure data reporting. The management of these databases is the responsibility of the ~~managed by~~ Istituto Zooprofilattico Sperimentale dell'Abruzzo e del Molise "G. Caporale" (IZS-Teramo) on behalf of the Italian Ministry of Health.

T-Racing collects detailed data for each reported outbreak, including the disease name, outbreak code, geographical coordinates, region, province, administrative unit, ~~the outbreak~~ suspected date, outbreak ~~and, the outbreak~~ state (i.e., suspect, extinct, and confirmed), and species involved (including animal, vector species such as mosquitoes and flies, and birds, etc.). Animal movement data includes the origin and destination holding codes, animal single identification codes or ~~batch~~batches of traded animals per species (including horses, cattle, buffalo, pigs, and sheep and goats), and the date of the movement. The system also considers various holding attributes (livestock properties), such as the holding type (e.g., farm, slaughterhouse, staging point, transit station, market, assembly center, foreign country, etc.), production type (e.g., milk, meat, wool, reproduction, etc.), address, and geographical coordinates. Additionally, in terms of genomic data, T-Racing records the holding code, animal single identification code, Sequence Type number, and date of sampling. Furthermore, T-Racing relies on data collected over the past 7 years extracted from the databases through daily scheduled procedures.

~~To ensure data accuracy and consistency, rigorous data collection, processing, and control rules have been implemented for each of these systems. These rules are continuously updated and adapted to comply with national and European laws and regulations, reflecting a commitment to maintaining the accuracy, relevance, and reliability of the data.~~

2.2 Temporal Network Analysis methods in T-Racing

The application uses ~~relies on~~ Temporal Network Analysis techniques [29] ~~to address and accounts for~~ the dynamic nature of animal trade, enabling quick and efficient backward (BCT) and forward (FCT) tracing strategies. ~~to facilitate rapid and efficient back and forward tracing processes~~ In the T-Racing environment, the animal trading network is represented as a time-ordered directed and weighted network, where holdings are the nodes, animal movements between holdings on a given day are the edges, and the number of moved animals is the weight (see Fig 1 on the left).

Animal movements are recorded in the NDB at the administrative farm owner (farmer) level. Consequently, multiple farmers from the same holding may be represented as different nodes (see Fig 1, top right), causing the network to be artificially fragmented. This fragmentation significantly impacts the analysis of diseases affecting multiple animal species. To address this issue, the application allows for network aggregation, ensuring

that each node represents the physical holding, i.e. the geographical location to where the animals transited. Thus, a node can represent multiple owners at a fiscal level (see Fig 1, bottom right).

The framework of time-ordered networks, essentially directed acyclic graphs [29], is useful for understanding flow dynamics [30], particularly in the context of diseases spread through direct contact. We assume diseases spread along paths driven by animal movements, so accounting for the direction and timing of these movements allows us to detect temporally valid paths, providing a likely estimation of disease spread, similar to an SI (Susceptible-Infection) network-based compartmental model (where the contact rate is one). Direction and the timing of animal movements are important for detecting disease spread because they highlight impossible paths, and or too-long time elapsed for short paths [30, 31]. For example, in Figure 1, if farm A is infected at time zero, it can infect holding B, which c**an then infect holding C through subsequent animal movements. Since the probability of disease transmission decreases with increasing degrees of separation, we typically limit the search for plausible paths to a maximum degree of separation, or "level depth." Depending on the disease, the time elapsed between events might be too long to assume** that nodes remain infectious and undetected, requiring a restriction of the time horizon to a specific number of days, known as "temporal depth." The choice of depth, in terms of both days and levels, affects the resulting subnetwork and depends on factors such as the disease's infectiousness, incubation period, and the timing of surveillance system controls. Furthermore, removal of node(s) and/or edge(s) from a time-ordered directed network involves the change of the resulting paths. Therefore, ~~–~~each network modification requires a recalculation of the paths.

Contact tracing following a positive detection has been widely used in various fields, with different interpretations and applications depending on the context [30, 32]. In T-Racing, FCT, BCT, and a combination of both (FCT-BCT) strategies have been implemented to support track and control the spread of diseases. These methods provide different perspectives and insights depending on the context and time~~ing~~ of disease outbreaks. **FCT method** focuses on identifying the pathways of pathogen spread starting from an index node, known as the seed, and an event date. By analyzing outgoing movements within a specified depth (in terms of days and levels)~~,~~ FCT highlights the nodes that the disease could have potentially reached. This approach supports targeted disease control more effectively than a general surveillance system, which, despite being timely conducted, can only prevent a portion of the potential disease spread (as illustrated in Fig 2). The control shown in Fig 2 can be considered a real-time monitoring and proactive control in response to a new outbreak detection. It is performed as soon as the new outbreak is notified to detect its outgoing movements in the recent past. The subnetwork found in a forward direction will help identify positive farms and prevent the spread of infection to other farms in the future. **BCT method**, on the other hand, aims to trace the primary source of infection from a newly detected outbreak. This retrospective strategy ~~consist~~consists in finding temporal valid pathways through incoming movements within specific time constraints and degrees of separation. Typically, BCT uses a broader time window compared to FCT, allowing for a comprehensive analysis of the infection's origins. Both methods rely on (require) updated data. Nowadays, current Italian legislation requires the registration of animal movements in NDB within 7 days~~)~~, but this does not always happen. More often, and especially for species regulated more recently, registration times are longer. This means that some of the real connections may be missing in NDB when performing FCT or BCT strategies, leading to undetected possible infectious nodes. **Combination of strategies (FCT-BCT)** involve using both methods to enhance disease control efforts. For instance, after performing a BCT starting

from multiple recent outbreaks within a large time window, the resulting subnetwork may include numerous nodes. If among these nodes there are outbreaks notified in the past, an FCT can be conducted on this newly identified BCT subnetwork. This intersection (BCT ∩ FCT) reveals the subnetwork connecting the outbreaks through temporally valid paths. Then, b~~B~~y working in the past, when~~re~~ data is more likely to be updated in NDB, this strategy identifies nodes that have been exposed to infected animals but have not yet tested positive. A comparison of animal movement dates with the last inspections on the farms can help promptly identify nodes for further inspection, aiding in containing the disease spread. In the end, such kind of strategies could be performed using batch of animals (and thus letting the paths highlighting the spread accounting for transmission between animals sharing same farms) or considering the individual animals moving forward or backward from a seed farm. These strategies can be applied either to batches of animals (highlighting the spread through shared farm contacts) or to individual animals moving between farms. The T-Racing application facilitates these analyses, enabling users to leverage epidemiological information and expert knowledge to transform vast amounts of data into clear, actionable guidelines for disease control.

~~**T-Racing has been developed using the Shiny library by the R statistical environment, and leaflet and visnetwork libraries were used for map and graph visualization respectively (in Table 1 are listed all of the packages used). The application lets manage large amounts of data from different information systems and share them efficiently and securely in real-time.**~~
~~**Indeed, computationally heavy services are executed on a remote server, differently from the serverless web apps, and the Plumber package let the application query data sources in real-time, through web API.**~~
~~**The system architecture (Figure 1) makes sure the application scalability, thanks to shiny proxy (a free and open-source Docker-based application).**~~

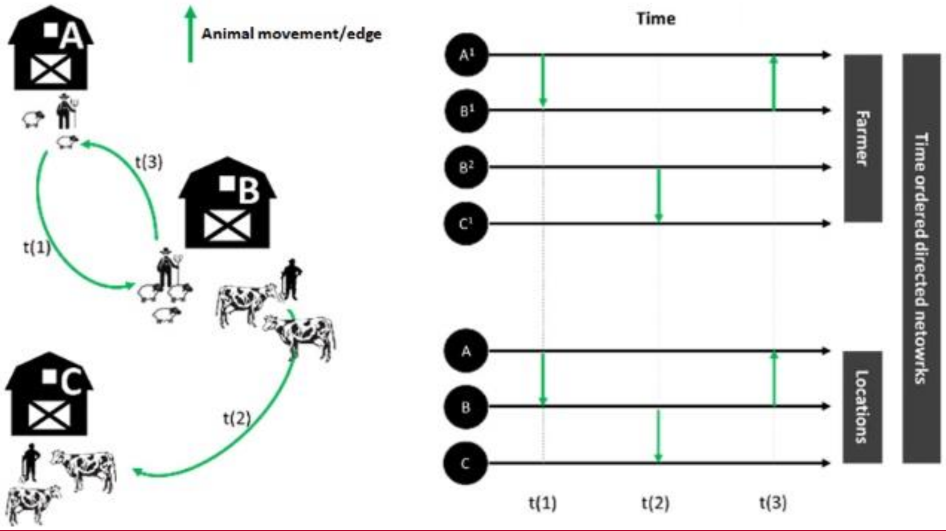

Fig~~ure~~ 1 Network fragmentation and aggregation in animal movement records. The top right panel shows how multiple farmers of the same holding are represented as different nodes, leading to artificial fragmentation. The bottom right panel demonstrates the aggregation process, where each node represents the physical holding, encompassing multiple owners at a fiscal level.

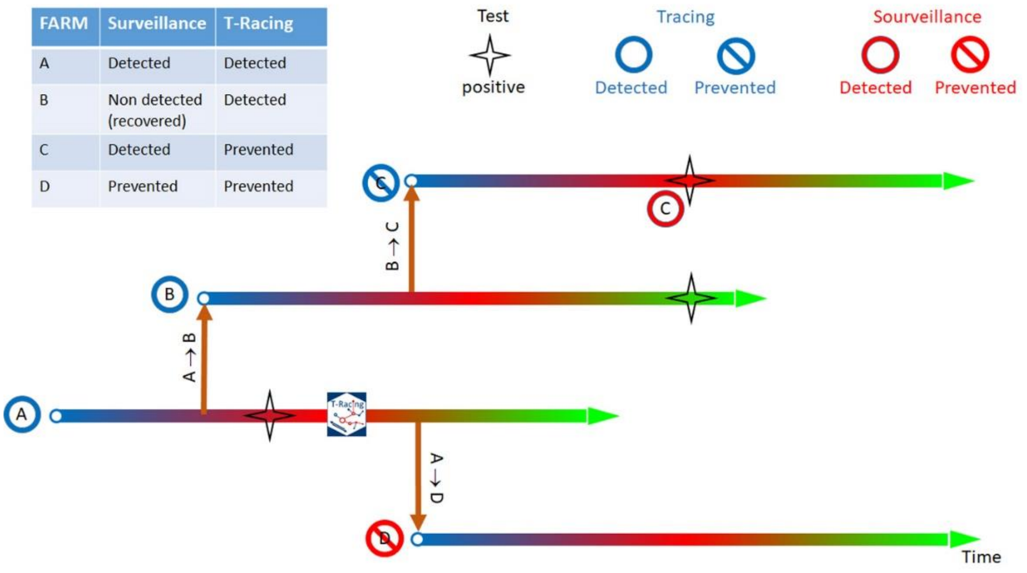

~~Figure 8~~2~~.~~ Schematic ~~I~~illustration of the importance of tracing in preventing and detecting diffusion ~~path ways~~pathways versus the simple farm isolation when only positivity recorded in the context of the surveillance program is considered.

2.3 T-Racing Design

T-Racing interface allows users to quickly query, filter, ~~analyse~~analyze, and ~~visualise~~visualize data through ~~The application makes use of an~~ interactive graph, map, and tables for a seamless and intuitive experience. Fig~~ure~~ 3 provides a conceptual map of the application, highlighting its primary dashboard sections and features.~~to improve the user experience.~~

–Tools for extracting and viewing animal trade data, including: ~~The control bar, displayed in the center of the dashboard, provides different tools to:~~

- ~~Filter~~ Filtering data based on multi-species selections, time frame~~period interval~~, tracing direction mode (backward or forward methods), network geo-aggregation mode, and ~~include~~including/~~or~~ excludeing animal trade data from~~ and~~ /to foreign countries or slaughterhouses;
- ~~Implement m~~Multi-seed selection~~, including those within a spatial buffer around a farm or outbreak. Moreover, T-Racing offers two methods for selecting the seeds~~ for tracing,~~: either~~ allowing users to either type or select ~~by choosing~~ one or more farm codes from the list of farms or ~~by selecting~~select ~~an~~ outbreak codes from the list of officially notified outbreaks within a specified time frame. Alternatively, the user can select all farms and/or outbreaks falling within a spatial buffer (neighbors distant by kilometers) around selected farms and outbreaks;
- Tracking a single animal by typing its identification code and viewing all resulting spatial-temporal paths within a specified time frame.~~;~~

The system retrieves movement data based on these filters from the NDB and builds a time-ordered directed weighted network originated by the set seeds. These tools are useful for ~~analysing~~analyzing diseases that are transmitted through direct contact (i.e. b~~B~~rucellosis or t~~T~~uberculosis) and diseases that are transmitted through spatial proximity (i.e. ~~African~~African s~~S~~wine f~~F~~ever or vector-borne diseases such as west Nile or Bluetongue, etc.), using spatial buffering and contact tracing methods.

Tools for customizing network visualization and optimization:

- C~~C~~ustomizeing ~~the~~ graph visualization by ~~choosing~~ setting ~~a~~ from a range of layout options~~: —~~ such as hierarchical, geographical, or nicely mode (default mode).

~~Additionally, the operator~~Users can also ~~thematise~~ make the shape and color of nodes or the color of edges reflect selected data attributes ~~edge/nodes by selecting attributes of data~~ such as ~~facility~~ holding type or production type ~~orientation~~ for nodes, and level, time, or~~,~~ species~~,~~ ~~or depth level~~ for edges. ~~;~~All possible attribute ~~colors~~colors and shapes are predefined in a configuration file.

- R~~R~~emoving~~e~~ unnecessary nodes or edges from performed tracing;
- Highlighting within the network all edges involving animals moved~~ing~~ from/to the seed, or reducing the network to only those edges involving animals moved~~ing~~ from/to the seed, according to the tracing direction mode set by the user;
- Reducing the network size by decreasing network deepness (in days or levels).

For each network reduction method applied, a new time-ordered network will be created to ensure and maintain the temporal coherence in the events.

Tools for epidemiological information integration and results printing:

- Adding a layer of sequenced pathogens for the animals;
- ~~or outbreaks data engaged in a specific disease epidemiological investigation;~~Adding a layer of outbreaks for a specific disease or reducing the resulting network by keeping only temporally valid paths among involved outbreaks by a combination of FCT and BCT methods;
- Download the network's tables (or a selection of them) in Excel format;
- Stor~~e~~ing the results in a dynamic HTML report for offline use.

By using these tools, the network graph can be customized and fine-tuned to provide a more meaningful representation of the data.

The T-Racing application was created by a team of experts including epidemiologists, veterinarians, mathematicians, and computer scientists. This collaborative effort spanned all stages of development, from gathering data to creating tools and building a user-friendly interface. The team has extensive experience in epidemiological tracing and dynamic network analysis, as evidenced by similar web applications they have developed since 2011 [24, 33, 34, 35, 36]. T-Racing is an improved version of previous applications, offering faster speeds, additional features, and integration of various epidemiological datasets. Currently, it is available on the internal network of IZS-Teramo and used by our veterinarians and epidemiologists. It is pending technical adjustments and official approval from the Italian Ministry of Health to be integrated into the VETINFO portal. This integration will ensure secure access and the protection of sensitive data, as T-Racing inherits the robust GDPR techniques already applied in the portal.

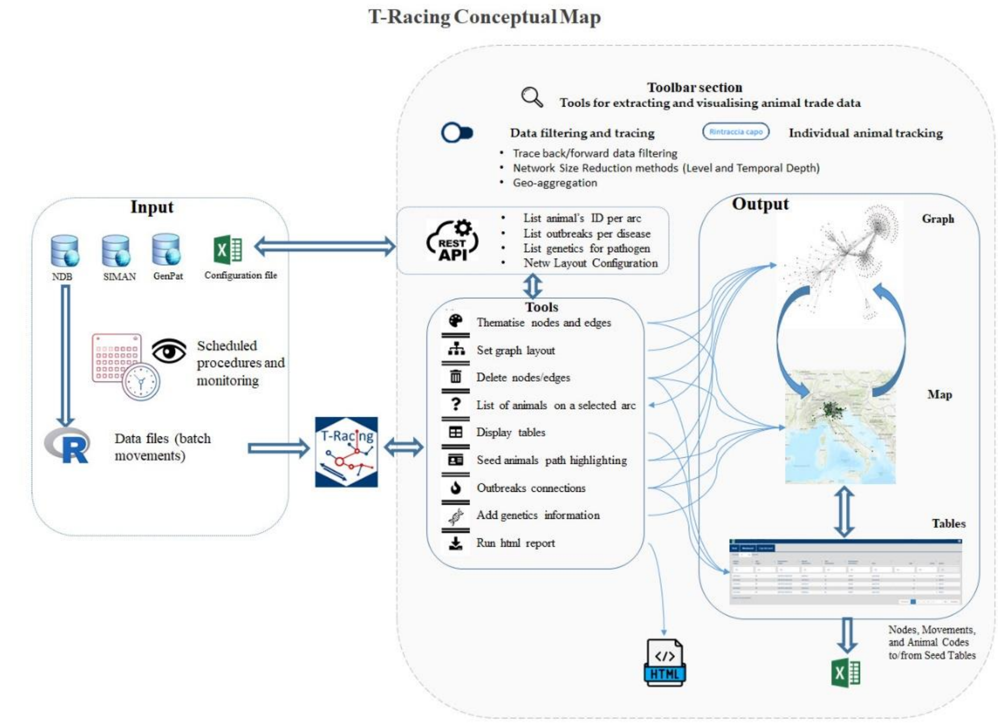

Figure 3. T-Racing conceptual map.

2.4 Data flow and system architecture

T-Racing has been developed using the Shiny library by the R statistical environment, and leaflet and visnetwork libraries were used for map and graph visualization respectively (in Table 1 contains are listed all of all the packages used). The application lets manages large amounts of data from different information systems and share them efficiently and securely in real-time. Differently from the serverless javascriptJavaScript web apps, computationally heavy services are executed on a remote server, and the Plumber package letallows the application to query data sources in real-time, through web API.

The system architecture (Figure 1) makes surefacilitates the application scalability, thanks to shiny proxy (a free and open-source Docker-based application). The described stack and the involved application modules are displayed in Figure 24.

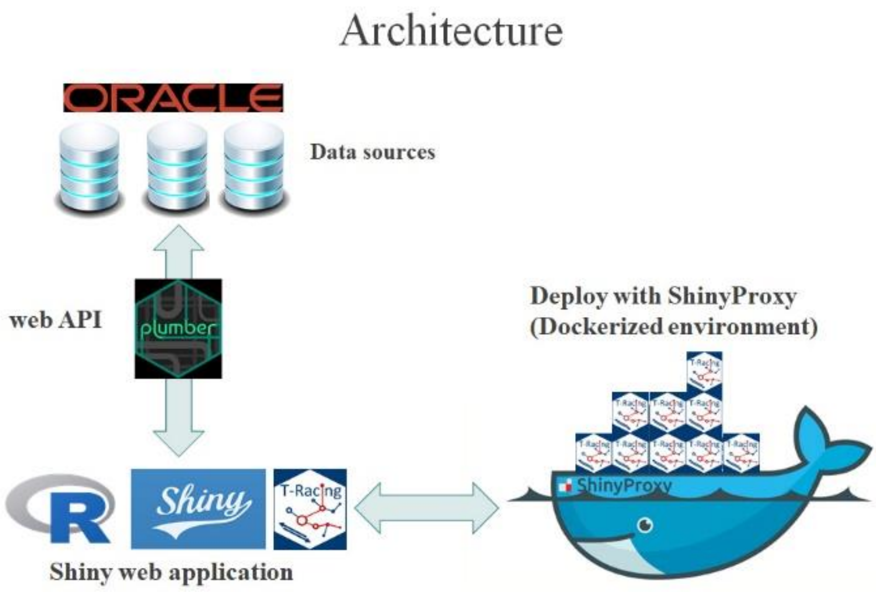

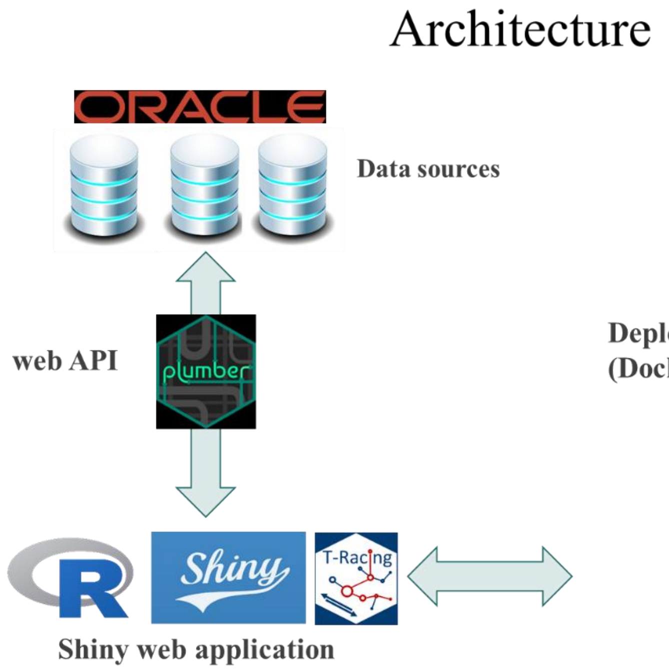

Figure 14. T-Racing architecture: T-Racing makes use of web services to retrieve data from databases, through the plumber package, and has been distributed using Shinyproxy.

Table 21. List of the R-packages used in T-Racing developingdevelopment.

R-package	Use	Reference
shiny (v. 1.7.1)Shiny	UI/SERVER	Winston Chang, Joe Cheng, JJ Allaire, Carson Sievert, Barret Schloerke, Yihui Xie, Jeff Allen, Jonathan McPherson, Alan Dipert and Barbara Borges (2021)
shinyjs (v. 2.1.0)shinyjs	UI/SERVER	Dean Attali (2020). shinyjs: Easily Improve the User Experience of Your Shiny Apps in Seconds
shinyWidgets (v. 0.6.4)shinyWidgets	UI	Victor Perrier, Fanny Meyer and David Granjon (2021). shinyWidgets: Custom Inputs Widgets for Shiny
Shinydashboard (v. 0.7.2)shinydashboard	UI	Winston Chang and Barbara Borges Ribeiro (2018). shinydashboard: Create Dashboards with 'Shiny'

shinyBS (v. 0.61.1)~~shinyBS~~	UI	Eric Bailey (2015). shinyBS: Twitter Bootstrap Components for Shiny
shinybusy (v. 0.3.0)~~shinybusy~~	UI/SERVER	Fanny Meyer and Victor Perrier (2020). shinybusy: Busy Indicator for 'Shiny' Applications
DT (v. 0.22)~~DT~~	UI	Yihui Xie, Joe Cheng and Xianying Tan (2021). DT: A Wrapper of the JavaScript Library 'DataTables'
rintrojs (v. 0.3.0)~~rintrojs~~	UI/SERVER	Carl Ganz (2016). rintrojs: A Wrapper for the Intro.js Library. Journal of Open Source Software, 1(6), October 2016
shinycssloaders (v. 1.0.0)~~shinycssloaders~~	UI/SERVER	Andras Sali and Dean Attali (2020). shinycssloaders: Add Loading Animations to a 'shiny' Output While It's Recalculating
htmltools (v. 0.5.2)~~htmltools~~	UI	Joe Cheng, Carson Sievert, Barret Schloerke, Winston Chang, Yihui Xie and Jeff Allen (2021). htmltools: Tools for HTML
leaflet (v. 2.1.1)~~leaflet~~	UI	Joe Cheng, Bhaskar Karambelkar and Yihui Xie (2022). leaflet: Create Interactive Web Maps with the JavaScript 'Leaflet' Library
rgdal (v. 1.5.31)~~rgdal~~	SERVER	Roger Bivand, Tim Keitt and Barry Rowlingson (2021). rgdal: Bindings for the 'Geospatial' Data Abstraction Library
sp (v. 1.4.7)~~sp~~	SERVER	Pebesma, E.J., R.S. Bivand, 2005. Classes and methods for spatial data in R. R News 5 (2)
bezier (v. 1.1.2)~~bezier~~	SERVER	Aaron Olsen (2018). bezier: Toolkit for Bezier Curves and Splines
geosphere (v. 1.5.14)~~geosphere~~	SERVER	Robert J. Hijmans (2021). geosphere: Spherical Trigonometry
visNetwork (v. 2.1.0)~~visNetwork~~	UI	Almende B.V., Benoit Thieurmel and Titouan Robert (2019). visNetwork: Network Visualization using 'vis.js' Library
igraph (v. 1.3.1)~~igraph~~	SERVER	Csardi G, Nepusz T: The igraph software package for complex network research, InterJournal, Complex Systems 1695. 2006
ggplot2 (v. 3.3.6)~~ggplot2~~	SERVER	H. Wickham. ggplot2: Elegant Graphics for Data Analysis. Springer-Verlag New York, 2016
RColorBrewer (v. 1.1.3)~~RColorBrewer~~	SERVER	Erich Neuwirth (2014). RColorBrewer: ColorBrewer Palettes
xlsx (v. 0.6.5)~~xlsx~~	SERVER	Adrian Dragulescu and Cole Arendt (2020). xlsx: Read, Write, Format Excel 2007 and Excel 97/2000/XP/2003 Files
dplyr (v. 1.0.9)~~dplyr~~	SERVER	Hadley Wickham, Romain François, Lionel Henry and Kirill Müller (2021). dplyr: A Grammar of Data Manipulation
jsonlite (v. 1.8.0)~~jsonlite~~	SERVER	Jeroen Ooms (2014). The jsonlite Package: A Practical and Consistent Mapping Between JSON Data and R Objects. arXiv:1403.2805
curl (v. 4.3.2)	SERVER	Jeroen Ooms, Hadley Wickham, RStudio
rJava (v. 1.0.6)	UI	

R-package	Use	Reference
Shiny	UI/SERVER	Winston Chang, Joe Cheng, JJ Allaire, Carson Sievert, Barret Schloerke, Yihui Xie, Jeff Allen, Jonathan McPherson, Alan Dipert and Barbara Borges (2021)
shinyjs	UI/SERVER	Dean Attali (2020). shinyjs: Easily Improve the User Experience of Your Shiny Apps in Seconds
shinyWidgets	UI	Victor Perrier, Fanny Meyer and David Granjon (2021). shinyWidgets: Custom Inputs Widgets for Shiny
shinydashboard	UI	Winston Chang and Barbara Borges Ribeiro (2018). shinydashboard: Create Dashboards with 'Shiny'
shinyBS	UI	Eric Bailey (2015). shinyBS: Twitter Bootstrap Components for Shiny
shinybusy	UI/SERVER	Fanny Meyer and Victor Perrier (2020). shinybusy: Busy Indicator for 'Shiny' Applications
DT	UI	Yihui Xie, Joe Cheng and Xianying Tan (2021). DT: A Wrapper of the JavaScript Library 'DataTables'
rintrojs	UI/SERVER	Carl Ganz (2016). rintrojs: A Wrapper for the Intro.js Library. Journal of Open Source Software, 1(6), October 2016
shinycssloaders	UI/SERVER	Andras Sali and Dean Attali (2020). shinycssloaders: Add Loading Animations to a 'shiny' Output While It's Recalculating
htmltools	UI	Joe Cheng, Carson Sievert, Barret Schloerke, Winston Chang, Yihui Xie and Jeff Allen (2021). htmltools: Tools for HTML
leaflet	UI	Joe Cheng, Bhaskar Karambelkar and Yihui Xie (2022). leaflet: Create Interactive Web Maps with the JavaScript 'Leaflet' Library
rgdal	SERVER	Roger Bivand, Tim Keitt and Barry Rowlingson (2021). rgdal: Bindings for the 'Geospatial' Data Abstraction Library
sp	SERVER	Pebesma, E.J., R.S. Bivand, 2005. Classes and methods for spatial data in R. R News 5 (2)
bezier	SERVER	Aaron Olsen (2018). bezier: Toolkit for Bezier Curves and Splines
geosphere	SERVER	Robert J. Hijmans (2021). geosphere: Spherical Trigonometry
visNetwork	UI	Almende B.V., Benoit Thieurmel and Titouan Robert (2019). visNetwork: Network Visualization using 'vis.js' Library
igraph	SERVER	Csardi G, Nepusz T: The igraph software package for complex network research, InterJournal, Complex Systems 1695. 2006
ggplot2	SERVER	H. Wickham. ggplot2: Elegant Graphics for Data Analysis. Springer-Verlag New York, 2016
RColorBrewer	SERVER	Erich Neuwirth (2014). RColorBrewer: ColorBrewer Palettes
xlsx	SERVER	Adrian Dragulescu and Cole Arendt (2020). xlsx: Read, Write, Format Excel 2007 and Excel 97/2000/XP/2003 Files
dplyr	SERVER	Hadley Wickham, Romain François, Lionel Henry and Kirill Müller (2021). dplyr: A Grammar of Data Manipulation
jsonlite	SERVER	Jeroen Ooms (2014). The jsonlite Package: A Practical and Consistent Mapping Between JSON Data and R Objects. arXiv:1403.2805

2.6 Demo

The T-Racing application is not publicly available as it handles sensitive data from Italian national information systems. Therefore, a demo version has been developed to demonstrate its potential to the scientific community. This demo uses a limited and anonymized dataset. Adjustments have been made by adding noise and rounding coordinates to the second decimal place in the georeferencing of holdings, which may result in some holdings falling outside the Italian boundaries. Additionally, it has been translated into English, while the original application is in Italian. The original development and publication environment, as well as the web services, have been duplicated and adapted for the demo. The underlying dataset is contained in a single Oracle database. The demo environment provides a comprehensive preview of its features, as listed in Table 2.

Table 2. List of Data available in T-Racing Demo.

**Data**	**From**	**To**	**Type/feature**
**Movements**	01/12/2017	30/06/2023	batch and individual for bovine and buffalo
**Outbreaks**	01/12/2017	30/06/2023	*Brucellosis, Tuberculosis*
**ST**	01/01/2015	01/12/2018	*B.abortus* and *B. melitensis* species

2.5 Brucellosis and Tuberculosis: Rationale for Case Studies and Italian Regulations

The following case studies were considered:

- **Brucellosis in the Marche region (central Italy).** On December 9, 2019, a brucellosis outbreak was confirmed in central Italy, specifically in the Marche region. The T-Racing application was utilized to identify the potential source of the infection. Only bovine species were considered, while slaughterhouses and foreign countries were excluded from the analysis. The known outbreak code from a list of proposed brucellosis outbreaks was selected as the seed for a trace backwards analysis encompassing all incoming movements to the identified outbreak over the past two years at 4 depth levels. The resulting network was refined using the "outbreaks connection" tool, and genetic data was added to support the conclusions drawn from the final network.
- **Tubercolosis outbreaks affecting Elba Island and the nearby Tuscany coast in June 2023**. A forward tracing from March to December 2023 to identify the likely spread trajectory leading up to the outbreak on Elba Island was used. Only bovine species were considered, slaughterhouses were included, and foreign countries were excluded from the analysis. Additionally, we utilized a "Seed animal's path highlighting" tool to emphasize the flow of the infection about the animals coming from the seed.
- **Tubercolosis outbreak occurred in the Cuneo province in December 2022**. A trace backward analysis over a 13-month temporal window with a level depth of 4 was used to determine the most likely source of infection. Network reduction was applied using the dedicated tools for highlighting paths among involved outbreaks ("outbreaks connection" tool).

We used T-Racing to investigate brucellosis and tuberculosis outbreaks in non–endemic areas of Italy, where an occurrence of an outbreak should trigger a rigorous investigation by the official veterinary service to prevent the disease from spreading, demonstrating the speed and effectiveness of the investigation carried out with the proposed web app.

Brucellosis caused by *Brucella abortus*, and tuberculosis caused by *Mycobacterium bovis* are important zoonosis that constitute a serious hazard to public health, and ~~where~~were the chosen diseases due to their chronic development, allowing for the use of FCT and BCT methods with a broad time-elapse. Prevention of these zoonoses in humans depends on the control of the disease in animals. Livestock movement data represent a

valuable source of information to understand the pattern of contacts between holdings, which may determine the spread of such diseases. The first national plans for the control of bovine tuberculosis and brucellosis were approved with the Ministerial Decree of 11 and 12 March 1965, respectively. Through the Ministerial Decree of 27 August 1994 n. 651 entered into force in Italy the national eradication plan for bovine brucellosis, which establishes the measures to be applied to cattle farms to obtain the eradication of the disease for the protection of public health and protection of officially free herds. The regulation determines for control the use of official serological test twice a year at an interval of not less than three and not more than six months. All animals over the age of twelve months in all cattle farms has to be tested, even if is located in the wild. Similar procedures have been established (test all animals in all heads at once, with 3 to 6 months interval) with Ministerial Decree 15 December 1995, n. 592 – Regulation concerning the national plan for the eradication of tuberculosis in cattle and buffalo herds – by testing with the tuberculin intradermal test.

3. Results

T-Racing Demo, which uses a limited and anonymized dataset, is available at ~~this link~~this link https://demo.izs.it/app_direct/tracing/~~ that has been utilized in the case studiesand it .~~. The basemaps throughout all the following figures were created using ArcGIS® software by Esri and integrated into the shiny web application using the leaflet.-providers R library, sourced from leaflet-providers.js (https://github.com/leaflet-extras/leaflet-providers). ArcGIS® and ArcMap™ are the intellectual property of Esri and are used herein under license. Copyright © Esri. All rights reserved. For more information about Esri® software, please visit www.esri.com.

Fig~~ure~~ 45 shows the T-Racing interface, which displays temporally valid paths originating from a b~~B~~rucellosis outbreak in Sicily, an Italian southern island, using the trace forward method. ~~by displaying the network related to bovine movements on a map and graph~~ The investigation revealed ~~that~~ this outbreak, detected in July 2020, was likely the source of two subsequent outbreaks in Pisa, Tuscany, northern Italy. Animals moved from the seed in March reaching via a transit station the two farms in Pisa in April and May, but the outbreaks were only detected by surveillance in December 2020. ~~resulting from investigating temporally valid paths starting from a Brucellosis outbreak in Sicily (an Italian southern island).~~ The simultaneous visualization of the ~~queryable~~qu~~a~~er~~ri~~yable and interactive graph, ~~and~~ map, and tables, combined with the ~~iterative~~iterative use of tools for network calibration and targeted result personalization, ~~provides~~ offers a more comprehensive and user-friendly result viewing~~exploratory experience~~.

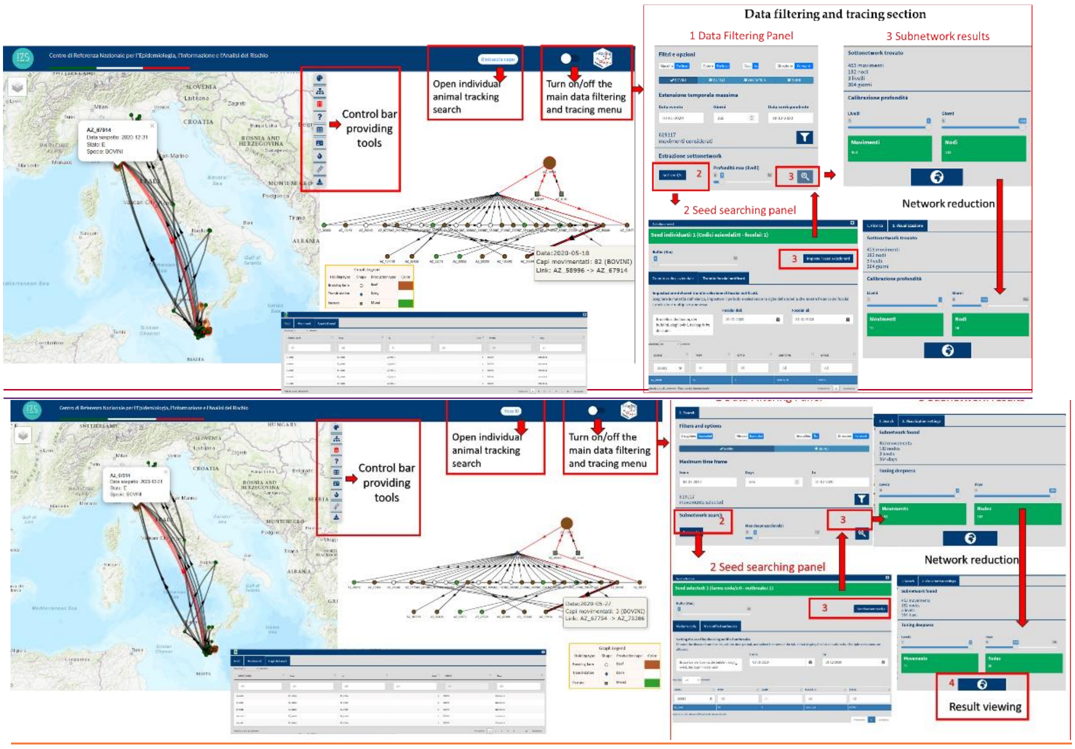

~~Node's shape represents the type of facilities (pastures being squares, holdings circles, and assembly or rest points ellipses), arrows represent movements (the width is proportional to the number of moved animals, and the red colour highlights paths followed by animals, singularly identified, had gone out of the seed). The fire icons on the map represent the Brucellosis outbreaks reached by the seed farm (red contoured circle).~~

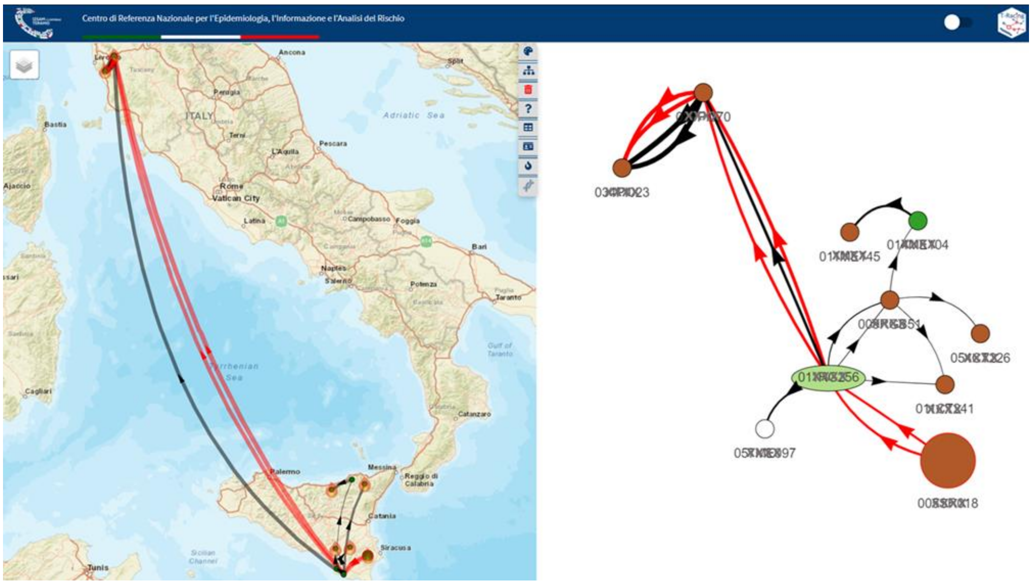

Fig~~ure~~ ~~2~~45~~.~~ The T-Racing interface (on the left) show~~ing~~s the subnetwork ~~(map and graph)~~ resulting from the trace ~~-~~forward analysis on a map, graph, and tables and the steps followed to get the results in the interface are shown on the right. This analysis was performed using ~~investigating temporally valid paths starting from~~ a b~~B~~rucellosis outbreak in July 2020 in the Sicily Region as the seed, extracting all bovine movements in 2020 up to the third level of depth (three degrees of separation). A time frame-based network reduction/optimization method was applied for ~~the final result~~the result. The "hierarchical" graph layout shows all nodes and edges in the subnetwork, highlighting probable disease pathways and nodes reached during the study period ~~from the seed~~. Node shape and color ~~indicate~~represent farm type and production activity (see legend). The ~~larger red-bordered~~larger, red-bordered icon represents the seed, with each node labeled by farm code. Arrows

indicate animal movements between two nodes, with the width proportional to the number of animals moved. Red-colored arrows highlight paths followed by animals moving from the seed (using the “Seed animals path highlighting” tool). Fire icons on the map represent b~~B~~rucellosis outbreaks detected during the selected period and included in the subnetwork (using the "Outbreaks connections" tool). Tooltips and tables provide detailed information, facilitating navigation between the map, graph, and tables.

~~*, Italy. Node’s shape represents the type of facilities (pastures being squares, holdings circles, and assembly or rest points ellipses), arrows represent movements (the width is proportional to the number of animals moved, and the red colour highlights paths followed by animals, singularly identified, had gone out of the seed). The fire icons on the map represent the Brucellosis outbreaks reached by the seed farm (red contoured circle).*~~

3.1 T-Racing ~~: -~~ ~~Simulation Exercise~~ Case Studies~~ies~~ ~~and investigation~~

~~We demonstrated the use of~~used T-Racing ~~to investigate~~ for a Brucellosis and ~~Tuberculosis outbreaks that occurred in non-endemic areas, in~~ of Italy~~, where an occurrence of an outbreak should trigger a rigorous investigation of the official service to find the origin of the infection and prevent the disease from spreading and turning the region endemic. Source detection is the key point of adopting future control measures for maintaining free area status.~~

3.1.1. Tracing backward from a notified outbreak of b~~B~~rucellosis in the Marche region of central Italy, in December 2019, to identify its potential sources of infection.~~–~~

~~At the end of 2019 (2019-12-09), a brucellosis outbreak was confirmed in central Italy, in the Marche region, which was free from the disease. The T-Racing application was utilized to identify the possible source of infection, focusing on animal trade. For tracing purposes, only bovine species were selected, excluding slaughterhouses and foreign countries as sources. A trace-backward analysis backward search was conducted, considering a broad time frame encompassing all incoming movements to the identified outbreak over the past two years. In this case study, t~~The seed was selected set ~~using one of the methods provided by the application, namely by selecting the known outbreak code from a list of proposed brucellosis outbreaks for the study period.~~ The search for the subnetwork resulted in 1.~~826~~ 825 movements involving 386 nodes. The subnetwork covers a substantial area of central Italy, including ~~establishments~~ holdings that reach the seed even from the islands of Sicily and Sardinia. The theming of nodes based on type reveals the involvement of different ~~facility~~ ~~holdings~~holding types. In addition to breeding farms, there are pastures (structures that play a significant role in the spread of brucellosis) and a hub node, a genetic collection center located in Perugia province, Umbria region, in central Italy, that ~~and~~ connect~~sing~~ 132 nodes (Fig~~ure~~ ~~3~~6). To make the network more manageable and relevant for the conducted tracing, a ~~subset~~ subnetwork was created by setting 3 levels and reducing the depth to 399 days, resulting in ~~199~~ 198 movements and 38 nodes. To identify plausible paths of disease spread, the application allows the utilization of notified outbreaks within a customizable ~~time~~ time ~~period~~ frame for the specific disease. These outbreaks are visualized on the map, enabling the tracing to be narrowed down to the connections between outbreaks included in the tracing. This helps identify a focused subset of connections to investigate. The subnetwork includes multiple b~~B~~rucellosis outbreaks, primarily located in the southern and central regions. The reduced network, ~~—~~ created by keeping only temporally valid paths among involved outbreaks~~specifically focusing on bBrucellosis outbreaks~~ within the network, consists of ~~103~~ 94 movements involving ~~14~~ 11 nodes ~~(Figure. 4)~~.

At this point, we can assess the presence of genetic information as further confirmation of the tracing's plausibility. To do so, we utilize the tool by adding the available sequence type for *Brucella abortus*. The seed is an outbreak for which sequence type "2" is available, and the identified origin zones are also affected by clusters of the same sequence type.

An interesting aspect is to determine how many of these links involve the same animals that reached the seed. The dedicated tool allows for theming the links based on this characteristic by ~~conducting a search~~searching for individually identified animals. The visualization highlights ~~that only a small part of the~~on the subnetwork the paths ~~involves~~ involving the same animals that reach the seed. These highlights paths ~~final result~~ consist~~s~~ of only 21 movements involving 5 nodes (including the seed and a pasture, which is the presumed source of infection). Further investigation of individually identified and traced animals revealed that an infected animal, identified as sequence type "2" in the seed, originated from a pasture, which was subsequently identified as the plausible source of the sought-after infection (Fig~~ure.~~ ~~5~~7).

~~The presented tracing example has highlighted a plausible path of disease diffusion and the presumed origin. Starting from a relatively large and heterogeneous network (1.826x386) in terms of both spatial and temporal dimensions, it wasbeing possible to focus attention on a result of 21 movements and 5 nodes in just a few steps, saving a great amount of time by utilizing interactive and iterative use of complementary information, such as disease outbreaks, genetic information, and movements of individually identified animals.~~

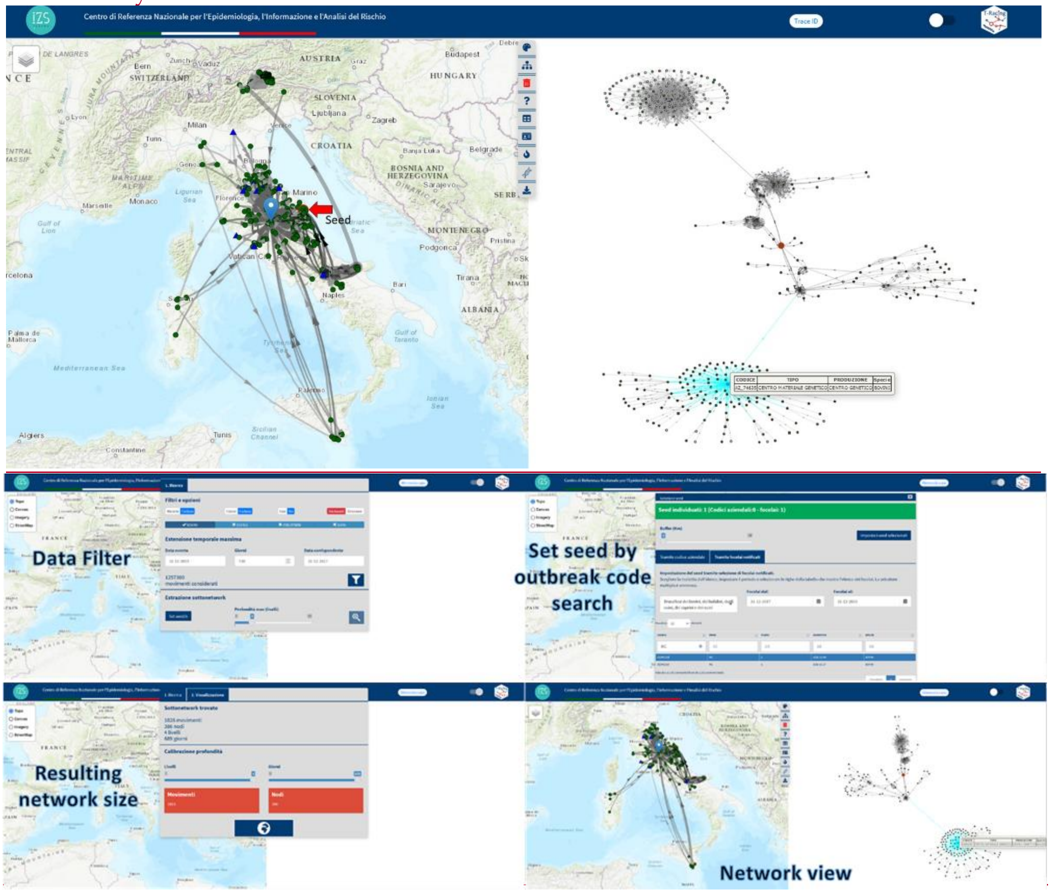

Fig~~ure~~ ~~3~~6~~.~~ Trace -backward ~~simulation~~method. Sequential investigation ~~depiction of four~~ steps: 1) Data Filter, 2) Set seed by outbreak code search, ~~3) Resulting network size,~~ and ~~4~~3) Network view. The trace-back focusing on bovine species, excluding slaughterhouses and foreign countries, was conducted to trace the outbreak occurring in December in the Marche region, in Italy. 1,~~826~~ 825 movements involving 386 nodes were identified within a broad time spanning the past two years. The seed is contoured in red. The network covers a substantial area of central Italy, including farms extending to the islands (Sicily and Sardinia). Node theming based on structure type highlights diverse ~~farm~~ holding types, including pastures and a genetic collection center ~~located~~ in Perugia, ~~and~~ highlighted in light blue on the graph (simultaneously, its marker appears on the map)~~in the figure~~.

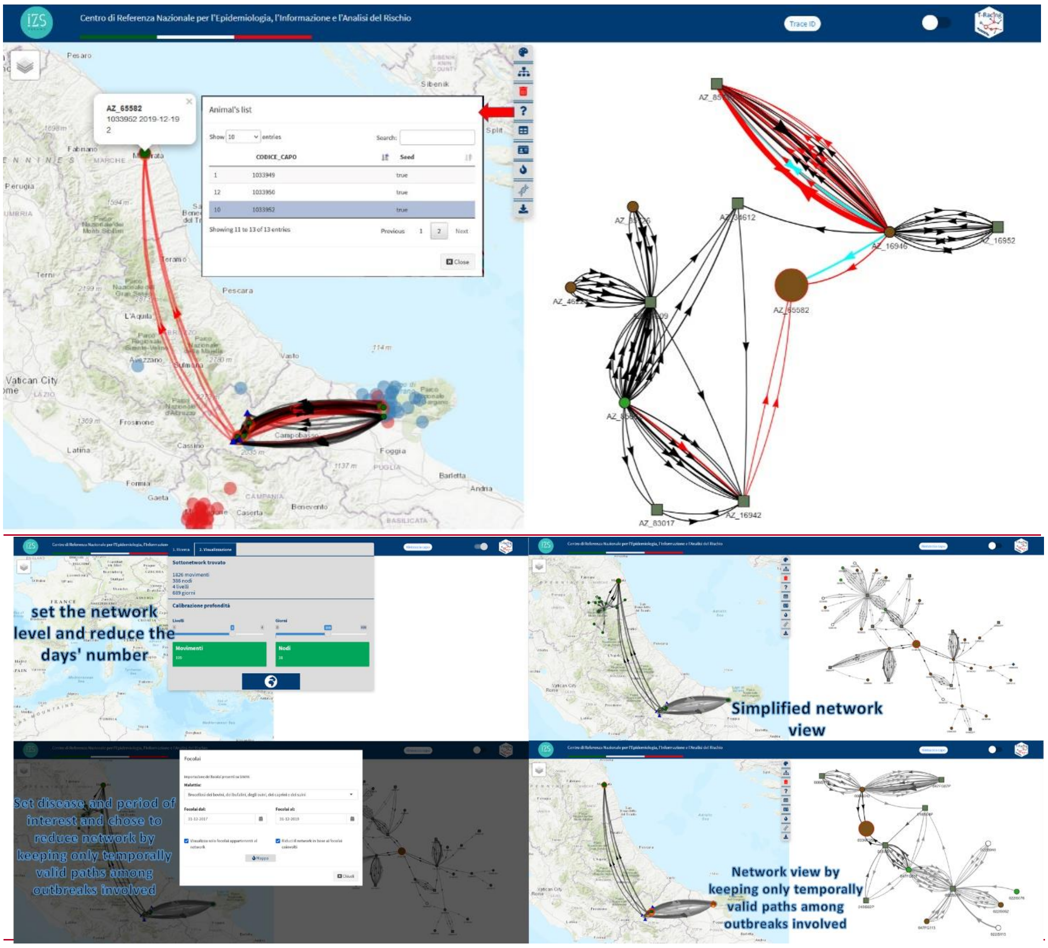

Figure ~~4~~7~~.~~ ~~A large network is often impractical and less informative for the tracing process. T-Racing allows for reducing the network using diverse tools.~~ Th~~e figure shows a possible~~the subnetwork result after the following four sequential ~~of four~~ steps: 1) Network reduction by setting the network level to 3 –and ~~reducing~~ days~~'~~ depth to 399 ~~number~~ (the new subnetwork size is ~~199~~ 198 links and 38 nodes), ~~2) Reduced network view, 3~~2) Further ~~R~~reducing the subnetwork by ~~setting outbreaks for a specific disease and period of interest 4) Resulting network by~~ keeping only temporally valid paths among involved outbreaks ~~involved~~ (resulting in a ~~the~~ subnetwork size ~~is~~of ~~103~~ 94 links and ~~14~~ 11 nodes~~; and~~ the ~~three~~ four detected b~~B~~rucellosis' outbreaks involved ~~involved~~ are shown on the map with ~~the~~ red fire icons), 3) Adding the sequence type for *Brucella abortus* layer to the map (type 2 in blue circles) and displaying a popup on the seed showing the identification code of the animal, sampled date, and detected *Brucella abortus* sequence type, and 4) Highlighting on the graph are all links involving the same animals that reached the seed (red links). Further investigation on "individually identified animals," found by querying the highlighted links (in light blue on the graph), revealed that an infected animal originating from the pasture reached the seed. This animal is listed in the Animal's list produced by the application, matching the animal reported in the map popup.

3.1.2. Tracing the flow of t~~T~~uberculosis o~~O~~utbreaks: Elba Island and Cuneo Province.~~Case study: In Italy, Bovine Tuberculosis (BTB) is endemic in the southern region. However, sporadic outbreaks occur in non-endemic area~~areas~~. Two interesting example~~examples of ~~how this application can help in controlling the spread of the disease or in tracing the source of the infection, are presented hereafter: The Elba island outbreak and the Cuneo province in Piedmont region (northern Italy) outbreak.~~

-**Four BTB outbreaks were notified ~~during~~ in June 2023 in a small, non-endemic area, in Italy, affecting Elba ~~island~~ Island and the ~~near~~ nearby Tuscany coast**. Using T-Racing allowed for the detection of the real flow of the disease spread. ~~Indeed, a forward tracing starting from the outbreak detected in Elba island ,~~The resulting network –highlighted

an animal movement ~~(~~of nine cattle~~)~~ ~~during~~ in April 2023, which ~~reaching~~ reached ~~the~~ farms on the nearby coast. ~~and~~ This ~~let to establish~~ establish~~inged~~ ~~–~~the real source of the infection and the most likely flow of the spread (Fig~~ure~~ ~~6~~8~~.~~). ~~). Interestingly, the movements reached four farms in~~on ~~the Tuscany coast (, yet only three of which,~~these ~~were~~ ~~confirmed as outbreaks). . Using a specific tool to query the individually identified animals,~~ W~~w~~e discovered that the animals reaching the outbreaks were the same ~~animals~~ ones that had ~~leaving~~ left Elba ~~island~~Island and one of them (at the time of writing this article) had not yet been slaughtered. ~~and some of them (at the time of writing this article) had not yet been slaughtered .~~

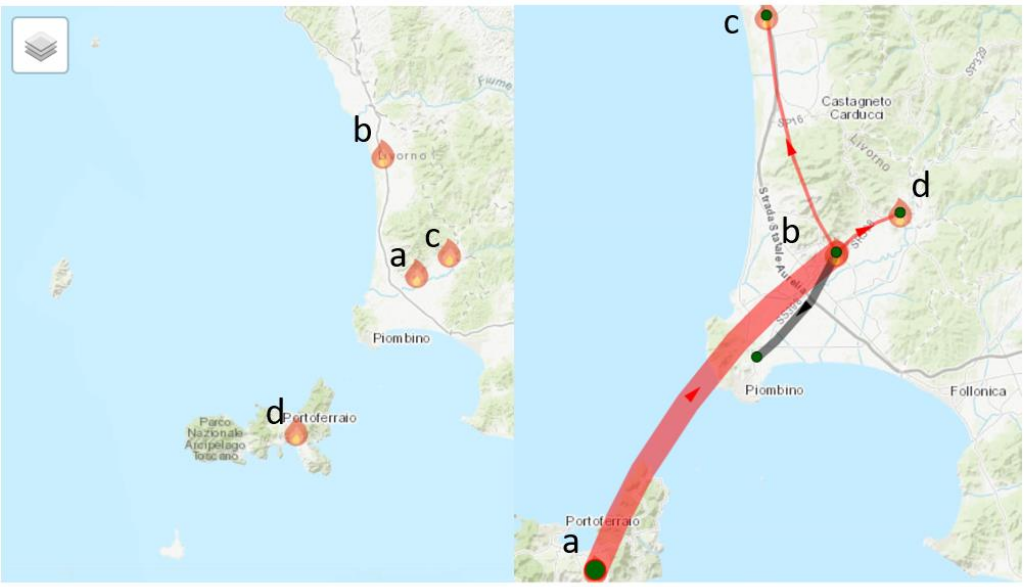

~~**Figure 6.** Trace –forward simulation. Left: show~~~~Outbreaks that occurred in the study area, arranged in the order of their notification timing (with 'a' representing the earliest and 'd' the most recent). Right: Network of animal movements involving the outbreaks (red links indicate the presence of at least one animal originating from the seeding site, while black links represent movements of animals not originating from the seed).~~

~~**BTB outbreaks in northern Italy are considered uncommon.**~~ **In December 2022, an outbreak of BTB occurred in the Cuneo province of the Piedmont region in northern Italy.** ~~To determine the most likely source of infection, we employed T-Racing, utilizing a wide temporal window of one year to track incoming movements.~~ The resulting network ~~encompassed~~ comprised ~~353~~ 306 farms~~, with~~ and ~~approximately six hundred~~664 connections, predominantly located in the Sicily region. For ~~To facilitate~~ a more focused investigation, we considered only temporally valid ~~employed a tool that retained only~~ paths connecting BTB outbreaks included in the network resulting~~resulting~~ in a ~~simplified~~ subnetwork of ~~72~~ 50 connections involving ~~nine~~ 9 breeding farms in Sicily region, ~~three~~ four of which ~~were~~ BTB outbreaks, and two pastures. The investigation ~~thus~~ suggested that ~~three~~ four outbreaks ~~occurred~~ occurring ~~during~~ in the previous months ~~as~~ are the most likely source of infection. ~~Furthermore~~Additionally, the 119 animals~~-~~ reaching the Cuneo province in June and July ~~came~~ originated from a fattening farm where they were ~~probably~~ likely infected by mixing with animals coming from the four outbreaks. The results indicate ~~a~~the need for targeted control measures for the other five farms involved in the revealed diffusion paths (Fig~~ure~~ ~~7~~9).

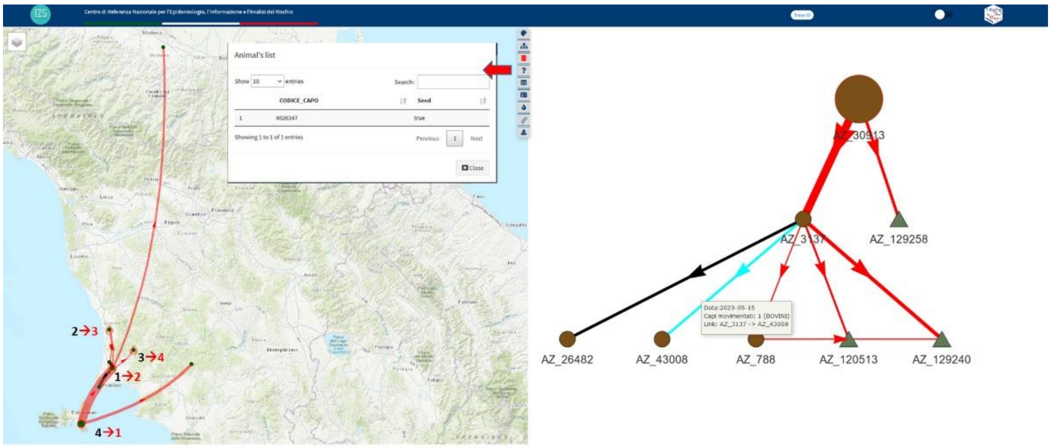

Fig 8 Trace forward method. The map and graph show outbreaks that occurred on Elba island and the nearby Tuscany coast (black numbers '1' to '4', represent the notification timing, with 1 the earliest and '4' the most recent notified). The network of animal movements involving the outbreaks is displayed with red links indicating the presence of at least one animal originating from the seed, and black link representing the movement of animals not originating from the seed. The notified outbreaks timing contrasts with the real flow of disease spread revealed by T-Racing analysis (red numbers '1-4'). The triangles on the graph are slaughterhouse. Further investigation on "individually identified animals," found by querying the highlighted link (in light blue on the graph), revealed that one animal originating from the seed had not yet been slaughtered. This animal is listed in the Animal's list produced by the application.

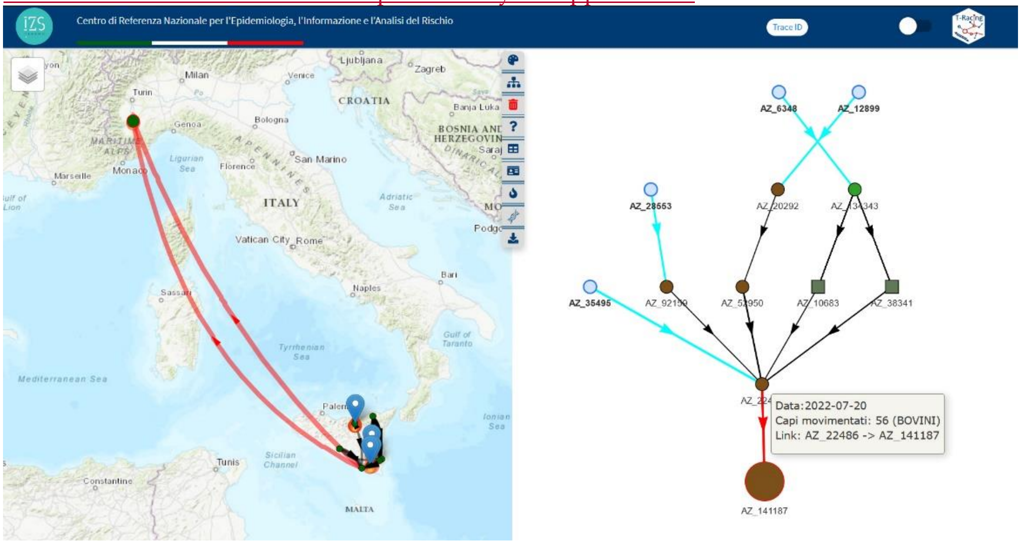

Fig~~ure~~ ~~7~~9~~.~~ Trace backward method. The network resulting from the BTB outbreak occurred in Cuneo province, in the Tuscany region in December 2022 involving 306 farms and 664 links, mostly located in the Sicily region. The map and graph show the reduced network by considering only temporally valid paths connecting involved outbreaks consisting of 50 connections and 12 holdings which 9 breeding farms in the Sicily region (circles on the graph), four of which are BTB outbreaks (fire icons and marked on the map and highlighted in light blue on the graph), and two pastures (squares on the graph). The links involving animals reaching the Cuneo outbreak are in red color.

~~Trace back simulation. The network resulting from the trace back from the Cuneo BTB outbreak involved 353 farms (with about six hundred links), mostly located in the Sicily region (left-up). To simplify this network and make it possible to investigate deeply, we used the tool letting to retain only paths among outbreaks. The simplified network (left-down) resulted in 72 links involving 10 farms (3 being BTB outbreaks). Details on the network involving 3 outbreaks in the Sicily region and their outgoing movements are light blue highlighted on the right-up side~~upside~~. The links involving the farm where the mixing of infected animals may have occurred are shown in the right-down panel, along with the links involving animals reaching the Cuneo outbreak (red arcs).~~

4. Discussion

Preventing and controlling communicable diseases in livestock, through outbreak early detection, increases the effectiveness of the emergency response and reduce the social, economic, and environmental costs associated with the outbreak [1, 2]. TTimely identification of outbreaks and systematic contact tracing can be effective in stopping the transmission chain of highly contagious diseases, especially with provided there is strong collaborations between epidemiological teams, laboratory surveillance, and decision-making officials, improving outbreak management, in a One- Health context [212337]. An important feature of the modern response to epidemics is the increasing focus on exploiting all available data to inform the response in nearly real-time and allow for the data evidence-based decision-making [38]. Therefore, the aim of this paper was to develop a web-based tracing app, T-Racing, that improved the identification of outbreak origin and dissemination, by focusing the traceability on almost real-time data, increasing the decision power of animal health authorities dealing with diseases control and eradication.

T-Racing has the potential to significantly enhance animal disease control measures, as it can rapidly organize crucial contact information from extensive datasets and identify connections in a multi-area outbreaks investigation. It introduces new methods and tools currently not available in other systems or tools [2, 24, 33, 34, 35, 36]. These include multi-seed/species selection, seed selection using a spatial buffer around cases or outbreaks, and the combination of strategies (FCT-BCT) configuring itself as an innovative application in its field. Ts, thus, the application efficiently supportsing epidemiological approachesinvestigations, as it was shown in the demo examples for brucellosis and tuberculosis. In these examples, it is possible to see how deep into the transmission chain the T-Racing allows us to explore. The initial tracing example has identified a likely path of disease spread and the presumed origin, starting from a relatively large and diverse network (1.825x386) in both spatial and temporal dimensions. By using interactive and iterative tools, along with additional information such as disease outbreaks, genetic data, and movements of individually identified animals, it was possible to narrow down the focus to 21 movements and 5 nodes in just a few steps, saving a significant amount of time. Trying to achieve the same result by simply extracting the movement network through a query of the NDB would have been extremely difficult, like finding a needle in a haystack. The second example clarified the actual timing of the tuberculosis spread. Importantly, the animals from the seed had not been slaughtered at the time of writing this article, and in the last example results indicate a need for targeted control measures for the other five farms involved in the revealed diffusion paths.

Considering chronical diseasesinvestigations., such as brucellosis and tuberculosis, clarifying the diseases spread patterns allows for focus measures of control, improving targeting financial and human resources, as well as time sensitive decisions. Additional features .

One Health is the collaborative efforts of multiple disciplines to attain optimal health for people, animals and the environment, a concept that historically owes much to the study of brucellosis, including recently political and ethical considerations. Brucellosis One Health actors include Public Health and Veterinary Services, microbiologists, medical and veterinary practitioners and breeders. Brucellosis awareness, and correct use of diagnostic, epidemiological and prophylactic tools is essential [22]. Thus, racing the spread of diseases within animal populations, and tracing potential routes of exposure and spread is critical for effective diseases control [15] and a further important actor of the One-Health concept. Contact tracing plays a pivotal role in breaking the transmission chain, reducing the spread and duration of infectious diseases and preventing new cases [16]. Animal movements (from an origin to a destination farm) represent the major source of infection between animal populations, especially in the case of direct-contact diseases, so should be always explored as a path for disease spreading. Indeed, the actions following outbreak detections, although well-timed, can avoid only a part of the potential disease diffusion pathways that tracing on animal movements can detect (Figure 8).

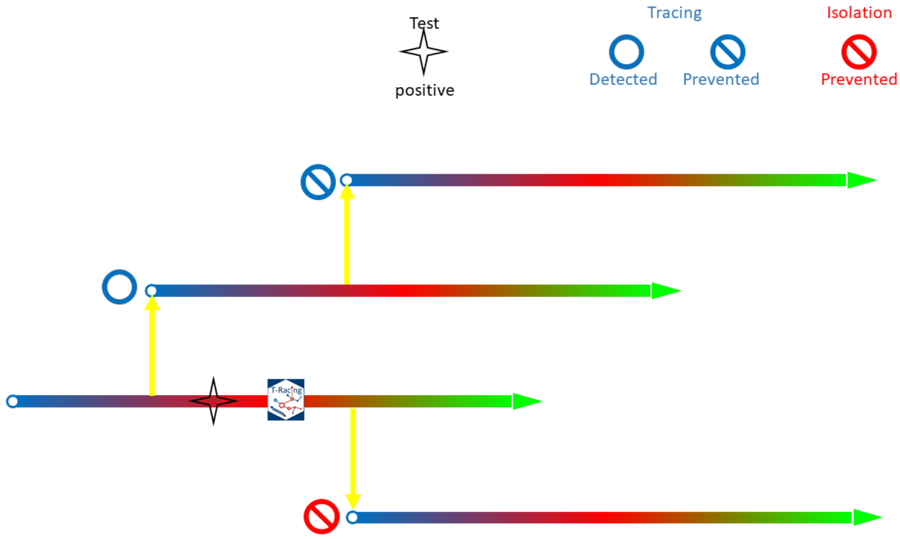

Figure 8. Illustration of the importance of tracing in preventing and detecting diffusion path-ways versus the simple farm isolation when only positivity recorded in the context of the surveillance programme is considered.

So having data about traded animals may seem enough for an effective approach. However, a simple and raw query on traded animal data may result in a huge network, sometimes confusing or completely useless. Then, a tool able to manage huge datasets and to add useful information is needed to understand where the disease most likely came from, or could have gone.A goodT-Racing, such asIts scalability, of the system would enables the easy incorporation of additional functionalities and information, such as test and inspection histories, additional information linked to single animal like the last date of parto, as well as disease status of the establishments, in the light of an enhanced disease control programs. Noteworthy, the almost real-time data used, the possibility of analyzing many datasets at once and the user-friendly environment are also features that could greatly advance responses to emergencies with acute and highly contagious diseases, when movement data could be generated on real time.

Complementarily, the application of molecular epidemiological data into the analysis, validating the identified paths of disease (brucellosis) spread, is an example of a One Health approach by a multidisciplinary team on building a new technology for disease control. An important feature of the modern response to epidemics is the increasing focus on exploiting all available data to inform the response in nearly real-time and allow the data evidence-based decision-making [2325].

This tool has the potential to significantly enhance animal disease control measures, as it can rapidly organize crucial contact information from extensive datasets and identify connections in multi-area outbreak investigations, thus efficiently supporting epidemiological investigations.

In this senseLastly, in an era of rapid technological advancements, it is essential to foster innovation and develop tools that revolutionize epidemiological investigations, bBy combining cutting-edge technology with comprehensive data analysis, which could, T-Racing opens new possibilities for effective and efficient disease control in animal disease outbreaks.

The presented simulation exercises highlightedhighlights the most plausible path of disease diffusion and the presumed origin/source of the infection in just a few steps, utilizing interactive and iterative use of complementary information, such as disease outbreaks, genetic information, and movements of individually identified animals.

benefitBuilding trust should be one critical goal of One Health programs; thus, it should exist at all levels, including between the Veterinary and Human Health systems while dealing with zoonosis – [222438]. HanceHence,

wWith its user-friendly interface and interactive features, T-Racing empowers clinicians, veterinarians, ecosystem health professionals, researchers, and policymakers to leverage the power of animal surveillance within the One Health perspective [3, 242639]. In that regard, byBy embracing this innovative tool, stakeholders can gain valuable insights, make informed decisions, and take proactive measures to mitigate the impact of infectious diseases on animal and human health. Therewith, tThis dynamic approach enables the T-Racing application to provide accurate and timely data for epidemiological investigations. Complementary,, the system emphasizing emphasizes its data-driven nature,. rIt recognizing es the crucial role of accurate data in conducting effective investigations and highlights the need for robust practices on data management practices. Noteworthy, wWithout reliable data, the utility and reliability of the T-Racing application would be compromised.

Among the limitations of T-Racing is that the app is an ongoing project, and in future versions, the application will be enhanced with new datasets and functionalities. Additionally, the current genetic data is limited to the *Brucella* genus, and additional pathogen data will be added as soon as they are available in Genpat. Furthermore, data about controls from surveillance systems in farms will be included, and functions will be developed to account for the timing controls in selecting the most likely disease spread paths. Simultaneously, we are evaluating the development of a scheduled algorithm that works directly on the database to identify critical networks to be provided to the application users. This will allow us to check and start investigations more efficiently, rapidly, and straightforwardly.Although T-Racing is currently operational, the authors and team of experts will continue to develop future versions to improve the application by adding new datasets and functionalities. At present, the genetic data is restricted to the *Brucella* genus, but additional pathogen data will be incorporated as soon as it becomes available in GenPat. Data regarding testing from surveillance systems on farms will be included, and functions will be created to factor in the timing of testings when selecting the most probable disease spread paths. Also, detailed information about single animals, like demographics and parturition dates for calves, along with humans' cases will be considered. There is also an evaluation underway for the development of scheduled algorithms that will operate directly on the database to identify critical networks to be automatically provided to T-Racing's users. This will enable more efficient, rapid, and straightforward validation and initiation of investigations. Concurrently, work is ongoing for a more general application that allows users to upload their own data, and its source code will be freely distributed on GitHub.

5. Conclusions

Therefore, theThe T-Racing presented web app, T-Racing, represents a modern and forward-thinking approach fortoto supporting epidemiological investigations in animal disease outbreaks., It provides a unified environment to manage and analyze the expanding pool of data sources iteratively bBby. By integrating advanced technology, comprehensive data management, and cutting-edge analytics, it it provideingses a unified environment to manage and analyseanalyze the expanding pool of data sources iteratively. Moreover, T-Racing Withhas the its capability to draw meaningful conclusions based on data-driven, user-guided approach while leveraging their expertise. offer meaningful insights andIt facilitates evidence-based decision-making, and serves as a leadingT-Racing standings at the forefront of disease control tools in disease control, strengthening enhancing our ability capacity to address emerging challenges and, and protecting safeguard global health, in from a One- Health perspective.

Author Contributions: Conceptualization, L.C., L.S., A.C., P.C. and F.D.M.; methodology, L.C. and L.S.; software, L.C. and S.P.; validation, F.D.M. and P.C.; data curation, D.P.; writing—original draft preparation, L.S., L.C., A.C.T.R.B.C. and F.D.M.; writing—review and editing, L.C., L.S., A.C., F.D.M., P.C., S.P., A.D.L., D.P., S.T., S.S., and M.T.; project administration, P.C.; funding acquisition, P.C.. All authors have read and agreed to the published version of the manuscript.

Funding: This research was funded by Italian Ministry of Health (RC MSRCTE0219).

Conflicts of Interest: The authors declare no conflict of interest.

References

~~Schirdewahn, F., K. Lentz, H. H., Colizza, V., Koher, A., Hövel, P., & Vidondo, B. Early warning of infectious disease outbreaks on cattle transport networks. PLOS ONE, 16(1), e0244999.~~

1 Schirdewahn, F., K. Lentz, H. H., Colizza, V., Koher, A., Hövel, P., & Vidondo, B. Early warning of infectious disease outbreaks on cattle-transport networks. PLOS ONE, 16(1), e0244999. https://doi.org/10.1371/journal.pone.0244999

2 Maria Nöremark and Stefan Widgren. EpiContactTrace: an R-package for contact tracing during livestock disease outbreaks and for risk-based surveillance. BMC Veterinary Research 2014, 10:71 Page 2 of 9 http://www.biomedcentral.com/1746-6148/10/71

3 Prata, J. C., Ribeiro, A. I., & Rocha-Santos, T. (2022). An introduction to the concept of One Health. One Health, 1-31. https://doi.org/10.1016/B978-0-12-822794-7.00004-6

4 Wondwossen A. Gebreyes, Daral Jackwood, Celso Jose Bruno de Oliveira, Chang-Won Lee, Armando E. Hoet, Siddhartha Thakur. Molecular Epidemiology of Infectious Zoonotic and Livestock Diseases. 2020. Microbiology Spectrum, 10.1128/microbiolspec.ame-0011-2019, 8, 2, https://journals.asm.org/doi/abs/10.1128/microbiolspec.ame-0011-2019

5 Neo, J. P. S., & Tan, B. H. (2017). The use of animals as a surveillance tool for monitoring environmental health hazards, human health hazards and bioterrorism. Veterinary Microbiology, 203, 40-48. https://doi.org/10.1016/j.vetmic.2017.02.007

6 D Kriebel, J Tickner, P Epstein, J Lemons, R Levins, E L Loechler, M Quinn, R Rudel, T Schettler, A M Stoto. The precautionary principle in environmental science. 2001, Environmental Health Perspectives, 871-876, 109, 9, https://ehp.niehs.nih.gov/doi/abs/10.1289/ehp.01109871

7 Rocha VC Figueiredo, Souza-Filho AF, Ikuta CY, et al. High discrimination of Mycobacterium bovis isolates in Brazilian herds by spoligotyping. **Preventive Veterinary Medicine**, v.179, p.104976, 2020. Doi: 10.1016/j.prevetmed.2020.104976.

8 Taye H, Alemu K, Mihret A, Wood JLN, Shkedy Z, Berg S, Aseffa A. Global prevalence of Mycobacterium bovis infections among human tuberculosis cases: Systematic review and meta-analysis. Zoonoses Public Health. 2021 Nov;68(7):704-718. doi: 10.1111/zph.12868. Epub 2021 Jun 24. PMID: 34169644; PMCID: PMC8487997.

~~1~~9 Laine CG, Johnson VE, Scott H, Arenas-Gamboa AM. Global Estimate of Human Brucellosis Incidence. Emerg Infect Dis. 2023;29(9):1789-1797. https://doi.org/10.3201/eid2909.230052

10 Ansari, S., Heitzig, J., Brzoska, L., Lentz, H. H., Mihatsch, J., Fritzemeier, J., & Moosavi, M. R. (2021). A Temporal Network Model for Livestock Trade Systems. Frontiers in Veterinary Science, 8. https://doi.org/10.3389/fvets.2021.766547

11 Loria GR, Migliore S, Bongiorno C, Ciaccio G and Laddomada A (2022) New European rules introduced by regulation (EU) 2016/429 to facilitate animal trade: With great risk comes great responsibility. Front. Vet. Sci. 9:1003732. https://doi.org/10.3389/fvets.2022.1003732

12 Porphyre, T., Boden, L. A., Correia-Gomes, C., Auty, H. K., Gunn, G. J., & J. Woolhouse, M. E. (2016). Using national movement databases to help inform responses to swine disease outbreaks in Scotland: The impact of uncertainty around incursion time. Scientific Reports, 6. https://doi.org/10.1038/srep20258

13 Regulation (EU) 2016/429 of the European Parliament and of the Council of 9 March 2016 on transmissible animal diseases and amending and repealing certain acts in the area of animal health ('Animal Health Law') (Text with EEA relevance). Off J Eur Communities. (2016) L84:1–208

14 ORDINANZA 9 agosto 2012 Misure straordinarie di polizia veterinaria in materia di tubercolosi, brucellosi bovina e bufalina, brucellosi ovi-caprina, leucosi, nelle regioni Calabria, Campania, Puglia e Sicilia. (12A09729) (GU Serie Generale n.212 del 11-09-2012) https://www.gazzettaufficiale.it/eli/id/2012/09/11/12A09729/sg (visitato il 25/08/2023)

15 ORDINANZA 28 maggio 2015, Misure straordinarie di polizia veterinaria in materia di tubercolosi, brucellosi bovina e bufalina, brucellosi ovi-caprina, leucosi bovina enzootica. (15A04879) (GU n.144 del 24-6-2015) and following amendments and additions

16 2003/24/EC: Commission Decision of 30 December 2002 concerning the development of an integrated computerised veterinary system (notified under document number C(2002) 5496)

17 EU, 2019. Commission Implementing Regulation (EU) 2019/1715 of 30 September 2019 laying down rules for the functioning of the information management system for official controls and its system components (the IMSOC Regulation) OJ L 261, 14.10.2019

18 Mardones, F., zu Donha, H., Thunes, C., Velez, V., & Carpenter, T. (2013). The value of animal movement tracing: A case study simulating the spread and control of foot-and-mouth disease in California. Preventive Veterinary Medicine, 110(2), 133-138. https://doi.org/10.1016/j.prevetmed.2012.11.024

19 Judite Monteiro Braga et al. Investigation and contact tracing of the first cases of COVID-19 in Mozambique, 2020. Pan African Medical Journal. 2022;41:302. https://doi.org/10.11604/pamj.2022.41.302.26979

20 Kölzsch, A., Davidson, S.C., Gauggel, D. et al. MoveApps: a serverless no-code analysis platform for animal tracking data. Mov Ecol 10, 30 (2022). https://doi.org/10.1186/s40462-022-00327-4

21 Bingham, P., Wada, M., van Andel, M., McFadden, A., Sanson, R., & Stevenson, M. (2020). Real-Time Standard Analysis of Disease Investigation (SADI)—A Toolbox Approach to Inform Disease Outbreak Response. Frontiers in Veterinary Science, 7, 563140. https://doi.org/10.3389/fvets.2020.563140

22 Hang, C.-N.; Tsai, Y.-Z.; Yu, P.-D.; Chen, J.; Tan, C.-W. Privacy-Enhancing Digital Contact Tracing with Machine Learning for Pandemic Response: A Comprehensive Review. Big Data Cogn. Comput. 2023, 7, 108. https://doi.org/10.3390/bdcc7020108

23 Anglemyer A, Moore TH, Parker L, Chambers T, Grady A, Chiu K, Parry M, Wilczynska M, Flemyng E, Bero L. Digital contact tracing technologies in epidemics: a rapid review. Cochrane Database Syst Rev. 2020 Aug 18;8(8):CD013699. doi: 10.1002/14651858.CD013699. PMID: 33502000; PMCID: PMC8241885.

24 Savini, L., Candeloro, L., Perticara, S., & Conte, A. (2019). EpiExploreR: A Shiny Web Application for the Analysis of Animal Disease Data. Microorganisms, 7.SIMAN. Available online: https://www.vetinfo.sanita.it/j6_siman/.

25 Sistema Informativo Veterinario. Available online: https://www.vetinfo.sanita.it/sso_portale/accesso.pl .

26 Decreto 30 maggio 2017. Istituzione di un Centro di referenza nazionale nel settore veterinario. G.U. n 196 del 26 Agosto, 2017

27 Di Lorenzo A, Mangone I, Colangeli P, Cioci D, Curini V, Vincifori G, Mercante MT, Di Pasquale A, Iannetti S. One health system supporting surveillance during COVID-19 epidemic in Abruzzo region, southern Italy. One Health. 2023 Jun;16:100471. https://doi.org/10.1016/j.onehlt.2022.100471. Epub 2022 Dec 7. PMID: 36507072; PMCID: PMC9726647.

28 Diestel, R. 2010. Graph theory. Graduate Texts in Mathematics, Vol. 173, Fourth Edition, Springer-Verlag,866 Heidelberg.

29 Blonder, B., Wey, T.W., Dornhaus, A., James, R. and Sih, A. (2012), Temporal dynamics and network analysis. Methods in Ecology and Evolution, 3: 958-972. https://doi.org/10.1111/j.2041-210X.2012.00236.x

30 Chan J, Holmes A, Rabadan R. Network analysis of global influenza spread. PLoS Comput Biol. 2010 Nov 18;6(11):e1001005. doi: 10.1371/journal.pcbi.1001005. PMID: 21124942; PMCID: PMC2987833

31 Chris Rorres, Maria Romano, Jennifer A. Miller, Jana M. Mossey, Tony H. Grubesic, David E. Zellner, Gary Smith, Contact tracing for the control of infectious disease epidemics: Chronic Wasting Disease in deer farms, Epidemics, Volume 23, 2018, Pages 71-75, ISSN 1755-4365. https://doi.org/10.1016/j.epidem.2017.12.006.

32 Natale F, Savini L, Giovannini A, Calistri P, Candeloro L, Fiore G. Evaluation of risk and vulnerability using a Disease Flow Centrality measure in dynamic cattle trade networks. Prev Vet Med. 2011 Feb 1;98(2-3):111-8. doi: 10.1016/j.prevetmed.2010.11.013. Epub 2010 Dec 15. PMID: 21159393.

33 Calistri P, Conte A, Natale F, Possenti L, Savini L, Danzetta ML, Iannetti S, Giovannini A. Systems for prevention and control of epidemic emergencies. Vet Ital. 2013 Jul-Sep;49(3):255-61. doi: 10.12834/VetIt.1206.06. http://www.izs.it/vet_italiana/pdf/VetIt_1206_06.pdf

34 Iannetti S, Savini L, Palma D, Calistri P, Natale F, Di Lorenzo A, Cerella A, Giovannini A. An integrated web system to support veterinary activities in Italy for the management of information in epidemic emergencies. Prev Vet Med. 2014 Mar 1;113(4):407-16. doi: 10.1016/j.prevetmed.2014.01.015. Epub 2014 Jan 22. PMID: 24485707.

35 Di Lorenzo A., Savini L., Candeloro L., Tora S., Cerella A., Di Sabatino D., Conte A. "Web-GIS and livestock trace tools for epidemiological surveillance, control and management" Front. Vet. Sci. doi: 10.3389/conf.fvets.2019.05.0004

36 Tegegne, H. A., Bogaardt, C., Collineau, L., Cazeau, G., Lailler, R., Reinhardt, J., Freeth, F. T., Taylor, E., Prada, J. M., & Hénaux, V. (2023). OH-EpiCap: A semi-quantitative tool for the evaluation of One Health epidemiological surveillance capacities and capabilities. Frontiers in Public Health, 11, 1053986. https://doi.org/10.3389/fpubh.2023.1053986

37 Jonathan A. Polonsky, Amrish Baidjoe, Zhian N. Kamvar, Anne Cori, Kara Durski, W. John Edmunds, Rosalind M. Eggo, Sebastian Funk, Laurent Kaiser, Patrick Keating, Olivier le Polain de Waroux, Michael Marks, Paula Moraga, Oliver Morgan, Pierre Nouvellet, Ruwan Ratnayake, Chrissy H. Roberts, Jimmy Whitworth and Thibaut Jombart. Outbreak analytics: a developing data science for informing the response to emerging pathogens. Philosophical Transactions of the Royal Society B: Biological Sciences, Volume 374, Issue 1776. https://doi.org/10.1098/rstb.2018.0276

38 Moriyón, I., Blasco, J. M., Letesson, J. J., De Massis, F., & Moreno, E. Brucellosis and One Health: Inherited and Future Challenges. Microorganisms, 11(8), 2070. https://doi.org/10.3390/microorganisms11082070

39 Charlier, J., Barkema, H. W., Becher, P., De Benedictis, P., Hansson, I., Hennig-Pauka, I., La Ragione, R., Larsen, L. E., Madoroba, E., Maes, D., Marín, C. M., Mutinelli, F., Nisbet, A. J., Podgórska, K., Vercruysse, J., Vitale, F., Williams, D. J. L., & Zadoks, R. N. (2022). Disease control tools to secure animal and public health in a densely populated world. The Lancet Planetary Health, 6(10), e812-e824. https://doi.org/10.1016/S2542-5196(22)00147-4

Responses to Reviewers

We are grateful to the reviewers for their detailed review and for acknowledging the main purpose of describing the T-Racing application in a scientific article. T-Racing is the result of extensive efforts by a multidisciplinary team that collaborated throughout all stages of development— from data analysis to creating the tools and developing a user-friendly interface. This process involves analyzing large and diverse datasets that, on their own, cannot provide useful information. These data continually evolve and as new or edited information and attributes are provided T-Racing is consequently adjusted. These data are harmonized and made usable within an application that allows for iterative analysis and visualization in a user-friendly manner. The scalability of the application permits easily integration of future datasets and *ad hoc* tools.

The One Health approach is relevant to this work because most zoonotic diseases are transmitted by animals. Effective control of these animals can help break or contain the transmission chain to humans. Future development on tracing will include the incorporation of additional information, such as expanding genomic data on sampled animals or even adding human cases and other relevant data as soon as they become available.

We acknowledge the complexity of describing such work in a paper and appreciate the reviewers' comments. While these comments have required significant revisions, they have provided the opportunity to prepare a more complete and significantly improved version of the manuscript.

Below, we have provided punctual responses to the requests and comments from each of the reviewers (our responses are highlighted in red).

Specific Responses to Reviewers

1. The reviewers requested providing a working instance of the tool (online or as a stand-alone application to be downloaded): This concern has been addressed by providing a demo version of the application. The development and dataset to the demo were described in a dedicated section in the Materials and Methods, as well as the link to the demo is provided at the beginning of the Results section.
2. The reviewers suggested restructuring the organization and narrative of the manuscript: We have thoroughly restructured the manuscript by creating new paragraphs and sub-sections in the Materials and Methods, redistributing the text as suggested by the reviewers and editor, and completely rewriting the Discussion section. Additionally, all redundancies present in the previous manuscript version have been eliminated.
   The reviewers requested higher quality figures: We have recreated all the figures in higher resolution. We used the demo version of T-Racing to redo the case studies and the related figures.

Reviewer 1

The insertion of the case studies is essential for describing the tool but the subject matters, BTB and Brucellosis, aren't be properly described in terms of their aetiology, natural history, etc.; nor can they be. I'm not sure how to address this. Possibly a single paragraph on each disease as part of the case study introduction? Thank you for the suggestion. We have added a specific paragraph in the Materials and Methods section (subparagraph 2.5) and have removed all non-pertinent information from the Results section (subparagraphs 3.1.1 and 3.1.2). This information has been redistributed to the Materials and Methods or Discussion sections as appropriate.

It is inferred in the text, but I think there should be more a more explicit description of how the user's expert elicitation is required to drive the tool. The tools helps guide the user rather than offering an instant solution.

That is a good consideration. We have integrated this concept at the end of the Abstract, Introduction, and Discussion sections.

I assume the tool is a work-in-progress? This should also be addressed. Are their future plans for improvements/enhancements? Can the tool be leveraged to provide researchers with data on animal contacts of disease modelling, etc? These would make useful discussion points. As clarified in the revised manuscript (at the end of section 2.3, T-Racing Design), the tool is "…available on the internal network of IZS-Teramo and is awaiting some technical adjustments and official approval from the Italian Ministry of Health before it can be integrated into the VETINFO portal. This integration will ensure secure access and the protection of sensitive data, as T-Racing inherits the robust GDPR techniques already applied in the portal." Moreover, the system is scalable and can be easily modified to adapt to the dynamic nature of available data/new data and the needs of Veterinary Services in addressing emergencies. All improvements will be based on near-real-time data from Italian information systems and tools to enhance the control and containment capabilities of Veterinary Services and decision-makers. These future improvements have been discussed and integrated into the Discussion section. Currently, there are no plans to include epidemic spread prediction.

Brucella spread usually has a strong temporal component. The lead up to the calving season is the highest risk time for spread of infection. Could a temporal window be applied to tracings to add a risk weighting to cohorts of animals residing together at these times? This could help narrow down the number of nodes. Currently, regarding the seed farm, outgoing movements can be limited to the time period right after the parturition (this is to focus on the movements with the highest risk of transmission). We assume that the user has access to the birth dates. We are also looking into adding more data, such as the birth dates of calves, in future versions of the application.

The Brucella case study cited genetic sequencing as an additional layer to confirming tracings. Has this been done with BTB in this study? In this study, integrating genetic sequencing with the bovine tuberculosis (BTB) case study was not feasible. Presently, the genetic information pertinent to BTB is not available in GenPat. However, efforts are underway to incorporate this data into the database, which holds promise for future investigations with T-Racing, which is automatically ready to handle and visualize such data.

The stated sensitivity of the skin test is at an animal level. See text in manuscript comments. we answered this in the manuscript comments.

The figures are quite small and pixelated. Perhaps this is just in the review version (pdf)? We have redone all the figures in higher resolution.

The following comments relate to editorial changes within the manuscript:

Abstarct
Line 10: "and lets" suggest change to "helping": Done
Line 11: "to safeguard" suggest change to "of safeguarding": Done
Line 13: "multiple simultaneously and" change to "multiple sources simultaneously at" Done
Line 14: delete "sources" Done
Line 14: "has been" change to "is" Done
Line 18: Delete "some" Done
Introduction
Line 27: "reduce" to "reduces" Done
Line 8, 33, 311,312, 320, etc. One Health or One-health or One-Health?! Stick with one! Done
Line 36 "data is" change to "data are" Done

Lines 48. 52. 57. 60. 63. etc. New paragraphs created unnecessarily. Remove paragraph breaks and associated indentation where not required. Done
Line 55. “require to rapidly” change to “require to be able to rapidly” Done
Line 63. “of disease spreading” suggest change to “within the spread of disease” Done

Line 69. “who” change to “that” Done
Line 70. “virus” change to “disease” Done
Line 72. “This could be an invaluable tool that allows” change to “This would be an invaluable tool allowing” Done
Line 76. “interacting” change to “interactive” Done

M&M

Line 80. “accounting for the” change to “utilizing” Done
Line 82. Delete “Along with” and don”t start a new paragraph. Done
Line 102. “(in Table 1 are listed all of the packages used)” change to “(Table 1 contains a list of all packages used) Done
Line 102 - 103 “The application lets manage large amounts of data from different information systems and share them efficiently and securely in real-time.” Change to “103 “ The application manages large amounts of data from different information systems and shares them efficiently and securely in real-time”. Done
Line 105. Delete “Indeed” Done
Line 105. Suggest change “differently” to “separate”: We have rephrased the sentence in “Differently from the serverless JavaScript web apps, computationally heavy services are executed on a remote server, and the Plumber package allows the application to query data sources in real-time, through web API.”
Line 106. Suggest change “let” to “allows” Done
Line 108. Change “makes sure the” to “facilitates” Done
Line 125. Change “out-breaks” to “outbreaks” Done
Line 127. Change “outbreaks” to “outbreak” Done

Results

Line 172. What is this? A law? “Through the DM 27 August 1994 n. 651 entered into force in Italy” yes, it is a low, we rephrased in "Through the Ministerial Decree of 27 August 1994 n. 651 …"
Line 176. “provides for control official serological test” change to “demands serological testing” Done
Line 259. “near” suggest change to “nearby” Done
Line 262. “let to establish” replace with “establishing” Done
Line 264. “(three of which, were outbreaks)”. I’m a bit confused by this. Do you mean 3 out of the 4 farms had confirmed outbreaks? We apologize for the confusion. We have reanalyzed the data, and in the meantime, the outbreak data has been updated in the database, so it has changed slightly. The description of the case study has been rewritten, and we believe it is now clearer.
Line 289. Is the whole herd tested at the same time? This has the effect of increasing the sensitivity of the test as it is effectively not a single animal test if the whole herd is restricted if a positive animal is found. This is an important consideration. According to EU rules, all animals aged more than 12 months are subject to testing, and they are tested once a year in the same time, except for large herds where more days can be needed, however, in any case, they must be consecutive days and the testing must be completed in the shortest time period possible, in any case no more than a week. Data about sensitivity are related to this testing regime, which is the same employed in the rest of EU countries (as described by De la Rua-Domenech et al., 2006)

Discussion

Line 310. “and outbreak management” Does this refer to the field staff? Maybe rewrite to clarify. Done
Line 317. “racing” suggest change to “racing against” Done
Line 325. “always explored” change to “always be explored” Done
Line 333. “So having data about traded animals” change to “Thus, having data on traded animals” Done
Line 353. “highlighted” change to “highlights” Done

Reviewer 2

Main comments

The manuscript does not provide sufficient information about the data and the methods. There is only an inventory of the sources of data and one sentence on the methodology. More details are needed to understand how the application manage and analyse the data to obtain outputs. Thank you for the suggestion. We have provided a thorough description of the data and methods in the Materials and Methods section, with dedicated sub-sections (2.1 Data sources and collection and 2.2 Temporal Network Analysis methods in T-Racing).

Regarding data, the authors should also discuss how the application complies with the General Data Protection Regulation (GDPR), given that various types of users are identified and will have access outputs (movements tracing of individual animals, genomic information) that are sensitive**.** This issue has been clarified in the text by adding the following sentences in M&M section (to the end of subparagraph 2.3 T-Racing Design): "Currently, it is available on the internal network of IZS-Teramo and is awaiting some technical adjustments and official approval from the Italian Ministry of Health before it can be integrated into the VETINFO portal. This integration will ensure secure access and the protection of sensitive data, as T-Racing inherits the robust GDPR techniques already applied in the portal"

The authors mentioned that the T-Racing application is currently in testing phase but no information is provided about this pilot study (see the specific comment below, Line 138). Besides, I suggest discussing the main results of these tests (i.e. strengths and weaknesses of the application) and provide feedback regarding the friendliness and relevance of the application, and how the results from the testing phase were used to improve the application. We have clarified in the text that T-Racing is ready to be used (see the previous response). Additionally, T-Racing application has not undergone a formal and structured testing phase but rather represents the evolution of previous applications developed by the same team with extensive usage in the Italian veterinary healthcare system. It is the outcome of collaborative work by a multidisciplinary team including epidemiologists, veterinarians, mathematicians, and computer scientists, who collectively contributed their expertise to its development and "testing phase". This concept has been clarified at the end of subparagraph 2.3 T-Racing Design.

Specific comments

Introduction

- Line 38: TRACES has become TRACES-NT (new technology) for few years: https://webgate.ec.europa.eu/IMSOC/tracesnt-help/Content/en/index.html. Done

- Line 45-60: Avoid 1-2 sentences paragraphs. All these sentences should be gathered in one paragraph about the interest of tracing disease spread for disease surveillance and control. Done

- Lines 61-63: Please expand this part about existing tools for animal tracing and explain what are the current limits of these tools or what features are missing (justifying the development and use of T-racing)? Done

- Lines 64-77: All these sentences should be gathered in one paragraph. Done

Materials and Methods

- Lines 85-90: Please provide a description of the data and datasets of these three databases (including spatial and temporal unit of the data and detailed information on livestock owners and producers). Done

- Line 97: Add references of previous studies using such methods for tracing disease spread. Describe how the temporal network analyses were conducted (including specifications about spatial and temporal units). We have included a subsection in the Materials and Methods section that explains this in detail: '2.2 Temporal Network Analysis methods in T-Racing.' This section provides information on how the temporal network analyses were conducted, including specifications about spatial and temporal units.

- Lines 100-109: All these sentences should be gathered in one paragraph. Done

- Line 110: Are the tools proposed in the T-Racing application available to all types of users? How does the application comply with the General Data Protection Regulation (GDPR)? Yes, the data used by T-Racing are sensitive, so only the authorized users (veterinarians, Veterinary Services, public health institutions, etc.,) can access through their credentials. This issue has been clarified in the text by adding the following sentences in the Materials and Methods section (to the end of subparagraph 2.3 T-Racing Design): "Currently, it is available on the internal network of IZS-Teramo and is awaiting some technical adjustments and official approval from the Italian Ministry of Health before it can be integrated into the VETINFO portal. This integration will ensure secure access and the protection of sensitive data, as T-Racing inherits the robust GDPR techniques already applied in the portal.".

Results

- Line 138: The testing phase is an important step in the development of a tool to ensure that it meets the needs of users. What does the testing phase consist of? Please provide some details about the number and types of users (authorities, operational actors, etc.). Which indicators are used for testing the application and evaluating its performances? Please, see the previous answer above

- Line 151 (and 158): please clarify the sentence: "and the red colour highlights paths followed by animals, singularly identified", (which?) "had gone out of the seed" This sentence has been completely rephrased as: 'Highlighting on the graph are all links involving the same animals that reached the seed (red links). ...' and made clearer by the detailed explanation of the T-Racing tool, which allows for highlighting within the network all edges involving animals moved from/to the seed based on the tracing strategy applied (see Materials and Methods section in 2.2 Temporal Network Analysis methods in T-Racing and 2.3 T-Racing Design sub-sections).

- Line 161: These examples seem to be based on real (not simulated) data. If so, replace "simulation Exercise" by "Case studies". If simulated data were used, please describe this step (methods, data, etc.) in the Materials and Method part. Thank you for the observation. Yes, it is correct that the data used are real, so we have replaced "simulation Exercise" by "Case studies" as suggested.

- Line 172 : Does DM mean Ministerial Decree ? If so, specify it on line 171 and use DM at each occurrence (e.g. line178). Otherwise, replace by "Ministerial Decree". Done

- Line 187: In this case study on bovine brucellosis, first you used a period of 2 years for tracing movements, and then, line 198, a period of 399 days to reduce the number of movements. How did you select these values? Please also explain more broadly in the Discussion how the application should be calibrated (species selection, time window, etc.) for tracing back and tracing forward movements depending on the diseases of interest. Is the application more appropriate for specific types of diseases (like, for example, slow spreading diseases)? The questions raised by the reviewer are legitimate. In the new version of the manuscript, we have emphasized that T-Racing is an application designed to make the job easier, provide flexibility, and make use of the user's knowledge. There are no universal rules, and each investigation has its unique characteristics. As explained in the text, the choice of depth (of levels and days) depends on various factors related to the specific disease being considered, such as contact, incubation and recovery rates, and the frequency of health checks carried out on the farms. Users may find additional information on other systems

or make a choice based on their knowledge of the disease. Indeed, the example provided is just one possible use case and does not constitute a legal report signed by tracing officers.

- Line 198: What are those three levels? The levels refer to degrees of separation. We have explained the methods of level and day depth to calibrate the network of movements in the Materials and Methods section, specifically in subsection 2.2 Temporal Network Analysis methods in T-Racing.

- Line 204-205: “The reduced network, focusing on outbreaks within the network, “ Please, clarify what these two networks refer to? These are not two distinct networks, but one (the reduced network) is a subset of the other. The sentence has been rephrased to: 'The reduced network, created by keeping only temporally valid paths among involved outbreaks within the network.' The details of this network reduction method are explained in the Materials and Methods section.

- Line L209 : Write brucela abortus in italics. Done

- Line 214: The application enables search on “individually identified animals”. Such outputs are certainly sensitive information and I am expecting that this will be a barrier to the use of the tool. Can you please explain how the application deals with this issue? Authorized users with access to T-Racing have no restrictions or limitations. Please refer to the previous answer.

- Figures 3, 4, 5 and 7 are too small to be readable. Done

- Line 254 : “examples” Done

- Figure 6: What does the black link mean? in the figure, the black link represents the movement of animals that do not come from seeds, unlike the red links.

- Line 278: What tool do you refer to in the sentence “we employed a tool” We rephrased the sentence to: 'For a more focused investigation, we considered only temporally valid paths connecting outbreaks, which resulted in a subnetwork of...' The tool referred to is the network reduction method, which is explained in the Materials and Methods section.

Discussion

- Line 305-307: This sentence is exactly the same as the first one of the Introduction! The Discussion session has been completely rewritten, and all redundancies present in the previous manuscript version have been eliminated.

- Line 312-322: This is not Discussion. I suggest moving this paragraph to the Introduction to underline the interest of One Health in disease surveillance, and highlight how tracing disease spread is critical for effective disease control. Done

- Figure 8: This figure would be better positioned in the Results part to demonstrate the relevance of T-Racing in preventing and detecting diffusion pathways versus the simple farm isolation, using a retrospective analysis of data. In Figure 8, please describe more specifically the detection and prevention of cases when T-racing is used versus without T-Racing (=surveillance program only). Letters of the alphabet could be used to distinguish the four farms and explain the chain of events regarding the disease transmission between farms (disease introduction and spread) and detection (through surveillance program and using T-Racing). Specify what is the “surveillance programme”: programmed surveillance (serology twice a year) or abortion notification? What do the yellow arrows mean? Besides, I suggest using a darker colour than yellow to be more visible on the figure. Thank you for the valuable suggestion. We have included this part in a dedicated subparagraph (2.2 Temporal Network Analysis methods in T-Racing) in the Materials and Methods section. We have modified the figure according to your indications. Regarding what we mean by 'surveillance

program,' this has been clarified in the dedicated subparagraph '2.5 Brucellosis and Tuberculosis: Rationale for Case Studies and Italian Regulations.

- Lines 333-337: These sentences (at least the main idea) should be in the Introduction to explain the interest of developing and using the T-Racing tool versus getting an extraction of the movement network through a query of the BDN (National Database for Animal Registration)? Thank you for the suggestion. We have moved this information to the Introduction and in the Discussion.

- L356-357: How is this sentence related to the topic of the paper? How the tool can foster collaboration between sectors? In the updated version of the paper, it should be clearer the practical utility of the application within the One Health framework for all stakeholders involved. We described how the application streamlines the work of epidemiologists, leading to prompt and more efficacious interventions by veterinary services. This, in turn, enables the containment of disease spread in animals and diminishes the risk of zoonotic transmission to humans.

- Line 367: What do you mean by “reliable data”? What criteria should be taken into account to consider that data is reliable? Thanks Thank you for the question. In our context, 'reliable data' means accurate, consistent, and timely information that is free from significant errors or biases. It's crucial to have a genuine and precise representation of a phenomenon, even if it's not entirely exhaustive, rather than relying on erroneous data that could lead to incorrect interpretations of the results. We achieve this by linking T-Racing to established and continuously updated national databases. Despite the occasional incompleteness, such as delays in timely data updates (e.g., for bovine movements, which may be almost a week behind), these databases offer verified and regularly maintained data.

Additional comments

- Can the tool be used to trace emerging diseases (so-called Disease X)? Yes, the tool can be used to trace emerging diseases (so-called Disease X) if the data for Disease X are entered into the databases linked to T-Racing.

- Can the tool be used by other countries with similar data and if so, how would the tool be made available? Currently, it's challenging to imagine other countries using the application, but it could be possible in the future. The main issue is that the application's data structure is tailored specifically for use in Italy. It's a complex and diverse structure, and the effort to standardize it was done only for Italian data. Another concern is the architecture of the application: it's not a simple "shiny" app, but rather requires databases and REST API services, necessitating the establishment of an IT infrastructure to support its operation. Because of this, we mentioned in the section about future developments that we will be working on a more universal version of the application. This version will have its source code available on GitHub and will also support the import of user’s data.

Reviewer 3

Introduction: It’s partially vague. As a veterinarian and epidemiologist, I understand the problem, but what is state-of-the-art, and why is your tool unique? What motivated you to create this tool? Please also remove redundancy in several phrases. Be more explicit about the objectives of the work. Thank you for your insightful feedback. We have made significant revisions to the introduction to address your concerns. Specifically, we have added a section that discusses existing applications and methodologies used for tracing disease spread, highlighting the current state-of-the-art. Additionally, we have clearly articulated what makes T-Racing unique, including its ability to integrate diverse data sources, its user-friendly interface, and its scalability. Moreover, we have elaborated on the motivation behind creating T-Racing, emphasizing the need for a robust tool to aid in the rapid and effective control of animal disease outbreaks and its capability to guide users in epidemiological investigations by adopting a data-driven approach, via an iterative analysis

while leveraging their expertise. The objectives of our work have been clearly stated to provide a comprehensive understanding of the study's aims. Furthermore, the text has been reorganized to enhance its flow, a logical progression of ideas and eliminating redundancy.

Methods: It's vague. The methodology (the tool) is the heart of your work; if we need to help to understand your tool clearly, how would we know its usefulness? Please restructure this part into sections and subsections. For example, the section on the tool should have an explanation of each module. The section on your experiments should provide details of the disease models and regions. Check for inspiration and organisation of the work via other methods/tools papers published in Plosone. For example: https://journals.plos.org/plosone/article?id=10.1371/journal.pone.0199960 We appreciate the opportunity to improve the clarity and organization of our Methods and Materials section. We have restructured this section into subsections and provided detailed explanations for each component based on your suggestions.

Results: There is a mix between methods and results. Calrity and reorganisation is necessary. We have carefully reviewed the Results section and have reorganized it to improve clarity. Any content that was more appropriate for the Materials and Methods section has been relocated accordingly.

Discussion: It's an introduction and not a discussion. I dont see how the results are original compared to simillar works and tools in Europe. Their originality, limitations and perspectives. This section needs most reworking. We have completely revised the Discussion section to clearly distinguish it from the Introduction. The Discussion now focuses on interpreting our results, highlighting the original contributions of our study, discussing its limitations, and proposing future research directions.

Other questions:

Is the code of the R shiny app available? The application is currently based on a specific data structure used in Italy and has a complex architecture with databases and rest API services. Because of this, we haven't yet worked on making the application available for general use, as it was developed for restricted use in Italy. However, we plan to work on a more generic version of the application, and its source code will be available on GitHub. This version will also allow for the import of own data, as stated in the discussion.

How the tool was tested with end-users and co-created with end-users? As indicated in our previous response, T-Racing application has not undergone a formal and structured testing phase but rather represents the evolution of previous applications developed by the same team with extensive usage in the Italian veterinary healthcare system. It is the outcome of collaborative work by a multidisciplinary team including epidemiologists, veterinarians, mathematicians, and computer scientists, who collectively contributed their expertise to its development and "testing phase". We have provided clarification on this in the Methods and Materials section, in subsection 2.3 titled "T-Racing Design"

Why is the tool a demo and not operational? T-Racing is ready to use and is operational for epidemiologists and veterinarians who work inside our Public Health Institute. This issue has been clarified in the text by adding the following sentence in the Materials and Methods section (to the end of subparagraph 2.3 T-Racing Design): "Currently, it is available on the internal network of IZS-Teramo and is awaiting some technical adjustments and official approval from the Italian Ministry of Health before it can be integrated into the VETINFO portal. This integration will ensure secure access and the protection of sensitive data, as T-Racing inherits the robust GDPR techniques already applied in the portal."

The tool allows us to assess the risk based on animal movements only. What about human movements and contaminated products? T-Racing is a scalable system, and if structured data on human movements were available, we could integrate tailored tools for their representation and analysis. Similarly, regarding contaminated products, we could implement specific features in the tool to analyze and assess their risk if

relevant data were accessible. Structured and updated data on human movements are not readily available, and their availability often depends on personal choices. This aspect would lead to a broader discussion. Concerning contaminated products, information on these could be incorporated, and if a national system for collecting such data becomes available in the future, our scalable system could easily accommodate it within T-Racing.

For example, how would it be for African swine fever? in the case of African swine fever (ASF), the management of disease outbreaks is handled by the SIMAN system, while T-Racing has been used to monitor pig movements, providing valuable insights into the disease spread and identifying risks for large Italian pig-producing companies. Indeed, there are ASF-infected wild boar cases available in SIMAN and by tracing with the spatial buffer tool, we can evaluate the impact of the virus introduced in nearby farms on the trade network. in the Based on this evidence, our institute's epidemiologists were engaged by the Ministry of Health in a task force to define appropriate control and containment plans.

You mention the tool is important from a public health perspective. How do you envisage using it with human data and human outbreaks of Brucellosis and Tuberculosis, for example? T-Racing is intricately linked to National Databases. If these databases were to include human cases, we could seamlessly utilize T-Racing as it currently stands to pinpoint potential disease sources or transmission between animals and humans. The tool allows for the selection of geographical proximity from outbreak sites (buffer from the outbreak). See also the following answers.

How do you envisage using the tool for movements originating outside Italy? Origin EU and outside EU, for example? Our tool, which incorporates data from the Animal Movement Database (NDB), contains information on movements to and from abroad, encompassing both EU and non-EU countries. This is reported in M&M subsection "Data source and collection".

In the introduction and discussion, you discuss the importance of your tool for One Health; this term is overused because I would like to see more explicitly how the tool adds to the human and environmental Health presented in the methods and the results. Say it clearly if the tool is just for animal health and the perspectives are to enlarge to One Health. We acknowledge the reviewer's feedback regarding the excessive use of the word "OneHealth" in the previous version of the manuscript, and have since reduced its frequency. However, it's essential to emphasize that achieving a state of "one health" requires concerted efforts in animal, environmental, and human health. This doesn't necessarily mean employing a method that addresses all aspects simultaneously. Taking steps to prevent the spread of diseases transmitted from animals, which could potentially affect humans if left uncontrolled, involves actions aimed at safeguarding animal health for the protection of humans. This approach is central to the One Health perspective.

Reviewer 4

First, it is very poorly written, and it needs a substantial restructuring (see further comments). Second, it is hard to evaluate the tool without having access to it. I would suggest the author to resubmit the manuscript once a demo of the tool is available online (or provide it) and with a dummy dataset (i.e. made-up network/farms/outbreaks) to allow running the scripts. Thank you for your comments/suggestions. We revised deeply the manuscript and made a demo application available with anonymized datasets for public use.

Comments:
1. The “Introduction” section is poorly written, it is a collection of statements without a logic flow. Please rewrite. The whole manuscript has been revised.
2. Lines 94-96: please report how the tool complies with the privacy laws (GDPR, etc). This is addressed in M&M (2.3 T-Racing Design).

3. Lines 97-98: “Temporal Network Techniques” is too generic, please say which ones and provide references. We added a new dedicated paragraph in M&M (2.2 Temporal Network Analysis methods in T-Racing).
4. Table 1: in my understanding R packages sp and rgdal might not going to be maintained anymore, their functionalities could be found in sf and terra. I’d suggest to investigate this issue and eventually change the code. The reviewer's observation is accurate. However, when the spatial trajectory drawing functions for T-Racing were developed, the packages were still being maintained. Because the usage of the packages is limited to a few basic spatial functions, the authors chose not to translate the code into sf (the terra package is not required by T-Racing). However, the shareable version of the application we are going to develop will utilize the new sf package.
5. Why does Fig 2 not show a point in the Marche region where the brucellosis outbreak was detected? If it is because the outbreak was traced back between Sicily and Tuscany? If this is the case, please add some indication on where the outbreak was found (Marche?), otherwise it is very confusing. We improved Fig.2 (now Fig 6) as suggested and add a red arrow on the map pointing to the seeding farm.
6. In general, figures are not consistent with the text and look randomly placed. All figures have been improved in resolution and placed accordingly to the text.
7. Paragraph 3.1.1 (and 3.1.2): the first describing general information about brucellosis (and bTB) should go into introduction or material and methods. We do agree, added a dedicated paragraph (Brucellosis and Tuberculosis: Rationale for Case Studies and Italian Regulations)
8. Lines 196-199: it is not clear how you narrowed down from 386 to 38 farms (and 1826 to 199 movements). Which type of tracing was done? Is it going to be available in T-Racing? We described in M&M the tools and the steps to achieve the results reported in figures.
9. Lines 278-280: similarly, which tool was used to connect “outbreaks”? With which criteria movements/farms were excluded? See above answer.
