# Supplementary material for: T-Racing: a modern tool for supporting epidemiological investigation in animal disease outbreaks in Italy
Source: PLoS One. 2025 Feb 19;20(2):e0317313. doi: 10.1371/journal.pone.0317313 (PMC11838889; doi:10.1371/journal.pone.0317313)
Supplement: Attachment — Submitted filename: PONE-D-24-01354_R2_reviwer_ea_2024_09_23.pdf [file pone.0317313.s005.pdf]

PLOS ONE

# T-Racing: a modern tool for supporting epidemiological investigation in animal disease outbreaks in Italy

--Manuscript Draft--

| | |
|---|---|
| Manuscript Number: | PONE-D-24-01354R2 |
| Article Type: | Research Article |
| Full Title: | T-Racing: a modern tool for supporting epidemiological investigation in animal disease outbreaks in Italy |
| Short Title: | T-Racing epidemiological web application |
| Corresponding Author: | Luca Candeloro, M.D.<br>Istituto Zooprofilattico Sperimentale dell'Abruzzo e del Molise G Caporale<br>Teramo, ITALY |
| Keywords: | One Health; Multidisciplinary approach; epidemiology; Tracing; Temporal Network Analysis; Shiny web application; Outbreak investigation; Zoonoses management. |
| Abstract: | The One Health approach unites efforts across human-animal-environment interfaces against shared threats like zoonotic diseases. T-Racing is a Shiny web application, that supports epidemiological investigations and helps contain livestock-related disease spread, aligning with multidisciplinary principles to safeguard public health. The application uses Temporal Network Analysis techniques to address the dynamic nature of animal trade, facilitating backward and forward tracing strategies. T-Racing leverages web services to retrieve data from multiple sources simultaneously and in near real-time through the plumber package and is distributed using Shinyproxy. Indeed, T-Racing manages and analyze extensive and diverse datasets within the same environment, including animal movement data, disease outbreak data, and genomic data, all obtained from Italian National databases. In this work, we show T-Racing's capabilities by simulating epidemiological investigations of brucellosis and tuberculosis outbreaks that occurred in non–endemic areas of Italy. This tool supports epidemiological investigations by adopting a data-driven approach, guiding users through the analysis via an iterative process while leveraging their expertise. Therefore, it enables faster data analysis, improves understanding of disease transmission patterns, and facilitates prompt and targeted interventions. |
| Order of Authors: | Luca Candeloro, M.D. |
| | Lara Savini |
| | Annamaria Conte |
| | Paolo Calistri |
| | Diana Palma |
| | Samuel Perticara |
| | Anna Cecilia Trolesi Reis Borges Costa, PhD |
| | Alessio Di Lorenzo |
| | Susanna Tora |
| | Sara Serrani |
| | Michela Toro |
| | Karina Diniz Baumgarten, PhD |
| | Fabrizio De Massis |
| Response to Reviewers: | Please, find responses to reviewer in the attached word file "T-Racing-Response to reviewers comments.doc" |
| Additional Information: | |
| **Question** | **Response** |

| | |
|---|---|
| **Financial Disclosure**<br><br>Enter a financial disclosure statement that describes the sources of funding for the work included in this submission. Review the submission guidelines for detailed requirements. View published research articles from *PLOS ONE* for specific examples.<br><br>This statement is required for submission and **will appear in the published article** if the submission is accepted. Please make sure it is accurate.<br><br>**Funded studies**<br>Enter a statement with the following details:<br>• Initials of the authors who received each award<br>• Grant numbers awarded to each author<br>• The full name of each funder<br>• URL of each funder website<br>• Did the sponsors or funders play any role in the study design, data collection and analysis, decision to publish, or preparation of the manuscript?<br><br>Did you receive funding for this work? | Yes |
| Please add funding details.<br>as follow-up to "**Financial Disclosure**<br><br>Enter a financial disclosure statement that describes the sources of funding for the work included in this submission. Review the submission guidelines for detailed requirements. View published research articles from *PLOS ONE* for specific examples.<br><br>This statement is required for submission and **will appear in the published article** if the submission is accepted. Please make sure it is accurate. | This research was funded by Italian Ministry of Health (MSRCTE0219). The funders had no role in study design, data collection and analysis, decision to publish, or preparation of the manuscript. |

| | |
|---|---|
| **Funded studies**<br>Enter a statement with the following details:<br>• Initials of the authors who received each award<br>• Grant numbers awarded to each author<br>• The full name of each funder<br>• URL of each funder website<br>• Did the sponsors or funders play any role in the study design, data collection and analysis, decision to publish, or preparation of the manuscript?<br><br>Did you receive funding for this work?" | |
| Please select the country of your main research funder (please select carefully as in some cases this is used in fee calculation).<br>as follow-up to "**Financial Disclosure**<br><br>Enter a financial disclosure statement that describes the sources of funding for the work included in this submission. Review the submission guidelines for detailed requirements. View published research articles from *PLOS ONE* for specific examples.<br><br>This statement is required for submission and **will appear in the published article** if the submission is accepted. Please make sure it is accurate.<br><br>**Funded studies**<br>Enter a statement with the following details:<br>• Initials of the authors who received each award<br>• Grant numbers awarded to each author<br>• The full name of each funder<br>• URL of each funder website<br>• Did the sponsors or funders play any role in the study design, data collection and analysis, decision to publish, or preparation of the manuscript?<br><br>Did you receive funding for this work?" | ITALY - IT |
| **Competing Interests** | The authors have declared that no competing interests exist |

*Powered by Editorial Manager® and ProduXion Manager® from Aries Systems Corporation*

| | |
|---|---|
| Use the instructions below to enter a competing interest statement for this submission. On behalf of all authors, disclose any competing interests that could be perceived to bias this work—acknowledging all financial support and any other relevant financial or non-financial competing interests.<br><br>This statement is required for submission and **will appear in the published article** if the submission is accepted. Please make sure it is accurate and that any funding sources listed in your Funding Information later in the submission form are also declared in your Financial Disclosure statement.<br><br>View published research articles from *PLOS ONE* for specific examples.<br><br>**NO authors have competing interests**<br><br>Enter: *The authors have declared that no competing interests exist.*<br><br>**Authors with competing interests**<br><br>Enter competing interest details beginning with this statement:<br><br>*I have read the journal's policy and the authors of this manuscript have the following competing interests: [insert competing interests here]*<br><br>* typeset | |
| **Ethics Statement**<br><br>Enter an ethics statement for this submission. This statement is required if the study involved:<br><br>• Human participants<br>• Human specimens or tissue<br>• Vertebrate animals or cephalopods<br>• Vertebrate embryos or tissues | N/A |

Powered by Editorial Manager® and ProduXion Manager® from Aries Systems Corporation

| | |
|---|---|
| • Field research<br><br>Write "N/A" if the submission does not require an ethics statement.<br><br>General guidance is provided below. Consult the submission guidelines for detailed instructions. **Make sure that all information entered here is included in the Methods section of the manuscript.** | |

Powered by Editorial Manager® and ProduXion Manager® from Aries Systems Corporation

| | |
|---|---|
| **Format for specific study types**<br><br>**Human Subject Research (involving human participants and/or tissue)**<br>• Give the name of the institutional review board or ethics committee that approved the study<br>• Include the approval number and/or a statement indicating approval of this research<br>• Indicate the form of consent obtained (written/oral) or the reason that consent was not obtained (e.g. the data were analyzed anonymously)<br><br>**Animal Research (involving vertebrate animals, embryos or tissues)**<br>• Provide the name of the Institutional Animal Care and Use Committee (IACUC) or other relevant ethics board that reviewed the study protocol, and indicate whether they approved this research or granted a formal waiver of ethical approval<br>• Include an approval number if one was obtained<br>• If the study involved *non-human primates*, add *additional details* about animal welfare and steps taken to ameliorate suffering<br>• If anesthesia, euthanasia, or any kind of animal sacrifice is part of the study, include briefly which substances and/or methods were applied<br><br>**Field Research**<br><br>Include the following details if this study involves the collection of plant, animal, or other materials from a natural setting:<br>• Field permit number<br>• Name of the institution or relevant body that granted permission | |
| **Data Availability**<br><br>Authors are required to make all data underlying the findings described fully available, without restriction, and from the time of publication. PLOS allows rare exceptions to address legal and ethical concerns. See the PLOS Data Policy and FAQ for detailed information. | Yes - all data are fully available without restriction |

Powered by Editorial Manager® and ProduXion Manager® from Aries Systems Corporation

| | |
|---|---|
| A Data Availability Statement describing where the data can be found is required at submission. Your answers to this question constitute the Data Availability Statement and **will be published in the article**, if accepted.<br><br>**Important:** Stating 'data available on request from the author' is not sufficient. If your data are only available upon request, select 'No' for the first question and explain your exceptional situation in the text box.<br><br>Do the authors confirm that all data underlying the findings described in their manuscript are fully available without restriction? | |
| **Describe where the data may be found in full sentences. If you are copying our sample text, replace any instances of XXX with the appropriate details.**<br><br>• If the data are **held or will be held in a public repository**, include URLs, accession numbers or DOIs. If this information will only be available after acceptance, indicate this by ticking the box below. For example: *All XXX files are available from the XXX database (accession number(s) XXX, XXX.)*.<br>• If the data are all contained **within the manuscript and/or Supporting Information files**, enter the following: *All relevant data are within the manuscript and its Supporting Information files.*<br>• If neither of these applies but you are able to provide **details of access elsewhere**, with or without limitations, please do so. For example:<br><br>*Data cannot be shared publicly because of [XXX]. Data are available from the XXX Institutional Data Access / Ethics Committee (contact via XXX) for researchers who meet the criteria for access to confidential data.*<br><br>*The data underlying the results presented in the study are available from (include the name of the third party* | All data used are publicly available, a representative sample of these datasets has been anonymized and spatial coordinates have been noised and rounded to the second decimal to be included in the demo version, publicly accessible at the link: https://demo.izs.it/app_direct/tracing/. |

| | |
|---|---|
| *and contact information or URL).*<br>• This text is appropriate if the data are owned by a third party and authors do not have permission to share the data.<br><br>* typeset | |
| Additional data availability information: | |

Manuscript

Click here to access/download;Manuscript;Manuscript_T-Racing.docx

*Article*

# T-Racing: a modern tool for supporting epidemiological investigation in animal disease outbreaks in Italy

**Luca Candeloro [1], Lara Savini [1], Annamaria Conte [1], Paolo Calistri [1], Diana Palma [1], Samuel Perticara [1], Anna Cecília Trolesi Reis Borges Costa [2*], Alessio di Lorenzo [1], Susanna Tora [1], Sara Serrani [1], Michela Toro [1], Karina Diniz Baumgarten [2, 3] and Fabrizio De Massis [1]**

[1] Istituto Zooprofilattico Sperimentale dell'Abruzzo e del Molise "G.Caporale", Italy
[2] Departamento de Medicina Veterinária, Escola de Zootecnia e Medicina Veterinária, Universidade Federal de Lavras, Lavras Minas Gerais, Brazil.
[3] State Department of Animal Health. Companhia Integrada de Desenvolvimento Agrícola de Santa Catarina – CIDASC, Florianópolis, SC, Brazil

[*] Correspondence: anninha_veterinaria@hotmail.com

**Abstract:** The One Health approach unites efforts across human-animal-environment interfaces against shared threats like zoonotic diseases. T-Racing is a Shiny web application, that supports epidemiological investigations and helps contain livestock-related disease spread, aligning with multidisciplinary principles to safeguard public health. The application uses Temporal Network Analysis techniques to address the dynamic nature of animal trade, facilitating backward and forward tracing strategies. T-Racing leverages web services to retrieve data from multiple sources simultaneously and in near real-time through the plumber package and is distributed using Shinyproxy. ~~Indeed,~~ T-Racing manages and analyze extensive and diverse datasets within the same environment, including animal movement data, disease outbreak data, and genomic data, all obtained from Italian National databases. In this work, we show T-Racing's capabilities by simulating epidemiological investigations of brucellosis and tuberculosis outbreaks that occurred in non–endemic areas of Italy. This tool supports epidemiological investigations by adopting a data-driven approach, guiding users through the analysis via an iterative process while leveraging their expertise. Therefore, it enables faster data analysis, improves understanding of disease transmission patterns, and facilitates prompt and targeted interventions.

**Keywords:** One Health; Multidisciplinary approach; Epidemiology; Tracing; Temporal Network Analysis; Shiny web application; Outbreak investigation; Zoonoses management.

## 1. Introduction

Preventing and controlling communicable diseases in livestock through early detection of outbreaks increases the effectiveness of the response and reduces the social, economic, and environmental costs associated with these events [1, 2]. In Italy, veterinary surveillance programs target several zoonotic diseases in livestock, including brucellosis, tuberculosis, leptospirosis, rabies, salmonellosis, campylobacteriosis, listeriosis, and echinococcosis etc. Animals serve as an excellent proxy for monitoring both novel and known zoonotic pathogens given that more than 60% of emerging infectious diseases in humans originate from animal sources [3-5]. This underscores the critical role of animal health in implementing of the One Health approach, by detecting, monitoring, and predicting health hazards that could also impact environmental and human health since

some pathogens can survive long periods in the environment [6]. Among the diseases that could affect human health are tuberculosis and brucellosis, both chronic infectious diseases caused by bacteria. Bovine tuberculosis, caused by *Mycobacterium bovis* [7], is a zoonotic disease that was estimated to have a human prevalence varying from 0.4% to 76.7% worldwide [8]. Animal brucellosis caused by *Brucella* spp., is a zoonosis of utmost significance, since it was projected to globally affect 2.1 million people by year [9]. The control of both diseases is anchored on the surveillance and control in animals, with the traceability of animal cases being of paramount importance to prevent human infections, especially for human brucellosis that does not have vaccination.

In this context, animal movement is often considered a major risk for disease spreading between herds [10] and to humans when zoonoses are involved. Hence, livestock transportation data is registered in national and international databases to facilitate contact tracing in the event of an outbreak [11-15]. An example of an international official movements database is TRACES-NT (TRAde Control and Expert System New Technology). TRACES-NT is an information platform of the European Commission for the reporting, certification, and control of imports of animals, origin of animal products, food and feed of plants, and other products of veterinary interest. ~~t is only accessible to competent authorities and is considered sensitive data.~~ The system is only accessible to competent authorities and is considered sensitive data. It also registers trade and exports of animals and certain products of animal origin into the EU and intra-EU. Currently, TRACES-NT is the integrated computerized veterinary system with a single architecture provided in Decisions 2003/24/EC and 2004/292/EC (EU, 2019) [16, 17]. The use of animal movement data facilitates tracing the spread of diseases within animal populations and the potential routes of exposure being critical for effective disease control and crucial in the final steps of eradication programs [18]. Contact tracing plays a pivotal role in identifying the necessary actions to break the transmission chain, to reduce the spread and duration of infectious diseases, thus preventing new cases [19]. When zoonoses are considered, this also means protecting public health, once contact tracing has the subsequent effect of providing assessments to stakeholders which is useful for quickly identifying and implementing containment, surveillance, and control systems.

Technology plays a fundamental role in developing online tracing applications, that can improve the results of epidemiological investigations. Advanced analytics and link analysis can be used to uncover hidden patterns in the spread of diseases [2, 20]. Therewith, tracing applications have been developed to assist animal health officials in quickly identifying the animals involved in an outbreak, where and when the infected animals are located, and whether other susceptible animals might have been exposed to the disease. These actions would be taken by uncovering the missing or unexpected links from the contact details, identifying the animals that should undergo testing, tracking the geographical spread of the disease, and determining which farms are at the highest risk. Thus, tracing applications can help in disease control and surveillance during critical outbreak stages, saving time, preventing further spreading, minimizing costs and reducing the consequences of the disease outbreaks [2, 21]. While tracing applications enables veterinary services, epidemiologists, and competent authorities to respond quickly by identifying the "hot spots" and implementing effective targeted control and eradication measures. Although it may sound simple, the tracing application can become overwhelmingly complex and resource-intensive, however, using data visualization and analytics can significantly improve the performance and effectiveness, making the application an invaluable tool. Currently, due to the COVID-19 global pandemic, contact tracing strategies have been widely used and many contact-tracing web apps have been

developed for this purpose in human health [22, 23]. In veterinary epidemiology, there is limited literature on contact tracing web applications, with notable examples being EpiContactTrace [2] and EpiExploreR [24]. EpiContactTrace, an open-source R package, allows end users to conduct forward and backward contact tracing by analyzing livestock movement data. However, it requires technical knowledge in R programming, manipulation and adjustment of data, which limits its accessibility for many users having different expertise. Additionally, it lacks of integration of heterogeneous and epidemiologically relevant data necessary for a comprehensive epidemiological investigation. EpiExploreR, an R shiny web application, offers tools for disease mapping, spatiotemporal analysis, and integration of diverse datasets, but its complexity need for specialized expertise make it less accessible for field operators and veterinarians who require immediate and user-friendly contact tracing tools. Moreover, both tools lack effective management of large networks, which is crucial for avoiding chaotic and unusable results.

Even though there are tracing applications already developed in the veterinary field, there is still a need for a versatile and user-friendly web tool that can manage various and heterogeneous datasets and incorporate analytical features to quickly perform forward and backward tracing within a spatial-temporal environment. Therefore, the aim of this paper was to introduce T-Racing, a web-based operational tool developed to assist veterinary epidemiological investigations in Italy. Unlike existing tools that may require specialized knowledge or are limited by complexity, T-Racing is designed to be easily accessible to local and national authorities, working in public health across various roles, including veterinary and public health institutes, veterinary services, and epidemiologists. Based on our knowledge, T-Racing offers several distinctive features that overcome the limitations of existing applications. These include its ability to integrate diverse data sources, a user-friendly interface, and scalability. It integrates certified data directly from national databases, ensuring that the underlying data is both reliable and easily interpretable. This is possible because of the tool's foundation in a deep knowledge of the data and the Italian animal trade system. T-Racing allows users to explore and analyze the complex spatiotemporal pathways that shape disease spread enabling them to draw meaningful conclusions based on a data-driven, user-guided approach.

## 2. Materials and Methods

T-Racing is an application providing users with flexibility in analyzing epidemiological data by facilitating the use of specific methods and tools through an interacting interface. The main objective of T-Racing is to support veterinary epidemiological investigations in Italy by integrating and visualizing data such as outbreaks, animal movements, and other relevant information. Its flexibility lies in the ability to handle various types of epidemiologically useful data, allowing users to perform both forward and backward tracing within a spatial-temporal framework. This capability enables users to tailor analyses to their specific needs and draw meaningful conclusions based on comprehensive data integration.

### *2.1 Data sources and collection*

T-Racing gathers near-real-time data from Italian National Databases which include disease notified outbreaks obtained from the National Information System for the Notification and Management of Animal Diseases (SIMAN) which manages data on more than 100 diverse animal diseases, including both zoonotic and non-zoonotic diseases [25], animal movements records extracted from the Italian National Database for Animal Identification and Registration (NDB) [26], and genomic data, such as Multi Loci Sequence

Type (MLST), provided by National Reference Centre for Whole Genome Sequencing of microbial pathogens database and bioinformatics analysis (GenPat) [27, 28]. These systems can be accessed through the VETINFO portal (https://www.vetinfo.sanita.it/), which is a secure platform to collect and present data for managing Italy's Animal Health and Food Safety system. VETINFO provides unified access to various stakeholders, including public health institutes, veterinary services, farmers, and competent authorities. To ensure user trust and regulatory compliance, the portal strictly adheres to General Data Protection Regulation (GDPR) compliance techniques, requiring users to access it with personal credentials and mandating specific functionalities for transparent and secure data reporting. The management of these databases is the responsibility of the Istituto Zooprofilattico Sperimentale dell'Abruzzo e del Molise "G. Caporale" (IZS-Teramo) on behalf of the Italian Ministry of Health.

T-Racing collects detailed data for each reported suspected or confirmed outbreak, including the disease name (i.e., brucellosis, tuberculosis, leptospirosis, rabies, salmonellosis, campylobacteriosis, listeriosis, foot and mouth disease, african swine fever, blutongue, etc.), unique outbreak identifier code, geographical coordinates (which are related to the outbreak location that can be a farm or another site where the infected case was identified by the veterinary service), region, province, administrative unit, ~~suspected and confirmed~~ outbreak date, outbreak state (i.e., suspect, extinct, and confirmed), and species involved (including animal, vector species such as mosquitoes and flies, and birds, etc.). Animal movement data includes the origin and destination unique holding identifier codes, animal single identification codes or batches of traded animals per species (including horses, cattle, buffalo, pigs, sheep and goats), and the date of the movement. The system also considers various holding attributes (livestock properties) for both origin and destination, such as the holding type (e.g., farm, slaughterhouse, staging point, transit station, market, assembly centre, pasture, foreign country, etc.), production type (e.g., milk, meat, wool, reproduction, etc.), address, and geographical coordinates. Additionally, in terms of genomic data, T-Racing records the holding code, animal single identification code, Sequence Type number, and date of sampling. Furthermore, T-Racing relies on data collected over the past 7 years extracted from the databases through daily scheduled procedures.

### *2.2 Temporal Network Analysis Methods in T-Racing*

The application uses Temporal Network Analysis techniques [29] to address the dynamic nature of animal trade, enabling quick and efficient Backward and Forward Contact Tracing strategies (BCT and FCT). In the T-Racing environment, the animal trading network is represented as a time-ordered directed and weighted network, where holdings are the nodes, animal movements between holdings on a given day are the edges, and the number of moved animals is the weight (see Fig 1 on the left).

Animal movements are recorded in the NDB at the administrative farm owner (farmer) level. Consequently, multiple farmers from the same holding may be represented as different nodes (see Fig 1, top right), causing the network to be artificially fragmented. This fragmentation significantly impacts the analysis of diseases affecting multiple animal species. To address this issue, the application allows for network aggregation, ensuring that each node represents the physical holding, i.e. the geographical location to where the animals transited. Thus, a node can represent multiple owners at a fiscal level (see Fig 1, bottom right).

The framework of time-ordered networks, essentially directed acyclic graphs [29], is useful for understanding flow dynamics [30], particularly in the context of diseases spread through direct contact. We assume diseases spread along paths driven by animal movements, so accounting for the direction and timing of these movements allows us to

detect temporally valid paths, providing a likely estimation of disease spread, similar to the Susceptible-Infection network-based compartmental model (where the contact rate is one). Direction and the timing of animal movements are important for detecting disease spread because they highlight impossible paths, and or too-long time elapsed for short paths [30, 31]. For example, in Fig 1, if farm A is infected at time zero, it can infect holding B, which can then infect holding C through subsequent animal movements. Since the probability of disease transmission decreases with increasing degrees of separation, we typically limit the search for plausible paths to a maximum degree of separation, or "level depth." Depending on the disease, the time elapsed between events might be too long to assume that nodes remain infectious and undetected, requiring a restriction of the time horizon to a specific number of days, known as "temporal depth." The choice of depth, in terms of both days and levels, affects the resulting subnetwork and depends on factors such as the disease's infectiousness, incubation period, and the timing of surveillance system controls. Furthermore, the removal of node(s) and/or edge(s) from a time-ordered directed network involves the change of the resulting paths. Therefore, each network modification requires a recalculation of the paths.

Contact tracing following a positive detection has been widely used in various fields, with different interpretations and applications depending on the context [30, 32]. In T-Racing, FCT, BCT, and a combination of both (FCT-BCT) strategies have been implemented to support track and control the spread of diseases. These methods provide different perspectives and insights depending on the context and time of disease outbreaks.

**FCT method** focuses on identifying the pathways of pathogen spread starting from an index node, known as the seed, and an event date. By analyzing outgoing movements within a specified depth (in terms of days and levels) FCT highlights the nodes that the disease could have potentially reached. Here, levels represent the degrees of separation from the seed node, where each level corresponds to a different stage of contact tracing (e.g., direct contacts at Level 1, and contacts of contacts at Level 2). This approach supports targeted disease control more effectively than a general surveillance system, which, despite being timely conducted, can only prevent a portion of the potential disease spread (as illustrated in Fig 2). The control shown in Fig 2 can be considered a real-time monitoring and proactive control in response to a new outbreak detection. It is performed as soon as the new outbreak is notified to detect its outgoing movements in the recent past. The subnetwork found in a forward direction will help identify positive farms and prevent the spread of infection to other farms in the future.

**BCT method**, on the other hand, aims to trace the primary source of infection from a newly detected outbreak. This retrospective strategy consists of finding temporal valid pathways through incoming movements within specific time constraints and degrees of separation. Typically, BCT uses a broader time window compared to FCT, allowing for a comprehensive analysis of the infection's origins. Both methods rely on (require) updated data. Nowadays, current Italian legislation requires the registration of animal movements in NDB within 7 days, but this does not always happen. More often, and especially for species regulated more recently, registration times are longer. This means that some of the real connections may be missing in NDB when performing FCT or BCT strategies, leading to undetected possible infectious nodes.

**Combination of strategies (FCT-BCT)** involves using both methods to enhance disease control efforts. For instance, after performing a BCT starting from multiple recent outbreaks within a large time window, the resulting subnetwork may include numerous nodes. If among these nodes there are outbreaks notified in the past, an FCT can be conducted on this newly identified BCT subnetwork. This intersection (BCT ∩ FCT) reveals the subnetwork connecting the outbreaks through temporally valid paths. Then,

by working in the past, when data is more likely to be updated in NDB, this strategy identifies nodes that have been exposed to infected animals but have not yet tested positive. A comparison of animal movement dates with the last inspections on the farms can help promptly identify nodes for further inspection, aiding in containing the disease's spread. Ultimately, such kind of strategies could be performed using batches of animals (thus letting the paths highlight the spread accounting for transmission between animals sharing the same farms) or considering the individual animals moving forward or backward from a seed farm. These strategies can be applied either to batches of animals (highlighting the spread through shared farm contacts) or to individual animals moving between farms. The T-Racing application facilitates these analyses, enabling users to leverage epidemiological information and expert knowledge to transform vast amounts of data into clear, actionable guidelines for disease control.

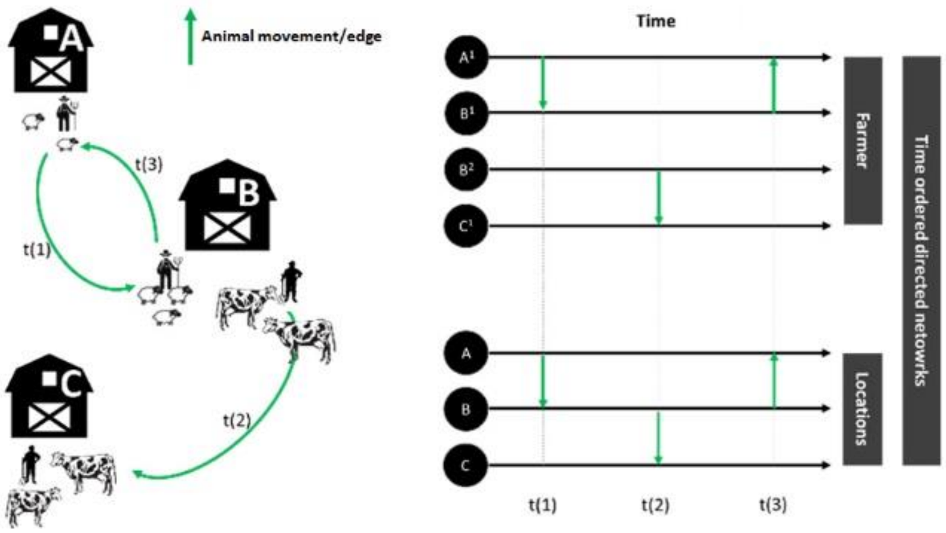


**Fig 1** Network fragmentation and aggregation in animal movement records. The top right panel shows how multiple farmers of the same holding are represented as different nodes, leading to artificial fragmentation. The bottom right panel demonstrates the aggregation process, where each node represents the physical holding, encompassing multiple owners at a fiscal level.

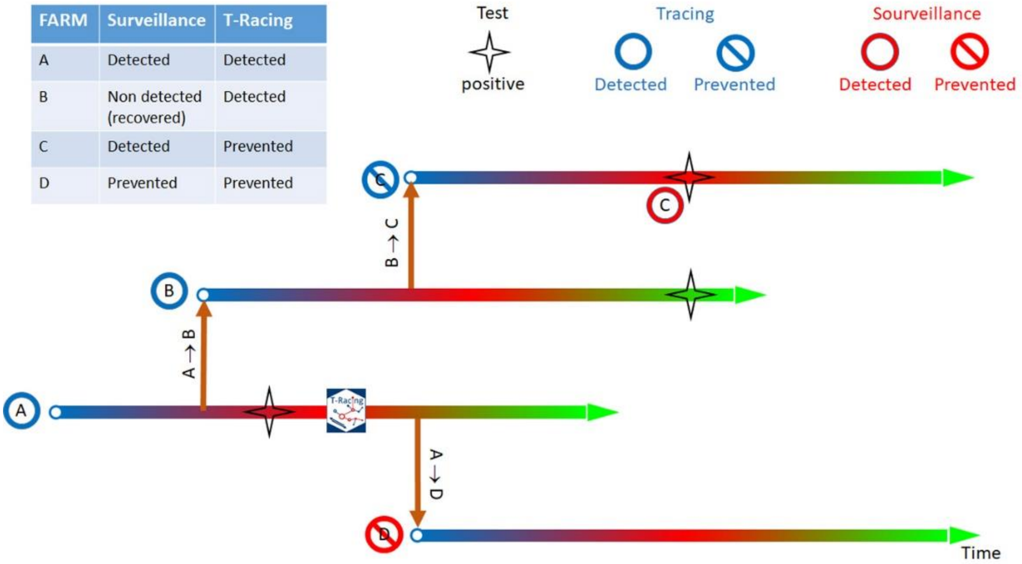


**Fig 2** Schematic illustration of the importance of tracing in preventing and detecting diffusion pathways versus the simple farm isolation when only positivity recorded in the context of the surveillance program is considered.

*2.3 T-Racing Design*

The T-Racing interface allows users to quickly query, filter, analyze, and visualize data through interactive graphs, maps, and tables for a seamless and intuitive experience. Fig 3 provides a conceptual map of the application, highlighting its primary dashboard sections and features.

2.3.1 Tools for extracting and viewing animal trade data

- **Filtering data** based on multi-species selections, time frame, tracing direction mode (backward or forward methods), network geo-aggregation mode, and including/excluding animal trade data from/to foreign countries or slaughterhouses;
- **Multi-seed selection** for tracing, allowing users to either type or select one or more farm codes from the list of farms or select outbreak codes from the list of officially notified outbreaks within a specified time frame. Alternatively, the user can select all farms and/or outbreaks falling within a spatial buffer (neighbours distant by kilometres) around selected farms and outbreaks;
- **Tracking a single animal** by typing its identification code and viewing all resulting spatial-temporal paths within a specified time frame.

The system retrieves movement data based on these filters from the NDB and builds a time-ordered directed weighted network originated by the set seeds. These tools are useful for analyzing diseases that are transmitted through direct contact (i.e. brucellosis or tuberculosis) and diseases that are transmitted through spatial proximity (i.e. African swine fever or vector-borne diseases such as west Nile or Bluetongue, etc.), using spatial buffering and contact tracing methods.

2.3.2 Tools for customizing network visualization and optimization

- **Customizing graph visualization** by setting from a range of layout options such as hierarchical, geographical, or nicely mode (default mode). Users can also select the shape and colour of nodes or the colour of edges to reflect chosen data attributes such as holding type or production type for nodes, and level, time, or species for edges. All possible attribute colours and shapes are predefined in a configuration file;
- **Removing** unnecessary **nodes or edges** from performed tracing;
- **Highlighting within the network all edges involving animals moved from/to the seed, or reducing the network to only those edges involving animals moved from/to the seed**, according to the tracing direction mode set by the user;
- **Reducing the network size** by decreasing network deepness (in days or levels).

For each network reduction method applied, a new time-ordered network will be recalculated to ensure and maintain the temporal coherence in the events.

2.3.3 Tools for epidemiological information integration and results printing

- **Adding a layer** of sequenced pathogens for the animals, and/or of outbreaks for a specific disease **or reducing the resulting network** by keeping only temporally valid paths among involved outbreaks by a combination of FCT and BCT methods;
- **Download the network's tables** (based on selection) in Excel format;
- Storing the results in a **dynamic HTML report** for offline use.

By using these tools, the network graph can be customized and fine-tuned to provide a more meaningful representation of the data.

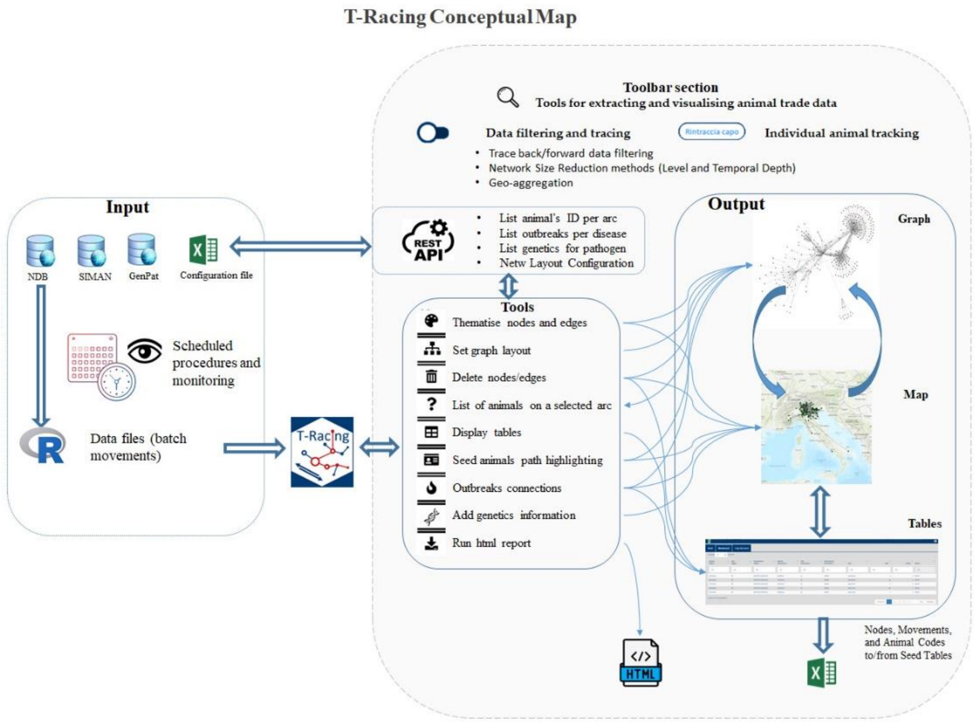


**Fig 3** T-Racing conceptual map.

*2.4 Data flow and system architecture*

T-Racing has been developed using the Shiny library by the R statistical environment, and leaflet and visnetwork libraries were used for map and graph visualization respectively (Table 1 contains a list of all packages used). The application manages large amounts of data from different information systems and shares them efficiently and securely in real-time. Differently from the serverless JavaScript web apps, computationally heavy services are executed on a remote server, and the Plumber package allows the application to query data sources in real-time, through web API.

The system architecture facilitates the application scalability, thanks to shiny proxy (a free and open-source Docker-based application). The described stack and the involved application modules are displayed in Fig 4.

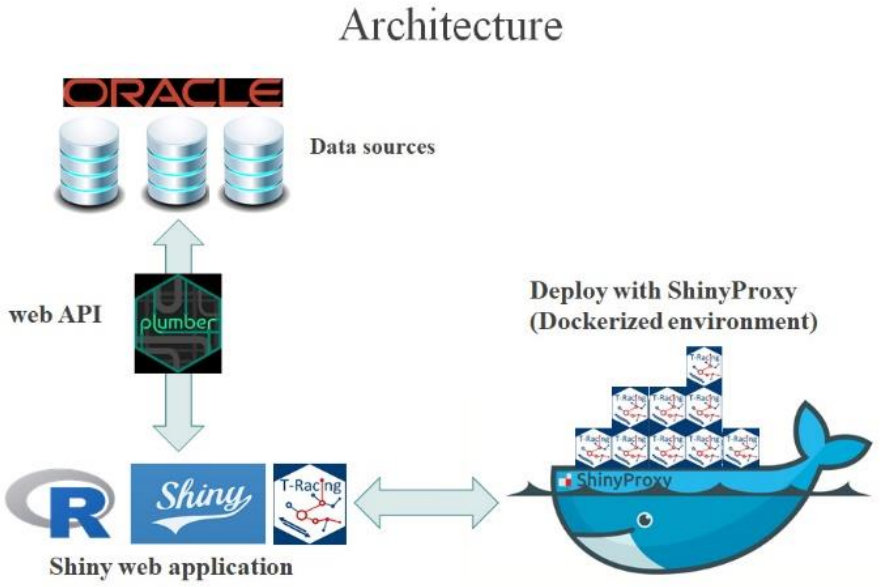


**Fig 4** T-Racing architecture.

**Table 1.** List of the R-packages used in T-Racing development.

| R-package | Use | Reference |
|---|---|---|
| shiny (v. 1.7.1) | UI/SERVER | Winston Chang, Joe Cheng, JJ Allaire, Carson Sievert, Barret Schloerke, Yihui Xie, Jeff Allen, Jonathan McPherson, Alan Dipert and Barbara Borges (2021) |
| shinyjs (v. 2.1.0) | UI/SERVER | Dean Attali (2020). shinyjs: Easily Improve the User Experience of Your Shiny Apps in Seconds |
| shinyWidgets (v. 0.6.4) | UI | Victor Perrier, Fanny Meyer and David Granjon (2021). shinyWidgets: Custom Inputs Widgets for Shiny |
| Shinydashboard (v. 0.7.2) | UI | Winston Chang and Barbara Borges Ribeiro (2018). shinydashboard: Create Dashboards with 'Shiny' |
| shinyBS (v. 0.61.1) | UI | Eric Bailey (2015). shinyBS: Twitter Bootstrap Components for Shiny |
| shinybusy (v. 0.3.0) | UI/SERVER | Fanny Meyer and Victor Perrier (2020). shinybusy: Busy Indicator for 'Shiny' Applications |
| DT (v. 0.22) | UI | Yihui Xie, Joe Cheng and Xianying Tan (2021). DT: A Wrapper of the JavaScript Library 'DataTables' |
| rintrojs (v. 0.3.0) | UI/SERVER | Carl Ganz (2016). rintrojs: A Wrapper for the Intro.js Library. Journal of Open Source Software, 1(6), October 2016 |
| shinycssloaders (v. 1.0.0) | UI/SERVER | Andras Sali and Dean Attali (2020). shinycssloaders: Add Loading Animations to a 'shiny' Output While It's Recalculating |
| htmltools (v. 0.5.2) | UI | Joe Cheng, Carson Sievert, Barret Schloerke, Winston Chang, Yihui Xie and Jeff Allen (2021). htmltools: Tools for HTML |
| leaflet (v. 2.1.1) | UI | Joe Cheng, Bhaskar Karambelkar and Yihui Xie (2022). leaflet: Create Interactive Web Maps with the JavaScript 'Leaflet' Library |
| rgdal (v. 1.5.31) | SERVER | Roger Bivand, Tim Keitt and Barry Rowlingson (2021). rgdal: Bindings for the 'Geospatial' Data Abstraction Library |
| sp (v. 1.4.7) | SERVER | Pebesma, E.J., R.S. Bivand, 2005. Classes and methods for spatial data in R. R News 5 (2) |
| bezier (v. 1.1.2) | SERVER | Aaron Olsen (2018). bezier: Toolkit for Bezier Curves and Splines |
| geosphere (v. 1.5.14) | SERVER | Robert J. Hijmans (2021). geosphere: Spherical Trigonometry |
| visNetwork (v. 2.1.0) | UI | Almende B.V., Benoit Thieurmel and Titouan Robert (2019). visNetwork: Network Visualization using 'vis.js' Library |
| igraph (v. 1.3.1) | SERVER | Csardi G, Nepusz T: The igraph software package for complex network research, InterJournal, Complex Systems 1695. 2006 |
| ggplot2 (v. 3.3.6) | SERVER | H. Wickham. ggplot2: Elegant Graphics for Data Analysis. Springer-Verlag New York, 2016 |

| | | |
|---|---|---|
| RColorBrewer (v. 1.1.3) | SERVER | Erich Neuwirth (2014). RColorBrewer: ColorBrewer Palettes |
| xlsx (v. 0.6.5) | SERVER | Adrian Dragulescu and Cole Arendt (2020). xlsx: Read, Write, Format Excel 2007 and Excel 97/2000/XP/2003 Files |
| dplyr (v. 1.0.9) | SERVER | Hadley Wickham, Romain François, Lionel Henry and Kirill Müller (2021). dplyr: A Grammar of Data Manipulation |
| jsonlite (v. 1.8.0) | SERVER | Jeroen Ooms (2014). The jsonlite Package: A Practical and Consistent Mapping Between JSON Data and R Objects. arXiv:1403.2805 |
| curl (v. 4.3.2) | SERVER | Jeroen Ooms, Hadley Wickham, RStudio |
| rJava (v. 1.0.6) | UI | |

*2.6 Demo*

The T-Racing application is not publicly available as it handles sensitive data from Italian national information systems. Therefore, a demo version has been developed to demonstrate its potential to the scientific community. This demo operates with a pre-configured dataset, which has been adjusted by adding noise and rounding coordinates to the second decimal place in the georeferencing of holdings, which may result in some holdings falling outside the Italian boundaries. The demo has been translated into English, while the original application is in Italian. The original development and publication environment, as well as the web services, have been duplicated and adapted for the demo. The underlying dataset is contained in a single Oracle database. The demo version is intended to allow users to explore and replicate the results presented in our study, providing a comprehensive preview of its features as listed in Table 2.

**Table 2.** List of Data available in T-Racing Demo.

| **Data** | **From** | **To** | **Type/feature** |
|---|---|---|---|
| **Movements** | 01/12/2017 | 30/06/2023 | batch and individual for bovine and buffalo |
| **Outbreaks** | 01/12/2017 | 30/06/2023 | *Brucellosis*, *Tuberculosis* |
| **ST** | 01/01/2015 | 01/12/2018 | *B. abortus* and *B. melitensis* species |

*2.5 Brucellosis and Tuberculosis: Rationale for Case Studies and Italian Regulations*

The following case studies were considered:

**Brucellosis in the Marche region (central Italy)**. On December 9, 2019, a brucellosis outbreak was confirmed in central Italy, specifically in the Marche region. The T-Racing application was utilized to identify the potential source of the infection. Only bovine species were considered, while slaughterhouses and foreign countries were excluded from the analysis. The known outbreak code from a list of proposed brucellosis outbreaks was selected as the seed for a trace backwards analysis encompassing all incoming movements to the identified outbreak over the past two years at 4 depth levels. The resulting network was refined using the "outbreaks connection" tool, and genetic data was added to support the conclusions drawn from the final network.

**Tuberculosis outbreaks affected Elba Island and the nearby Tuscany coast** in June 2023. A forward tracing from March to December 2023 to identify the likely spread trajectory leading up to the outbreak on Elba Island was used. Only bovine species were considered, slaughterhouses were included, and foreign countries were excluded from the analysis. Additionally, we utilized a "Seed animal's path highlighting" tool to emphasize the flow of the infection about the animals coming from the seed.

**A tuberculosis outbreak occurred in the Cuneo province in December 2022**. A trace backward analysis over a 13-month temporal window with a level depth of 4 was used to determine the most likely source of infection. Network reduction was applied using the

dedicated tools for highlighting paths among involved outbreaks ("outbreaks connection" tool).

We used T-Racing to investigate brucellosis and tuberculosis outbreaks in non–endemic areas of Italy, where an occurrence of an outbreak should trigger a rigorous investigation by the official veterinary service to prevent the disease from spreading, demonstrating the speed and effectiveness of the investigation carried out with the proposed web app.Brucellosis caused by *Brucella abortus* is an important zoonoses worldwide, and tuberculosis caused by *Mycobacterium bovis* constitute a serious hazard to public health in developing countries and a great economic burden to livestock production in Europe. These diseases were chosen due to their public health and economic importance and their chronic development, allowing for the use of FCT and BCT methods with a broad time-elapse. Prevention of these zoonoses in humans depends on the control of the disease in animals. Livestock movement data represent a valuable source of information to understand the pattern of contacts between holdings, which may determine the spread of such diseases. The first national plans for the control of bovine tuberculosis and brucellosis were approved with the Ministerial Decree of 11 and 12 March 1965, respectively. Through the Ministerial Decree of 27 August 1994 n. 651 entered into force in Italy the national eradication plan for bovine brucellosis, which establishes the measures to be applied to cattle farms to obtain the eradication of the disease for the protection of public health and protection of officially free herds. The regulation determines for control the use of official serological tests twice a year at an interval of not less than three and not more than six months. All animals over the age of twelve months in all cattle farms have to be tested, even if are located in the wild. Similar procedures have been established (test all animals in all heads at once, with 3 to 6 months intervals) with Ministerial Decree 15 December 1995, n. 592 – Regulation concerning the national plan for the eradication of tuberculosis in cattle and buffalo herds – by testing with the tuberculin intradermal test.

## 3. Results

T-Racing Demo, which uses a limited and anonymized dataset, is available at this link https://demo.izs.it/app_direct/tracing/. The basemaps throughout all the following figures were created using ArcGIS® software by Esri and integrated into the shiny web application using the leaflet.providers R library, sourced from leaflet-providers.js (https://github.com/leaflet-extras/leaflet-providers). ArcGIS® and ArcMap™ are the intellectual property of Esri and are used herein under license. Copyright © Esri. All rights reserved. For more information about Esri® software, please visit www.esri.com.

Fig 5 shows the T-Racing interface, which displays temporally valid paths originating from a brucellosis outbreak in Sicily, an Italian southern island, using the trace forward method. The investigation revealed this outbreak, detected in July 2020, was likely the source of two subsequent outbreaks in Pisa, Tuscany, northern Italy. Animals moved from the seed in March reaching, via a transit station, two farms in Pisa in April and May, but the outbreaks were only detected by surveillance in December 2020. The simultaneous visualization of the queryable and interactive graph, map, and tables, combined with the iterative use of tools for network calibration and targeted result personalization, offers a more comprehensive and user-friendly result viewing.

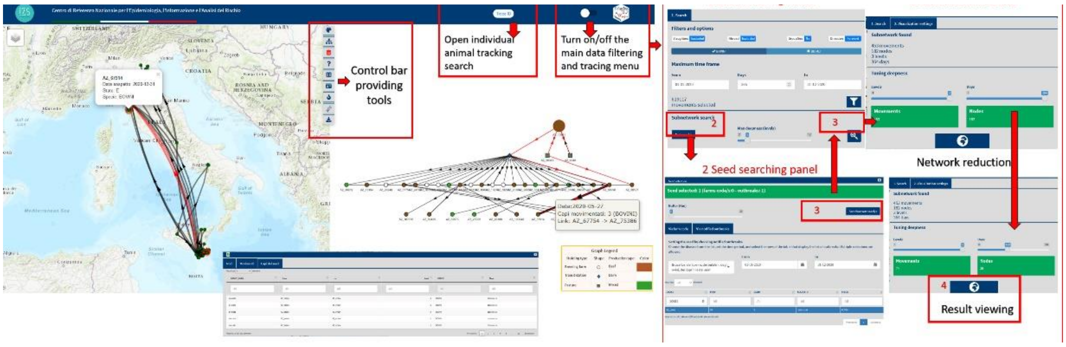


**Fig 5** The T-Racing interface (on the left) shows the subnetwork resulting from the trace forward analysis on a map, graph, and tables and the steps followed to get the results in the interface are shown on the right. This analysis was performed using a brucellosis outbreak in July 2020 in the Sicily Region as the seed, extracting all bovine movements in 2020 up to the third level of depth (three degrees of separation). A time frame-based network reduction/optimization method was applied for the result. The "hierarchical" graph layout shows all nodes and edges in the subnetwork, highlighting probable disease pathways and nodes reached during the study period. Node shape and colour represent farm type and production activity (see legend). The larger, red-bordered icon represents the seed, with each node labelled by farm code. Arrows indicate animal movements between two nodes, with the width proportional to the number of animals moved. Red-coloured arrows highlight paths followed by animals moving from the seed (using the "Seed animals path highlighting" tool). Fire icons on the map represent brucellosis outbreaks detected during the selected period and included in the subnetwork (using the "Outbreaks connections" tool). Tooltips and tables provide detailed information, facilitating navigation between the map, graph, and tables.

### *3.1 T-Racing - Case Studies*

3.1.1. Tracing backward from a notified outbreak of brucellosis in the Marche region of central Italy, in December 2019, to identify its potential sources of infection.

The search for the subnetwork resulted in 1.825 movements involving 386 nodes. The subnetwork covers a substantial area of central Italy, including holdings that reach the seed even from the islands of Sicily and Sardinia. The theming of nodes based on type reveals the involvement of different holding types. In addition to breeding farms, there are pastures (structures that play a significant role in the spread of brucellosis) and a hub node, a genetic collection centre located in Perugia province, Umbria region, in central Italy, that connects 132 nodes (Fig 6). To make the network more manageable and relevant for the conducted tracing, a subnetwork was created by setting 3 levels and reducing the depth to 399 days, resulting in 198 movements and 38 nodes. To identify plausible paths of disease spread, the application allows the utilization of notified outbreaks within a customizable time frame for the specific disease. These outbreaks are visualized on the map, enabling the tracing to be narrowed down to the connections between outbreaks included in the tracing. This helps identify a focused subset of connections to investigate. The subnetwork includes multiple brucellosis outbreaks, primarily located in the southern and central regions. The reduced network, created by keeping only temporally valid paths among involved outbreaks within the network, consists of 94 movements involving 11 nodes. At this point, we can assess the presence of genetic information as further confirmation of the tracing's plausibility. To do so, we utilize the tool by adding the available sequence type for *Brucella abortus*. The seed is an outbreak for which sequence type "2" is available, and the identified origin zones are also affected by clusters of the same sequence type. An interesting aspect is to determine how many of these links involve the same animals that reached the seed. The dedicated tool allows for theming the links based on this characteristic by searching for individually identified animals. The visualization highlights on the subnetwork the paths involving the same animals that reach the seed. These highlights paths consist of only 21 movements involving 5 nodes (including the seed and one pasture, which is the presumed source of infection). Further investigation of individually identified and traced animals revealed that an infected

animal, identified as sequence type "2" in the seed, originated from a pasture, which was subsequently identified as the plausible source of the sought-after infection (Fig 7).

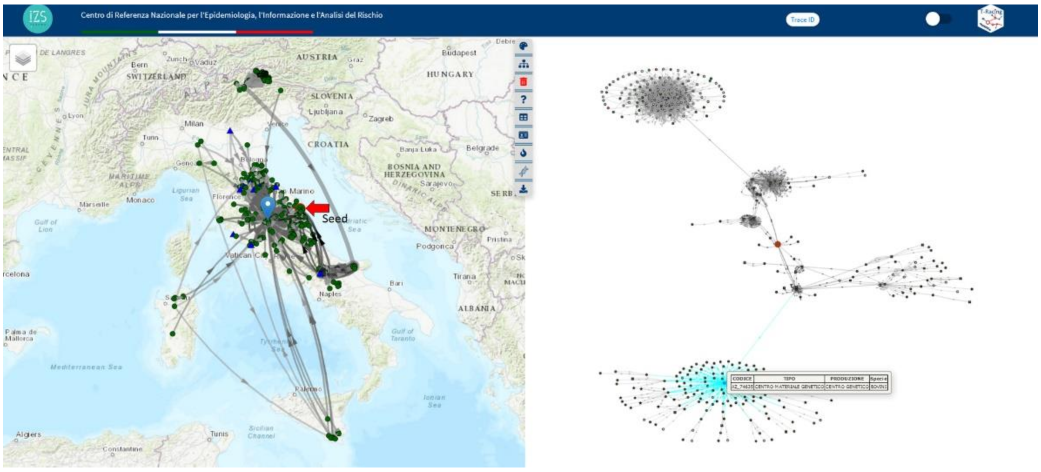


**Fig 6** Trace backward method. Sequential investigation steps: 1) Data Filter, 2) Set seed by outbreak code search, and 3) Network view. The trace-back focusing on bovine species, excluding slaughterhouses and foreign countries, was conducted to trace the outbreak occurring in December in the Marche region, in Italy. 1,825 movements involving 386 nodes were identified within a broad time spanning the past two years. The seed is contoured in red. The network covers a substantial area of central Italy, including farms extending to the islands (Sicily and Sardinia). Node theming based on structure type highlights diverse holding types, including pastures and a genetic collection centre in Perugia, highlighted in light blue on the graph (simultaneously, its marker appears on the map).

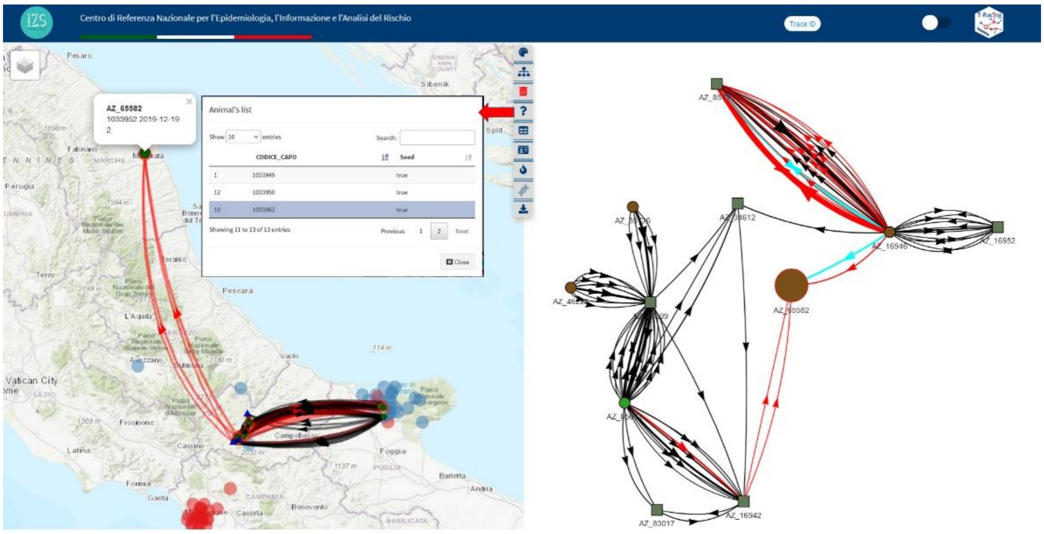


**Fig 7** The subnetwork result after the following four sequential steps: 1) Network reduction by setting the network level to 3 and days depth to 399 (the new subnetwork size is 198 links and 38 nodes), 2) Further reducing the subnetwork by keeping only temporally valid paths among involved outbreaks (resulting in a subnetwork size of 94 links and 11 nodes; the four detected brucellosis' outbreaks involved are shown on the map with red fire icons), 3) Adding the sequence type for Brucella abortus layer to the map (type 2 in blue circles) and displaying a popup on the seed showing the identification code of the animal, sampled date, and detected Brucella abortus sequence type, and 4) Highlighting on the graph are all links involving the same animals that reached the seed (red links). Further investigation on "individually identified animals," found by querying the highlighted links (in light blue on the graph), revealed that an infected animal originating from the pasture reached the seed. This animal is listed in the Animal list produced by the application, matching the animal reported in the map popup.

3.1.2. Tracing the flow of tuberculosis outbreaks: Elba Island and Cuneo Province.

**-Four BTB outbreaks were notified in June 2023 in a small, non-endemic area, in Italy, affecting Elba Island and the nearby Tuscany coast.** Using T-Racing allowed for the detection of the real flow of the disease spread. The resulting network highlighted an animal movement of nine cattle in April 2023, which reached farms on the nearby coast. This established the real source of the infection and the most likely flow of the spread (Fig 8). We discovered that the animals reaching the outbreaks were the same ones that had left Elba Island and one of them (at the time of writing this article) had not yet been slaughtered.

-**In December 2022, an outbreak of BTB occurred in the Cuneo province of the Piedmont region in northern Italy.** The resulting network comprised 306 farms and 664 connections, predominantly located in the Sicily region. For a more focused investigation, we considered only temporally valid paths connecting BTB outbreaks included in the network resulting in a subnetwork of 50 connections involving 9 breeding farms in the Sicily region, four of which were BTB outbreaks, and two pastures. The investigation suggested that four outbreaks occurring in the previous months are the most likely source of infection. Additionally, the 119 animals reaching the Cuneo province in June and July originated from a fattening farm where they were likely infected by mixing with animals coming from the four outbreaks. The results indicate the need for targeted control measures for the other five farms involved in the revealed diffusion paths (Fig 9).

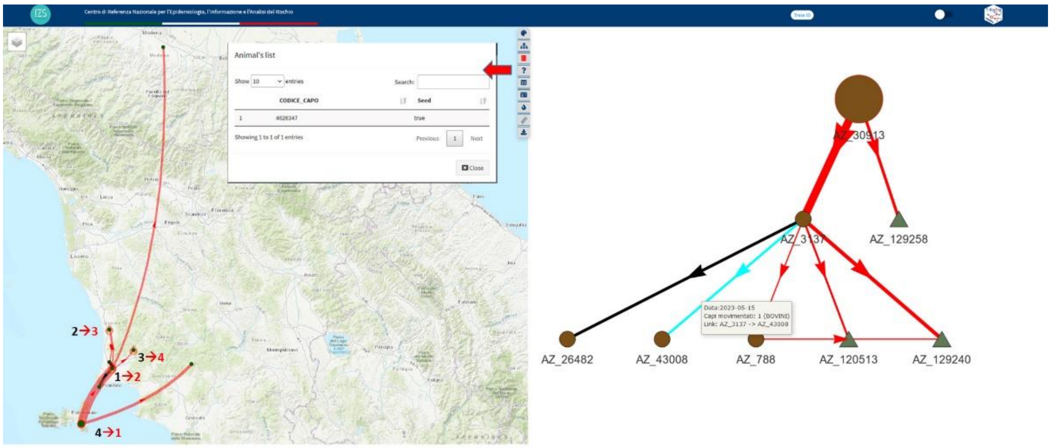


**Fig 8** Trace forward method. The map and graph show outbreaks that occurred on Elba island and the nearby Tuscany coast (black numbers '1' to '4', represent the notification timing, with 1 the earliest and '4' the most recent notified one). The network of animal movements involving the outbreaks is displayed with red links indicating the presence of at least one animal originating from the seed, and black link representing the movement of animals not originating from the seed. The notified outbreaks' timing contrasts with the real flow of disease spread revealed by T-Racing analysis (red numbers '1-4'). The triangles on the graph are slaughterhouses. Further investigation on "individually identified animals," found by querying the highlighted link (in light blue on the graph), revealed that one animal originating from the seed had not yet been slaughtered. This animal is listed in the Animal’s list produced by the application.

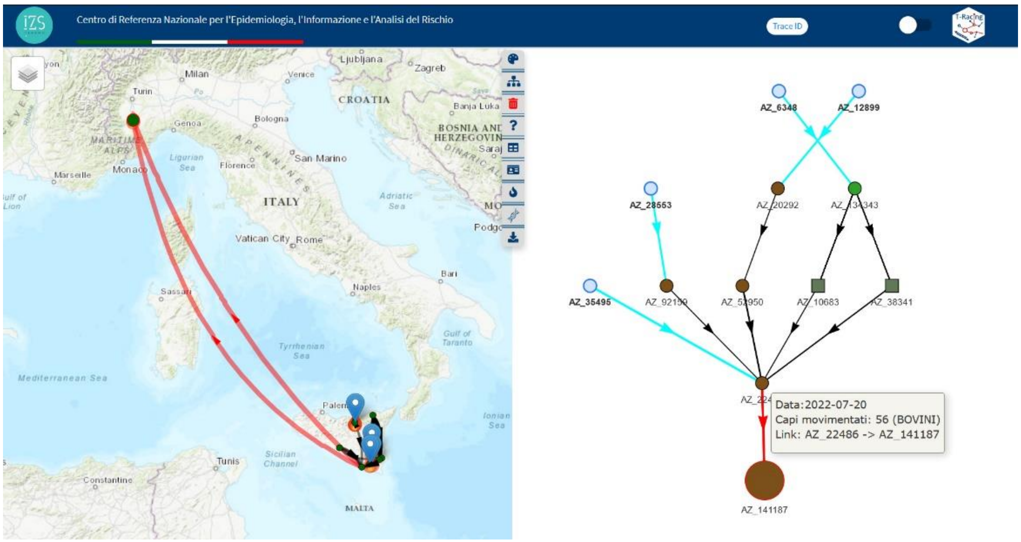


**Fig 9** Trace backward method. The network resulting from the BTB outbreak occurred in Cuneo province, in the Tuscany region in December 2022 involving 306 farms and 664 links, mostly located in the Sicily region. The map and graph show the reduced network by considering only temporally valid paths connecting involved outbreaks consisting of 50 connections and 12 holdings which 9 breeding farms in the Sicily region (circles on the graph), four of which are BTB outbreaks (fire icons and marked on the map and highlighted in light blue on the graph), and two pastures (squares on the graph). The links involving animals reaching the Cuneo outbreak are in red colour.

## 4. Discussion

The T-Racing application was created by a team of experts including epidemiologists, veterinarians, mathematicians, and computer scientists. This collaborative effort spanned all stages of development, from gathering data to creating tools and building a user-friendly interface. The team has extensive experience in epidemiological tracing and dynamic network analysis, as evidenced by similar web applications they have developed since 2011 [24, 33, 34, 35, 36]. T-Racing is an improved version of previous applications, offering faster speeds, additional features, and integration of various epidemiological datasets. Currently, it is available on the internal network of IZS-Teramo and used by our veterinarians and epidemiologists. It is pending technical adjustments and official approval from the Italian Ministry of Health to be integrated into the VETINFO portal. This integration will ensure secure access and the protection of sensitive data, as T-Racing inherits the robust GDPR techniques already applied in the portal.

Timely identification of outbreaks and systematic contact tracing can be effective in stopping the transmission chain of contagious diseases, especially with strong collaborations between epidemiological teams, laboratory surveillance, and decision-making officials, improving outbreak management, in a multidisciplinary context [37].

An important feature of the modern response to epidemics is the increasing focused on exploiting all available data to inform the response in near real-time and to allow for the data evidence-based decision-making [38]. The capability of T-Racing to handle zoonotic diseases such as brucellosis, tuberculosis, and others under veterinary surveillance in Italy highlights its crucial role in the One Health approach. By effectively managing and analyzing data related to these diseases, T-Racing not only aids in preventing and controlling the spread of infections across species but also supports broader public health initiatives. This functionality is particularly important given the complex nature of zoonotic diseases, which can have significant impacts on both animal and human populations. T-Racing has the potential to significantly enhance animal disease control measures, as it can rapidly organize crucial contact information from extensive datasets and identify connections in a multi-outbreak investigation. It introduces new methods and tools currently not available in other systems or tools [2, 24,

33, 34, 35, 36]. These include multi-seed/species selection, seed selection using a spatial buffer around cases or outbreaks, and the combination of strategies (FCT-BCT) configuring itself as an innovative application in its field. Thus, the application efficiently supports epidemiological investigations, as it was shown in the demo examples for brucellosis and tuberculosis. In these examples, it is possible to see how deep into the transmission chain the T-Racing allows us to explore. The initial tracing example has identified a likely path of disease spread and the presumed origin, starting from a relatively large and diverse network (1.825x386) in both spatial and temporal dimensions. By using interactive and iterative tools, along with additional information such as disease outbreaks, genetic data, and movements of individually identified animals, it was possible to narrow down the focus to 21 movements and 5 nodes in just a few steps, saving a significant amount of time. Trying to achieve the same result by simply extracting the movement network through a query of the NDB would have been extremely difficult, like finding a needle in a haystack. The second example clarified the actual timing of the tuberculosis spread. Importantly, the animals from the seed had not been slaughtered at the time of writing this article, and in the last example, results indicate a need for targeted control measures for the other five farms involved in the revealed diffusion paths.

Considering chronic diseases, such as brucellosis and tuberculosis, clarifying the disease's spread patterns allows for focus measures of control, improving targeting financial and human resources, as well as time sensitive decisions. Noteworthy, the almost real-time data used, the possibility of analyzing many datasets at once and the user-friendly environment are also features that could greatly advance responses to emergencies with acute and highly contagious diseases when movement data could be generated in real-time.

The integration of molecular epidemiological data into the analysis complements the validation of identified disease (brucellosis) transmission paths, exemplifying a One Health approach developed by a multidisciplinary team to advance disease control technology. In an era of rapid technological advancements, fostering innovation in epidemiological investigations is essential, as it can strengthen trust between the veterinary and human health systems in addressing zoonosis [38]. T-Racing empowers ~~clinicians, veterinarians, ecosystem health professionals,~~ researchers, and policymakers to leverage animal surveillance data for more comprehensive, integrated disease control strategies [3, 39], enabling informed decisions and proactive measures to mitigate the impact of zoonotic diseases. This tool's data-driven nature underscores the importance of accurate data management, as reliable data is crucial for effective and trustworthy epidemiological investigations.

Although T-Racing is currently operational, it does have some limitations that are important to consider. The application's specific structure, tailored to the Italian context, limits its broader usability. At present, the genetic data is restricted to the Brucella genus, but additional pathogen data will be incorporated as soon as it becomes available in GenPat. Data regarding testing from surveillance systems on farms will be included, and functions will be created to factor in the timing of testing when selecting the most probable disease spread paths. Also, detailed information about single animals, like demographics and parturition dates for calves, along with human cases will be considered. There is also an evaluation underway for the development of scheduled algorithms that will operate directly on the database to identify critical networks to be automatically provided to T-Racing's users. This will enable more efficient, rapid, and straightforward validation and initiation of investigations. However, the authors and team of experts will continue to develop future versions to improve the application by adding new datasets and functionalities. Concurrently, work is ongoing for a more general application that allows users to upload their own data, and its source code will be freely distributed on GitHub.

## 5. Conclusions

The T-Racing web app represents a modern and forward-thinking approach to support epidemiological investigations in animal disease outbreaks in Italy. It provides a unified environment to manage and analyze the expanding pool of data sources iteratively by integrating advanced technology, comprehensive data management, and cutting-edge analytics. T-Racing has the capability to draw meaningful conclusions based on a data-driven, user-guided approach while leveraging its expertise. It facilitates evidence-based decision-making and serves as a leading tool in disease control, enhancing our capacity to address emerging challenges and safeguard global health from a One Health perspective.

**Author Contributions:** Conceptualization, L.C., L.S., A.C., P.C. and F.D.M.; methodology, L.C. and L.S.; software, L.C. and S.P.; validation, F.D.M. and P.C.; data curation, D.P.; writing—original draft preparation, L.S., L.C., A.C.T.R.B.C. and F.D.M.; writing—review and editing, L.C., L.S., A.C., F.D.M., P.C., S.P., A.D.L., D.P., S.T., S.S., and M.T.; project administration, P.C.; funding acquisition, P.C.. All authors have read and agreed to the published version of the manuscript.

**Funding:** This research was funded by Italian Ministry of Health (RC MSRCTE0219).

**Conflicts of Interest:** The authors declare no conflict of interest.

## References


1 Schirdewahn, F., K. Lentz, H. H., Colizza, V., Koher, A., Hövel, P., & Vidondo, B. Early warning of infectious disease outbreaks on cattle-transport networks. PLOS ONE, 16(1), e0244999. https://doi.org/10.1371/journal.pone.0244999

2 Maria Nöremark and Stefan Widgren. EpiContactTrace: an R-package for contact tracing during livestock disease outbreaks and for risk-based surveillance. BMC Veterinary Research 2014, 10:71 Page 2 of 9 http://www.biomedcentral.com/1746-6148/10/71

3 Prata, J. C., Ribeiro, A. I., & Rocha-Santos, T. (2022). An introduction to the concept of One Health. One Health, 1-31. https://doi.org/10.1016/B978-0-12-822794-7.00004-6

4 Wondwossen A. Gebreyes, Daral Jackwood, Celso Jose Bruno de Oliveira, Chang-Won Lee, Armando E. Hoet, Siddhartha Thakur. Molecular Epidemiology of Infectious Zoonotic and Livestock Diseases. 2020. Microbiology Spectrum, 10.1128/microbiolspec.ame-0011-2019, 8, 2, https://journals.asm.org/doi/abs/10.1128/microbiolspec.ame-0011-2019

5 Neo, J. P. S., & Tan, B. H. (2017). The use of animals as a surveillance tool for monitoring environmental health hazards, human health hazards and bioterrorism. Veterinary Microbiology, 203, 40-48. https://doi.org/10.1016/j.vetmic.2017.02.007

6 D Kriebel, J Tickner, P Epstein, J Lemons, R Levins, E L Loechler, M Quinn, R Rudel, T Schettler, A M Stoto. The precautionary principle in environmental science. 2001, Environmental Health Perspectives, 871-876, 109, 9, https://ehp.niehs.nih.gov/doi/abs/10.1289/ehp.01109871

7 Rocha VC Figueiredo, Souza-Filho AF, Ikuta CY, et al. High discrimination of Mycobacterium bovis isolates in Brazilian herds by spoligotyping. **Preventive Veterinary Medicine**, v.179, p.104976, 2020. Doi: 10.1016/j.prevetmed.2020.104976.

8 Taye H, Alemu K, Mihret A, Wood JLN, Shkedy Z, Berg S, Aseffa A. Global prevalence of Mycobacterium bovis infections among human tuberculosis cases: Systematic review and meta-analysis. Zoonoses Public Health. 2021 Nov;68(7):704-718. doi: 10.1111/zph.12868. Epub 2021 Jun 24. PMID: 34169644; PMCID: PMC8487997.

9 Laine CG, Johnson VE, Scott H, Arenas-Gamboa AM. Global Estimate of Human Brucellosis Incidence. Emerg Infect Dis. 2023;29(9):1789-1797. https://doi.org/10.3201/eid2909.230052

10 Ansari, S., Heitzig, J., Brzoska, L., Lentz, H. H., Mihatsch, J., Fritzemeier, J., & Moosavi, M. R. (2021). A Temporal Network Model for Livestock Trade Systems. Frontiers in Veterinary Science, 8. https://doi.org/10.3389/fvets.2021.766547

11 Loria GR, Migliore S, Bongiorno C, Ciaccio G and Laddomada A (2022) New European rules introduced by regulation (EU) 2016/429 to facilitate animal trade: With great risk comes great responsibility. Front. Vet. Sci. 9:1003732. https://doi.org/10.3389/fvets.2022.1003732

12 Porphyre, T., Boden, L. A., Correia-Gomes, C., Auty, H. K., Gunn, G. J., & J. Woolhouse, M. E. (2016). Using national movement databases to help inform responses to swine disease outbreaks in Scotland: The impact of uncertainty around incursion time. Scientific Reports, 6. https://doi.org/10.1038/srep20258

13 Regulation (EU) 2016/429 of the European Parliament and of the Council of 9 March 2016 on transmissible animal diseases and amending and repealing certain acts in the area of animal health (‘Animal Health Law') (Text with EEA relevance). Off J Eur Communities. (2016) L84:1–208

14 ORDINANZA 9 agosto 2012 Misure straordinarie di polizia veterinaria in materia di tubercolosi, brucellosi bovina e bufalina, brucellosi ovi-caprina, leucosi, nelle regioni Calabria, Campania, Puglia e Sicilia. (12A09729) (GU Serie Generale n.212 del 11-09-2012) https://www.gazzettaufficiale.it/eli/id/2012/09/11/12A09729/sg (visitato il 25/08/2023)

15 ORDINANZA 28 maggio 2015, Misure straordinarie di polizia veterinaria in materia di tubercolosi, brucellosi bovina e bufalina, brucellosi ovi-caprina, leucosi bovina enzootica. (15A04879) (GU n.144 del 24-6-2015) and following amendments and additions

16 2003/24/EC: Commission Decision of 30 December 2002 concerning the development of an integrated computerised veterinary system (notified under document number C(2002) 5496)

17 EU, 2019. Commission Implementing Regulation (EU) 2019/1715 of 30 September 2019 laying down rules for the functioning of the information management system for official controls and its system components (the IMSOC Regulation) OJ L 261, 14.10.2019

18 Mardones, F., zu Donha, H., Thunes, C., Velez, V., & Carpenter, T. (2013). The value of animal movement tracing: A case study simulating the spread and control of foot-and-mouth disease in California. Preventive Veterinary Medicine, 110(2), 133-138. https://doi.org/10.1016/j.prevetmed.2012.11.024

19 Judite Monteiro Braga et al. Investigation and contact tracing of the first cases of COVID-19 in Mozambique, 2020. Pan African Medical Journal. 2022;41:302. https://doi.org/10.11604/pamj.2022.41.302.26979

20 Kölzsch, A., Davidson, S.C., Gauggel, D. et al. MoveApps: a serverless no-code analysis platform for animal tracking data. Mov Ecol 10, 30 (2022). https://doi.org/10.1186/s40462-022-00327-4

21 Bingham, P., Wada, M., van Andel, M., McFadden, A., Sanson, R., & Stevenson, M. (2020). Real-Time Standard Analysis of Disease Investigation (SADI)—A Toolbox Approach to Inform Disease Outbreak Response. Frontiers in Veterinary Science, 7, 563140. https://doi.org/10.3389/fvets.2020.563140

22 Hang, C.-N.; Tsai, Y.-Z.; Yu, P.-D.; Chen, J.; Tan, C.-W. Privacy-Enhancing Digital Contact Tracing with Machine Learning for Pandemic Response: A Comprehensive Review. Big Data Cogn. Comput. 2023, 7, 108. https://doi.org/10.3390/bdcc7020108

23 Anglemyer A, Moore TH, Parker L, Chambers T, Grady A, Chiu K, Parry M, Wilczynska M, Flemyng E, Bero L. Digital contact tracing technologies in epidemics: a rapid review. Cochrane Database Syst Rev. 2020 Aug 18;8(8):CD013699. doi: 10.1002/14651858.CD013699. PMID: 33502000; PMCID: PMC8241885.

24 Savini, L., Candeloro, L., Perticara, S., & Conte, A. (2019). EpiExploreR: A Shiny Web Application for the Analysis of Animal Disease Data. Microorganisms, 7.SIMAN. Available online: https://www.vetinfo.sanita.it/j6_siman/.

25 Sistema Informativo Veterinario. Available online: https://www.vetinfo.sanita.it/sso_portale/accesso.pl .

26 Decreto 30 maggio 2017. Istituzione di un Centro di referenza nazionale nel settore veterinario. G.U. n 196 del 26 Agosto, 2017

27 Di Lorenzo A, Mangone I, Colangeli P, Cioci D, Curini V, Vincifori G, Mercante MT, Di Pasquale A, Iannetti S. One health system supporting surveillance during COVID-19 epidemic in Abruzzo region, southern Italy. One Health. 2023 Jun;16:100471. https://doi.org/10.1016/j.onehlt.2022.100471. Epub 2022 Dec 7. PMID: 36507072; PMCID: PMC9726647.

28 Diestel, R. 2010. Graph theory. Graduate Texts in Mathematics, Vol. 173, Fourth Edition, Springer-Verlag,866 Heidelberg.

29 Blonder, B., Wey, T.W., Dornhaus, A., James, R. and Sih, A. (2012), Temporal dynamics and network analysis. Methods in Ecology and Evolution, 3: 958-972. https://doi.org/10.1111/j.2041-210X.2012.00236.x

30 Chan J, Holmes A, Rabadan R. Network analysis of global influenza spread. PLoS Comput Biol. 2010 Nov 18;6(11):e1001005. doi: 10.1371/journal.pcbi.1001005. PMID: 21124942; PMCID: PMC2987833

31 Chris Rorres, Maria Romano, Jennifer A. Miller, Jana M. Mossey, Tony H. Grubesic, David E. Zellner, Gary Smith, Contact tracing for the control of infectious disease epidemics: Chronic Wasting Disease in deer farms, Epidemics, Volume 23, 2018, Pages 71-75, ISSN 1755-4365. https://doi.org/10.1016/j.epidem.2017.12.006.

32 Natale F, Savini L, Giovannini A, Calistri P, Candeloro L, Fiore G. Evaluation of risk and vulnerability using a Disease Flow Centrality measure in dynamic cattle trade networks. Prev Vet Med. 2011 Feb 1;98(2-3):111-8. doi: 10.1016/j.prevetmed.2010.11.013. Epub 2010 Dec 15. PMID: 21159393.

33 Calistri P, Conte A, Natale F, Possenti L, Savini L, Danzetta ML, Iannetti S, Giovannini A. Systems for prevention and control of epidemic emergencies. Vet Ital. 2013 Jul-Sep;49(3):255-61. doi: 10.12834/VetIt.1206.06. http://www.izs.it/vet_italiana/pdf/VetIt_1206_06.pdf

34 Iannetti S, Savini L, Palma D, Calistri P, Natale F, Di Lorenzo A, Cerella A, Giovannini A. An integrated web system to support veterinary activities in Italy for the management of information in epidemic emergencies. Prev Vet Med. 2014 Mar 1;113(4):407-16. doi: 10.1016/j.prevetmed.2014.01.015. Epub 2014 Jan 22. PMID: 24485707.

35 Di Lorenzo A., Savini L., Candeloro L., Tora S., Cerella A., Di Sabatino D., Conte A. "Web-GIS and livestock trace tools for epidemiological surveillance, control and management" Front. Vet. Sci. doi: 10.3389/conf.fvets.2019.05.0004

36 Tegegne, H. A., Bogaardt, C., Collineau, L., Cazeau, G., Lailler, R., Reinhardt, J., Freeth, F. T., Taylor, E., Prada, J. M., & Hénaux, V. (2023). OH-EpiCap: A semi-quantitative tool for the evaluation of One Health epidemiological surveillance capacities and capabilities. Frontiers in Public Health, 11, 1053986. https://doi.org/10.3389/fpubh.2023.1053986

37 Jonathan A. Polonsky, Amrish Baidjoe, Zhian N. Kamvar, Anne Cori, Kara Durski, W. John Edmunds, Rosalind M. Eggo, Sebastian Funk, Laurent Kaiser, Patrick Keating, Olivier le Polain de Waroux, Michael Marks, Paula Moraga, Oliver Morgan, Pierre Nouvellet, Ruwan Ratnayake, Chrissy H. Roberts, Jimmy Whitworth and Thibaut Jombart. Outbreak analytics: a developing data science for informing the response to emerging pathogens. Philosophical Transactions of the Royal Society B: Biological Sciences, Volume 374, Issue 1776. https://doi.org/10.1098/rstb.2018.0276

38 Moriyón, I., Blasco, J. M., Letesson, J. J., De Massis, F., & Moreno, E. Brucellosis and One Health: Inherited and Future Challenges. Microorganisms, 11(8), 2070. https://doi.org/10.3390/microorganisms11082070

39 Charlier, J., Barkema, H. W., Becher, P., De Benedictis, P., Hansson, I., Hennig-Pauka, I., La Ragione, R., Larsen, L. E., Madoroba, E., Maes, D., Marín, C. M., Mutinelli, F., Nisbet, A. J., Podgórska, K., Vercruysse, J., Vitale, F., Williams, D. J. L., & Zadoks, R. N. (2022). Disease control tools to secure animal and public health in a densely populated world. The Lancet Planetary Health, 6(10), e812-e824. https://doi.org/10.1016/S2542-5196(22)00147-4

Revised Manuscript with Track Changes

*Article*

# T-Racing: a modern tool for supporting epidemiological investigation in animal disease outbreaks in Italy

Commented [LS1]: Rev3: in Italy
Answer: Done

**Luca Candeloro [1], Lara Savini [1], Annamaria Conte [1], Paolo Calistri [1], Diana Palma [1], Samuel Perticara [1], Anna Cecília Trolesi Reis Borges Costa [2*], Alessio di Lorenzo [1], Susanna Tora [1], Sara Serrani [1], Michela Toro [1], Karina Diniz Baumgarten [2,3] and Fabrizio De Massis [1]**

[1] Istituto Zooprofilattico Sperimentale dell'Abruzzo e del Molise "G.Caporale", Italy
[2] Departamento de Medicina Veterinária, Escola de Zootecnia e Medicina Veterinária, Universidade Federal de Lavras, Lavras Minas Gerais, Brazil.
[3] State Department of Animal Health. Companhia Integrada de Desenvolvimento Agrícola de Santa Catarina – CIDASC, Florianópolis, SC, Brazil

* Correspondence: anninha_veterinaria@hotmail.com

**Abstract:** The One Health approach unites efforts across human-animal-environment interfaces against shared threats like zoonotic diseases. T-Racing is a Shiny web application, that supports epidemiological investigations and helps contain livestock-related disease spread, aligning with multidisciplinary ~~One Health~~ principles ~~of~~ to safeguard~~ing~~ public health. The application uses Temporal Network Analysis techniques to address the dynamic nature of animal trade, facilitating backward and forward tracing strategies. ~~Therefore,~~ T-Racing leverages web services to retrieve data from multiple sources simultaneously and ~~almost~~ in near real-time through the plumber package and is distributed using Shinyproxy. Indeed, T-Racing manages and analyze extensive and diverse datasets within the same environment, including animal movement data, disease outbreak data, and genomic data, all obtained from Italian National databases. In this work, we show T-Racing's capabilities by simulating epidemiological investigations of brucellosis and tuberculosis outbreaks that occurred in non–endemic areas of Italy. This tool supports epidemiological investigations by adopting a data-driven approach, guiding users through the analysis via an iterative process while leveraging their expertise. Therefore, it enables faster data analysis, improves understanding of disease transmission patterns, and facilitates prompt and targeted interventions.

**Keywords:** One Health; Multidisciplinary approach; Epidemiology; Tracing; Temporal Network Analysis; Shiny web application; Outbreak investigation; Zoonoses management.

## 1. Introduction

Commented [LS2]: Rev3: Please divide the introduction into paragraphs based on the idea presented in each enormous paragraph. It will facilitate understanding and flow.
Answer: Thank you for your valuable suggestion. We have followed your advice and divided the introduction into separate paragraphs, each reflecting the key ideas presented. This restructuring aims to improve the clarity and flow of the introduction, making it easier to follow the concepts discussed.

Preventing and controlling communicable diseases in livestock~~,~~ through early detection of outbreaks~~,~~ increases the effectiveness of the ~~emergency~~ response and reduces the social, economic, and environmental costs associated with these events [1, 2]. In Italy, veterinary surveillance programs target several zoonotic diseases in livestock, including brucellosis, tuberculosis, leptospirosis, rabies, salmonellosis, campylobacteriosis, listeriosis, and echinococcosis etc. Animals serve as an excellent proxy for monitoring both novel and known zoonotic pathogens given that more than 60% of emerging infectious diseases in humans originate from animal sources [3-5]. This underscores the critical role of animal health in ~~the~~ implement~~ation~~ing of the One Health approach, by detecting, monitoring, and predicting health hazards that could also ~~affect~~ impact environmental

and human health since some pathogens can survive long periods in the environment [6]. Among the diseases that could affect human health are tuberculosis and brucellosis, both chronic infectious diseases caused by bacteria. Bovine tuberculosis, caused by *Mycobacterium bovis* [7], is a zoonotic disease that was estimated to have a human prevalence varying from 0.4% to 76.7% worldwide [8]. ~~On the other hand, a~~Animal brucellosis caused by *Brucella* spp., is a zoonosis of utmost significance, since it was projected to globally affect 2.1 million people by year [9]. The control of both diseases is anchored on the surveillance and control in animals, with the traceability of animal cases being of paramount importance to prevent ~~occurrences in~~ human infections, especially for human brucellosis that does not have vaccination.

In this context, ~~the movement of~~ animal movement is often considered a major risk for disease spreading between herds [10]~~,~~ and to humans when zoonoses are involved. Hence, livestock transportation data is registered in national and international databases to facilitate contact tracing in the event of an outbreak [11-15]. An example of an international official movements database is TRACES-NT (TRAde Control and Expert System New Technology). TRACES-NT is an information platform of the European Commission for the reporting, certification, and control of imports of animals, origin of animal products, food and feed of plants, and other products of veterinary interest. t is only accessible to competent authorities and is considered sensitive data. The system is only accessible to competent authorities and is considered sensitive data. ~~The system~~It also registers trade and exports of animals and certain products of animal origin into the EU and intra-EU. Currently, TRACES-NT is the integrated computerized veterinary system with a single architecture provided in Decisions 2003/24/EC and 2004/292/EC (EU, 2019) [16, 17]. ~~Consequently, t~~The ~~usage~~ use of animal movement ~~systems~~ data facilitates tracing the spread of diseases within animal populations and the potential routes of exposure being critical for effective disease control and crucial in the final steps of eradication programs [18]. Contact tracing plays a pivotal role in identifying the necessary actions to break the transmission chain, to reduce the spread and duration of infectious diseases, thus preventing new cases [19]. When zoonoses are considered, this also means protecting public health, once contact tracing has the subsequent effect of providing assessments to stakeholders which is useful for quickly identifying and implementing containment, surveillance, and control systems.

~~In this sense, t~~Technology plays a ~~is~~ fundamental role in~~to~~ developing ~~building~~ online tracing applications, that can improve the results of epidemiological investigations. Advanced analytics and link analysis can be used to uncover hidden patterns in the spread of diseases [2~~0~~, 20]. Therewith, tracing applications have been developed to assist animal health officials in quickly identifying the animals involved in an outbreak, where and when the infected animals are located, and whether other susceptible animals might have been exposed to the disease. These actions would be taken by uncovering the missing or unexpected links from the contact details, identifying the animals that should undergo testing, tracking the geographical spread of the disease, and determining which farms are at the highest risk. Thus, tracing applications can help in disease control and surveillance during critical outbreak stages, saving time, preventing further spreading, minimizing costs and reducing the consequences of the disease outbreaks [2~~1~~, 21]. While ~~T~~tracing applications enables veterinary services, epidemiologists, and competent authorities to respond quickly ~~in~~ by identifying the "hot spots" and ~~by~~ implementing effective targeted control and eradication measures. Although it may sound simple, the tracing application can become overwhelmingly complex and resource-intensive, however, using data visualization and analytics can significantly improve the performance and effectiveness,

**Commented [LS3]:** Rev3: switch the order fo the ref, first 2, then 20
Answer: Done

**Commented [LS4]:** Rev3: Since you present the tracing applications below, I would somehow simplify this section or link it with what you present below on epiContact Trace and it's back-and-forth information up and down. It's a pretty dense introduction. I would divide it into paragraphs based on the idea presented in each section.
- the zoonotic diseases and the importance of tracing
- the existing tracing apps and their limitations
- your tracing app - improving the state of the art and aim of the paper
Answer: See the previous comment

**Commented [LS5]:** Rev2: can you provide some tracing applications developed and refernces thereof?
Answer: Thank you for your feedback. We acknowledge the need for a broader overview of existing tracing applications. However, after an extensive review of the literature, we found that detailed references and documentation for additional specific applications in veterinary epidemiology are limited. We have included the most relevant tools, EpiContactTrace and EpiExploreR, which are well-documented but have limitations related to complexity and usability.
If there are specific tracing applications or references that you suggest including, we would be grateful for your recommendations.
Thank you for helping us improve our manuscript.

making the application an invaluable tool. Currently, due to the COVID-19 global pandemic, contact tracing strategies have been widely used and many contact-tracing web apps have been developed for this purpose in human health [22, 23]. In veterinary epidemiology, there is limited literature on specific contact tracing web applications, with notable examples being EpiContactTrace [2] and EpiExploreR [24]. such as EpiContactTrace, [2], which is an open-source tool implemented in R package, allows end users to conduct forward and backward contact tracing by analyzing livestock movement data. However, it requires technical knowledge in R programming, manipulation and adjustment of data, which limits its accessibility for many users having different expertise. Additionally, it lacks of integration of heterogeneous and epidemiologically relevant data necessary for a comprehensive epidemiological investigation. and EpiExploreR, an R Shiny shiny web application, that integrates offers tools for disease mapping, and spatiotemporal analysis tools [24], and integration of diverse datasets, but its complexity need for specialized expertise make it less accessible for field operators and veterinarians who require immediate and user-friendly contact tracing tools. Moreover, both tools lack effective management of large networks, which is crucial for avoiding chaotic and unusable results.

Even though there are tracing applications already developed in the veterinary field, there is still a need for a versatile and user-friendly web tool that can manage various and heterogeneous datasets and incorporate analytical features to quickly perform forward and backward tracing within a spatial-temporal environment. Therefore, the aim of this paper was to introduce T-Racing, a web-based operational tool that has been created developed to assist veterinary epidemiological investigations in Italy. Unlike existing tools that may require specialized knowledge or are limited by complexity, T-Racing is designed to be easily accessible to local and national authorities, working in public health across various roles, including veterinary and public health institutes, veterinary services, and epidemiologists. Based on our knowledge, T-Racing has several distinctiveoffers several distinctive features that overcome the limitations of existing applications. These, including include its ability to integrate diverse data sources, its a user-friendly interface, and its scalability. This tool is designed specifically to facilitate veterinary public health institutes, services, epidemiologists, veterinary professionals, and competent authorities It integrates certified data directly from national databases, ensuring that the underlying data is both reliable and easily interpretable. This is possible because of the tool's foundation in a deep knowledge of the data and the Italian animal trade system. T-Racing allows users to explore and analyze the complex spatiotemporal pathways that shape disease spread. It enables enabling users them to draw meaningful conclusions based on a data-driven, user-guided approach.

## 2. Materials and Methods

T-Racing is an application providing users with flexibility in analyzing epidemiological data by and facilitating the usage of specific methods and tools through an interacting interface. in an environment composed by epidemiologically useful data. The main objective of T-Racing is to support veterinary epidemiological investigations in Italy by integrating and visualizing data such as outbreaks, animal movements, and other relevant information. Its flexibility lies in the ability to handle various types of epidemiologically useful data, allowing users to perform both forward and backward tracing within a spatial-temporal framework. This capability enables users to tailor analyses to their specific needs and draw meaningful conclusions based on comprehensive data integration.

**Commented [LS6]:** Rev2: besides being open source, what it allows to end users this tool?
Answer: Thank you for your comment. We have integrated the requested information into the text. Specifically, it now highlights that EpiContactTrace not only being open source, allows end users to perform forward and backward contact tracing by analyzing livestock movement data.

**Commented [LS7]:** Rev2: R shiny
Answer: Done

**Commented [LS8]:** Rev2: does it have contact tracng ?
Answer: Yes, this tool is included within the broader category of the spatial-temporal analysis tools mentioned. Both examples, EpiexploreR and EpicontactTrace, are specific examples of contact tracing applications.

**Formatted:** Space Before: 0 pt

**Commented [LS9]:** Rev2: because the abovementioned tools do not integrate heterogenius datasets - your tol is original and beyond state of the art - say it clearly what your tool bring more than what already exists
Answer: The text has been revised to include the missing information and address your comments. Specifically, in comparison to existing tools, we have developed a user-friendly tool that explores and integrates certified data directly from national databases. This tool is designed for use by public authorities and professionals in the ...

**Commented [LS10]:** Rev2: so the previous tools, the existing ones, were not useful enough for the italian services? why? you should immediately say - if that is the case if your tool will be a open source tool - if that will be the case ...

**Commented [LS11]:** Rev2: I would replace the term veterinary by animal health authorities, but its a detail
accross the entire manuscript ...

**Commented [LS12]:** Rev2: if its freely available - please say it - then I can agree we can say the tool is designed to help epdiemiologists and veterinary professionals at AH agencies to explore and analyse etc.. ...

**Formatted:** English (United Kingdom)

**Commented [LS13]:** Rev3: what is the main objective of the tool, this is a generic phrase that is ot specific to the tool? Lany tools are flexible (in which sense is yours flexible)
methods and tools for what - be precise ...

*2.1 Data sources and collection*

T-Racing gathers near-real-time data from Italian National Databases which include disease notified outbreaks obtained from the National Information System for the Notification and Management of Animal Diseases (SIMAN) which manages data on more than 100 diverse animal diseases, including both zoonotic and non-zoonotic diseases ~~(SIMAN)~~ [25], animal movements records extracted from the Italian National Database for Animal Identification and Registration (NDB) [26], and genomic data, such as Multi Loci Sequence Type (MLST), provided by National Reference Centre for Whole Genome Sequencing of microbial pathogens database and bioinformatics analysis (GenPat) [27, 28]. These systems can be accessed through the VETINFO portal (https://www.vetinfo.sanita.it/), which is a secure platform to collect and present data for managing Italy's Animal Health and Food Safety system. VETINFO provides unified access to various stakeholders, including public health institutes, veterinary services, farmers, and competent authorities. To ensure user trust and regulatory compliance, the portal strictly adheres to General Data Protection Regulation (GDPR) compliance techniques, requiring users to access it with personal credentials and mandating specific functionalities for transparent and secure data reporting. The management of these databases is the responsibility of the Istituto Zooprofilattico Sperimentale dell'Abruzzo e del Molise "G. Caporale" (IZS-Teramo) on behalf of the Italian Ministry of Health.

T-Racing collects detailed data for each reported suspected or confirmed outbreak, including the disease name (i.e., brucellosis, tuberculosis, leptospirosis, rabies, salmonellosis, campylobacteriosis, listeriosis, foot and mouth disease, african swine fever, blutongue, etc.), ~~-~~unique outbreak identifier code, geographical coordinates ~~,~~ (which are related to the outbreak location that can be a farm or another site where the infected case was identified by the veterinary service), region, province, administrative unit, suspected and confirmed outbreak date, outbreak state (i.e., suspect, extinct, and confirmed), and species involved (including animal, vector species such as mosquitoes and flies, and birds, etc.). Animal movement data includes the origin and destination unique holding identifier codes, animal single identification codes or batches of traded animals per species (including horses, cattle, buffalo, pigs, sheep and goats), and the date of the movement. The system also considers various holding attributes (livestock properties) for both origin and destination, such as the holding type (e.g., farm, slaughterhouse, staging point, transit station, market, assembly centre, pasture, foreign country, etc.), production type (e.g., milk, meat, wool, reproduction, etc.), address, and geographical coordinates. Additionally, in terms of genomic data, T-Racing records the holding code, animal single identification code, Sequence Type number, and date of sampling. Furthermore, T-Racing relies on data collected over the past 7 years extracted from the databases through daily scheduled procedures.

*2.2 Temporal Network Analysis Methods in T-Racing*

The application uses Temporal Network Analysis techniques [29] to address the dynamic nature of animal trade, enabling quick and efficient ~~backward~~ Backward~~(BCT)~~ and ~~forward~~ Forward~~(FCT)~~ Contact ~~tracing~~ Tracing strategies (BCT and FCT). In the T-Racing environment, the animal trading network is represented as a time-ordered directed and weighted network, where holdings are the nodes, animal movements between holdings on a given day are the edges, and the number of moved animals is the weight (see Fig 1 on the left).

Animal movements are recorded in the NDB at the administrative farm owner (farmer) level. Consequently, multiple farmers from the same holding may be represented as different nodes (see Fig 1, top right), causing the network to be artificially fragmented. This fragmentation significantly impacts the analysis of diseases affecting multiple animal

**Commented [LS14]:** Rev3: so this tool can only work with the italian database because otther databases in other animal health services from other countries (as you say in the end of the introdcution) should have the same database so that the tool works?

in this case, i suggest that from the start you mention that the tool is for Italy and in the discussion you talk about the possibility of extending the tool to other services (if the tool alowss this option)

answer: Yes, currently, T-Racing is specifically designed to work with Italian databases. This design choice ensures optimal functionality given the specific structure and data types of the Italian animal health system. We agree with your suggestion to emphasize the tool's current focus on Italy. We have updated the introduction to clearly state that T-Racing is tailored for use with Italian databases. Additionally, in the discussion section, we have highlighted the potential for ...

**Commented [LS15]:** Rev3: full name
answer: Done

**Commented [LS16]:** Rev3: you mean unique identifier
answer: Yes, we clarified in the text that we refer to a unique identifier.

**Commented [LS17]:** Rev3: of which palce, place of the farm?
answer: we clarified in the text that the place refers is the location of the outbreak as declared ...

**Commented [LS18]:** Rev3: of the outbreak?
answer: Yes, we clarified in the text

**Commented [LS19]:** Rev3: of the outbreak?
answer: Yes, we clarified in the text

**Commented [LS20]:** Rev3: for the origin and for the destination?
answer: Yes, we clarified in the text

**Commented [LS21]:** Rev3: for the origin and destination?
answer: Yes, we clarified in the text

**Commented [LS22]:** Rev3: you mean at a given time daily automatically updates?
answer: Yes, that's correct

**Commented [LS23]:** Rev3: Previosly
answer: We are not clear on the request. Could you please provide more context or clarify your question?

species. To address this issue, the application allows for network aggregation, ensuring that each node represents the physical holding, i.e. the geographical location to where the animals transited. Thus, a node can represent multiple owners at a fiscal level (see Fig 1, bottom right).

The framework of time-ordered networks, essentially directed acyclic graphs [29], is useful for understanding flow dynamics [30], particularly in the context of diseases spread through direct contact. We assume diseases spread along paths driven by animal movements, so accounting for the direction and timing of these movements allows us to detect temporally valid paths, providing a likely estimation of disease spread, similar to the Susceptible-Infection network-based compartmental model (where the contact rate is one). Direction and the timing of animal movements are important for detecting disease spread because they highlight impossible paths, and or too-long time elapsed for short paths [30, 31]. For example, in Fig 1, if farm A is infected at time zero, it can infect holding B, which can then infect holding C through subsequent animal movements. Since the probability of disease transmission decreases with increasing degrees of separation, we typically limit the search for plausible paths to a maximum degree of separation, or "level depth." Depending on the disease, the time elapsed between events might be too long to assume that nodes remain infectious and undetected, requiring a restriction of the time horizon to a specific number of days, known as "temporal depth." The choice of depth, in terms of both days and levels, affects the resulting subnetwork and depends on factors such as the disease's infectiousness, incubation period, and the timing of surveillance system controls. Furthermore, the removal of node(s) and/or edge(s) from a time-ordered directed network involves the change of the resulting paths. Therefore, each network modification requires a recalculation of the paths.

Contact tracing following a positive detection has been widely used in various fields, with different interpretations and applications depending on the context [30, 32]. In T-Racing, FCT, BCT, and a combination of both (FCT-BCT) strategies have been implemented to support track and control the spread of diseases. These methods provide different perspectives and insights depending on the context and time of disease outbreaks.

**FCT method** focuses on identifying the pathways of pathogen spread starting from an index node, known as the seed, and an event date. By analyzing outgoing movements within a specified depth (in terms of days and levels) FCT highlights the nodes that the disease could have potentially reached. Here, levels represent the degrees of separation from the seed node, where each level corresponds to a different stage of contact tracing (e.g., direct contacts at Level 1, and contacts of contacts at Level 2). This approach supports targeted disease control more effectively than a general surveillance system, which, despite being timely conducted, can only prevent a portion of the potential disease spread (as illustrated in Fig 2). The control shown in Fig 2 can be considered a real-time monitoring and proactive control in response to a new outbreak detection. It is performed as soon as the new outbreak is notified to detect its outgoing movements in the recent past. The subnetwork found in a forward direction will help identify positive farms and prevent the spread of infection to other farms in the future.

**BCT method**, on the other hand, aims to trace the primary source of infection from a newly detected outbreak. This retrospective strategy consists of finding temporal valid pathways through incoming movements within specific time constraints and degrees of separation. Typically, BCT uses a broader time window compared to FCT, allowing for a comprehensive analysis of the infection's origins. Both methods rely on (require) updated data. Nowadays, current Italian legislation requires the registration of animal movements in NDB within 7 days, but this does not always happen. More often, and especially for species regulated more recently, registration times are longer. This means that some of the

**Commented [LS24]:** Rev3: full name
answer: The full name is reported in revised lines 171-172 of the manuscript.

**Commented [LS25]:** Rev3: what are these leveles? I did not understand?
answer: "levels" refer to the stages of contact tracing, where each level represents a different degree of separation from the index node (seed). For example, level 1 might involve direct contacts, level 2 includes contacts of contacts, and so on. We have added a clarification in the revised manuscript to explain the term "levels"

real connections may be missing in NDB when performing FCT or BCT strategies, leading to undetected possible infectious nodes.

**Combination of strategies (FCT-BCT)** involves using both methods to enhance disease control efforts. For instance, after performing a BCT starting from multiple recent outbreaks within a large time window, the resulting subnetwork may include numerous nodes. If among these nodes there are outbreaks notified in the past, an FCT can be conducted on this newly identified BCT subnetwork. This intersection (BCT ∩ FCT) reveals the subnetwork connecting the outbreaks through temporally valid paths. Then, by working in the past, when data is more likely to be updated in NDB, this strategy identifies nodes that have been exposed to infected animals but have not yet tested positive. A comparison of animal movement dates with the last inspections on the farms can help promptly identify nodes for further inspection, aiding in containing the disease's spread. Ultimately~~In the end~~, such kind of strategies could be performed using batches of animals (~~and~~ thus letting the paths highlight the spread accounting for transmission between animals sharing the same farms) or considering the individual animals moving forward or backward from a seed farm. These strategies can be applied either to batches of animals (highlighting the spread through shared farm contacts) or to individual animals moving between farms. The T-Racing application facilitates these analyses, enabling users to leverage epidemiological information and expert knowledge to transform vast amounts of data into clear, actionable guidelines for disease control.

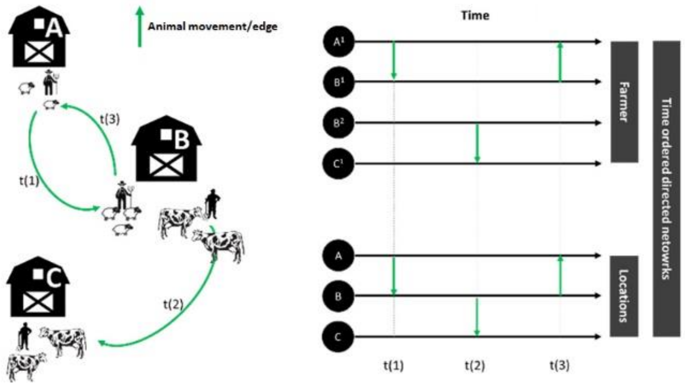


**Fig 1** Network fragmentation and aggregation in animal movement records. The top right panel shows how multiple farmers of the same holding are represented as different nodes, leading to artificial fragmentation. The bottom right panel demonstrates the aggregation process, where each node represents the physical holding, encompassing multiple owners at a fiscal level.

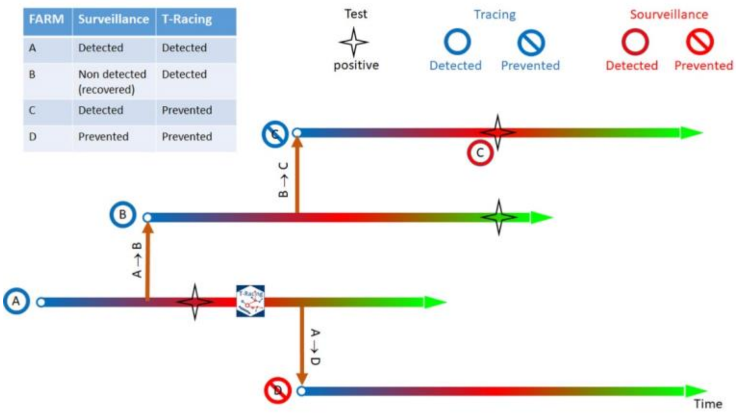


**Fig 2** Schematic illustration of the importance of tracing in preventing and detecting diffusion pathways versus the simple farm isolation when only positivity recorded in the context of the surveillance program is considered.

### *2.3 T-Racing Design*

The T-Racing interface allows users to quickly query, filter, analyze, and visualize data through interactive graphs, maps, and tables for a seamless and intuitive experience. Fig 3 provides a conceptual map of the application, highlighting its primary dashboard sections and features.

2.3.1 Tools for extracting and viewing animal trade data~~, including:~~

- **Filtering data** based on multi-species selections, time frame, tracing direction mode (backward or forward methods), network geo-aggregation mode, and including/excluding animal trade data from/to foreign countries or slaughterhouses;
- **Multi-seed selection** for tracing, allowing users to either type or select one or more farm codes from the list of farms or select outbreak codes from the list of officially notified outbreaks within a specified time frame. Alternatively, the user can select all farms and/or outbreaks falling within a spatial buffer (neighbours distant by kilometres) around selected farms and outbreaks;
- **Tracking a single animal** by typing its identification code and viewing all resulting spatial-temporal paths within a specified time frame.

The system retrieves movement data based on these filters from the NDB and builds a time-ordered directed weighted network originated by the set seeds. These tools are useful for analyzing diseases that are transmitted through direct contact (i.e. brucellosis or tuberculosis) and diseases that are transmitted through spatial proximity (i.e. African swine fever or vector-borne diseases such as west Nile or Bluetongue, etc.), using spatial buffering and contact tracing methods.

2.3.2 Tools for customizing network visualization and optimization~~:~~

- **Customizing graph visualization** by setting from a range of layout options such as hierarchical, geographical, or nicely mode (default mode). Users can also ~~make~~ select the shape and colour of nodes or the colour of edges to reflect chosen~~selected~~ data attributes such as holding type or production type for nodes, and level, time, or species for edges. All possible attribute colours and shapes are predefined in a configuration file;

- **Removing** unnecessary **nodes or edges** from performed tracing;
- **Highlighting within the network all edges involving animals moved from/to the seed, or reducing the network to only those edges involving animals moved from/to the seed**, according to the tracing direction mode set by the user;
- **Reducing the network size** by decreasing network deepness (in days or levels).

For each network reduction method applied, a new time-ordered network will be ~~created~~ recalculated to ensure and maintain the temporal coherence in the events.

2.3.3 Tools for epidemiological information integration and results printing~~:~~

- **Adding a layer** of sequenced pathogens for the animals, and/or ~~;~~
- ~~**Adding a layer**~~ of outbreaks for a specific disease **or reducing the resulting network** by keeping only temporally valid paths among involved outbreaks by a combination of FCT and BCT methods;

- **Download the network's tables** (~~or a selection of them~~based on selection) in Excel format;
- Storing the results in a **dynamic HTML report** for offline use.

By using these tools, the network graph can be customized and fine-tuned to provide a more meaningful representation of the data.

~~The T-Racing application was created by a team of experts including epidemiologists, veterinarians, mathematicians, and computer scientists. This collaborative effort spanned all stages of development, from gathering data to creating tools and building a user-friendly interface. The team has extensive experience in epidemiological tracing and dynamic network analysis, as evidenced by similar web applications they have developed since 2011 [24, 33, 34, 35, 36]. T-Racing is an improved version of previous applications, offering faster speeds, additional features, and integration of various epidemiological datasets. Currently, it is available on the internal network of IZS-Teramo and used by our veterinarians and epidemiologists. It is pending technical adjustments and official approval from the Italian Ministry of Health to be integrated into the VETINFO portal. This integration will ensure secure access and the protection of sensitive data, as T-Racing inherits the robust GDPR techniques already applied in the portal.~~

**Commented [LS26]:** Rev3: Move in the discussion
answer: Done

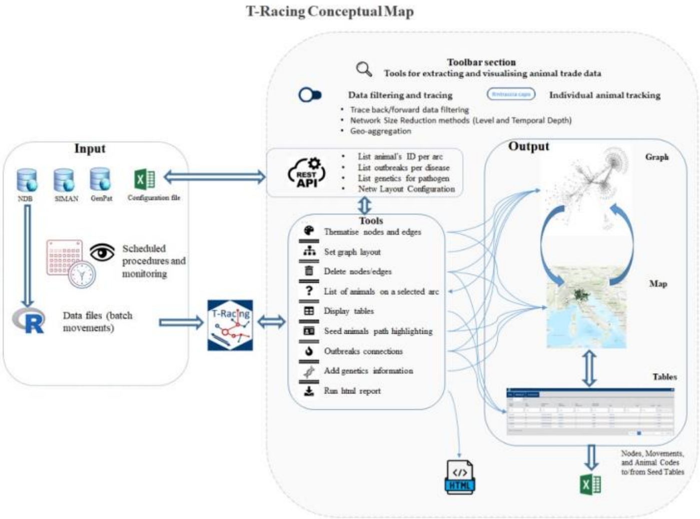


**Fig 3** T-Racing conceptual map.

*2.4 Data flow and system architecture*

T-Racing has been developed using the Shiny library by the R statistical environment, and leaflet and visnetwork libraries were used for map and graph visualization respectively (Table 1 contains a list of all packages used). The application manages large amounts of data from different information systems and shares them efficiently and securely in real-time. Differently from the serverless JavaScript web apps, computationally heavy services are executed on a remote server, and the Plumber package allows the application to query data sources in real-time, through web API.

The system architecture facilitates the application scalability, thanks to shiny proxy (a free and open-source Docker-based application). The described stack and the involved application modules are displayed in Fig 4.

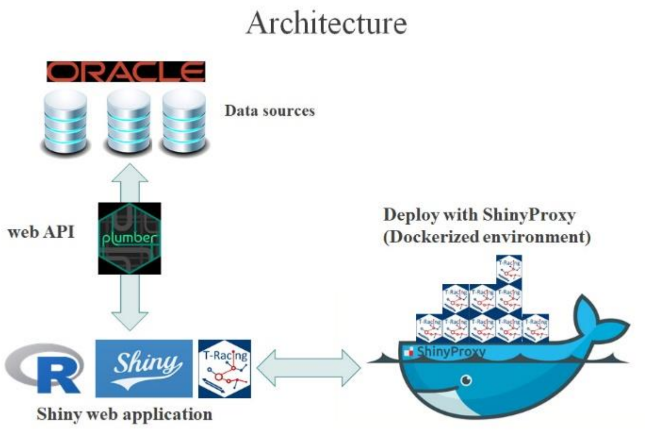


**Fig 4** T-Racing architecture.

**Table 1.** List of the R-packages used in T-Racing development.

| **R-package** | **Use** | **Reference** |
|---|---|---|
| shiny (v. 1.7.1) | UI/SERVER | Winston Chang, Joe Cheng, JJ Allaire, Carson Sievert, Barret Schloerke, Yihui Xie, Jeff Allen, Jonathan McPherson, Alan Dipert and Barbara Borges (2021) |
| shinyjs (v. 2.1.0) | UI/SERVER | Dean Attali (2020). shinyjs: Easily Improve the User Experience of Your Shiny Apps in Seconds |
| shinyWidgets (v. 0.6.4) | UI | Victor Perrier, Fanny Meyer and David Granjon (2021). shinyWidgets: Custom Inputs Widgets for Shiny |
| Shinydashboard (v. 0.7.2) | UI | Winston Chang and Barbara Borges Ribeiro (2018). shinydashboard: Create Dashboards with 'Shiny' |
| shinyBS (v. 0.61.1) | UI | Eric Bailey (2015). shinyBS: Twitter Bootstrap Components for Shiny |
| shinybusy (v. 0.3.0) | UI/SERVER | Fanny Meyer and Victor Perrier (2020). shinybusy: Busy Indicator for 'Shiny' Applications |
| DT (v. 0.22) | UI | Yihui Xie, Joe Cheng and Xianying Tan (2021). DT: A Wrapper of the JavaScript Library 'DataTables' |
| rintrojs (v. 0.3.0) | UI/SERVER | Carl Ganz (2016). rintrojs: A Wrapper for the Intro.js Library. Journal of Open Source Software, 1(6), October 2016 |
| shinycssloaders (v. 1.0.0) | UI/SERVER | Andras Sali and Dean Attali (2020). shinycssloaders: Add Loading Animations to a 'shiny' Output While It's Recalculating |
| htmltools (v. 0.5.2) | UI | Joe Cheng, Carson Sievert, Barret Schloerke, Winston Chang, Yihui Xie and Jeff Allen (2021). htmltools: Tools for HTML |
| leaflet (v. 2.1.1) | UI | Joe Cheng, Bhaskar Karambelkar and Yihui Xie (2022). leaflet: Create Interactive Web Maps with the JavaScript 'Leaflet' Library |
| rgdal (v. 1.5.31) | SERVER | Roger Bivand, Tim Keitt and Barry Rowlingson (2021). rgdal: Bindings for the 'Geospatial' Data Abstraction Library |
| sp (v. 1.4.7) | SERVER | Pebesma, E.J., R.S. Bivand, 2005. Classes and methods for spatial data in R. R News 5 (2) |
| bezier (v. 1.1.2) | SERVER | Aaron Olsen (2018). bezier: Toolkit for Bezier Curves and Splines |
| geosphere (v. 1.5.14) | SERVER | Robert J. Hijmans (2021). geosphere: Spherical Trigonometry |
| visNetwork (v. 2.1.0) | UI | Almende B.V., Benoit Thieurmel and Titouan Robert (2019). visNetwork: Network Visualization using 'vis.js' Library |
| igraph (v. 1.3.1) | SERVER | Csardi G, Nepusz T: The igraph software package for complex network research, InterJournal, Complex Systems 1695. 2006 |
| ggplot2 (v. 3.3.6) | SERVER | H. Wickham. ggplot2: Elegant Graphics for Data Analysis. Springer-Verlag New York, 2016 |

**Commented [LS27]:** Rev3: I would add this in the supp material
answer: Thank you for your suggestion. We prefer to keep the table listing the R packages used for the development of the application within the main text because we believe this ensures that readers have immediate access to details about the tools and resources utilized in developing T-Racing. We think this is crucial for understanding the complexity of implementation and functionality of the application.

| | | |
|---|---|---|
| RColorBrewer (v. 1.1.3) | SERVER | Erich Neuwirth (2014). RColorBrewer: ColorBrewer Palettes |
| xlsx (v. 0.6.5) | SERVER | Adrian Dragulescu and Cole Arendt (2020). xlsx: Read, Write, Format Excel 2007 and Excel 97/2000/XP/2003 Files |
| dplyr (v. 1.0.9) | SERVER | Hadley Wickham, Romain François, Lionel Henry and Kirill Müller (2021). dplyr: A Grammar of Data Manipulation |
| jsonlite (v. 1.8.0) | SERVER | Jeroen Ooms (2014). The jsonlite Package: A Practical and Consistent Mapping Between JSON Data and R Objects. arXiv:1403.2805 |
| curl (v. 4.3.2) | SERVER | Jeroen Ooms, Hadley Wickham, RStudio |
| rJava (v. 1.0.6) | UI | |

*2.6 Demo*

The T-Racing application is not publicly available as it handles sensitive data from Italian national information systems. Therefore, a demo version has been developed to demonstrate its potential to the scientific community. This demo ~~uses~~ operates with a pre-configured ~~limited and anonymized~~ dataset, ~~-~~ which has been ~~A~~adjust~~ments~~d ~~have been made~~ by adding noise and rounding coordinates to the second decimal place in the georeferencing of holdings, which may result in some holdings falling outside the Italian boundaries. ~~Additionally, it~~The demo has been translated into English, while the original application is in Italian. The original development and publication environment, as well as the web services, have been duplicated and adapted for the demo. The underlying dataset is contained in a single Oracle database. The demo version is intended to allow users to explore and replicate the results presented in our study, ~~environment~~ provid~~ing~~es a comprehensive preview of its features~~,~~ as listed in Table 2.

**Commented [LS28]:** Rev3: where can we find this demo?
answer: The link to the demo is provided at the beginning of the Results section. We have included it there to ensure that readers can easily access and review the demonstration of the application.

**Commented [LS29]:** Rev3: the demo is it funcional? does it allow a person to upload its own network data, from their own country and run the tool ?
answer: Thank you for your question. As stated in the manuscript: "The T-Racing application is not publicly available as it handles sensitive data from Italian national information systems. Therefore, a demo version has been developed to demonstrate its potential to the scientific community." The demo is intended solely for illustrative purposes and does not allow users to upload their own network data. It utilizes a pre-configured, anonymized dataset focused on two specific diseases—Brucellosis and Tuberculosis—to enable readers to replicate the results reported in the study.

**Table 2.** List of Data available in T-Racing Demo.

| Data | From | To | Type/feature |
|---|---|---|---|
| **Movements** | 01/12/2017 | 30/06/2023 | batch and individual for bovine and buffalo |
| **Outbreaks** | 01/12/2017 | 30/06/2023 | *Brucellosis*, *Tuberculosis* |
| **ST** | 01/01/2015 | 01/12/2018 | *B. abortus* and *B. melitensis* species |

*2.5 Brucellosis and Tuberculosis: Rationale for Case Studies and Italian Regulations*

The following case studies were considered:

**Brucellosis in the Marche region (central Italy)**. On December 9, 2019, a brucellosis outbreak was confirmed in central Italy, specifically in the Marche region. The T-Racing application was utilized to identify the potential source of the infection. Only bovine species were considered, while slaughterhouses and foreign countries were excluded from the analysis. The known outbreak code from a list of proposed brucellosis outbreaks was selected as the seed for a trace backwards analysis encompassing all incoming movements to the identified outbreak over the past two years at 4 depth levels. The resulting network was refined using the "outbreaks connection" tool, and genetic data was added to support the conclusions drawn from the final network.

**Tuberculosis outbreaks affected Elba Island and the nearby Tuscany coast** in June 2023. A forward tracing from March to December 2023 to identify the likely spread trajectory leading up to the outbreak on Elba Island was used. Only bovine species were considered, slaughterhouses were included, and foreign countries were excluded from the analysis. Additionally, we utilized a "Seed animal's path highlighting" tool to emphasize the flow of the infection about the animals coming from the seed.

**A tuberculosis outbreak occurred in the Cuneo province in December 2022**. A trace backward analysis over a 13-month temporal window with a level depth of 4 was used to determine the most likely source of infection. Network reduction was applied using the dedicated tools for highlighting paths among involved outbreaks ("outbreaks connection" tool).

We used T-Racing to investigate brucellosis and tuberculosis outbreaks in non–endemic areas of Italy, where an occurrence of an outbreak should trigger a rigorous investigation by the official veterinary service to prevent the disease from spreading, demonstrating the speed and effectiveness of the investigation carried out with the proposed web app.

Brucellosis caused by *Brucella abortus* is an important zoonoses worldwide, and tuberculosis caused by *Mycobacterium bovis* ~~are important zoonoses that~~ constitute a serious hazard to public health in developing countries and a great economic burden to livestock production in Europe. These diseases~~, and~~ were ~~the~~ chosen ~~diseases~~ due to their public health and economic importance and their chronic development, allowing for the use of FCT and BCT methods with a broad time-elapse. Prevention of these zoonoses in humans depends on the control of the disease in animals. Livestock movement data represent a valuable source of information to understand the pattern of contacts between holdings, which may determine the spread of such diseases. The first national plans for the control of bovine tuberculosis and brucellosis were approved with the Ministerial Decree of 11 and 12 March 1965, respectively. Through the Ministerial Decree of 27 August 1994 n. 651 entered into force in Italy the national eradication plan for bovine brucellosis, which establishes the measures to be applied to cattle farms to obtain the eradication of the disease for the protection of public health and protection of officially free herds. The regulation determines for control the use of official serological tests twice a year at an interval of not less than three and not more than six months. All animals over the age of twelve months in all cattle farms have to be tested, even if are located in the wild. Similar procedures have been established (test all animals in all heads at once, with 3 to 6 months intervals) with Ministerial Decree 15 December 1995, n. 592 – Regulation concerning the national plan for the eradication of tuberculosis in cattle and buffalo herds – by testing with the tuberculin intradermal test.

## 3. Results

T-Racing Demo, which uses a limited and anonymized dataset, is available at this link https://demo.izs.it/app_direct/tracing/. The basemaps throughout all the following figures were created using ArcGIS® software by Esri and integrated into the shiny web application using the leaflet.providers R library, sourced from leaflet-providers.js (https://github.com/leaflet-extras/leaflet-providers). ArcGIS® and ArcMap™ are the intellectual property of Esri and are used herein under license. Copyright © Esri. All rights reserved. For more information about Esri® software, please visit www.esri.com.

Fig 5 shows the T-Racing interface, which displays temporally valid paths originating from a brucellosis outbreak in Sicily, an Italian southern island, using the trace forward method. The investigation revealed this outbreak, detected in July 2020, was likely the source of two subsequent outbreaks in Pisa, Tuscany, northern Italy. Animals moved from the seed in March reaching, via a transit station, two farms in Pisa in April and May, but the outbreaks were only detected by surveillance in December 2020. The simultaneous visualization of the queryable and interactive graph, map, and tables, combined with the iterative use of tools for network calibration and targeted result personalization, offers a more comprehensive and user-friendly result viewing.

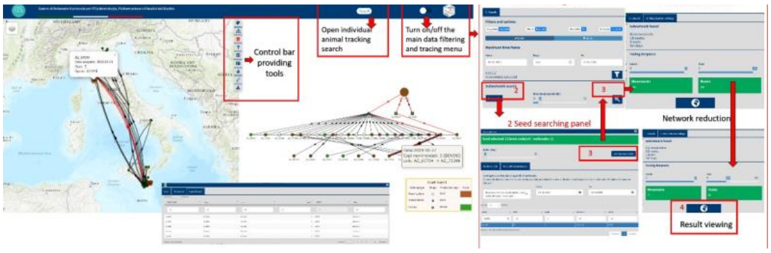


**Fig 5** The T-Racing interface (on the left) shows the subnetwork resulting from the trace forward analysis on a map, graph, and tables and the steps followed to get the results in the interface are shown on the right. This analysis was performed using a brucellosis outbreak in July 2020 in the Sicily Region as the seed, extracting all bovine movements in 2020 up to the third level of depth (three degrees of separation). A time frame-based network reduction/optimization method was applied for the result. The "hierarchical" graph layout shows all nodes and edges in the subnetwork, highlighting probable disease pathways and nodes reached during the study period. Node shape and colour represent farm type and production activity (see legend). The larger, red-bordered icon represents the seed, with each node labelled by farm code. Arrows indicate animal movements between two nodes, with the width proportional to the number of animals moved. Red-coloured arrows highlight paths followed by animals moving from the seed (using the “Seed animals path highlighting” tool). Fire icons on the map represent brucellosis outbreaks detected during the selected period and included in the subnetwork (using the "Outbreaks connections" tool). Tooltips and tables provide detailed information, facilitating navigation between the map, graph, and tables.

### *3.1 T-Racing - Case Studies*

3.1.1. Tracing backward from a notified outbreak of brucellosis in the Marche region of central Italy, in December 2019, to identify its potential sources of infection.

The search for the subnetwork resulted in 1.825 movements involving 386 nodes. The subnetwork covers a substantial area of central Italy, including holdings that reach the seed even from the islands of Sicily and Sardinia. The theming of nodes based on type reveals the involvement of different holding types. In addition to breeding farms, there are pastures (structures that play a significant role in the spread of brucellosis) and a hub node, a genetic collection centre located in Perugia province, Umbria region, in central Italy, that connects 132 nodes (Fig 6). To make the network more manageable and relevant for the conducted tracing, a subnetwork was created by setting 3 levels and reducing the depth to 399 days, resulting in 198 movements and 38 nodes. To identify plausible paths of disease spread, the application allows the utilization of notified outbreaks within a customizable time frame for the specific disease. These outbreaks are visualized on the map, enabling the tracing to be narrowed down to the connections between outbreaks included in the tracing. This helps identify a focused subset of connections to investigate. The subnetwork includes multiple brucellosis outbreaks, primarily located in the southern and central regions. The reduced network, created by keeping only temporally valid paths among involved outbreaks within the network, consists of 94 movements involving 11 nodes. At this point, we can assess the presence of genetic information as further confirmation of the tracing's plausibility. To do so, we utilize the tool by adding the available sequence type for *Brucella abortus*. The seed is an outbreak for which sequence type "2" is available, and the identified origin zones are also affected by clusters of the same sequence type. An interesting aspect is to determine how many of these links involve the same animals that reached the seed. The dedicated tool allows for theming the links based on this characteristic by searching for individually identified animals. The visualization highlights on the subnetwork the paths involving the same animals that reach the seed. These highlights paths consist of only 21 movements involving 5 nodes (including the seed and one pasture, which is the presumed source of infection). Further investigation of individually identified and traced animals revealed that an infected

animal, identified as sequence type "2" in the seed, originated from a pasture, which was subsequently identified as the plausible source of the sought-after infection (Fig 7).

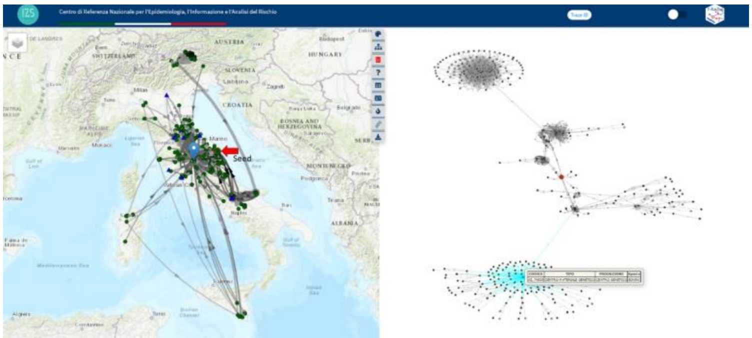


**Fig 6** Trace backward method. Sequential investigation steps: 1) Data Filter, 2) Set seed by outbreak code search, and 3) Network view. The trace-back focusing on bovine species, excluding slaughterhouses and foreign countries, was conducted to trace the outbreak occurring in December in the Marche region, in Italy. 1,825 movements involving 386 nodes were identified within a broad time spanning the past two years. The seed is contoured in red. The network covers a substantial area of central Italy, including farms extending to the islands (Sicily and Sardinia). Node theming based on structure type highlights diverse holding types, including pastures and a genetic collection centre in Perugia, highlighted in light blue on the graph (simultaneously, its marker appears on the map).

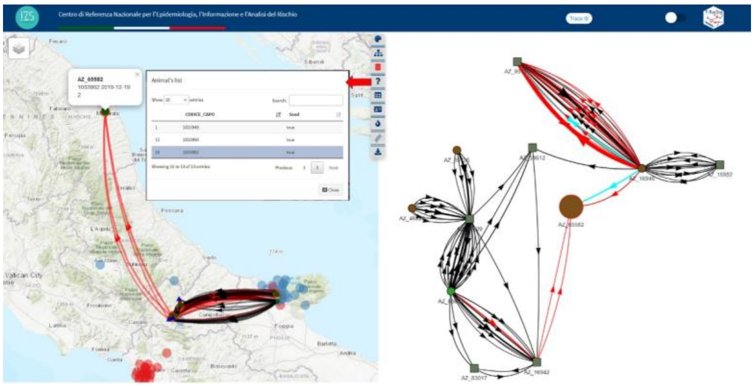


**Fig 7** The subnetwork result after the following four sequential steps: 1) Network reduction by setting the network level to 3 and days depth to 399 (the new subnetwork size is 198 links and 38 nodes), 2) Further reducing the subnetwork by keeping only temporally valid paths among involved outbreaks (resulting in a subnetwork size of 94 links and 11 nodes; the four detected brucellosis' outbreaks involved are shown on the map with red fire icons), 3) Adding the sequence type for Brucella abortus layer to the map (type 2 in blue circles) and displaying a popup on the seed showing the identification code of the animal, sampled date, and detected Brucella abortus sequence type, and 4) Highlighting on the graph are all links involving the same animals that reached the seed (red links). Further investigation on "individually identified animals," found by querying the highlighted links (in light blue on the graph), revealed that an infected animal originating from the pasture reached the seed. This animal is listed in the Animal list produced by the application, matching the animal reported in the map popup.

3.1.2. Tracing the flow of tuberculosis outbreaks: Elba Island and Cuneo Province.

**-Four BTB outbreaks were notified in June 2023 in a small, non-endemic area, in Italy, affecting Elba Island and the nearby Tuscany coast.** Using T-Racing allowed for the detection of the real flow of the disease spread. The resulting network highlighted an animal movement of nine cattle in April 2023, which reached farms on the nearby coast. This established the real source of the infection and the most likely flow of the spread (Fig 8). We discovered that the animals reaching the outbreaks were the same ones that had left Elba Island and one of them (at the time of writing this article) had not yet been slaughtered.

-**In December 2022, an outbreak of BTB occurred in the Cuneo province of the Piedmont region in northern Italy.** The resulting network comprised 306 farms and 664 connections, predominantly located in the Sicily region. For a more focused investigation, we considered only temporally valid paths connecting BTB outbreaks included in the network resulting in a subnetwork of 50 connections involving 9 breeding farms in the Sicily region, four of which were BTB outbreaks, and two pastures. The investigation suggested that four outbreaks occurring in the previous months are the most likely source of infection. Additionally, the 119 animals reaching the Cuneo province in June and July originated from a fattening farm where they were likely infected by mixing with animals coming from the four outbreaks. The results indicate the need for targeted control measures for the other five farms involved in the revealed diffusion paths (Fig 9).

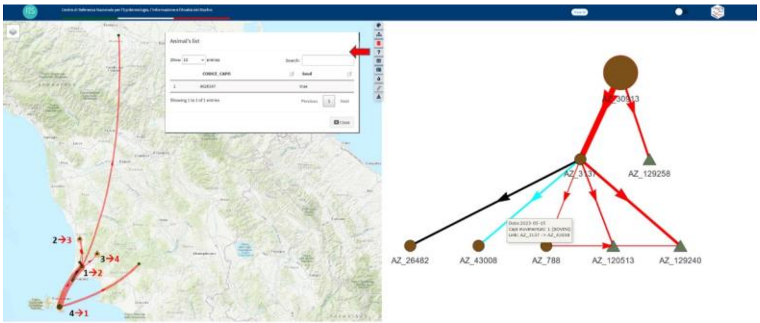


**Fig 8** Trace forward method. The map and graph show outbreaks that occurred on Elba island and the nearby Tuscany coast (black numbers '1' to '4', represent the notification timing, with 1 the earliest and '4' the most recent notified one). The network of animal movements involving the outbreaks is displayed with red links indicating the presence of at least one animal originating from the seed, and black link representing the movement of animals not originating from the seed. The notified outbreaks' timing contrasts with the real flow of disease spread revealed by T-Racing analysis (red numbers '1-4'). The triangles on the graph are slaughterhouses. Further investigation on "individually identified animals," found by querying the highlighted link (in light blue on the graph), revealed that one animal originating from the seed had not yet been slaughtered. This animal is listed in the Animal's list produced by the application.

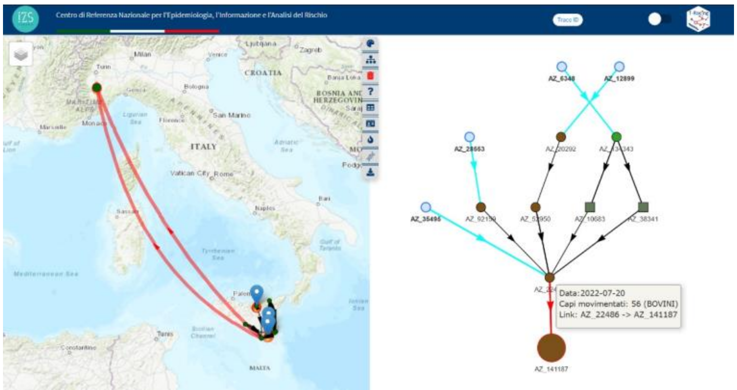


**Fig 9** Trace backward method. The network resulting from the BTB outbreak occurred in Cuneo province, in the Tuscany region in December 2022 involving 306 farms and 664 links, mostly located in the Sicily region. The map and graph show the reduced network by considering only temporally valid paths connecting involved outbreaks consisting of 50 connections and 12 holdings which 9 breeding farms in the Sicily region (circles on the graph), four of which are BTB outbreaks (fire icons and marked on the map and highlighted in light blue on the graph), and two pastures (squares on the graph). The links involving animals reaching the Cuneo outbreak are in red colour.

## 4. Discussion

The T-Racing application was created by a team of experts including epidemiologists, veterinarians, mathematicians, and computer scientists. This collaborative effort spanned all stages of development, from gathering data to creating tools and building a user-friendly interface. The team has extensive experience in epidemiological tracing and dynamic network analysis, as evidenced by similar web applications they have developed since 2011 [24, 33, 34, 35, 36]. T-Racing is an improved version of previous applications, offering faster speeds, additional features, and integration of various epidemiological datasets. Currently, it is available on the internal network of IZS-Teramo and used by our veterinarians and epidemiologists. It is pending technical adjustments and official approval from the Italian Ministry of Health to be integrated into the VETINFO portal. This integration will ensure secure access and the protection of sensitive data, as T-Racing inherits the robust GDPR techniques already applied in the portal.

Timely identification of outbreaks and systematic contact tracing can be effective in stopping the transmission chain of contagious diseases, especially with strong collaborations between epidemiological teams, laboratory surveillance, and decision-making officials, improving outbreak management, in a One Healthmultidisciplinary context [37].

–An important feature of the modern response to epidemics is the increasing focused on exploiting all available data to inform the response in nearly real-time and to allow for the data evidence-based decision-making [38]. The capability of T-Racing to handle zoonotic diseases such as brucellosis, tuberculosis, and others under veterinary surveillance in Italy highlights its crucial role in the One Health approach. By effectively managing and analyzing data related to these diseases, T-Racing not only aids in preventing and controlling the spread of infections across species but also supports broader public health initiatives. This functionality is particularly important given the complex nature of zoonotic diseases, which can have significant impacts on both animal and human populations. T-Racing has the potential to significantly enhance animal disease control measures, as it can rapidly organize crucial contact information from extensive datasets and identify connections in a multi-outbreak investigation. It

introduces new methods and tools currently not available in other systems or tools [2, 24, 33, 34, 35, 36]. These include multi-seed/species selection, seed selection using a spatial buffer around cases or outbreaks, and the combination of strategies (FCT-BCT) configuring itself as an innovative application in its field. Thus, the application efficiently supports epidemiological investigations, as it was shown in the demo examples for brucellosis and tuberculosis. In these examples, it is possible to see how deep into the transmission chain the T-Racing allows us to explore. The initial tracing example has identified a likely path of disease spread and the presumed origin, starting from a relatively large and diverse network (1.825x386) in both spatial and temporal dimensions. By using interactive and iterative tools, along with additional information such as disease outbreaks, genetic data, and movements of individually identified animals, it was possible to narrow down the focus to 21 movements and 5 nodes in just a few steps, saving a significant amount of time. Trying to achieve the same result by simply extracting the movement network through a query of the NDB would have been extremely difficult, like finding a needle in a haystack. The second example clarified the actual timing of the tuberculosis spread. Importantly, the animals from the seed had not been slaughtered at the time of writing this article, and in the last example, results indicate a need for targeted control measures for the other five farms involved in the revealed diffusion paths.

Considering chronic diseases, such as brucellosis and tuberculosis, clarifying the disease's spread patterns allows for focus measures of control, improving targeting financial and human resources, as well as time sensitive decisions. Noteworthy, the almost real-time data used, the possibility of analyzing many datasets at once and the user-friendly environment are also features that could greatly advance responses to emergencies with acute and highly contagious diseases when movement data could be generated in real-time.

The integrationComplementarily, the application of molecular epidemiological data into the analysis complements, the validating validation the of identified disease (brucellosis) transmission paths of disease (brucellosis) spread, exemplifyingis an example of a One Health approach developed by a multidisciplinary team to advance disease control on building a new technology for disease control. In this sense, in an era of rapid technological advancements, fostering innovation in epidemiological investigations is essential, as it can strengthen trust between the veterinary and human health systems in addressingit is essential to foster innovation and develop tools that revolutionize epidemiological investigations, by combining cutting edge technology with comprehensive data analysis, which could benefit trust between the Veterinary and Human Health systems while dealing with zoonosis [38]. T-Racing empowers clinicians, veterinarians, ecosystem health professionals, researchers, and policymakers to leverage the power of animal surveillance data for more comprehensive, integrated disease control strategies within the One Health perspective [3, 39], enabling informed decisions and proactive measures to mitigate the impact of zoonotic diseases. This tool's data-driven nature underscores the importance of accurate data management, as reliable data is crucial for effective and trustworthy epidemiological investigations.In that regard, by embracing this innovative tool, stakeholders can gain valuable insights, make informed decisions, and take proactive measures to mitigate the impact of infectious diseases on animal and human health. Therewith, this dynamic approach enables the T-Racing application to provide accurate and timely data for epidemiological investigations. Complementary, the system emphasizes its data driven nature, recognizing the crucial role of accurate data in conducting effective investigations and highlighting the need for robust practices on data management. Noteworthy, without reliable data, the utility and reliability of the T-Racing application would be compromised.

Although T-Racing is currently operational, it does have some limitations that are important to consider. The application's specific structure, tailored to the Italian context,

limits its broader usability.the authors and team of experts will continue to develop future versions to improve the application by adding new datasets and functionalities. At present, the genetic data is restricted to the Brucella genus, but additional pathogen data will be incorporated as soon as it becomes available in GenPat. Data regarding testing from surveillance systems on farms will be included, and functions will be created to factor in the timing of testing when selecting the most probable disease spread paths. Also, detailed information about single animals, like demographics and parturition dates for calves, along with human cases will be considered. There is also an evaluation underway for the development of scheduled algorithms that will operate directly on the database to identify critical networks to be automatically provided to T-Racing's users. This will enable more efficient, rapid, and straightforward validation and initiation of investigations. However, the authors and team of experts will continue to develop future versions to improve the application by adding new datasets and functionalities. Concurrently, work is ongoing for a more general application that allows users to upload their own data, and its source code will be freely distributed on GitHub.

## 5. Conclusions

The T-Racing web app represents a modern and forward-thinking approach to support epidemiological investigations in animal disease outbreaks in Italy. It provides a unified environment to manage and analyze the expanding pool of data sources iteratively by integrating advanced technology, comprehensive data management, and cutting-edge analytics. T-Racing has the capability to draw meaningful conclusions based on a data-driven, user-guided approach while leveraging its expertise. It facilitates evidence-based decision-making and serves as a leading tool in disease control, enhancing our capacity to address emerging challenges and safeguard global health from a One Health perspective.

**Author Contributions:** Conceptualization, L.C., L.S., A.C., P.C. and F.D.M.; methodology, L.C. and L.S.; software, L.C. and S.P.; validation, F.D.M. and P.C.; data curation, D.P.; writing—original draft preparation, L.S., L.C., A.C.T.R.B.C. and F.D.M.; writing—review and editing, L.C., L.S., A.C., F.D.M., P.C., S.P., A.D.L., D.P., S.T., S.S., and M.T.; project administration, P.C.; funding acquisition, P.C.. All authors have read and agreed to the published version of the manuscript.

**Funding:** This research was funded by Italian Ministry of Health (RC MSRCTE0219).

**Conflicts of Interest:** The authors declare no conflict of interest.

**Commented [LS30]:** Rev3: Currently the tool is not generic, or its made to be generic, but you started to feed the tool only with the Brucella data on national level?
answer: T-Racing is designed to be a generic tool, capable of visualizing genetic data for various pathogens as it becomes available. Currently, the tool is connected to the GenPat database, where the only genetic data available is for the Brucella genus. However, as soon as additional pathogen data is included in GenPat, T-Racing will automatically be able to visualize and analyze this new data without any further modifications needed to the tool.

**Commented [LS31]:** Rev3: I would like to see the code and tool being distributed as open source - rather than being set at the end of the discussion as a "promisse"
answer: We appreciate your feedback regarding making the code and tool open source. As noted, we understand the significance of open-sourcing the application and fully acknowledge the additional effort and time required to prepare the code for public release. We assure you that this is a priority, and it is included in our roadmap for future developments.
In the meantime, we believe that it is valuable to share and promote the T-Racing application as it currently stands. This allows us to gather feedback from the community and refine the tool further, while working towards open-sourcing it.

**Commented [LS32]:** Rev3: what are the limitations of the work? this is important to be discussed before we move into the conclusions
answer: Thank you for your feedback. The limitations of T-Racing were indeed discussed in the original manuscript. However, we have now made these limitations more explicit in the discussion section to ensure they are clearly communicated. We have emphasized that the application's specific design for the Italian context limits its broader usability in other countries.

## References


1 Schirdewahn, F., K. Lentz, H. H., Colizza, V., Koher, A., Hövel, P., & Vidondo, B. Early warning of infectious disease outbreaks on cattle-transport networks. PLOS ONE, 16(1), e0244999. https://doi.org/10.1371/journal.pone.0244999

2 Maria Nöremark and Stefan Widgren. EpiContactTrace: an R-package for contact tracing during livestock disease outbreaks and for risk-based surveillance. BMC Veterinary Research 2014, 10:71 Page 2 of 9 http://www.biomedcentral.com/1746-6148/10/71

3 Prata, J. C., Ribeiro, A. I., & Rocha-Santos, T. (2022). An introduction to the concept of One Health. One Health, 1-31. https://doi.org/10.1016/B978-0-12-822794-7.00004-6

4 Wondwossen A. Gebreyes, Daral Jackwood, Celso Jose Bruno de Oliveira, Chang-Won Lee, Armando E. Hoet, Siddhartha Thakur. Molecular Epidemiology of Infectious Zoonotic and Livestock Diseases. 2020. Microbiology Spectrum, 10.1128/microbiolspec.ame-0011-2019, 8, 2, https://journals.asm.org/doi/abs/10.1128/microbiolspec.ame-0011-2019

5 Neo, J. P. S., & Tan, B. H. (2017). The use of animals as a surveillance tool for monitoring environmental health hazards, human health hazards and bioterrorism. Veterinary Microbiology, 203, 40-48. https://doi.org/10.1016/j.vetmic.2017.02.007

6 D Kriebel, J Tickner, P Epstein, J Lemons, R Levins, E L Loechler, M Quinn, R Rudel, T Schettler, A M Stoto. The precautionary principle in environmental science. 2001, Environmental Health Perspectives, 871-876, 109, 9, https://ehp.niehs.nih.gov/doi/abs/10.1289/ehp.01109871

7 Rocha VC Figueiredo, Souza-Filho AF, Ikuta CY, et al. High discrimination of Mycobacterium bovis isolates in Brazilian herds by spoligotyping. **Preventive Veterinary Medicine**, v.179, p.104976, 2020. Doi: 10.1016/j.prevetmed.2020.104976.

8 Taye H, Alemu K, Mihret A, Wood JLN, Shkedy Z, Berg S, Aseffa A. Global prevalence of Mycobacterium bovis infections among human tuberculosis cases: Systematic review and meta-analysis. Zoonoses Public Health. 2021 Nov;68(7):704-718. doi: 10.1111/zph.12868. Epub 2021 Jun 24. PMID: 34169644; PMCID: PMC8487997.

9 Laine CG, Johnson VE, Scott H, Arenas-Gamboa AM. Global Estimate of Human Brucellosis Incidence. Emerg Infect Dis. 2023;29(9):1789-1797. https://doi.org/10.3201/eid2909.230052

10 Ansari, S., Heitzig, J., Brzoska, L., Lentz, H. H., Mihatsch, J., Fritzemeier, J., & Moosavi, M. R. (2021). A Temporal Network Model for Livestock Trade Systems. Frontiers in Veterinary Science, 8. https://doi.org/10.3389/fvets.2021.766547

11 Loria GR, Migliore S, Bongiorno C, Ciaccio G and Laddomada A (2022) New European rules introduced by regulation (EU) 2016/429 to facilitate animal trade: With great risk comes great responsibility. Front. Vet. Sci. 9:1003732. https://doi.org/10.3389/fvets.2022.1003732

12 Porphyre, T., Boden, L. A., Correia-Gomes, C., Auty, H. K., Gunn, G. J., & J. Woolhouse, M. E. (2016). Using national movement databases to help inform responses to swine disease outbreaks in Scotland: The impact of uncertainty around incursion time. Scientific Reports, 6. https://doi.org/10.1038/srep20258

13 Regulation (EU) 2016/429 of the European Parliament and of the Council of 9 March 2016 on transmissible animal diseases and amending and repealing certain acts in the area of animal health ('Animal Health Law') (Text with EEA relevance). Off J Eur Communities. (2016) L84:1–208

14 ORDINANZA 9 agosto 2012 Misure straordinarie di polizia veterinaria in materia di tubercolosi, brucellosi bovina e bufalina, brucellosi ovi-caprina, leucosi, nelle regioni Calabria, Campania, Puglia e Sicilia. (12A09729) (GU Serie Generale n.212 del 11-09-2012) https://www.gazzettaufficiale.it/eli/id/2012/09/11/12A09729/sg (visitato il 25/08/2023)

15 ORDINANZA 28 maggio 2015, Misure straordinarie di polizia veterinaria in materia di tubercolosi, brucellosi bovina e bufalina, brucellosi ovi-caprina, leucosi bovina enzootica. (15A04879) (GU n.144 del 24-6-2015) and following amendments and additions

16 2003/24/EC: Commission Decision of 30 December 2002 concerning the development of an integrated computerised veterinary system (notified under document number C(2002) 5496)

17 EU, 2019. Commission Implementing Regulation (EU) 2019/1715 of 30 September 2019 laying down rules for the functioning of the information management system for official controls and its system components (the IMSOC Regulation) OJ L 261, 14.10.2019

18 Mardones, F., zu Donha, H., Thunes, C., Velez, V., & Carpenter, T. (2013). The value of animal movement tracing: A case study simulating the spread and control of foot-and-mouth disease in California. Preventive Veterinary Medicine, 110(2), 133-138. https://doi.org/10.1016/j.prevetmed.2012.11.024

19 Judite Monteiro Braga et al. Investigation and contact tracing of the first cases of COVID-19 in Mozambique, 2020. Pan African Medical Journal. 2022;41:302. https://doi.org/10.11604/pamj.2022.41.302.26979

20 Kölzsch, A., Davidson, S.C., Gauggel, D. et al. MoveApps: a serverless no-code analysis platform for animal tracking data. Mov Ecol 10, 30 (2022). https://doi.org/10.1186/s40462-022-00327-4

21 Bingham, P., Wada, M., van Andel, M., McFadden, A., Sanson, R., & Stevenson, M. (2020). Real-Time Standard Analysis of Disease Investigation (SADI)—A Toolbox Approach to Inform Disease Outbreak Response. Frontiers in Veterinary Science, 7, 563140. https://doi.org/10.3389/fvets.2020.563140

22 Hang, C.-N.; Tsai, Y.-Z.; Yu, P.-D.; Chen, J.; Tan, C.-W. Privacy-Enhancing Digital Contact Tracing with Machine Learning for Pandemic Response: A Comprehensive Review. Big Data Cogn. Comput. 2023, 7, 108. https://doi.org/10.3390/bdcc7020108

23 Anglemyer A, Moore TH, Parker L, Chambers T, Grady A, Chiu K, Parry M, Wilczynska M, Flemyng E, Bero L. Digital contact tracing technologies in epidemics: a rapid review. Cochrane Database Syst Rev. 2020 Aug 18;8(8):CD013699. doi: 10.1002/14651858.CD013699. PMID: 33502000; PMCID: PMC8241885.
24 Savini, L., Candeloro, L., Perticara, S., & Conte, A. (2019). EpiExploreR: A Shiny Web Application for the Analysis of Animal Disease Data. Microorganisms, 7.SIMAN. Available online: https://www.vetinfo.sanita.it/j6_siman/.
25 Sistema Informativo Veterinario. Available online: https://www.vetinfo.sanita.it/sso_portale/accesso.pl .
26 Decreto 30 maggio 2017. Istituzione di un Centro di referenza nazionale nel settore veterinario. G.U. n 196 del 26 Agosto, 2017
27 Di Lorenzo A, Mangone I, Colangeli P, Cioci D, Curini V, Vincifori G, Mercante MT, Di Pasquale A, Iannetti S. One health system supporting surveillance during COVID-19 epidemic in Abruzzo region, southern Italy. One Health. 2023 Jun;16:100471. https://doi.org/10.1016/j.onehlt.2022.100471. Epub 2022 Dec 7. PMID: 36507072; PMCID: PMC9726647.
28 Diestel, R. 2010. Graph theory. Graduate Texts in Mathematics, Vol. 173, Fourth Edition, Springer-Verlag,866 Heidelberg.
29 Blonder, B., Wey, T.W., Dornhaus, A., James, R. and Sih, A. (2012), Temporal dynamics and network analysis. Methods in Ecology and Evolution, 3: 958-972. https://doi.org/10.1111/j.2041-210X.2012.00236.x
30 Chan J, Holmes A, Rabadan R. Network analysis of global influenza spread. PLoS Comput Biol. 2010 Nov 18;6(11):e1001005. doi: 10.1371/journal.pcbi.1001005. PMID: 21124942; PMCID: PMC2987833
31 Chris Rorres, Maria Romano, Jennifer A. Miller, Jana M. Mossey, Tony H. Grubesic, David E. Zellner, Gary Smith, Contact tracing for the control of infectious disease epidemics: Chronic Wasting Disease in deer farms, Epidemics, Volume 23, 2018, Pages 71-75, ISSN 1755-4365. https://doi.org/10.1016/j.epidem.2017.12.006.
32 Natale F, Savini L, Giovannini A, Calistri P, Candeloro L, Fiore G. Evaluation of risk and vulnerability using a Disease Flow Centrality measure in dynamic cattle trade networks. Prev Vet Med. 2011 Feb 1;98(2-3):111-8. doi: 10.1016/j.prevetmed.2010.11.013. Epub 2010 Dec 15. PMID: 21159393.
33 Calistri P, Conte A, Natale F, Possenti L, Savini L, Danzetta ML, Iannetti S, Giovannini A. Systems for prevention and control of epidemic emergencies. Vet Ital. 2013 Jul-Sep;49(3):255-61. doi: 10.12834/VetIt.1206.06. http://www.izs.it/vet_italiana/pdf/VetIt_1206_06.pdf
34 Iannetti S, Savini L, Palma D, Calistri P, Natale F, Di Lorenzo A, Cerella A, Giovannini A. An integrated web system to support veterinary activities in Italy for the management of information in epidemic emergencies. Prev Vet Med. 2014 Mar 1;113(4):407-16. doi: 10.1016/j.prevetmed.2014.01.015. Epub 2014 Jan 22. PMID: 24485707.
35 Di Lorenzo A., Savini L., Candeloro L., Tora S., Cerella A., Di Sabatino D., Conte A. "Web-GIS and livestock trace tools for epidemiological surveillance, control and management" Front. Vet. Sci. doi: 10.3389/conf.fvets.2019.05.0004
36 Tegegne, H. A., Bogaardt, C., Collineau, L., Cazeau, G., Lailler, R., Reinhardt, J., Freeth, F. T., Taylor, E., Prada, J. M., & Hénaux, V. (2023). OH-EpiCap: A semi-quantitative tool for the evaluation of One Health epidemiological surveillance capacities and capabilities. Frontiers in Public Health, 11, 1053986. https://doi.org/10.3389/fpubh.2023.1053986
37 Jonathan A. Polonsky, Amrish Baidjoe, Zhian N. Kamvar, Anne Cori, Kara Durski, W. John Edmunds, Rosalind M. Eggo, Sebastian Funk, Laurent Kaiser, Patrick Keating, Olivier le Polain de Waroux, Michael Marks, Paula Moraga, Oliver Morgan, Pierre Nouvellet, Ruwan Ratnayake, Chrissy H. Roberts, Jimmy Whitworth and Thibaut Jombart. Outbreak analytics: a developing data science for informing the response to emerging pathogens. Philosophical Transactions of the Royal Society B: Biological Sciences, Volume 374, Issue 1776. https://doi.org/10.1098/rstb.2018.0276
38 Moriyón, I., Blasco, J. M., Letesson, J. J., De Massis, F., & Moreno, E. Brucellosis and One Health: Inherited and Future Challenges. Microorganisms, 11(8), 2070. https://doi.org/10.3390/microorganisms11082070
39 Charlier, J., Barkema, H. W., Becher, P., De Benedictis, P., Hansson, I., Hennig-Pauka, I., La Ragione, R., Larsen, L. E., Madoroba, E., Maes, D., Marín, C. M., Mutinelli, F., Nisbet, A. J., Podgórska, K., Vercruysse, J., Vitale, F., Williams, D. J. L., & Zadoks, R. N. (2022). Disease control tools to secure animal and public health in a densely populated world. The Lancet Planetary Health, 6(10), e812-e824. https://doi.org/10.1016/S2542-5196(22)00147-4

Response to reviewers' comments

In blue the authors' answers to reviewers' comments

**Reviewer #1:** Dear authors,

Thank you for addressing the concerns and observations I made in the initial review of this paper. In my opinion, you have greatly improved the manuscript. I reiterate on my past comment that this work isn't unique to Italy. Many other countries build their own tracing tools. However, this is usually done by IT professionals under contract and rarely gets shared to the wider scientific community which is why I'm pleased you have gone to the effort to seek to publish your work. As this is not a conventional research paper, it doesn't quite fit the template for presenting your methodologies and results. You have improved the structure and layout in this version of the manuscript and I've added some more minor edit suggestions below:

Thank you for your positive feedback and for acknowledging the improvements made to the manuscript. We appreciate your recognition of the importance of sharing our work with the wider scientific community. We have carefully reviewed and incorporated all the additional edit suggestions you provided.

Line 20: delete 'Therefore' Answer: Done
Line 21: replace "and almost in real-time" with "in near real-time" Answer: Done
Line 35: delete "emergency" Answer: Done
Line 45,46. Delete "On the other hand". Answer: Done
Line 212: Replace "In the end, such kind of strategies" with "Ultimately, such strategies" Answer: Done
Line 213: Delete "and" from "(and thus) Answer: Done
Line 233: Add "The"… "The T-Racing interface" Answer: Done
Line 234: "graphs, maps," Answer: Done
Section 2.3 is poorly laid out in terms of flow of information. I would suggest a adopting clearer headings and subheadings rather than multiple descriptions followed by bullet points.
Answer: Thank you for your valuable feedback we have revised this section to improve the flow of information and make it clearer for the reader. Specifically, we have reorganized the content into distinct subheadings to better structure the information and avoid lengthy descriptions followed by bullet points. The revised section now includes three subheadings:

- 2.3.1 Tools for extracting and viewing animal trade data
- 2.3.2 Tools for customizing network visualization and optimization
- 2.3.3 Tools for epidemiological information integration and results printing

Each subheading clearly groups related functionalities and describes them in a concise and organized manner. We believe these changes address your concerns and significantly enhance the readability and coherence of the section.
We hope that the revised structure meets your expectations.
Line 255: Replace "make" with "select" Answer: Done
Section 2.6 Demo and 3. Results.
https://demo.izs.it/app_direct/tracing/
I can access the interface but can't select any dummy data.
Answer: We apologize for the inconvenience. It appears that other reviewers did not encounter this issue. However, if you're interested, please feel free to contact us directly, and we'll be happy to assist you in resolving the problem.
Line 503: "inform the response in near real-time and to allow" Answer: Done

Line 427: Replace “focus” with “focused” Answer: Done
Line 533: I would suggest changing “Complementarily,” to “Coupling”.
Answer: Thank you for your suggestion to replace “Complementarily” with “Coupling” in line 533. After careful consideration, we chose to retain “Complementarily” because it more accurately conveys our intention to highlight how molecular epidemiological data enhances and completes the analysis, rather than simply combining elements. To better express this concept, we have rephrased the text as follows: “The integration of molecular epidemiological data into the analysis complements the validation of identified disease (brucellosis) transmission paths, exemplifying a One Health approach by a multidisciplinary team to advance disease control technology.” (in revised lines 540-543).
Lines 533 – 550. This is mostly repetition and overly wordy. Is it necessary or could it be replaced with a more simple and humble sentence?
Answer: Thank you for your valuable feedback. The paragraph in question has been revised and streamlined to address your concerns regarding repetition and wordiness. We have simplified the language while preserving the essential points of the original content (in revised lines 540-551).
**Reviewer #3:** Dear authors,
Thank you for providing an improved version of the manuscript.
I provide my detailed comments in the reviwed manuscript attached.

Thank you for your detailed feedback and suggestions. We have addressed all your comments directly in the revised manuscript. Our responses are provided in the comment sections under each request.

I have several general suggestions:
- you should clearly mention that the tool is not open source and freely available and say why
Answer: We have explicitly mentioned in the revised manuscript that T-Racing is not open-source or freely available due to the sensitive nature of the data it handles, which comes from Italian national information systems. This clarification has been added to ensure transparency regarding the tool's accessibility. Regarding why the tool is not yet open-source, it's because all the developed functionalities, from data visualization to the various integrated analyses and services, are tightly linked and tailored to the specific realities of the Italian animal trade system. This means that additional effort and time would be required to decouple these functionalities and make the tool suitable for sharing as an open-source solution.
- you dont discuss the limitations of the tool - this should be clearly addressed in the discussion
Answer: The limitations of T-Racing were previously discussed in the manuscript; however, we have now made these points more explicit in the discussion section. We have clearly addressed the tool’s current limitations, including its specificity to the Italian context and the restriction of genetic data to the Brucella genus. These updates aim to ensure that the limitations are fully transparent to the reader.
-if the tool is generic should be underlined as an advantage and say how it can be of use one day for other diseases and other services in Italy, for eample One health, as you mentuon this in the discussion
Answer: The manuscript has been updated to emphasize that T-Racing is designed to be a versatile tool. While it is currently applied within the Italian context, it already manages data for around 100 different diseases, including zoonotic diseases such as brucellosis, tuberculosis, leptospirosis, rabies, salmonellosis, campylobacteriosis, listeriosis etc. Its structure allows for potential expansion to include additional data and services, particularly those relevant to One Health. This adaptability is now highlighted as a key advantage in the discussion, demonstrating the tool’s potential for broader applications in the future.

Kind regards

**Reviewer #5:** The subject of the article is of interest.
I have revisions to suggest to authors and some questions, see below:

Thank you for your feedback and for finding the subject of the article interesting. We appreciate your comments and have addressed them in detail below in response to your questions.

Introduction section

Line 47: specify that 2.1 million people is worldwide (I suppose it is that).

Answer: Thank you for your suggestion. The phrase states that the 2.1 million people is globally affected, which has the same meaning as worldwide, that was not used to avoid repeat the same word in sequential phrases.

Line 54-60: Is it really relevant to have this description of TRACES-NT because this databasis is not used in the article? If it is maintained, it should be specified that it is only accessible to competent authority and considered as sensitive data.

Answer: Thank you for your observation. We included a description of TRACES-NT in the introduction to emphasize the importance of tracing systems in epidemic contexts and the relative collection of trade data. Although TRACES-NT is not used by T-Racing, it serves as an example of a relevant database for illustrating the broader landscape of traceability in disease control. We acknowledge that TRACES-NT is not freely accessible and is only available to competent authorities and in the revised manuscript, we have clarified this by adding the statement "The system is only accessible to competent authorities and is considered sensitive data" in revised line 59, as suggested. Moreover, we highlighted in the revised manuscript that T-Racing relies on Italian national databases for its operations. Thank you for pointing out the need for this clarification.

Material and method

• I was surprised that no wildlife data (wildlife TB and brucellosis positive cases) were considered for TB and brucellosis. Are these data not available? Is it a possibility to improve the tool for future? It would have also justified the “one health” approach cited in the article.

Answer: We appreciate your question and suggestions. We would like to clarify that data on wildlife regarding brucellosis and tuberculosis are not available at the moment and no surveillance is systematically conducted in wildlife in Italy regarding these diseases that were used here just as an example of the functionality of the application. We understand your point of view regarding “One Health” and We have reduced its usage throughout the manuscript and replaced it with "multidisciplinary approach" where appropriate, however, we respectfully would like to clarify, that the approach considered in the paper for “One Health” has to do with the prevention of zoonosis by tracing the paths of positive animals, avoiding dissemination. To perform the construction of the application and the interpretations of its results, a multidisciplinary team was necessary. The original concept of “One Health” proposes that multidisciplinary teams work together to face the challenges to control diseases and decrease the burden imposed by them, and our application was the result of this approach. Regarding your specific concern on the importance of wildlife data, it is important to note that outbreaks managed in T-Racing (provided by SIMAN system) can include not only farm-based cases but also positive cases in wildlife. These positive cases can be used as seeds and buffers to include the commercial network, as explained in the manuscript. Wild boar data for ASF (African Swine Fever) have already been integrated into the system as cases (outbreaks) and serve as an example of how T-Racing manages wildlife data. Thank you for raising this important issue.

• How rendering plants and slaughterhouses are considered in the tool, are they considered in the same way as a farm in the network analysis? Idem for transit section that are cited in the result section (line 381) but we don’t have information on how it is considered in the tool in the material and method section.

Answer: Thank you for your query. In T-Racing, rendering plants and slaughterhouses are treated as distinct attributes within the network analysis. These facilities are represented as nodes within the network, with specific shapes and labels used to identify and highlight their roles. This approach ensures that these structures, which are integral to the animal trade system and of veterinary interest, are clearly distinguished in the analysis. Similarly, transit stations are also considered in the tool. They are included as part of the network analysis and are marked accordingly to reflect their function and relevance in tracing disease outbreaks. This inclusion helps to identify and analyze patterns of disease spread within the entire animal trade network (as stated in lines: 165-168 and 289-293 of the revised manuscript).

• Line 242-243: the tool considere spatial buffer using distance by kilometers. Do you think of using pasture data to improve your tool in the future?
Answer: Thank you for your question. We would like to clarify that nodes described as pasture were used in the dataset as part of the network data. Pastures are represented as points, and you can apply a buffer to account for the spread to neighbouring holdings.

• Line 330-331 and 340: how these parameters were determined? Taking into account movements only over the past 13 months seems for instance restrictive considering TB, a chronic infection that can be difficult to detect. Was it based on experts' elicitation?
Answer: Thank you for your question. The considered case study and parameters, such as the time interval and depth levels, are intended to illustrate the application of T-Racing rather than to serve as definitive measures for all analyses. In this example, the parameters were chosen based on the data available and were set iteratively to best highlight secondary outbreaks. The time interval of 13 months and depth levels were adjusted according to the specific context of the study, informed by the nature of the disease and the data constraints. These parameters are not fixed but can be tailored by users based on their expertise and the specific requirements of their investigations. For chronic infections like tuberculosis, which can be difficult to detect and may have longer incubation periods, users can adjust these parameters to extend the analysis period and depth as needed for more comprehensive investigations.

• Line 350: considering TB as important zoonosis seems not adequate. It has important economic impact due to movement and commercial restrictions for farmers but in Europe TB human cases are not anymore due to bovine tuberculosis as far as I know. Is there any publication that can support this zoonotic aspect in European countries? It seems to me that it is not a big issue for human health anymore.
Answer: Thank you for your consideration. In Europe TB is indeed not important as zoonosis, however in other countries, the reality is quite different. T-Racing was developed specifically for Italy, but its design allows for adaptability and potential use in other countries. We propose this application as a solution for other official veterinary services globally, which is why TB was included as an important zoonosis. To clarify, we have revised the text to state: "Brucellosis caused by Brucella abortus is an important zoonosis worldwide, and tuberculosis caused by Mycobacterium bovis constitutes a serious hazard to public health in developing countries and a great economic burden to livestock production in Europe. These diseases were chosen due to their public health and economic importance and their chronic development, allowing for the use of FCT and BCT methods with a broad time-elapse." Additionally, we highlighted in the discussion session that T-Racing will become open source and will be capable of incorporating proprietary data.

Results

• Line 471: it seems here that pasture data are included but I can't find this information in the material and method section.
Answer: Thank you for your comment. To comply with your comment we add pasture to the holding type in the methods section. Line: 134 to 136: "…such as the holding type (e.g., farm, slaughterhouse, staging point, transit station, market, assembly centre, foreign country, pasture, etc.),…"

Discussion

Several times in the article, and especially in the discussion section, the “One health” approach is cited but I can’t see how the tool could be considered as a One health approach tool at that stage.

There is non-human health data which could be relevant only for brucellosis. For BT it is relevant to consider only human cases due to bovine tuberculosis due to contamination in Italy which I suppose is very rare, nearly no case no? If data on human cases due to bovine tuberculosis and brucellosis are available it would be great to add it in the introduction section. If BT is of importance on a zoonotic aspect, authors should consider to improve the tool in the future, to add information on farm production type so as to distinguish raw milk production farms.

At that stage there is no data on wildlife surveillance considered which could have justified the one health approach with the environmental sector.

Answer: Thank you for your comment. We have reduced its usage throughout the manuscript and replaced it with "multidisciplinary approach" where appropriate

It is important to note that the majority of zoonotic diseases are controlled in humans by managing them in animals. The application presented in the paper serves as a critical surveillance tool for tracking animal diseases, which is a key component of the One Health approach. Effective tracing allows official veterinary services to intervene and break the chain of disease transmission, which is crucial for controlling and preventing the spread of diseases. For example, interventions such as the slaughter of infected animals and increasing public awareness about the risks associated with raw milk can help prevent the transmission of diseases to humans. In this sense, the application supports the One Health approach by contributing significantly to disease control in animals, which in turn protects human health. Additionally, the tool was developed by a multidisciplinary team with the goal of preventing diseases, which aligns with the One Health perspective. It is important to note that the application’s objective - focused on preventing disease spread and protecting both animal and human health- aligns with the One Health approach, rather than the specific data used. As previously highlighted in responses to reviewers, achieving a One Health state requires coordinated efforts across animal, environmental, and human health. This does not necessarily mean addressing all aspects simultaneously but involves steps to prevent disease spread from animals to humans. This focus on safeguarding animal health to protect human health is central to the One Health approach.

First, we would like to clarify that the diseases used in the paper were chosen as examples of how the application works. Indeed, these diseases have different importance considering the zoonotic aspect in Europe, however, the data used here (as an example) was from 2022 and 2023 about tuberculosis identified in bovines and this is a concern for human health because the zoonotic potential of the disease exists. Therefore, rigorous surveillance should be applied when cases occur in bovines, to prevent the disease to spread, preventing transmission to humans. Regarding the production type of the farms, it is presented in the data as milk and beef production, and all milk produced is raw milk and it is transported to the dairy industry to be distributed to the population after pasteurization.

Even though wildlife data are limited, the One Health approach is justified by other points, such as using a technological tool, that contributes to disease control and benefits different professionals such as clinicians, veterinarians, ecosystem health professionals, researchers, and policymakers to leverage the power of animal surveillance. As previously mentioned, the system includes all reported cases of various animal diseases (both zoonotic and non-zoonotic), which can also affect wildlife, as seen with diseases like African Swine Fever. We have made efforts to better describe the outbreak data and diseases contained in the database in the paper.
