# Supplementary material for: T-Racing: a modern tool for supporting epidemiological investigation in animal disease outbreaks in Italy
Source: PLoS One. 2025 Feb 19;20(2):e0317313. doi: 10.1371/journal.pone.0317313 (PMC11838889; doi:10.1371/journal.pone.0317313)
Supplement: Attachment — Submitted filename: Answers to Reviewers.docx [file pone.0317313.s002.docx]

**Responses to Reviewers**

We are grateful to the reviewers for their detailed review and for acknowledging the main purpose of describing the T-Racing application in a scientific article. T-Racing is the result of extensive efforts by a multidisciplinary team that collaborated throughout all stages of development— from data analysis to creating the tools and developing a user-friendly interface. This process involves analyzing large and diverse datasets that, on their own, cannot provide useful information. These data continually evolve and as new or edited information and attributes are provided T-Racing is consequently adjusted. These data are harmonized and made usable within an application that allows for iterative analysis and visualization in a user-friendly manner. The scalability of the application permits easily integration of future datasets and *ad hoc* tools.

The One Health approach is relevant to this work because most zoonotic diseases are transmitted by animals. Effective control of these animals can help break or contain the transmission chain to humans. Future development on tracing will include the incorporation of additional information, such as expanding genomic data on sampled animals or even adding human cases and other relevant data as soon as they become available.

We acknowledge the complexity of describing such work in a paper and appreciate the reviewers' comments. While these comments have required significant revisions, they have provided the opportunity to prepare a more complete and significantly improved version of the manuscript.

Below, we have provided punctual responses to the requests and comments from each of the reviewers (our responses are highlighted in red).

**Specific Responses to Reviewers**

1. The reviewers requested providing a working instance of the tool (online or as a stand-alone application to be downloaded): This concern has been addressed by providing a demo version of the application. The development and dataset to the demo were described in a dedicated section in the Materials and Methods, as well as the link to the demo is provided at the beginning of the Results section.
2. The reviewers suggested restructuring the organization and narrative of the manuscript: We have thoroughly restructured the manuscript by creating new paragraphs and sub-sections in the Materials and Methods, redistributing the text as suggested by the reviewers and editor, and completely rewriting the Discussion section. Additionally, all redundancies present in the previous manuscript version have been eliminated.

The reviewers requested higher quality figures: We have recreated all the figures in higher resolution. We used the demo version of T-Racing to redo the case studies and the related figures.

**Reviewer 1**

The insertion of the case studies is essential for describing the tool but the subject matters, BTB and Brucellosis, aren’t be properly described in terms of their aetiology, natural history, etc.; nor can they be. I’m not sure how to address this. Possibly a single paragraph on each disease as part of the case study introduction? Thank you for the suggestion. We have added a specific paragraph in the Materials and Methods section (subparagraph 2.5) and have removed all non-pertinent information from the Results section (subparagraphs 3.1.1 and 3.1.2). This information has been redistributed to the Materials and Methods or Discussion sections as appropriate.

It is inferred in the text, but I think there should be more a more explicit description of how the user’s expert elicitation is required to drive the tool. The tools helps guide the user rather than offering an instant solution. That is a good consideration. We have integrated this concept at the end of the Abstract, Introduction, and Discussion sections.

I assume the tool is a work-in-progress? This should also be addressed. Are their future plans for improvements/enhancements? Can the tool be leveraged to provide researchers with data on animal contacts of disease modelling, etc? These would make useful discussion points. As clarified in the revised manuscript (at the end of section 2.3, T-Racing Design), the tool is “…available on the internal network of IZS-Teramo and is awaiting some technical adjustments and official approval from the Italian Ministry of Health before it can be integrated into the VETINFO portal. This integration will ensure secure access and the protection of sensitive data, as T-Racing inherits the robust GDPR techniques already applied in the portal.” Moreover, the system is scalable and can be easily modified to adapt to the dynamic nature of available data/new data and the needs of Veterinary Services in addressing emergencies. All improvements will be based on near-real-time data from Italian information systems and tools to enhance the control and containment capabilities of Veterinary Services and decision-makers. These future improvements have been discussed and integrated into the Discussion section. Currently, there are no plans to include epidemic spread prediction.

Brucella spread usually has a strong temporal component. The lead up to the calving season is the highest risk time for spread of infection. Could a temporal window be applied to tracings to add a risk weighting to cohorts of animals residing together at these times? This could help narrow down the number of nodes.
Currently, regarding the seed farm, outgoing movements can be limited to the time period right after the parturition (this is to focus on the movements with the highest risk of transmission). We assume that the user has access to the birth dates. We are also looking into adding more data, such as the birth dates of calves, in future versions of the application.

The Brucella case study cited genetic sequencing as an additional layer to confirming tracings. Has this been done with BTB in this study? In this study, integrating genetic sequencing with the bovine tuberculosis (BTB) case study was not feasible. Presently, the genetic information pertinent to BTB is not available in GenPat. However, efforts are underway to incorporate this data into the database, which holds promise for future investigations with T-Racing, which is automatically ready to handle and visualize such data.

The stated sensitivity of the skin test is at an animal level. See text in manuscript comments. we answered this in the manuscript comments.

The figures are quite small and pixelated. Perhaps this is just in the review version (pdf)? We have redone all the figures in higher resolution.

**The following comments relate to editorial changes within the manuscript:**

**Abstarct**

Line 10: “and lets” suggest change to “helping”: Done

Line 11: “to safeguard” suggest change to “of safeguarding”: Done

Line 13: “multiple simultaneously and” change to “multiple sources simultaneously at” Done

Line 14: delete “sources” Done

Line 14: “has been” change to “is” Done

Line 18: Delete “some” Done

**Introduction**

Line 27: “reduce” to “reduces” Done

Line 8, 33, 311,312, 320, etc. One Health or One-health or One-Health?! Stick with one! Done

Line 36 “data is” change to “data are” Done

Lines 48. 52. 57. 60. 63. etc. New paragraphs created unnecessarily. Remove paragraph breaks and associated indentation where not required. Done

Line 55. “require to rapidly” change to “require to be able to rapidly” Done

Line 63. “of disease spreading” suggest change to “within the spread of disease” Done

Line 69. “who” change to “that” Done

Line 70. “virus” change to “disease” Done

Line 72. “This could be an invaluable tool that allows” change to “This would be an invaluable tool allowing” Done
Line 76. “interacting” change to “interactive” Done

**M&M**

Line 80. “accounting for the” change to “utilizing” Done

Line 82. Delete “Along with” and don”t start a new paragraph. Done

Line 102. “(in Table 1 are listed all of the packages used)” change to “(Table 1 contains a list of all packages used) Done

Line 102 - 103 “The application lets manage large amounts of data from different information systems and share them efficiently and securely in real-time.” Change to “103 “ The application manages large amounts of data from different information systems and shares them efficiently and securely in real-time”. Done
Line 105. Delete “Indeed” Done

Line 105. Suggest change “differently” to “separate”: We have rephrased the sentence in “Differently from the serverless JavaScript web apps, computationally heavy services are executed on a remote server, and the Plumber package allows the application to query data sources in real-time, through web API.”

Line 106. Suggest change “let” to “allows” Done

Line 108. Change “makes sure the” to “facilitates” Done

Line 125. Change “out-breaks” to “outbreaks” Done

Line 127. Change “outbreaks” to “outbreak” Done

**Results**

Line 172. What is this? A law? “Through the DM 27 August 1994 n. 651 entered into force in Italy” yes, it is a low, we rephrased in "Through the Ministerial Decree of 27 August 1994 n. 651 …"

Line 176. “provides for control official serological test” change to “demands serological testing” Done

Line 259. “near” suggest change to “nearby” Done

Line 262. “let to establish” replace with “establishing” Done

Line 264. “(three of which, were outbreaks)”. I’m a bit confused by this. Do you mean 3 out of the 4 farms had confirmed outbreaks? We apologize for the confusion. We have reanalyzed the data, and in the meantime, the outbreak data has been updated in the database, so it has changed slightly. The description of the case study has been rewritten, and we believe it is now clearer.

Line 289. Is the whole herd tested at the same time? This has the effect of increasing the sensitivity of the test as it is effectively not a single animal test if the whole herd is restricted if a positive animal is found. This is an important consideration. According to EU rules, all animals aged more than 12 months are subject to testing, and they are tested once a year in the same time, except for large herds where more days can be needed, however, in any case, they must be consecutive days and the testing must be completed in the shortest time period possible, in any case no more than a week. Data about sensitivity are related to this testing regime, which is the same employed in the rest of EU countries (as described by De la Rua-Domenech et al., 2006)

**Discussion**
Line 310. “and outbreak management” Does this refer to the field staff? Maybe rewrite to clarify. Done
Line 317. “racing” suggest change to “racing against” Done

Line 325. “always explored” change to “always be explored” Done

Line 333. “So having data about traded animals” change to “Thus, having data on traded animals” Done
Line 353. “highlighted” change to “highlights” Done

**Reviewer 2**

**Main comments**

The manuscript does not provide sufficient information about the data and the methods. There is only an inventory of the sources of data and one sentence on the methodology. More details are needed to understand how the application manage and analyse the data to obtain outputs. Thank you for the suggestion. We have provided a thorough description of the data and methods in the Materials and Methods section, with dedicated sub-sections (2.1 Data sources and collection and 2.2 Temporal Network Analysis methods in T-Racing).

Regarding data, the authors should also discuss how the application complies with the General Data Protection Regulation (GDPR), given that various types of users are identified and will have access outputs (movements tracing of individual animals, genomic information) that are sensitive**.** This issue has been clarified in the text by adding the following sentences in M&M section (to the end of subparagraph 2.3 T-Racing Design): “Currently, it is available on the internal network of IZS-Teramo and is awaiting some technical adjustments and official approval from the Italian Ministry of Health before it can be integrated into the VETINFO portal. This integration will ensure secure access and the protection of sensitive data, as T-Racing inherits the robust GDPR techniques already applied in the portal”

The authors mentioned that the T-Racing application is currently in testing phase but no information is provided about this pilot study (see the specific comment below, Line 138). Besides, I suggest discussing the main results of these tests (i.e. strengths and weaknesses of the application) and provide feedback regarding the friendliness and relevance of the application, and how the results from the testing phase were used to improve the application. We have clarified in the text that T-Racing is ready to be used (see the previous response). Additionally, T-Racing application has not undergone a formal and structured testing phase but rather represents the evolution of previous applications developed by the same team with extensive usage in the Italian veterinary healthcare system. It is the outcome of collaborative work by a multidisciplinary team including epidemiologists, veterinarians, mathematicians, and computer scientists, who collectively contributed their expertise to its development and “testing phase”. This concept has been clarified at the end of subparagraph 2.3 T-Racing Design.

**Specific comments**

**Introduction**
- Line 38: TRACES has become TRACES-NT (new technology) for few years:

<https://webgate.ec.europa.eu/IMSOC/tracesnt-help/Content/en/index.html.> Done

- Line 45-60: Avoid 1-2 sentences paragraphs. All these sentences should be gathered in one paragraph about the interest of tracing disease spread for disease surveillance and control. Done

- Lines 61-63: Please expand this part about existing tools for animal tracing and explain what are the current limits of these tools or what features are missing (justifying the development and use of T-racing)? Done

- Lines 64-77: All these sentences should be gathered in one paragraph. Done

**Materials and Methods**

- Lines 85-90: Please provide a description of the data and datasets of these three databases (including spatial and temporal unit of the data and detailed information on livestock owners and producers). Done

- Line 97: Add references of previous studies using such methods for tracing disease spread. Describe how the temporal network analyses were conducted (including specifications about spatial and temporal units). We have included a subsection in the Materials and Methods section that explains this in detail: '2.2 Temporal Network Analysis methods in T-Racing.' This section provides information on how the temporal network analyses were conducted, including specifications about spatial and temporal units.

- Lines 100-109: All these sentences should be gathered in one paragraph. Done

- Line 110: Are the tools proposed in the T-Racing application available to all types of users? How does the application comply with the General Data Protection Regulation (GDPR)? Yes, the data used by T-Racing are sensitive, so only the authorized users (veterinarians, Veterinary Services, public health institutions, etc., ) can access through their credentials. This issue has been clarified in the text by adding the following sentences in the Materials and Methods section (to the end of subparagraph 2.3 T-Racing Design): “Currently, it is available on the internal network of IZS-Teramo and is awaiting some technical adjustments and official approval from the Italian Ministry of Health before it can be integrated into the VETINFO portal. This integration will ensure secure access and the protection of sensitive data, as T-Racing inherits the robust GDPR techniques already applied in the portal.”.

**Results**
- Line 138: The testing phase is an important step in the development of a tool to ensure that it meets the needs of users. What does the testing phase consist of? Please provide some details about the number and types of users (authorities, operational actors, etc.). Which indicators are used for testing the application and evaluating its performances? Please, see the previous answer above

- Line 151 (and 158): please clarify the sentence: “and the red colour highlights paths followed by animals, singularly identified”, (which?) “had gone out of the seed” This sentence has been completely rephrased as: 'Highlighting on the graph are all links involving the same animals that reached the seed (red links). ...' and made clearer by the detailed explanation of the T-Racing tool, which allows for highlighting within the network all edges involving animals moved from/to the seed based on the tracing strategy applied (see Materials and Methods section in 2.2 Temporal Network Analysis methods in T-Racing and 2.3 T-Racing Design sub-sections).

- Line 161: These examples seem to be based on real (not simulated) data. If so, replace “simulation Exercise” by “Case studies”. If simulated data were used, please describe this step (methods, data, etc.) in the Materials and Method part. Thank you for the observation. Yes, it is correct that the data used are real, so we have replaced "simulation Exercise" by "Case studies” as suggested.

- Line 172 : Does DM mean Ministerial Decree ? If so, specify it on line 171 and use DM at each occurrence (e.g. line178). Otherwise, replace by “Ministerial Decree”. Done

- Line 187: In this case study on bovine brucellosis, first you used a period of 2 years for tracing movements, and then, line 198, a period of 399 days to reduce the number of movements. How did you select these values? Please also explain more broadly in the Discussion how the application should be calibrated (species selection, time window, etc.) for tracing back and tracing forward movements depending on the diseases of interest. Is the application more appropriate for specific types of diseases (like, for example, slow spreading diseases)? The questions raised by the reviewer are legitimate. In the new version of the manuscript, we have emphasized that T-Racing is an application designed to make the job easier, provide flexibility, and make use of the user's knowledge. There are no universal rules, and each investigation has its unique characteristics. As explained in the text, the choice of depth (of levels and days) depends on various factors related to the specific disease being considered, such as contact, incubation and recovery rates, and the frequency of health checks carried out on the farms. Users may find additional information on other systems or make a choice based on their knowledge of the disease. Indeed, the example provided is just one possible use case and does not constitute a legal report signed by tracing officers.

- Line 198: What are those three levels? The levels refer to degrees of separation. We have explained the methods of level and day depth to calibrate the network of movements in the Materials and Methods section, specifically in subsection 2.2 Temporal Network Analysis methods in T-Racing.

- Line 204-205: “The reduced network, focusing on outbreaks within the network, “ Please, clarify what these two networks refer to? These are not two distinct networks, but one (the reduced network) is a subset of the other. The sentence has been rephrased to: 'The reduced network, created by keeping only temporally valid paths among involved outbreaks within the network.' The details of this network reduction method are explained in the Materials and Methods section.

- Line L209 : Write brucela abortus in italics. Done

- Line 214: The application enables search on “individually identified animals”. Such outputs are certainly sensitive information and I am expecting that this will be a barrier to the use of the tool. Can you please explain how the application deals with this issue? Authorized users with access to T-Racing have no restrictions or limitations. Please refer to the previous answer.

- Figures 3, 4, 5 and 7 are too small to be readable. Done

- Line 254 : “examples” Done

- Figure 6: What does the black link mean? in the figure, the black link represents the movement of animals that do not come from seeds, unlike the red links.

- Line 278: What tool do you refer to in the sentence “we employed a tool” We rephrased the sentence to: 'For a more focused investigation, we considered only temporally valid paths connecting outbreaks, which resulted in a subnetwork of…' The tool referred to is the network reduction method, which is explained in the Materials and Methods section.

**Discussion**
- Line 305-307: This sentence is exactly the same as the first one of the Introduction! The Discussion session has been completely rewritten, and all redundancies present in the previous manuscript version have been eliminated.

- Line 312-322: This is not Discussion. I suggest moving this paragraph to the Introduction to underline the interest of One Health in disease surveillance, and highlight how tracing disease spread is critical for effective disease control. Done

- Figure 8: This figure would be better positioned in the Results part to demonstrate the relevance of T-Racing in preventing and detecting diffusion pathways versus the simple farm isolation, using a retrospective analysis of data. In Figure 8, please describe more specifically the detection and prevention of cases when T-racing is used versus without T-Racing (=surveillance program only). Letters of the alphabet could be used to distinguish the four farms and explain the chain of events regarding the disease transmission between farms (disease introduction and spread) and detection (through surveillance program and using T-Racing). Specify what is the “surveillance programme”: programmed surveillance (serology twice a year) or abortion notification? What do the yellow arrows mean? Besides, I suggest using a darker colour than yellow to be more visible on the figure. Thank you for the valuable suggestion. We have included this part in a dedicated subparagraph (2.2 Temporal Network Analysis methods in T-Racing) in the Materials and Methods section. We have modified the figure according to your indications. Regarding what we mean by 'surveillance program,' this has been clarified in the dedicated subparagraph '2.5 Brucellosis and Tuberculosis: Rationale for Case Studies and Italian Regulations.

- Lines 333-337: These sentences (at least the main idea) should be in the Introduction to explain the interest of developing and using the T-Racing tool versus getting an extraction of the movement network through a query of the BDN (National Database for Animal Registration)? Thank you for the suggestion. We have moved this information to the Introduction and in the Discussion.

- L356-357: How is this sentence related to the topic of the paper? How the tool can foster collaboration between sectors? In the updated version of the paper, it should be clearer the practical utility of the application within the One Health framework for all stakeholders involved. We described how the application streamlines the work of epidemiologists, leading to prompt and more efficacious interventions by veterinary services. This, in turn, enables the containment of disease spread in animals and diminishes the risk of zoonotic transmission to humans.

- Line 367: What do you mean by “reliable data”? What criteria should be taken into account to consider that data is reliable? Thanks Thank you for the question. In our context, 'reliable data' means accurate, consistent, and timely information that is free from significant errors or biases. It's crucial to have a genuine and precise representation of a phenomenon, even if it's not entirely exhaustive, rather than relying on erroneous data that could lead to incorrect interpretations of the results. We achieve this by linking T-Racing to established and continuously updated national databases. Despite the occasional incompleteness, such as delays in timely data updates (e.g., for bovine movements, which may be almost a week behind), these databases offer verified and regularly maintained data.

**Additional comments**

- Can the tool be used to trace emerging diseases (so-called Disease X)? Yes, the tool can be used to trace emerging diseases (so-called Disease X) if the data for Disease X are entered into the databases linked to T-Racing.

- Can the tool be used by other countries with similar data and if so, how would the tool be made available? Currently, it's challenging to imagine other countries using the application, but it could be possible in the future. The main issue is that the application's data structure is tailored specifically for use in Italy. It's a complex and diverse structure, and the effort to standardize it was done only for Italian data. Another concern is the architecture of the application: it's not a simple "shiny" app, but rather requires databases and REST API services, necessitating the establishment of an IT infrastructure to support its operation. Because of this, we mentioned in the section about future developments that we will be working on a more universal version of the application. This version will have its source code available on GitHub and will also support the import of user’s data.

**Reviewer 3**

**Introduction**: It’s partially vague. As a veterinarian and epidemiologist, I understand the problem, but what is state-of-the-art, and why is your tool unique? What motivated you to create this tool? Please also remove redundancy in several phrases. Be more explicit about the objectives of the work. Thank you for your insightful feedback. We have made significant revisions to the introduction to address your concerns. Specifically, we have added a section that discusses existing applications and methodologies used for tracing disease spread, highlighting the current state-of-the-art. Additionally, we have clearly articulated what makes T-Racing unique, including its ability to integrate diverse data sources, its user-friendly interface, and its scalability. Moreover, we have elaborated on the motivation behind creating T-Racing, emphasizing the need for a robust tool to aid in the rapid and effective control of animal disease outbreaks and its capability to guide users in epidemiological investigations by adopting a data-driven approach, via an iterative analysis while leveraging their expertise. The objectives of our work have been clearly stated to provide a comprehensive understanding of the study’s aims. Furthermore, the text has been reorganized to enhance its flow, a logical progression of ideas and eliminating redundancy.

Methods: It’s vague. The methodology (the tool) is the heart of your work; if we need to help to understand your tool clearly, how would we know its usefulness? Please restructure this part into sections and subsections. For example, the section on the tool should have an explanation of each module. The section on your experiments should provide details of the disease models and regions. Check for inspiration and organisation of the work via other methods/tools papers published in Plosone. For example: <https://journals.plos.org/plosone/article?id=10.1371/journal.pone.0199960> We appreciate the opportunity to improve the clarity and organization of our Methods and Materials section. We have restructured this section into subsections and provided detailed explanations for each component based on your suggestions.

Results: There is a mix between methods and results. Calrity and reorganisation is necessary. We have carefully reviewed the Results section and have reorganized it to improve clarity. Any content that was more appropriate for the Materials and Methods section has been relocated accordingly.

Discussion: It's an introduction and not a discussion. I dont see how the results are original compared to simillar works and tools in Europe. Their originality, limitations and perspectives. This section needs most reworking. We have completely revised the Discussion section to clearly distinguish it from the Introduction. The Discussion now focuses on interpreting our results, highlighting the original contributions of our study, discussing its limitations, and proposing future research directions.

**Other questions:**

Is the code of the R shiny app available? The application is currently based on a specific data structure used in Italy and has a complex architecture with databases and rest API services. Because of this, we haven't yet worked on making the application available for general use, as it was developed for restricted use in Italy. However, we plan to work on a more generic version of the application, and its source code will be available on GitHub. This version will also allow for the import of own data, as stated in the discussion.

How the tool was tested with end-users and co-created with end-users? As indicated in our previous response, T-Racing application has not undergone a formal and structured testing phase but rather represents the evolution of previous applications developed by the same team with extensive usage in the Italian veterinary healthcare system. It is the outcome of collaborative work by a multidisciplinary team including epidemiologists, veterinarians, mathematicians, and computer scientists, who collectively contributed their expertise to its development and “testing phase”. We have provided clarification on this in the Methods and Materials section, in subsection 2.3 titled "T-Racing Design"

Why is the tool a demo and not operational? T-Racing is ready to use and is operational for epidemiologists and veterinarians who work inside our Public Health Institute. This issue has been clarified in the text by adding the following sentence in the Materials and Methods section (to the end of subparagraph 2.3 T-Racing Design): “Currently, it is available on the internal network of IZS-Teramo and is awaiting some technical adjustments and official approval from the Italian Ministry of Health before it can be integrated into the VETINFO portal. This integration will ensure secure access and the protection of sensitive data, as T-Racing inherits the robust GDPR techniques already applied in the portal.”

The tool allows us to assess the risk based on animal movements only. What about human movements and contaminated products? T-Racing is a scalable system, and if structured data on human movements were available, we could integrate tailored tools for their representation and analysis. Similarly, regarding contaminated products, we could implement specific features in the tool to analyze and assess their risk if relevant data were accessible. Structured and updated data on human movements are not readily available, and their availability often depends on personal choices. This aspect would lead to a broader discussion. Concerning contaminated products, information on these could be incorporated, and if a national system for collecting such data becomes available in the future, our scalable system could easily accommodate it within T-Racing.

For example, how would it be for African swine fever? in the case of African swine fever (ASF), the management of disease outbreaks is handled by the SIMAN system, while T-Racing has been used to monitor pig movements, providing valuable insights into the disease spread and identifying risks for large Italian pig-producing companies. Indeed, there are ASF-infected wild boar cases available in SIMAN and by tracing with the spatial buffer tool, we can evaluate the impact of the virus introduced in nearby farms on the trade network. in the Based on this evidence, our institute's epidemiologists were engaged by the Ministry of Health in a task force to define appropriate control and containment plans.

You mention the tool is important from a public health perspective. How do you envisage using it with human data and human outbreaks of Brucellosis and Tuberculosis, for example? T-Racing is intricately linked to National Databases. If these databases were to include human cases, we could seamlessly utilize T-Racing as it currently stands to pinpoint potential disease sources or transmission between animals and humans. The tool allows for the selection of geographical proximity from outbreak sites (buffer from the outbreak). See also the following answers.

How do you envisage using the tool for movements originating outside Italy? Origin EU and outside EU, for example? Our tool, which incorporates data from the Animal Movement Database (NDB), contains information on movements to and from abroad, encompassing both EU and non-EU countries. This is reported in M&M subsection “Data source and collection”.

In the introduction and discussion, you discuss the importance of your tool for One Health; this term is overused because I would like to see more explicitly how the tool adds to the human and environmental Health presented in the methods and the results. Say it clearly if the tool is just for animal health and the perspectives are to enlarge to One Health. We acknowledge the reviewer's feedback regarding the excessive use of the word "OneHealth" in the previous version of the manuscript, and have since reduced its frequency. However, it's essential to emphasize that achieving a state of "one health" requires concerted efforts in animal, environmental, and human health. This doesn't necessarily mean employing a method that addresses all aspects simultaneously. Taking steps to prevent the spread of diseases transmitted from animals, which could potentially affect humans if left uncontrolled, involves actions aimed at safeguarding animal health for the protection of humans. This approach is central to the One Health perspective.

Reviewer 4

First, it is very poorly written, and it needs a substantial restructuring (see further comments). Second, it is hard to evaluate the tool without having access to it. I would suggest the author to resubmit the manuscript once a demo of the tool is available online (or provide it) and with a dummy dataset (i.e. made-up network/farms/outbreaks) to allow running the scripts. Thank you for your comments/suggestions. We revised deeply the manuscript and made a demo application available with anonymized datasets for public use.

Comments:
1. The &ldquo;Introduction&rdquo; section is poorly written, it is a collection of statements without a logic flow. Please rewrite. The whole manuscript has been revised.

2. Lines 94-96: please report how the tool complies with the privacy laws (GDPR, etc). This is addressed in M&M (2.3 T-Racing Design).

3. Lines 97-98: &ldquo;Temporal Network Techniques&rdquo; is too generic, please say which ones and provide references. We added a new dedicated paragraph in M&M (2.2 Temporal Network Analysis methods in T-Racing).

4. Table 1: in my understanding R packages sp and rgdal might not going to be maintained anymore, their functionalities could be found in sf and terra. I&rsquo;d suggest to investigate this issue and eventually change the code. The reviewer's observation is accurate. However, when the spatial trajectory drawing functions for T-Racing were developed, the packages were still being maintained. Because the usage of the packages is limited to a few basic spatial functions, the authors chose not to translate the code into sf (the terra package is not required by T-Racing). However, the shareable version of the application we are going to develop will utilize the new sf package.

5. Why does Fig 2 not show a point in the Marche region where the brucellosis outbreak was detected? If it is because the outbreak was traced back between Sicily and Tuscany? If this is the case, please add some indication on where the outbreak was found (Marche?), otherwise it is very confusing. We improved Fig.2 (now Fig 6) as suggested and add a red arrow on the map pointing to the seeding farm.

6. In general, figures are not consistent with the text and look randomly placed. All figures have been improved in resolution and placed accordingly to the text.

7. Paragraph 3.1.1 (and 3.1.2): the first describing general information about brucellosis (and bTB) should go into introduction or material and methods. We do agree, added a dedicated paragraph (Brucellosis and Tuberculosis: Rationale for Case Studies and Italian Regulations)

8. Lines 196-199: it is not clear how you narrowed down from 386 to 38 farms (and 1826 to 199 movements). Which type of tracing was done? Is it going to be available in T-Racing? We described in M&M the tools and the steps to achieve the results reported in figures.

9. Lines 278-280: similarly, which tool was used to connect &ldquo;outbreaks&rdquo;? With which criteria movements/farms were excluded? See above answer.
