# Supplementary material for: T-Racing: a modern tool for supporting epidemiological investigation in animal disease outbreaks in Italy
Source: PLoS One. 2025 Feb 19;20(2):e0317313. doi: 10.1371/journal.pone.0317313 (PMC11838889; doi:10.1371/journal.pone.0317313)
Supplement: Attachment — Submitted filename: Response to reviewers.docx [file pone.0317313.s006.docx]

PONE-D-24-01354R2

T-Racing: a modern tool for supporting epidemiological investigation in animal disease outbreaks in Italy

PLOS ONE

Reviewer #1: Dear authors

Thank you for updating the manuscript to account for the last round of comments!

My main issue at present is I can't access the demonstration. The link in the paper isn't currently working: [https://demo.izs.it/app_direct/tracing/](https://urlsand.esvalabs.com/?u=https%3A%2F%2Fdemo.izs.it%2Fapp_direct%2Ftracing%2F&e=14a859a8&h=c326cb4b&f=n&p=y) Could you please resolve this so I can see everything working?
Thank you for your message. We're glad to hear that you were able to access the demonstration successfully on your own.
There is still a bit of repetition in the introduction and discussion (and the start of the materials and methods section). You could remove some of this without impacting on the message you're conveying.
Answer: Thank you for your feedback regarding the repetition in the introduction, discussion, and the beginning of the materials and methods section. In response to your comments, we have carefully reviewed the text and identified overlapping content between the introduction, M&M, and discussion. We have removed certain redundant phrases and concepts without compromising the overall message we aim to convey. This has helped to enhance the flow and coherence of the manuscript. We are grateful for your guidance, which has contributed to refining our work.

Finally, some minor editorial comments:

Line 60: This has been pasted twice - remove ". t is only accessible to
61 competent authorities and is considered sensitive data."

Answer: the test has been deleted

Line 71-73: "When zoonoses are considered, this also means protecting public health, once contact tracing has the subsequent effect of providing assessments to stakeholders which is useful for quickly identifying and implementing containment, surveillance, and control systems." Rephrase as "once contact tracing has the subsequent effect" does not make sense.

Answer: Thank you for your insightful comment. We have revised the sentence regarding contact tracing for clarity. The updated version now emphasizes its pivotal role in identifying necessary actions to break the transmission chain and protect public health.

Line 85: Delete 'While'

Answer: Done

Line 91: "Currently, due to the COVID-19 global pandemic" Rephrase, "Due to the recent COVID-19 global pandemic"

Answer: Done

Line 99: "it lacks of integration of heterogeneous" Rephrase "it lacks the ability to intergrate heterogeneous..."

Answer: Done

Line 102-103: "but its complexity need for specialized expertise make it less accessible" Rephrase "but its complexity requires specialized expertise making it less accessible..."
Answer: Done

Line 233: Delete "Nowadays, "

Answer: Done

Line 349: "The following case studies were considered:" Consider rephrasing to somthing like "To demonstrate the real-world utility of the application the following case studies were considered:"

Answer: Done

========

REVIEWER 1 --> comment provided at later stage
"Feedback to the authors. I would make the following suggestions:
The interactive demonstration of the application is very useful and the readers should probably be made more aware of its exitance, perhaps by making reference to it in the introduction and abstract?

Answer: We have now referenced the interactive demo of T-Racing in both the abstract and introduction sections.

The legend isn't obvious. It took me some time to find the legend and I couldn't re-size it. Would it be possible to have the legend in English on the demonstration version?

Answer: Thank you for your feedback regarding the legend. One of the improvements we plan to implement is making the legend dynamic so that it remains aligned with the graph content instead of being static as it is now, which will enhance its visibility. In the meantime, we have updated the legend images, translated them into English as requested, and these are now available in the demonstration version of the application.

When selecting nodes on the map, I couldn't find a way to de-select them.

Answer: Thank you for your observation. To deselect a node on the map, simply click anywhere outside the graph in the white space of the right-hand view. This will clear the selection.

For some reason, the application didn't work on Google Chrome (PC, Win 11) but did work on Edge (PC Win 11)"

Answer: Thank you for bringing this issue to our attention. We have successfully verified that the application runs on Google Chrome (PC, Win 11), Version 129.0.6668.70 (64-bit). Please ensure that your version of Google Chrome is updated.

Reviewer #3: Dear authors,
Thank you for sending an improved manuscript version and considering my comments carefully.
My main comment for this round of revision is that you present the tool as an OH tool and, in the beginning, talk about its usefulness to PH agencies. The tool is currently being developed in the AH and used internally for IZS Teramo. That is fine and should be acknowledged from the start.
Check the article where we present a platform developed for a specific purpose (AH in France in our case): [https://journals.plos.org/plosone/article?id=10.1371/journal.pone.0199960](https://urlsand.esvalabs.com/?u=https%3A%2F%2Fjournals.plos.org%2Fplosone%2Farticle%3Fid%3D10.1371%2Fjournal.pone.0199960&e=14a859a8&h=ab18f538&f=n&p=y)
So, I disagree that the tool can currently be useful for PH practitioners unless you mention in the discussion how you share information between ISZ Teramo and ISS Italy, for example, and how you communicate the results of the investigation, etc., to your peers for efficient risk assessment and decision-making (Oh approach)—or other examples of how PH agencies in Italy (e.g., ISS Roma) use this tool currently.
Otherwise, please improve the discussion of how the tool can evolve into an OH tool and provide suggestions on collaboration and sharing information with the PH sectors, for example. This is not a limitation but a perspective and a vision I am sure you have for the tool. So please don’t hesitate to be open and share it with the readers.
With best regards,
I hope you will find my comments constructive.

Thank you for your valuable feedback and for highlighting these important points. We acknowledge that the T-Racing tool is currently used in the context of animal health (AH). However, it is important to emphasize that in Italy, both animal health (AH) and public health (PH) services operate under the Ministry of Health, differently from how this occurs in other countries. This structural integration facilitates a closer collaboration between these sectors, particularly in the context of zoonotic disease management and environmental disaster responses, making it easier to adopt a One Health approach. This is particularly relevant for the Italian system, where PH and AH authorities regularly collaborate during outbreaks, and share information effectively.

Regarding your comment on how T-Racing is currently used, while the tool is still primarily focused on AH data, its integration within the national VetInfo system ensures that it will be accessible to both AH and PH agencies, enabling them to collaborate closely in risk assessment, outbreak investigation, and decision-making processes. We believe that this structural collaboration between public and animal health authorities in Italy—unique to our national system—already aligns with the One Health framework, as it inherently supports interdisciplinary cooperation.

In the future, as suggested, we plan to further develop T-Racing to include more PH data, enhancing its utility for public health practitioners and strengthening its role as a One Health tool. This will allow for the sharing of more comprehensive information between institutes and stakeholders in both the AH and PH sectors. We have expanded the discussion to reflect this vision, highlighting the tool’s potential evolution and future contributions to the One Health approach. An example of the value and efficacy of this collaboration can be found in the following paper:

Sorgentone et al., A large foodborne outbreak of campylobacteriosis in kindergartens and primary schools of Pescara, Italy, May-June 2018. 2021 2021 Journal of Medical Microbiology, 70(3). https://doi.org/10.1099/jmm.0.001262 Epub 2021 Jan 20.

The remaining comments from Reviewer 3 have been addressed directly in the manuscript file in the track changes version. Thank you again for your constructive feedback, which has greatly helped us improve the clarity and direction of our manuscript.
