# Supplementary material for: T-Racing: a modern tool for supporting epidemiological investigation in animal disease outbreaks in Italy
Source: PLoS One. 2025 Feb 19;20(2):e0317313. doi: 10.1371/journal.pone.0317313 (PMC11838889; doi:10.1371/journal.pone.0317313)
Supplement: Attachment — Submitted filename: T-Racing-Response to reviewers comments.docx [file pone.0317313.s004.docx]

Response to reviewers' comments

In blue the authors’ answers to reviewers' comments

**Reviewer #1:** Dear authors,

Thank you for addressing the concerns and observations I made in the initial review of this paper. In my opinion, you have greatly improved the manuscript. I reiterate on my past comment that this work isn't unique to Italy. Many other countries build their own tracing tools. However, this is usually done by IT professionals under contract and rarely gets shared to the wider scientific community which is why I'm pleased you have gone to the effort to seek to publish your work. As this is not a conventional research paper, it doesn't quite fit the template for presenting your methodologies and results. You have improved the structure and layout in this version of the manuscript and I've added some more minor edit suggestions below:

Thank you for your positive feedback and for acknowledging the improvements made to the manuscript. We appreciate your recognition of the importance of sharing our work with the wider scientific community. We have carefully reviewed and incorporated all the additional edit suggestions you provided.

Line 20: delete ‘Therefore’ Answer: Done
Line 21: replace “and almost in real-time” with “in near real-time” Answer: Done
Line 35: delete “emergency” Answer: Done
Line 45,46. Delete “On the other hand”. Answer: Done
Line 212: Replace “In the end, such kind of strategies” with “Ultimately, such strategies” Answer: Done
Line 213: Delete “and” from “(and thus) Answer: Done
Line 233: Add “The”… “The T-Racing interface” Answer: Done
Line 234: “graphs, maps,” Answer: Done
Section 2.3 is poorly laid out in terms of flow of information. I would suggest a adopting clearer headings and subheadings rather than multiple descriptions followed by bullet points.

Answer: Thank you for your valuable feedback we have revised this section to improve the flow of information and make it clearer for the reader. Specifically, we have reorganized the content into distinct subheadings to better structure the information and avoid lengthy descriptions followed by bullet points.

The revised section now includes three subheadings:

2.3.1 Tools for extracting and viewing animal trade data

2.3.2 Tools for customizing network visualization and optimization

2.3.3 Tools for epidemiological information integration and results printing

Each subheading clearly groups related functionalities and describes them in a concise and organized manner. We believe these changes address your concerns and significantly enhance the readability and coherence of the section.

We hope that the revised structure meets your expectations.
Line 255: Replace “make” with “select” Answer: Done

Section 2.6 Demo and 3. Results.
[https://demo.izs.it/app_direct/tracing/](https://urlsand.esvalabs.com/?u=https%3A%2F%2Fdemo.izs.it%2Fapp_direct%2Ftracing%2F&e=14a859a8&h=c326cb4b&f=n&p=y)
I can access the interface but can’t select any dummy data.

Answer: We apologize for the inconvenience. It appears that other reviewers did not encounter this issue. However, if you're interested, please feel free to contact us directly, and we'll be happy to assist you in resolving the problem.
Line 503: “inform the response in near real-time and to allow” Answer: Done
Line 427: Replace “focus” with “focused” Answer: Done
Line 533: I would suggest changing “Complementarily,” to “Coupling”.

Answer: Thank you for your suggestion to replace “Complementarily” with “Coupling” in line 533. After careful consideration, we chose to retain “Complementarily” because it more accurately conveys our intention to highlight how molecular epidemiological data enhances and completes the analysis, rather than simply combining elements. To better express this concept, we have rephrased the text as follows: “The integration of molecular epidemiological data into the analysis complements the validation of identified disease (brucellosis) transmission paths, exemplifying a One Health approach by a multidisciplinary team to advance disease control technology.” (in revised lines 540-543).
Lines 533 – 550. This is mostly repetition and overly wordy. Is it necessary or could it be replaced with a more simple and humble sentence?

Answer: Thank you for your valuable feedback. The paragraph in question has been revised and streamlined to address your concerns regarding repetition and wordiness. We have simplified the language while preserving the essential points of the original content (in revised lines 540-551).

**Reviewer #3:** Dear authors,
Thank you for providing an improved version of the manuscript.
I provide my detailed comments in the reviwed manuscript attached.

Thank you for your detailed feedback and suggestions. We have addressed all your comments directly in the revised manuscript. Our responses are provided in the comment sections under each request.

I have several general suggestions:
- you should clearly mention that the tool is not open source and freely available and say why

Answer: We have explicitly mentioned in the revised manuscript that T-Racing is not open-source or freely available due to the sensitive nature of the data it handles, which comes from Italian national information systems. This clarification has been added to ensure transparency regarding the tool's accessibility. Regarding why the tool is not yet open-source, it's because all the developed functionalities, from data visualization to the various integrated analyses and services, are tightly linked and tailored to the specific realities of the Italian animal trade system. This means that additional effort and time would be required to decouple these functionalities and make the tool suitable for sharing as an open-source solution.

- you dont discuss the limitations of the tool - this should be clearly addressed in the discussion

Answer: The limitations of T-Racing were previously discussed in the manuscript; however, we have now made these points more explicit in the discussion section. We have clearly addressed the tool’s current limitations, including its specificity to the Italian context and the restriction of genetic data to the Brucella genus. These updates aim to ensure that the limitations are fully transparent to the reader.

-if the tool is generic should be underlined as an advantage and say how it can be of use one day for other diseases and other services in Italy, for eample One health, as you mentuon this in the discussion

Answer: The manuscript has been updated to emphasize that T-Racing is designed to be a versatile tool. While it is currently applied within the Italian context, it already manages data for around 100 different diseases, including zoonotic diseases such as brucellosis, tuberculosis, leptospirosis, rabies, salmonellosis, campylobacteriosis, listeriosis etc. Its structure allows for potential expansion to include additional data and services, particularly those relevant to One Health. This adaptability is now highlighted as a key advantage in the discussion, demonstrating the tool’s potential for broader applications in the future.

Kind regards

**Reviewer #5:** The subject of the article is of interest.
I have revisions to suggest to authors and some questions, see below:

Thank you for your feedback and for finding the subject of the article interesting. We appreciate your comments and have addressed them in detail below in response to your questions.

Introduction section
Line 47: specify that 2.1 million people is worldwide (I suppose it is that).

Answer: Thank you for your suggestion. The phrase states that the 2.1 million people is globally affected, which has the same meaning as worldwide, that was not used to avoid repeat the same word in sequential phrases.

Line 54-60: Is it really relevant to have this description of TRACES-NT because this databasis is not used in the article? If it is maintained, it should be specified that it is only accessible to competent authority and considered as sensitive data.

Answer: Thank you for your observation. We included a description of TRACES-NT in the introduction to emphasize the importance of tracing systems in epidemic contexts and the relative collection of trade data. Although TRACES-NT is not used by T-Racing, it serves as an example of a relevant database for illustrating the broader landscape of traceability in disease control. We acknowledge that TRACES-NT is not freely accessible and is only available to competent authorities and in the revised manuscript, we have clarified this by adding the statement "The system is only accessible to competent authorities and is considered sensitive data" in revised line 59, as suggested. Moreover, we highlighted in the revised manuscript that T-Racing relies on Italian national databases for its operations. Thank you for pointing out the need for this clarification.

Material and method

• I was surprised that no wildlife data (wildlife TB and brucellosis positive cases) were considered for TB and brucellosis. Are these data not available? Is it a possibility to improve the tool for future? It would have also justified the “one health” approach cited in the article.

Answer: We appreciate your question and suggestions. We would like to clarify that data on wildlife regarding brucellosis and tuberculosis are not available at the moment and no surveillance is systematically conducted in wildlife in Italy regarding these diseases that were used here just as an example of the functionality of the application. We understand your point of view regarding “One Health” and We have reduced its usage throughout the manuscript and replaced it with "multidisciplinary approach" where appropriate, however, we respectfully would like to clarify, that the approach considered in the paper for “One Health” has to do with the prevention of zoonosis by tracing the paths of positive animals, avoiding dissemination. To perform the construction of the application and the interpretations of its results, a multidisciplinary team was necessary. The original concept of “One Health” proposes that multidisciplinary teams work together to face the challenges to control diseases and decrease the burden imposed by them, and our application was the result of this approach. Regarding your specific concern on the importance of wildlife data, it is important to note that outbreaks managed in T-Racing (provided by SIMAN system) can include not only farm-based cases but also positive cases in wildlife. These positive cases can be used as seeds and buffers to include the commercial network, as explained in the manuscript. Wild boar data for ASF (African Swine Fever) have already been integrated into the system as cases (outbreaks) and serve as an example of how T-Racing manages wildlife data. Thank you for raising this important issue.

• How rendering plants and slaughterhouses are considered in the tool, are they considered in the same way as a farm in the network analysis? Idem for transit section that are cited in the result section (line 381) but we don’t have information on how it is considered in the tool in the material and method section.

Answer: Thank you for your query. In T-Racing, rendering plants and slaughterhouses are treated as distinct attributes within the network analysis. These facilities are represented as nodes within the network, with specific shapes and labels used to identify and highlight their roles. This approach ensures that these structures, which are integral to the animal trade system and of veterinary interest, are clearly distinguished in the analysis. Similarly, transit stations are also considered in the tool. They are included as part of the network analysis and are marked accordingly to reflect their function and relevance in tracing disease outbreaks. This inclusion helps to identify and analyze patterns of disease spread within the entire animal trade network (as stated in lines: 165-168 and 289-293 of the revised manuscript).

• Line 242-243: the tool considere spatial buffer using distance by kilometers. Do you think of using pasture data to improve your tool in the future?

Answer: Thank you for your question. We would like to clarify that nodes described as pasture were used in the dataset as part of the network data. Pastures are represented as points, and you can apply a buffer to account for the spread to neighbouring holdings.

• Line 330-331 and 340: how these parameters were determined? Taking into account movements only over the past 13 months seems for instance restrictive considering TB, a chronic infection that can be difficult to detect. Was it based on experts’ elicitation?

Answer: Thank you for your question. The considered case study and parameters, such as the time interval and depth levels, are intended to illustrate the application of T-Racing rather than to serve as definitive measures for all analyses. In this example, the parameters were chosen based on the data available and were set iteratively to best highlight secondary outbreaks. The time interval of 13 months and depth levels were adjusted according to the specific context of the study, informed by the nature of the disease and the data constraints. These parameters are not fixed but can be tailored by users based on their expertise and the specific requirements of their investigations. For chronic infections like tuberculosis, which can be difficult to detect and may have longer incubation periods, users can adjust these parameters to extend the analysis period and depth as needed for more comprehensive investigations.

• Line 350: considering TB as important zoonosis seems not adequate. It has important economic impact due to movement and commercial restrictions for farmers but in Europe TB human cases are not anymore due to bovine tuberculosis as far as I know. Is there any publication that can support this zoonotic aspect in European countries? It seems to me that it is not a big issue for human health anymore.

Answer: Thank you for your consideration. In Europe TB is indeed not important as zoonosis, however in other countries, the reality is quite different. T-Racing was developed specifically for Italy, but its design allows for adaptability and potential use in other countries. We propose this application as a solution for other official veterinary services globally, which is why TB was included as an important zoonosis. To clarify, we have revised the text to state: “Brucellosis caused by Brucella abortus is an important zoonosis worldwide, and tuberculosis caused by Mycobacterium bovis constitutes a serious hazard to public health in developing countries and a great economic burden to livestock production in Europe. These diseases were chosen due to their public health and economic importance and their chronic development, allowing for the use of FCT and BCT methods with a broad time-elapse.” Additionally, we highlighted in the discussion session that T-Racing will become open source and will be capable of incorporating proprietary data.

Results

• Line 471: it seems here that pasture data are included but I can’t find this information in the material and method section.
Answer: Thank you for your comment. To comply with your comment we add pasture to the holding type in the methods section. Line: 134 to 136: “…such as the holding type (e.g., farm, slaughterhouse, staging point, transit station, market, assembly centre, foreign country, pasture, etc.),…”

Discussion
Several times in the article, and especially in the discussion section, the “One health” approach is cited but I can’t see how the tool could be considered as a One health approach tool at that stage.

There is non-human health data which could be relevant only for brucellosis. For BT it is relevant to consider only human cases due to bovine tuberculosis due to contamination in Italy which I suppose is very rare, nearly no case no? If data on human cases due to bovine tuberculosis and brucellosis are available it would be great to add it in the introduction section. If BT is of importance on a zoonotic aspect, authors should consider to improve the tool in the future, to add information on farm production type so as to distinguish raw milk production farms.

At that stage there is no data on wildlife surveillance considered which could have justified the one health approach with the environmental sector.

Answer: Thank you for your comment. We have reduced its usage throughout the manuscript and replaced it with "multidisciplinary approach" where appropriate

It is important to note that the majority of zoonotic diseases are controlled in humans by managing them in animals. The application presented in the paper serves as a critical surveillance tool for tracking animal diseases, which is a key component of the One Health approach. Effective tracing allows official veterinary services to intervene and break the chain of disease transmission, which is crucial for controlling and preventing the spread of diseases. For example, interventions such as the slaughter of infected animals and increasing public awareness about the risks associated with raw milk can help prevent the transmission of diseases to humans. In this sense, the application supports the One Health approach by contributing significantly to disease control in animals, which in turn protects human health. Additionally, the tool was developed by a multidisciplinary team with the goal of preventing diseases, which aligns with the One Health perspective. It is important to note that the application’s objective - focused on preventing disease spread and protecting both animal and human health- aligns with the One Health approach, rather than the specific data used. As previously highlighted in responses to reviewers, achieving a One Health state requires coordinated efforts across animal, environmental, and human health. This does not necessarily mean addressing all aspects simultaneously but involves steps to prevent disease spread from animals to humans. This focus on safeguarding animal health to protect human health is central to the One Health approach.

First, we would like to clarify that the diseases used in the paper were chosen as examples of how the application works. Indeed, these diseases have different importance considering the zoonotic aspect in Europe, however, the data used here (as an example) was from 2022 and 2023 about tuberculosis identified in bovines and this is a concern for human health because the zoonotic potential of the disease exists. Therefore, rigorous surveillance should be applied when cases occur in bovines, to prevent the disease to spread, preventing transmission to humans. Regarding the production type of the farms, it is presented in the data as milk and beef production, and all milk produced is raw milk and it is transported to the dairy industry to be distributed to the population after pasteurization.

Even though wildlife data are limitated, the One Health approach is justified by other points, such as using a technological tool, that contributes to disease control and benefits different professionals such as clinicians, veterinarians, ecosystem health professionals, researchers, and policymakers to leverage the power of animal surveillance. As previously mentioned, the system includes all reported cases of various animal diseases (both zoonotic and non-zoonotic), which can also affect wildlife, as seen with diseases like African Swine Fever. We have made efforts to better describe the outbreak data and diseases contained in the database in the paper.
